# Phosphoproteomics-Based Modeling Defines the Regulatory Mechanism Underlying Aberrant EGFR Signaling

**DOI:** 10.1371/journal.pone.0013926

**Published:** 2010-11-10

**Authors:** Shinya Tasaki, Masao Nagasaki, Hiroko Kozuka-Hata, Kentaro Semba, Noriko Gotoh, Seisuke Hattori, Jun-ichiro Inoue, Tadashi Yamamoto, Satoru Miyano, Sumio Sugano, Masaaki Oyama

**Affiliations:** 1 Medical Proteomics Laboratory, Institute of Medical Science, University of Tokyo, Tokyo, Japan; 2 Department of Medical Genome Sciences, Graduate School of Frontier Sciences, University of Tokyo, Tokyo, Japan; 3 Human Genome Center, Institute of Medical Science, University of Tokyo, Tokyo, Japan; 4 Department of Life Science and Medical Bio-Science, Waseda University, Tokyo, Japan; 5 Division of Systems Biomedical Technology, Institute of Medical Science, University of Tokyo, Tokyo, Japan; 6 Division of Cellular Proteomics (BML), Institute of Medical Science, University of Tokyo, Tokyo, Japan; 7 Department of Biochemistry, School of Pharmaceutical Sciences, Kitasato University, Tokyo, Japan; 8 Department of Cancer Biology, Institute of Medical Science, University of Tokyo, Tokyo, Japan; Keio University, Japan

## Abstract

**Background:**

Mutation of the epidermal growth factor receptor (EGFR) results in a discordant cell signaling, leading to the development of various diseases. However, the mechanism underlying the alteration of downstream signaling due to such mutation has not yet been completely understood at the system level. Here, we report a phosphoproteomics-based methodology for characterizing the regulatory mechanism underlying aberrant EGFR signaling using computational network modeling.

**Methodology/Principal Findings:**

Our phosphoproteomic analysis of the mutation at tyrosine 992 (Y992), one of the multifunctional docking sites of EGFR, revealed network-wide effects of the mutation on EGF signaling in a time-resolved manner. Computational modeling based on the temporal activation profiles enabled us to not only rediscover already-known protein interactions with Y992 and internalization property of mutated EGFR but also further gain model-driven insights into the effect of cellular content and the regulation of EGFR degradation. Our kinetic model also suggested critical reactions facilitating the reconstruction of the diverse effects of the mutation on phosphoproteome dynamics.

**Conclusions/Significance:**

Our integrative approach provided a mechanistic description of the disorders of mutated EGFR signaling networks, which could facilitate the development of a systematic strategy toward controlling disease-related cell signaling.

## Introduction

EGFR is a receptor tyrosine kinase that is widely expressed in epithelial tissues and plays important roles in information transfer from extracellular signals to the intercellular region, regulating many biological activities such as cell proliferation, differentiation, and survival. There is some evidence that the mutation of EGFR triggers the deregulation of the EGFR signal transduction system and is strongly associated with abnormal cell behavior [1], [2]. Therefore, the necessity to gain a deeper insight into mutation-initiated aberrant signaling has emerged as a major concern to understanding the ErbB signaling networks. However, the mechanism by which EGFR mutation alter downstream signaling is not yet completely understood at the system level mainly because of the absence of established methodologies to generate and analyze quantitative information on mutant EGFR signaling on a network-wide scale. Thus, an integrated platform is required for evaluating the system-level properties of cell-specific signaling dynamics.

In recent years, there have been great improvements in proteome analysis using tandem mass spectrometry coupled with liquid chromatography (LC-MS/MS) technology, thereby enabling large-scale identification of peptides and some types of protein modifications [3], [4]. Moreover, the establishment of protein labeling methods has enabled the quantitative measurement of proteins and peptides in samples on a proteome-wide scale [5], [6]. Recent time-course activation data from the LC-MS/MS experiments have provided a global view of EGFR signal transduction systems [7], accelerating system-level understanding of signal processing based on numerical and statistical analyses [8]–[13].

Because the complexity of biological networks prevents the intuitive understanding of signaling networks, many numerical representations of signal transduction, particularly in the ErbB signal transduction system, have also been investigated [14]–[27]. In our recent study, we constructed a numerical model of EGFR signaling based on the hybrid functional Petri net with extension (HFPNe) [25]. HFPNe is a computational modeling architecture that can describe not only continuous events but also discrete events [28]–[32] and enables the analysis of temporal data on biological entities within the data assimilation framework [33]–[35]. The data assimilation framework was originally developed and successfully implemented in geophysics to predict geological phenomena such as El Nino-Southern Oscillation by integrating a high-dimensional computational model and limited observed data [36] and is considered to be applicable for the construction of a reliable signal transduction model using time-dependent phosphoproteomic data.

EGFR signal transduction is initiated by receptor autophosphorylation triggered by ligand binding. Phosphorylated EGFR (pEGFR) serves as an adaptor for cellular proteins that can recognize phosphorylated tyrosine residues and subsequently catalyzes tyrosine phosphorylation of recruited proteins. Recently, comprehensive in vitro analyses of binding proteins for each autophosphorylation site in the ErbB family receptors were conducted using mass spectrometry and protein microarray [37]–[39]. The results from the protein microarray analysis indicated that phosphorylated Y992 (pY992) bound to multiple cellular proteins, serving as a multifunctional docking site of EGFR [38]. Under in vivo conditions, Y992 has been shown to bind to several EGF signaling modulators such as phospholipase C gamma 1 (Plcγ1) [40], Vav2 [41], and RAS p21 protein activator (RasGAP) [42], and also to act as a dephosphorylation target of protein tyrosine phosphatase, non-receptor type 1 (PTP1B) [43], and protein tyrosine phosphatase, non-receptor type 11 (Shp2) [44]. Of these EGF signaling modulators, Plcγ1 and Vav2 are known as positive regulators of mitogen-activated protein kinase (MAPK) pathway, whereas RasGAP is known to negatively regulate the MAPK pathway by enhancing the catalytic activity of Ras. Therefore, mutation at Y992 of EGFR would be expected to cause complex bidirectional effects on downstream signaling networks, and is particularly suitable as a model system to evaluate the performance of our approach.

Here, we report a novel phosphoproteomics-based framework to analyze the system-wide effect of single point mutation at Y992 of EGFR. We measured EGF-induced temporal activation of tyrosine phosphorylation-mediated signaling in two NIH3T3-derived cells expressing either wild-type EGFR (WT) or mutant EGFR with substitution of tyrosine to phenylalanine at position 992 (Y992F) (the numbering system excludes the 24 amino acid signal peptide of EGFR). The phosphotyrosine-dependent proteome dynamics in these two cell types characterized an unbiased landscape of the aberrant Y992F signaling. On the basis of the quantitative profiles, our computational modeling approach described the quantitative differences in EGFR signaling between WT and Y992F cells, presenting potential factors for generating the aberrant signaling dynamics.

## Results and Discussion

### Identification and Quantitation of EGF-Induced Phosphoproteome in WT and Y992F

**Figure 1:**
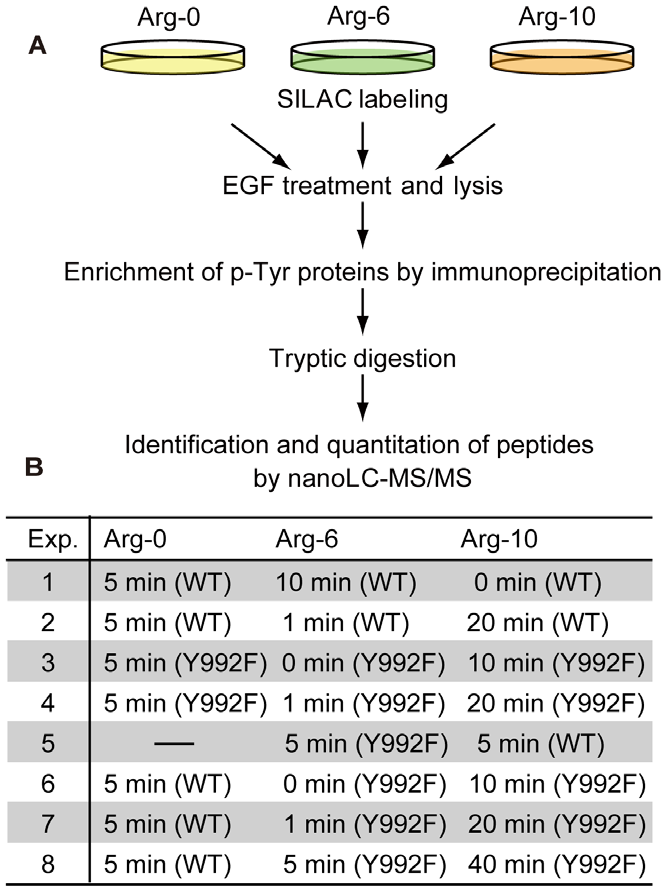
Strategy for measuring the quantitative behaviors of EGF-induced tyrosine phosphoproteome in WT and Y992F cells. **A**. Flowchart for SILAC experiment. Each cell population encoded with isotopically labeled arginine (Arg-0, Arg-6, or Arg-10) is stimulated with EGF for the time intervals indicated. Tyrosine phosphoproteins are enriched from equally mixed cell lysates using phospho-tyrosine specific antibodies. The purified complexes were digested in solution and directly applied to nano-flow LC-MS/MS system for protein identification and quantitation. **B**. An experimental design for acquiring temporal profiles of tyrosine phosphoproteome upon EGF stimulation. Temporal profiles were generated through integration of eight independent mass spectrometric measurements. For each time period, relative quantitative values were normalized to the values for WT at 5 min.

To elucidate the global differences in EGF-induced signaling dynamics between WT and Y992F cells, we performed quantitative proteomic analysis of phosphotyrosine-dependent signaling molecules. Stable isotope labeling by amino acids in cell culture (SILAC) was applied for time-dependent comprehensive quantitation of phosphotyrosine-dependent proteins using a nano-flow LC-MS/MS system as previously described [45] (Figure 1A). From the eight independent measurements (Figure 1B), 383 peptides were identified and assigned to 147 proteins in total (Table S1). Relative quantitation of the identified proteins was performed using the AYUMS algorithm [46] and MSQuant software [47] (Figure S1, Table S1). For each identified protein, relative activation values in each measurement were combined and normalized by that of WT at 5 min of EGF stimulation (Table S1). In order to extract EGF-dependent molecules of phosphotyrosine signaling, we adopted 1.5 as the threshold of fold change for the time-course activation data on either of the two cells. In this criterion, 41 proteins were extracted (Table S1) and used for further computational analyses. Among the 41 proteins, 15 proteins were quantified by a single peptide and 26 proteins were quantified by multiple peptides (Table S1). All experimental data were generated for this study using the methods described in our prior publication [45].

### Temporal Profiles of Phosphotyrosine-Dependent Proteome Reveal the Global Impact of the Mutation at Y992

**Figure 2:**
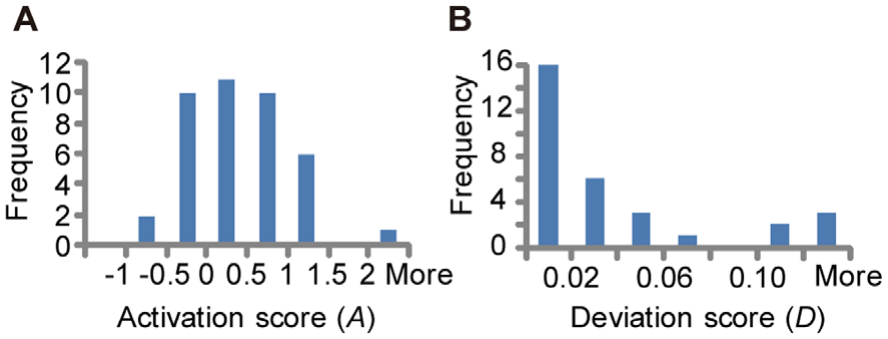
Distinct signal properties regarding WT and Y992F cells. **A**. The differential phosphorylation status between WT and Y992F cells. The distribution of the activation score (*A*) of each protein, which indicates the fold-change of phosphorylation amount of Y992F versus that of WT. **B**. The differential phosphorylation dynamics between WT and Y992F cells. The distribution of deviation score (*D*) of each protein, which indicates the deviation of the phosphorylation pattern.

To reveal the differences in signal transduction between the two cells, we calculated two types of scores—activation score (*A*) (Figure 2A) and deviation score (*D*) (Figure 2B) —which represent the differences in the amount of phosphotyrosine-dependent proteins and the deviation of the temporal pattern, respectively (see Methods section and Table S1). As shown in Figure 2A, the activation level of most of the proteins was increased or unchanged in Y992F cells compared to that in the WT cells, although that of EGFR was slightly decreased. Regarding temporal pattern, a limited fraction of molecules showed distinct pattern changes between the two cell types (Figure 2B). Modulators of EGFR trafficking such as HGF-regulated tyrosine kinase substrate (Hrs), zinc finger, FYVE domain containing 16 (ZFYVE16), and signal transducing adaptor molecule (SH3 domain and ITAM motif) 2 (STAM2) showed differences in the peak time or amplitude of activation; this clearly indicated that Y992F affected the regulation of the EGFR degradation pathway. Moreover, sustained activation of extracellular signal-regulated kinase 1 (ERK1) was remarkably observed in Y992F cells, whereas it was transiently activated in WT cells. This apparent pattern conversion was not observed in the upstream positive effectors of ERK1, such as EGFR, Grb2, Shc, Shp2, and Plcγ1.

### Construction of EGFR Signal Transduction Model Based on the HFPNe Architecture

**Figure 3:**
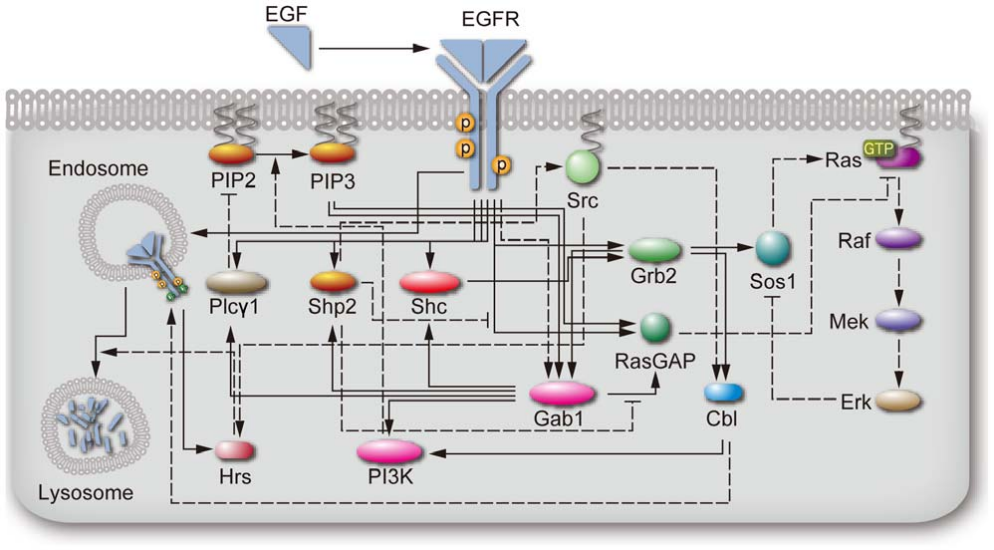
Schematic representation of HFPNe-based EGFR signal transduction model. Solid-line and dashed-line arrows denote direct physical interactions and indirect associations or catalytic reactions, respectively.

The time-dependent profiles of the EGF-induced phosphoproteome measured in the WT and Y992F cells revealed their qualitative and quantitative differences at the network level. To elucidate the mechanisms underlying these alterations as a system, we performed dedicated computational analysis using biochemical simulation based on the HFPNe architecture. The computational model for this study was essentially built based on the EGFR models previously reported [25], [26] with some modifications and extensions using information from published literature (Material S1). Our HFPNe-based EGFR model consists of sequential activation of EGF signaling networks from EGF stimulation to activation of the canonical MAPK cascade (Figure 3). To investigate the regulatory mechanisms underlying EGFR degradation pathway, we performed detailed modeling of the processes for ubiquitin modification of EGFR and its sorting from the plasma membrane compartment to the lysosomal compartment. The detailed specification of the model is shown in Figure S2, Table S2, and Table S3 and the electrical format of the model can be found in Material S2.

**Figure 4:**
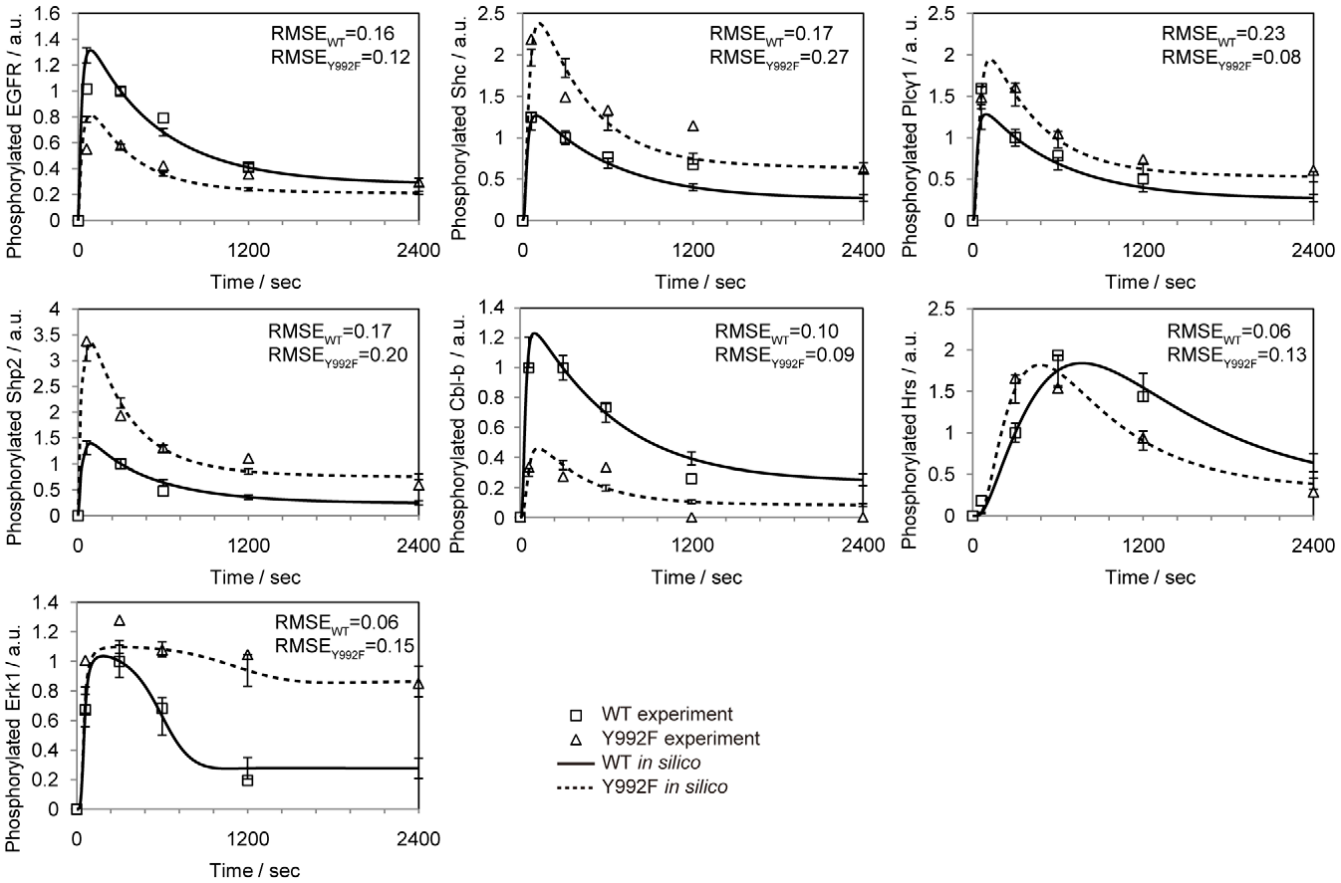
Comparison of simulation results with experimental data. The solid and dashed lines represent the averaged simulation results from the WT and Y992F parameter distributions, respectively. The squares and triangles represent the normalized experimental data on WT and Y992F, respectively. Root mean square error (RMSE) for each protein is indicated.

The parameters in our EGFR model were optimized using a sequential Monte Carlo method known as a particle filter as described previously [33] to generate the temporal activation dynamics of EGFR, Shc, Plcγ1, Hrs, Casitas B-lineage lymphoma b (Cbl-b), Shp2, and ERK1 measured by quantitative phosphoproteomics (detailed procedures of parameter estimation can be found in Material S1 ). Because our model contains many free parameters, it is extremely difficult to estimate probabilistic distributions of all parameters simultaneously within a feasible time. Then, we conducted two-step parameter optimization. In the first global optimization process, multiple parameters were varied at the same time within the narrow range to estimate the global parameter distributions. Next, local parameter distributions were estimated by applying a particle filter to each parameter within the broad range, while other parameters were varied according to the probabilistic distributions obtained by the global optimization process. We performed global optimization of 102 parameters to generate the phosphorylation dynamics of EGFR, Shc, Plcγ1, Hrs, Cbl-b, Shp2, and ERK1 in the WT cells. Next, we estimated the global parameter distributions of the Y992F model on the basis of those in the WT model. In all, 64 parameters in the WT model were fixed, and the remaining 54 parameters regarding the initial abundance of signaling molecules, the binding processes of EGFR to the related proteins, the phosphorylation processes catalyzed by EGFR, and the EGFR regulation pathway were varied for further parameter optimization. Finally, we estimated probabilistic distribution of each parameter in the WT and Y992F model (Figure S3). The final simulation results with the estimated distribution of parameters accurately reproduced the experimental data on both the WT (RMSE = 0.16) and Y992F cells (RMSE = 0.16) (Figure 4).

### Verification of the model accuracy

To evaluate the performance of our model, we compared the significant parameter differences between the WT and Y992F models with the already known properties of Y992F signaling described in the literature. We defined 13 of up or down regulated parameters that satisfy both fold-change≥2.0 and p-value≤0.001 simultaneously (Table 1). Our model suggested the increase in the dissociation constants of Plcγ1 from pEGFR. This result is consistent with those of the previous studies indicating that Plcγ1 preferentially bind to pY992 [40]. Regarding EGFR internalization dynamics, Y992F mutation is known to increase the rate of receptor internalization [48], and Y992-non-phosphorylated EGFR undergoes internalization more rapidly than Y992-phosphorylated EGFR [49]. In agreement with the above biological evidence, our model showed that the EGFR internalization rate was higher in the Y992F model than in the WT model (Table 1). On the other hand, our model did not clearly indicate corresponding differences of parameters regarding the interactions of EGFR with RasGAP and Shc [42], [38]. These results would reflect uncertainties of these parameters indicating that additional data is required for constraining the parameters. Our computational approach successfully captured a part of the already-known biological consequences regarding Y992F mutation without over-interpretation of the experimental data.

**Table 1:** Up or down regulated parameters with at least two-fold change of the mean value for each parameter distribution (p-value less than 0.001).

Description	Log_2_-fold change
**Up-regulated parameter**	
EGFR internalization*	4.1
Shp2_phosphorylation	3.6
EGFR_ubiqutination	3.0
EGFR_Plcγ1_dissociation*	2.1
Shc_phosphorylation	1.5
PI3K_abundance	1.1
Gab1_phosphorylation	1.1
EGFR_deubiquitination	1.1
**Down-regulated parameter**	
EGFR_dimer_dissociation	−1.5
EGFR_binding	−1.1

Parameters associated with the literature are indicated with an asterisk.

### Parameter-Based Discovery of the Critical Reactions Governing Cell-Specific Signaling

**Figure 5:**
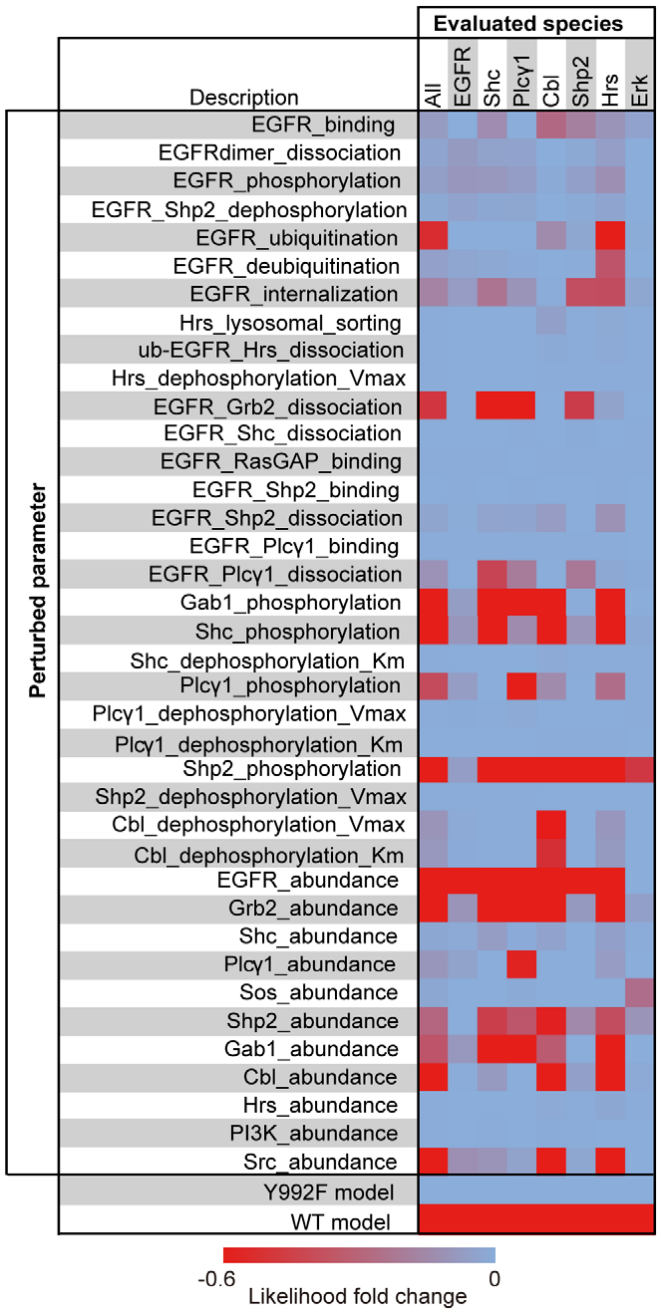
Parameter-based discovery of the critical reactions governing Y992F signaling. Each of the 38 estimated parameters in the Y992F model was reset to the value in the WT model, and the likelihood for the measured data was evaluated. The color bar shows the log-transformed fold change of the likelihood against the value in the original Y992F model. The likelihood for all the observed data was calculated as a geometric average of the likelihood for the data for each protein. The likelihood regarding the WT and Y992F models is indicated as positive and negative controls, respectively.

In the complex biological system, there are robust and fragile parameters against the system behavior [50]. Hence, the magnitude of fold change between the two models does not reflect the importance of the parameters themselves. Thus, we evaluated the importance of the 38 parameters that showed significant difference (p-value≤0.001) between the WT and Y992F models on the basis of phenotypical impact. Each of the parameters in the Y992F model was reset to the value in the WT model. Next, we calculated the likelihood of the model to the observed activation levels, which we termed as local parameter influence (LPI) analysis. The result of our analysis revealed that most of the parameters had a small effect on the re-simulation results, whereas a few parameters had a great impact on the system behavior (Figure 5, Table S4). Of the evaluated parameters, 12 parameters mainly governed the activation dynamics of all the observed proteins in the Y992F model. Of these 12 parameters, 6 defined the initial abundance of EGFR, Src, Shp2, Grb2, Cbl, and growth factor receptor bound protein 2-associated protein 1 (Gab1); 4 indicated the phosphorylation rates of Shc, Gab1, Shp2 and Plcγ1; and 2 indicated the binding constant of Grb2 to pEGFR and the rate of EGFR ubiquitination.

### Effect of Cellular Content on EGF Signaling Dynamics

**Figure 6:**
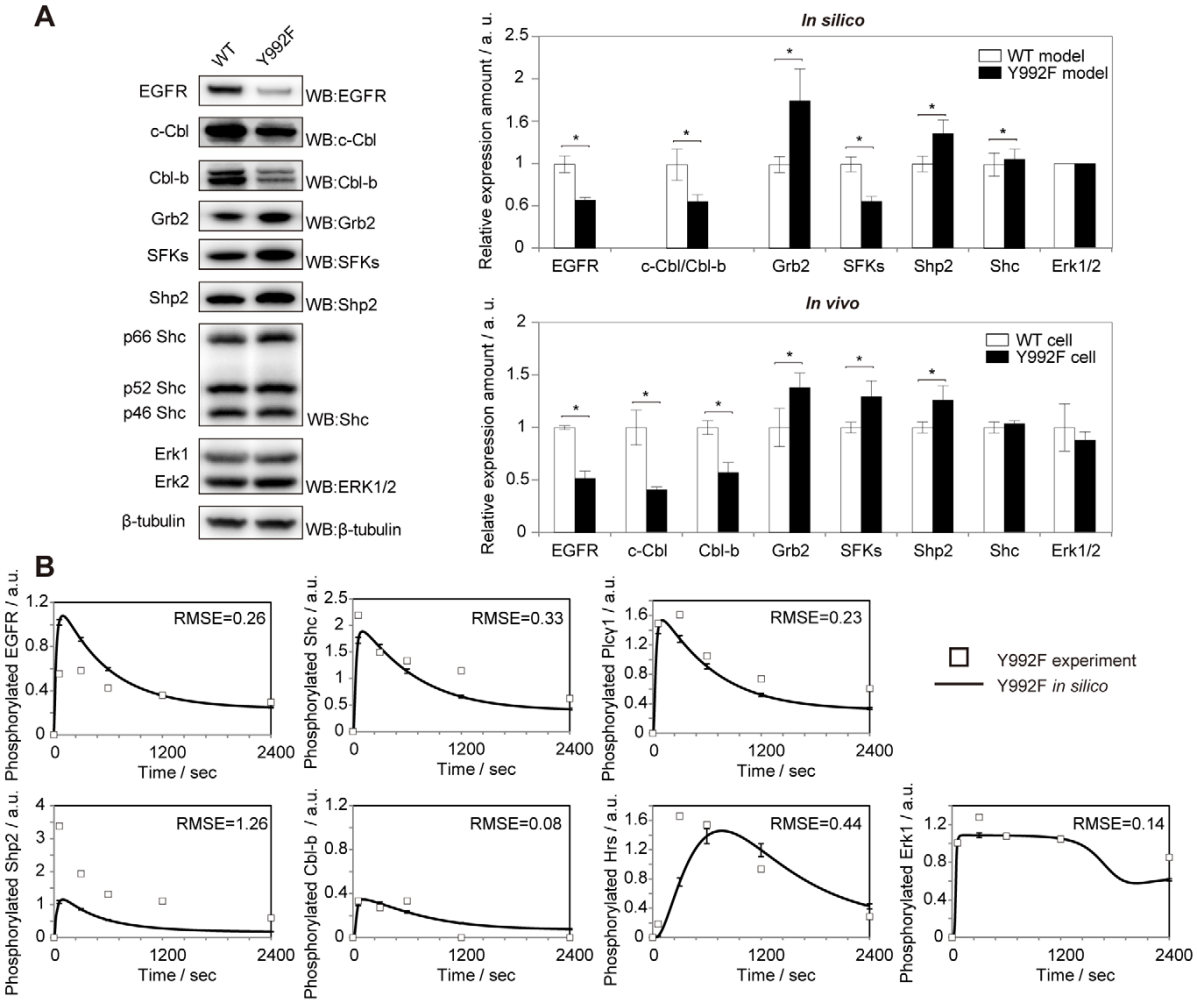
Analysis of the effect of the cellular content on EGF signaling dynamics. **A**. Experimental validation of relative expression level of model species. Upper right panel: predicted parameter value regarding the initial concentration of model species. Error bars represent standard deviations of 1,000 samples from an ensemble of the WT and Y992F models. Left and lower right panel: western blot analysis of relative concentration of model species. Unstimulated cell lysates of the WT and Y992F cells were dissolved by SDS-PAGE and probed using anti-EGFR, anti-Cbl, anti-Cbl-b, anti-Grb2, anti-Src, anti-Shp2, anti-Shc, anti-Erk1/2, and anti-β-tubulin as a loading control. Quantitated band intensities were normalized to the values for WT. With regard to Shc and Erk proteins, the intensities of three bands of Shc isoforms and two bands of Erk1 and Erk2 were combined, respectively, for the calculation of the relative protein amount. Error bars represent standard deviations of triplicate samples. *P<0.05 (unpaired *t*-test). **B**. The simulation results with the best estimated parameters regarding molecular abundance. We performed constrained parameter estimation where the abundance of all the model species was allowed to change within 0.1–10 fold. The parameter set with 1.1×10^−6^ of the likelihood was selected as the best one through 60 steps of calculation. The solid line represents the simulation results of the model with the best parameters. The squares represent the experimental data on the Y992F cells.

With regard to initial protein abundance, the magnitude of fold change between the two models was relatively small, but it had more effect on the simulation results than those of the other parameters. Then, we measured the differences in the total protein abundance of EGFR, c-Cbl, Cbl-b, Grb2, Src family kinases (SFKs), Shp2, Shc, and Erk1/2 between WT and Y992F cells in order to compare the results with those predicted by the in silico model. This analysis revealed that almost all the protein species, except SFKs, showed good correlations (Pearson's correlation coefficient = 0.94, p-value = 0.002) between in silico and in vivo (Figure 6A). These results strongly suggest that the distinct Y992F cell signaling dynamics depend on the differences in cellular context between the two cells to some extent. Next, we further examined the effect of total protein abundance on the signal dynamics of Y992F by using our EGFR model. The parameters that define the initial abundance of biological species in the WT model were randomly varied between 0.1 and 10 fold in order to reproduce the temporal activation data on Y992F cell signaling. Constrained Y992F model estimation revealed that the alterations in the total protein abundance alone could not completely reproduce the signal dynamics of Y992F (RMSE = 0.54) (Figure 6B). Thus, we speculated that the reactions defined by the other six parameters were mainly governed by pY992 in the short-term EGFR signaling.

### Model Prediction Reveals Quantitative Differences in EGFR Degradation Pathway

**Figure 7:**
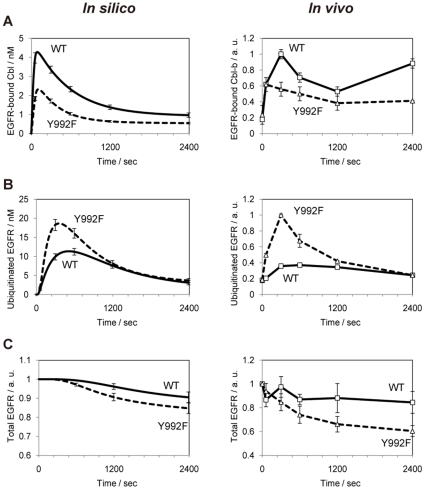
Comparison of in silico (left) with in vivo (right) dynamics of EGFR degradation pathway. **A, B**. The temporal dynamics of EGFR-bound Cbl-b and ubiquitinated EGFR. Extracted proteins were normalized to the initial expression amount of EGFR, and then subjected to EGFR immunoprecipitation. Immunoblottings were performed to detect co-immunoprecipitated Cbl-b and ubiquitinated EGFR. Error bars represent the deviations of two independent experiments. The simulated results were normalized to the initial abundance of EGFR predicted in silico. **C**. The temporal dynamics regarding the total amount of EGFR. EGF-stimulated cell lysates were applied to immunoblots to detect total EGFR protein. Band intensities and simulated results were normalized to the value at zero time point for each cell type. Error bars represent standard deviations of three independent experiments. The raw data on the immunoblot experiments can be found in Figure S4.

Our LPI analysis revealed that the increase in EGFR ubiquitination rate in Y992F model only influenced Cbl and Hrs (Figure 5), which are EGFR degradation-related molecules that showed distinct temporal activation patterns between WT and Y992F cells. Therefore, we examined the quantitative differences in the ubiquitin-dependent EGFR degradation pathway between the two cells by using our EGFR model. EGFR monoubiquitination is catalyzed by activated Cbl family proteins, thereby facilitating the lysosomal degradation of EGFR [51]. We have validated the model predictions regarding a decrease in the initial expression level of EGFR and Cbl family proteins—c-Cbl and Cbl-b—in Y992F cells (Figure 6A). This would probably affect the amount of EGFR-bound Cbl. Our model predicted the decrease in EGFR-bound Cbl per EGFR in Y992F cells compared to that in the WT cells; this result was then validated by measuring the level of Cbl-b co-immunoprecipitated with EGFR (Figure 7A). Note that c-Cbl was not sufficiently detected in the same sample. These results indicate that Cbl-b has dominant functions for EGFR signaling in our cell lines, which is also supported by the evidence that the number of mass detectable peptides derived from c-Cbl was fewer than that of Cbl-b (Table S1). Because Cbl-b is a ubiquitin ligase of EGFR, this decrease was considered to induce a decrease in the amount of ubiquitinated EGFR in the Y992F cells. Contrary to our intuitive prediction, however, increase in the amount of ubiquitinated EGFR was observed in the Y992F model (Figure 7B), which resulted in rapid EGFR degradation (Figure 7C). These counterintuitive predictions were all successfully validated in the corresponding experiments (Figure 7B, C). These results indicate that the increase in EGFR ubiquitination rate is responsible for the alterations in EGFR degradation pathway in Y992F cells and further suggest that the amount of Y992F ubiquitination does not correlate with the amount of EGFR-bound Cbl-b. There are some direct or indirect mechanistic explanations that should be considered regarding the relationships between EGFR mutation and the increase in EGFR ubiquitination rate in the Y992F model. The former suggest that EGFR mutation changes the receptivity of ubiquitination or that un-modeled factors such as other components in the ubiquitination system or deubiquitinating enzymes are regulated by the Y992 residue of EGFR. The latter could be supported by the Signaling Flux Redistribution (SFR) concept [52], which indicates that if one pathway is enhanced or impaired at the pathway branches, an alternative pathway is down-regulated or enhanced because the total signaling flux in a pathway junction is conserved. One of the possible mechanisms along with the SFR concept is that if there exist any novel positive regulators of EGFR ubiquitination that bind to EGFR, impairment of other proteins binding to EGFR can redistribute the positive signaling flux to the EGFR ubiquitination pathway. Another explanation is also associated with the model specification of the EGFR ubiquitination process. In our model, Cbl catalyzes ubiquitination of all non-ubiquitinated EGFR in the phosphorylated state, irrespective of whether signaling proteins bind to EGFR or not. Thus, diminishing protein binding to EGFR promoted the dephosphorylation of EGFR because the level of free EGFR, with which phosphatases can interact, was increased, resulting in the decrease of Cbl-substrate EGFR. However, if the binding of proteins to EGFR inhibits the catalyzation of EGFR ubiquitination and only free EGFR acts as the substrate of Cbl, decreased protein binding to the mutated EGFR would promote efficient EGFR ubiquitination due to the increase in the amount of free EGFR.

### Enhancement of Phosphorylation Rate Is Essential for Reproducing Y992F Dynamics

**Figure 8:**
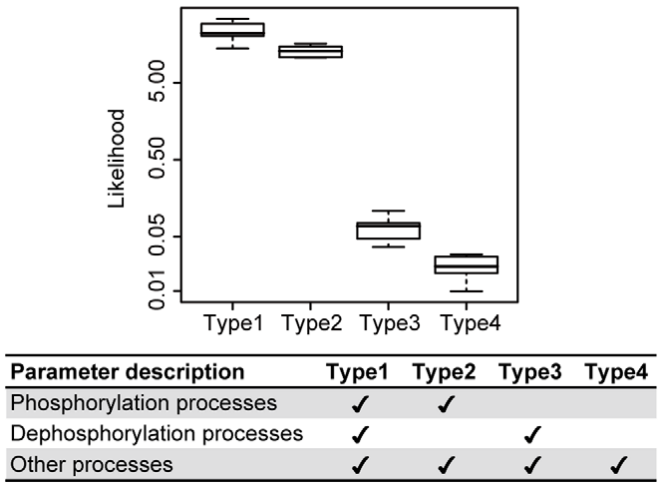
The enhancement of the phosphorylation rate is essential to reproduce the dynamics of Y992F signaling networks. Parameter estimation for the Y992F model was performed using different combinations of parameters indicated in the lower box (Types 1–4). Type 1 contains all 54 parameters, Type 2 contains 40 parameters without dephosphorylation processes, Type 3 contains 49 parameters without phosphorylation processes, and Type 4 contains 35 parameters without both phosphorylation and dephosphorylation processes. For each parameter combination, 10 independent parameter estimation processes were performed with 60 steps per process through data assimilation. The y-axis indicates the likelihood distribution of the best model within each process, while the x-axis indicates the types for parameter combinations.

Our computational model-based analyses indicated that the most obvious characteristic feature of Y992 signaling is the increase in the efficiency of the phosphorylation processes across the network (Figure 5). Since phosphorylation efficiency is determined by the balance between the rates of phosphorylation and dephosphorylation, there is a possibility that the decrease in dephosphorylation rate can also reproduce the dynamics of Y992F signaling. To clarify the aberrant processes in the Y992F cell, we re-estimated the Y992F model from the WT model by changing the parameters including both or either of the two processes. In all, four different combinations of parameters (Type 1–4) were estimated by data assimilation to reproduce the temporal activation data on Y992F, where the remaining parameters were fixed at the values in the WT model. Type 1 indicates an original combination of 54 parameters, which is the hypothesized model in which both phosphorylation and dephosphorylation are altered in the Y992F cell. Types 2 and 3 did not include the parameters for dephosphorylation and phosphorylation processes, respectively. These models were used to test the hypothesis that either of process is not affected by Y992F mutation. For Type 4 as a negative control, the parameters for both the processes were unchanged. Figure 8 shows the likelihood distribution of the best models from the 10 independent parameter estimation experiments using each type of parameter sets (the likelihood and the value of each parameter set can be found in Table S5). Type 2 retained the same degree of likelihood as that of Type 1, whereas Type 3 showed a significant decrease in the likelihood to the same extent as that of Type 4. Notably, Types 3 and 4 could not reproduce the enhancement of the activation level of Shp2 and the sustained activation of ERK (Figure S5). These results indicate that the increase in phosphorylation rates is essential for Y992F signaling in our model.

Although the direct interpretation for the increase in phosphorylation rates is that Y992F mutation increases the intrinsic tyrosine kinase activity of EGFR, it is however reported to remain unchanged by the mutation of Y992 [48]. This evidence strongly suggests the contribution of some extrinsic factors that need to be modeled, such as unknown kinase inhibitors like Mig6 [53], [54], or the alteration of the substrate specificity of EGFR caused by the mutation [55]. An alternative possibility could be the involvement of SFKs that are highly associated with EGF-induced tyrosine phosphoproteome [45]. Because we assumed that the phosphorylation of each molecule is defined by a single upstream kinase in our model (Figure 3 and S2), estimation of the contribution of multiple kinases to respective phosphorylation processes was difficult. There was a large discrepancy regarding the abundance of SFKs between the in silico prediction and the in vivo measurement, indicating that the model for SFKs in EGF signaling is incomplete. Therefore, more sophisticated models containing not only inhibitory molecules but also the complex involvement of multiple kinases are required for further clarification of the regulatory mechanisms underlying the increase in the phosphorylation rate on a network-wide scale.

### Conclusion

Our study reported a phosphoproteomics-based framework for providing a mechanistic view of aberrant signaling initiated by a mutated receptor. The low-biased quantitative data on EGF-induced tyrosine-phosphoproteomerevealed a network-wide enhancement in phosphotyrosine signaling, alteration in EGFR degradation pathway, and aberrant temporal activation of ERK1 in the Y992F cells. Furthermore, our EGFR signaling model based on the HFPNe architecture enabled reduction of the factors responsible for mutational effect to several alterations in the reaction parameters with consideration for different cellular contexts. Model-based analyses indicated that Y992F mutation caused rapid EGFR degradation through the up-regulation of EGFR ubiquitination and aberrant temporal activation of ERK1 by network-wide activation of tyrosine-phosphorylation; this suggests that pY992 strengthens and attenuates phosphotyrosine singling by distinct regulatory mechanisms. By applying our approach to disease-associated genetic alterations of signaling molecules, it will be possible to mechanistically describe the disorders of their cell signaling networks at the system level. Mass spectrometry-based quantitative phosphoproteomics, combined with computational network modeling, will enable the theoretical representation of potential therapeutic strategies for adjusting aberrant network behaviors.

## Materials and Methods

### SILAC Experiment

WT and Y992F cells expressing full-length human EGFR (WT) and mutant EGFR with substitution of tyrosine to phenylalanine at position 992 (Y992F) (the numbering system excludes the 24 amino acid signal peptide of EGFR), respectively, were maintained and used according to our previous study [56]. SILAC experiments were carried out as described previously [45], [57]. Briefly, WT and Y992F cells were labeled with l-arginine (Arg-0), l-arginine-U-13C6 (Arg-6), or l-arginine-U-13C6-15N4 (Arg-10). After overnight starvation, each cell population (5×10-cm dishes per condition) was stimulated with 150 ng/ml of EGF for the indicated time intervals. The cells were washed three times with cold phosphate buffered saline (PBS) and then lysed in TNE buffer containing 50 mM Tris-HCl (pH 7.5), 150 mM NaCl, 1% NP40, 0.1% sodium deoxycholate, 1 mM Na_3_VO_4_, and protease inhibitor cocktail (Roche Diagnostics). The protein concentration of cell extracts was quantified using the bicinchoninic acid (BCA) assay (Pierce Chemical Co.), according to the manufacturer's instructions. The cell lysates were mixed in ratios of 1∶1∶1 and 2∶1∶1 (Arg-0∶Arg-6∶Arg-10) in experiments 1–5 and in experiments 5–8, respectively, and then subjected to immunoprecipitation process. Tyrosine phosphoproteins were captured using anti-phosphotyrosine antibodies (4G10; Upstate and pTyr100; Cell Signaling Technology) and eluted with 25 mM of phenyl phosphate. The eluted proteins were digested by trypsin (Roche Diagnostics) overnight at 37°C, followed by purification with Zip-Tip C18 (Millipore).

### Mass Spectrometry Measurement

The purified peptide mixtures were analyzed using a high-resolution nanoflow reversed-phase liquid chromatography coupled with quadrupole time-of-flight tandem mass spectrometer (Q-Tof-2; Micromass Ltd.), as described previously [45]. The MS/MS signals were then converted to text files by MassLynx (version 3.5, Micromass) under the default parameter settings. The peak lists were searched against the RefSeq mouse (45,347 sequences; July 3, 2006) and human (33,506 sequences; June 25, 2007) sequences using Mascot software (version 2.2; Matrix Science) with a mass tolerance of 500 ppm for parent peptide ions and 0.5 Da for fragment ions. The peptides were constrained to be tryptic with a maximum of three missed cleavages. Acetylation of *N*-terminal residues; oxidation of methionine residues, Arg-6, Arg-10; and formation of pyroglutamic acid for peptides containing an *N*-terminal glutamine were considered as variable modifications. Protein identification was based on the criterion of having at least one MS/MS data with Mascot scores that exceeded the thresholds (p<0.05). The annotated MSMS spectra of peptides used for single peptide identification can be found in Material S3. If a subset of peptides was matched to multiple proteins, the protein that was supported by the most peptides over the eight mass spectrometric measurements was selected as the representative. A randomized decoy database generated by Mascot program estimated a false discovery rate at 0.09% for all the identified peptides. Regarding the proteins identified, quantitation was made on the basis of the mass spectra of SILAC-encoded peptides with Mascot scores (≧20) using the AYUMS algorithm [46] and the MSQuant software (version 1.4.0a16) [47]. Pearson's correlation coefficient between the duplicate measurements of Y992F and WT (Exps. 3–5 versus Exps. 6–8 in Figure 1B) using the independent SILAC-encoded cell populations were calculated as 0.90, indicating good biological reproducibility. To combine the duplicate time-series data regarding Y992F cell signaling, the average value of two time-series data normalized to the value at 5 min of Y992F activation were multiplied by the ratio of Y992F to WT activation at 5 min. For the proteins for which the value at 5 min was not measured in Exp. 8 (Figure 1B), either value of 1 min or 20 min was used for normalization. The procedures for protein identification and quantitation were strictly compliant with those reported previously [45].

### Functional Scoring

Differential activation profiles of EGFR downstream pathways were characterized on the basis of the fold change of phosphotyrosine-dependent proteins and the deviation of time-dependent activation patterns between the two cells. The activation score A of each protein is defined as
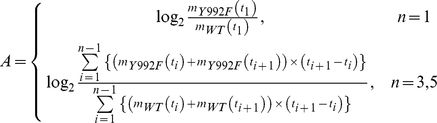
where m_WT_(t_i_) and m_Y992F_(t_i_) denote the relative quantitative values at the time point of t_i_ with regard to WT and Y992F, respectively, which were normalized to the values for WT at 5 min. The activation score *A* represents the ratio of the area under the curve of temporal dynamics data on the Y992F cells to that on the WT cells.

The deviation score *D* of each protein is defined as
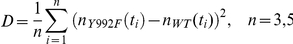
where n_WT_(t_i_) and n_Y992F_(t_i_) denote the relative quantitative values at the time point t_i_ with regard to WT and Y992F, respectively, which were each normalized to the values at 5 min. The calculated scores are indicated in Supplementary information (Table S1).

### Biochemical Simulation Analysis

Cell Illustrator Online (version 4.0; GNI) was used for constructing and performing simulation with 10-ms time-steps on our EGFR signal transduction model. Parameter optimization was performed on the basis of particle filtering to estimate an ensemble of the WT and Y992F models [33] (see Supplementary Text). In order to compare the experimental data with the corresponding simulation results, the SILAC experiment data were normalized by the following procedures. First, the temporal data on each protein were normalized by the value for WT at 5 min. Second, the value at time zero for each protein was subtracted from the values at all the time points. Finally, the value at each time point was renormalized by the value for WT at 5 min. Statistical analyses were performed using the R software (version 2.11.1). We used Welch's t-test for comparison of parameter distributions between the WT and Y992F models. Bonferroni correction was applied to adjust the p-values. Computing time was provided by HA8000 cluster system, Supercomputing Division, Information Technology Center, the University of Tokyo.

### Western Blot Analysis

WT and Y992F cells were cultured in Dulbecco's modified Eagle's medium (DMEM) supplemented with 10% (v/v) fetal bovine serum and antibiotics at 37°C in 5% CO_2_. The cells treated with EGF (150 ng/ml) were lysed in TNE buffer. The total cell lysates were mixed with 5× Laemmli buffer and boiled for 5 min at 100°C. For western blotting of EGFR-bound Cbl-b and ubiquitinated EGFR, the total cell lysates were immunoprecipitated with anti-EGFR (sc-120; Santa Cruz Biotechnology) at 4°C for 1 h. The immunocomplexes were recovered on protein G-agarose (Roche Diagnostics) by incubating overnight at 4°C. After the purified complexes were washed three times with lysis buffer, they were solubilized in Laemmli buffer, followed by boiling for 5 min at 100°C. Protein samples were separated by sodium dodecyl sulfate-polyacrylamide gel electrophoresis (SDS-PAGE) and transferred to polyvinylidene difluoride (PVDF) membranes. After the membranes were blocked in 3% bovine serum albumin/TBS-Tween 20 (TBS-T) at 4°C overnight, they were washed in TBS-T and incubated with the following primary antibodies at room temperature for 1 h: EGFR (sc-03; Santa Cruz Biotechnology), Cbl (sc-170; Santa Cruz Biotechnology), Cbl-b (sc-8006; Santa Cruz Biotechnology), mono ubiquitin (MMS-258; Covance), Shp2 (3752; Cell Signaling Technology), Shc (610081; BD Transduction Laboratories), Grb2 (610111; BD Transduction Laboratories), Src (2109; Cell Signaling Technology), Erk1/2 (9102; Cell Signaling Technology), and β-tubulin (T5293; SIGMA). The membranes were then washed three times with TBS-T and incubated with horseradish peroxidase (HRP)-conjugated secondary antibodies at room temperature for 1 h. The membranes were washed three times with TBS-T, developed using Immobilon Western (Millipore) and detected by Las3000 mini (FujiFilm). Quantification of the band intensities and back ground subtraction were conducted using Multi Gauge software (version 3.0; FujiFilm).

## Supporting Information

Figure S1 Representative mass spectra of SILAC-encoded peptide. Mass spectra of the SILAC-encoded peptide YLVIQGDER from the epidermal growth factor receptor isoform a observed in each experiment are shown. In experiments 1–5, the SILAC-encoded cell lysates were mixed in a ratio of 1∶1∶1 (Arg-0∶Arg-6∶Arg-10), while in experiments 6–8, 5 min of EGF-stimulated cell lysate from Arg-0-encoded WT cells was mixed with Y992F cell lysates in a ratio of 2∶1∶1 (Arg-0∶Arg-6∶Arg-10) to ensure the identification of WT-enriched peptides.
(0.82 MB TIF)

Figure S2 The reaction scheme of the EGFR signal transduction pathway. Single- and double-sided solid-line arrows denote irreversible and reversible state changes, respectively. Dashed-line arrows denote catalytic reactions or indirect association. Double solid-head arrows denote summation into a sigma-state. E, EGF; Er, EGFR; Er2, EGFR dimer; G, Grb2; S, Shc; R, RasGAP; H, Shp2; P, Plcγ1; O, Sos; C, Cbl; A, Gab1; I, PI3K; Hr, Hrs; Sr, Src; P2, PIP2; P3, PIP3; RsD, RasGDP; RsT, RasGTP; Φ, degradation; @EX, at extracellular compartment; @PM, at plasma membrane compartment; @E, at endosomal compartment; @L, at lysosomal compartment. Sigma denotes summation of each state; p denotes tyrosine phosphorylation; ub denotes ubiquitination; pt denotes serine/threonine phosphorylation; * denotes activation; and a dot denotes binding. [ ] denotes additional modification of the molecule. {,} denotes alternative condition of the molecule. The numbers attached to the arrows represent the reaction indexes indicated in Table S2B.
(0.78 MB TIF)

Figure S3 Parameter distributions of the WT and Y992F models. Probability densities of the model parameters were estimated using kernel density estimation based on an ensemble of WT and Y992F models. Mean of each ensemble and p-value obtained from unpaired t-test adjusted by Bonferroni method were indicated.
(1.37 MB PDF)

Figure S4 Western blot analysis of the EGF signaling molecules. A. The temporal dynamics of EGFR-bound Cbl-b and ubiquitinated EGFR. WT and Y992F cells were stimulated with 150 ng/ml of EGF for the time intervals indicated. Extracted proteins were normalized to the initial expression amount of EGFR, and then subjected to EGFR immunoprecipitation. Immunoblotting was performed to detect ubiquitinated EGFR and co-immunoprecipitated Cbl-b. B. Measurement of EGF-induced degradation of EGFR. WT and Y992F cells were stimulated with 150 ng/ml of EGF for the time intervals indicated. Extracted protein samples were dissolved by SDS-PAGE probed using anti-EGFR and anti-β-tubulin antibodies as a loading control.
(0.66 MB TIF)

Figure S5 The simulation results of the best model estimated using the different combinations of parameters (Types 1–4). The solid lines represent the simulation results of the model corresponding to each parameter type indicated in Figure 8. The squares represent the experimental data on the Y992F cells.
(1.06 MB TIF)

Table S1 Results of the SILAC experiments.
(0.16 MB XLS)

Table S2 EGFR model description.
(0.08 MB XLS)

Table S3 Model parameters.
(0.10 MB XLS)

Table S4 Results of the LPI analysis.
(0.03 MB XLS)

Table S5 Parameter estimation experiments using different combinations of parameters.
(0.08 MB XLS)

Material S1 Supplementary Materials and Methods
(0.06 MB DOC)

Material S2 Electronic format files of EGFR model.
(0.15 MB ZIP)

Material S3 Annotated MSMS spectra of peptides used for single peptide identification.
(4.19 MB PDF)

## References

[1] Bublil EM, Yarden Y (2007). The EGF receptor family: spearheading a merger of signaling and therapeutics.. Curr Opin Cell Biol.

[2] Sharma SV, Bell DW, Settleman J, Haber DA (2007). Epidermal growth factor receptor mutations in lung cancer.. Nat Rev Cancer.

[3] Oyama M, Itagaki C, Hata H, Suzuki Y, Izumi T (2004). Analysis of small human proteins reveals the translation of upstream open reading frames of mRNAs.. Genome Res.

[4] Oyama M, Kozuka-Hata H, Suzuki Y, Semba K, Yamamoto T (2007). Diversity of translation start sites may define increased complexity of the human short ORFeome.. Mol Cell Proteomics.

[5] Olsen JV, Blagoev B, Gnad F, Macek B, Kumar C (2006). Global, in vivo, and site-specific phosphorylation dynamics in signaling networks.. Cell.

[6] de Godoy LM, Olsen JV, Cox J, Nielsen ML, Hubner NC (2008). Comprehensive mass-spectrometry-based proteome quantification of haploid versus diploid yeast.. Nature.

[7] Dengjel J, Kratchmarova I, Blagoev B (2009). Receptor tyrosine kinase signaling: a view from quantitative proteomics.. Mol Biosyst.

[8] Zhang Y, Wolf-Yadlin A, Ross PL, Pappin DJ, Rush J (2005). Time-resolved mass spectrometry of tyrosine phosphorylation sites in the epidermal growth factor receptor signaling network reveals dynamic modules.. Mol Cell Proteomics.

[9] Bose R, Molina H, Patterson AS, Bitok JK, Periaswamy B (2006). Phosphoproteomic analysis of Her2/neu signaling and inhibition.. Proc Natl Acad Sci U S A.

[10] Wolf-Yadlin A, Kumar N, Zhang Y, Hautaniemi S, Zaman M (2006). Effects of HER2 overexpression on cell signaling networks governing proliferation and migration.. Mol Syst Biol.

[11] Guha U, Chaerkady R, Marimuthu A, Patterson AS, Kashyap MK (2008). Comparisons of tyrosine phosphorylated proteins in cells expressing lung cancer-specific alleles of EGFR and KRAS.. Proc Natl Acad Sci U S A.

[12] Joughin BA, Naegle KM, Huang PH, Yaffe MB, Lauffenburger DA (2009). An integrated comparative phosphoproteomic and bioinformatic approach reveals a novel class of MPM-2 motifs upregulated in EGFRvIII-expressing glioblastoma cells.. Mol Biosyst.

[13] Tong J, Taylor P, Peterman SM, Prakash A, Moran MF (2009). Epidermal growth factor receptor phosphorylation sites Ser991 and Tyr998 are implicated in the regulation of receptor endocytosis and phosphorylations at Ser1039 and Thr1041.. Mol Cell Proteomics.

[14] Kholodenko BN, Demin OV, Moehren G, Hoek JB (1999). Quantification of short term signaling by the epidermal growth factor receptor.. J Biol Chem.

[15] Brightman FA, Fell DA (2000). Differential feedback regulation of the MAPK cascade underlies the quantitative differences in EGF and NGF signalling in PC12 cells.. FEBS Lett.

[16] Schoeberl B, Eichler-Jonsson C, Gilles ED, Müller G (2002). Computational modeling of the dynamics of the MAP kinase cascade activated by surface and internalized EGF receptors.. Nat Biotechnol.

[17] Hendriks BS, Opresko LK, Wiley HS, Lauffenburger D (2003). Quantitative analysis of HER2-mediated effects on HER2 and epidermal growth factor receptor endocytosis: distribution of homo- and heterodimers depends on relative HER2 levels.. J Biol Chem.

[18] Hatakeyama M, Kimura S, Naka T, Kawasaki T, Yumoto N (2003). A computational model on the modulation of mitogen-activated protein kinase (MAPK) and Akt pathways in heregulin-induced ErbB signalling.. Biochem J.

[19] Resat H, Ewald JA, Dixon DA, Wiley HS (2003). An integrated model of epidermal growth factor receptor trafficking and signal transduction.. Biophys J.

[20] Bhalla US (2004). Signaling in small subcellular volumes. II. Stochastic and diffusion effects on synaptic network properties.. Biophys J.

[21] Sasagawa S, Ozaki Y, Fujita K, Kuroda S (2005). Prediction and validation of the distinct dynamics of transient and sustained ERK activation.. Nat Cell Biol.

[22] Fujioka A, Terai K, Itoh RE, Aoki K, Nakamura T (2006). Dynamics of the Ras/ERK MAPK cascade as monitored by fluorescent probes.. J Biol Chem.

[23] Shankaran H, Wiley HS, Resat H (2006). Modeling the effects of HER/ErbB1-3 coexpression on receptor dimerization and biological response.. Biophys J.

[24] Blinov ML, Faeder JR, Goldstein B, Hlavacek WS (2006). A network model of early events in epidermal growth factor receptor signaling that accounts for combinatorial complexity.. Biosystems.

[25] Tasaki S, Nagasaki M, Oyama M, Hata H, Ueno K (2006). Modeling and estimation of dynamic EGFR pathway by data assimilation approach using time series proteomic data.. Genome Inform.

[26] Birtwistle MR, Hatakeyama M, Yumoto N, Ogunnaike BA, Hoek JB (2007). Ligand-dependent responses of the ErbB signaling network: experimental and modeling analyses.. Mol Syst Biol.

[27] Chen WW, Schoeberl B, Jasper PJ, Niepel M, Nielsen UB (2009). Input-output behavior of ErbB signaling pathways as revealed by a mass action model trained against dynamic data.. Mol Syst Biol.

[28] Matsuno H, Doi A, Nagasaki M, Miyano S (2000). Hybrid Petri net representation of gene regulatory network.. Pac Symp Biocomput.

[29] Nagasaki M, Doi A, Matsuno H, Miyano S (2004). A versatile petri net based architecture for modeling and simulation of complex biological processes.. Genome Inform.

[30] Doi A, Fujita S, Matsuno H, Nagasaki M, Miyano S (2004). Constructing biological pathway models with hybrid functional Petri nets.. In Silico Biol.

[31] Troncale S, Tahi F, Campard D, Vannier J, Guespin J (2006). Modeling and simulation with Hybrid Functional Petri Nets of the role of interleukin-6 in human early haematopoiesis.. Pac Symp Biocomput.

[32] Koh G, Teong HF, Clément MV, Hsu D, Thiagarajan PS (2006). A decompositional approach to parameter estimation in pathway modeling: a case study of the Akt and MAPK pathways and their crosstalk.. Bioinformatics.

[33] Nagasaki M, Yamaguchi R, Yoshida R, Imoto S, Doi A (2006). Genomic data assimilation for estimating hybrid functional Petri net from time-course gene expression data.. Genome Inform.

[34] Yoshida R, Nagasaki M, Yamaguchi R, Imoto S, Miyano S (2008). Bayesian learning of biological pathways on genomic data assimilation.. Bioinformatics.

[35] Nakamura K, Yoshida R, Nagasaki M, Miyano S, Higuchi T (2009). Parameter estimation of in silico biological pathways with particle filtering towards a petascale computing.. Pac Symp Biocomput.

[36] Chen D, Zebiak SE, Busalacchi AJ, Cane MA (1995). An Improved Procedure for EI Nino Forecasting: Implications for Predictability.. Science.

[37] Schulze WX, Deng L, Mann M (2005). Phosphotyrosine interactome of the ErbB-receptor kinase family.. Mol Syst Biol.

[38] Jones RB, Gordus A, Krall JA, MacBeath G (2006). A quantitative protein interaction network for the ErbB receptors using protein microarrays.. Nature.

[39] Kaushansky A, Gordus A, Chang B, Rush J, MacBeath G (2008). A quantitative study of the recruitment potential of all intracellular tyrosine residues on EGFR, FGFR1 and IGF1R.. Mol Biosyst.

[40] Rotin D, Margolis B, Mohammadi M, Daly RJ, Daum G (1992). SH2 domains prevent tyrosine dephosphorylation of the EGF receptor: identification of Tyr992 as the high-affinity binding site for SH2 domains of phospholipase C gamma.. EMBO J.

[41] Tamás P, Solti Z, Bauer P, Illés A, Sipeki S (2003). Mechanism of epidermal growth factor regulation of Vav2, a guanine nucleotide exchange factor for Rac.. J Biol Chem.

[42] Soler C, Beguinot L, Sorkin A, Carpenter G (1993). Tyrosine phosphorylation of ras GTPase-activating protein does not require association with the epidermal growth factor receptor.. J Biol Chem.

[43] Milarski KL, Zhu G, Pearl CG, McNamara DJ, Dobrusin EM (1993). Sequence specificity in recognition of the epidermal growth factor receptor by protein tyrosine phosphatase 1B.. J Biol Chem.

[44] Agazie YM, Hayman MJ (2003). Molecular mechanism for a role of SHP2 in epidermal growth factor receptor signaling.. Mol Cell Biol.

[45] Oyama M, Kozuka-Hata H, Tasaki S, Semba K, Hattori S (2009). Temporal perturbation of tyrosine phosphoproteome dynamics reveals the system-wide regulatory networks.. Mol Cell Proteomics.

[46] Saito A, Nagasaki M, Oyama M, Kozuka-Hata H, Semba K (2007). AYUMS: an algorithm for completely automatic quantitation based on LC-MS/MS proteome data and its application to the analysis of signal transduction.. BMC Bioinformatics.

[47] Schulze WX, Mann M (2004). A novel proteomic screen for peptide-protein interactions.. J Biol Chem.

[48] Holbrook MR, O'Donnell JB, Slakey LL, Gross DJ (1999). Epidermal growth factor receptor internalization rate is regulated by negative charges near the SH2 binding site Tyr992.. Biochemistry.

[49] Emlet DR, Moscatello DK, Ludlow LB, Wong AJ (1997). Subsets of epidermal growth factor receptors during activation and endocytosis.. J Biol Chem.

[50] Kitano H (2004). Biological robustness.. Nat Rev Genet.

[51] Thien CB, Langdon WY (2001). Cbl: many adaptations to regulate protein tyrosine kinases.. Nat Rev Mol Cell Biol.

[52] Selvarajoo K, Takada Y, Gohda J, Helmy M, Akira S (2008). Signaling flux redistribution at toll-like receptor pathway junctions.. PLoS One.

[53] Zhang X, Pickin KA, Bose R, Jura N, Cole PA (2007). Inhibition of the EGF receptor by binding of MIG6 to an activating kinase domain interface.. Nature.

[54] Anastasi S, Baietti MF, Frosi Y, Alemà S, Segatto O (2007). The evolutionarily conserved EBR module of RALT/MIG6 mediates suppression of the EGFR catalytic activity.. Oncogene.

[55] Beebe JA, Wiepz GJ, Guadarrama AG, Bertics PJ, Burke TJ (2003). A carboxyl-terminal mutation of the epidermal growth factor receptor alters tyrosine kinase activity and substrate specificity as measured by a fluorescence polarization assay.. J Biol Chem.

[56] Gotoh N, Tojo A, Muroya K, Hashimoto Y, Hattori S (1994). Epidermal growth factor-receptor mutant lacking the autophosphorylation sites induces phosphorylation of Shc protein and Shc-Grb2/ASH association and retains mitogenic activity.. Proc Natl Acad Sci U S A.

[57] Blagoev B, Ong SE, Kratchmarova I, Mann M (2004). Temporal analysis of phosphotyrosine-dependent signaling networks by quantitative proteomics.. Nat Biotechnol.

