# Supplementary figures and images for: Phosphoproteomics-Based Modeling Defines the Regulatory Mechanism Underlying Aberrant EGFR Signaling
Source: PLoS One. 2010 Nov 10;5(11):e13926. doi: 10.1371/journal.pone.0013926 (PMC2978091; doi:10.1371/journal.pone.0013926)

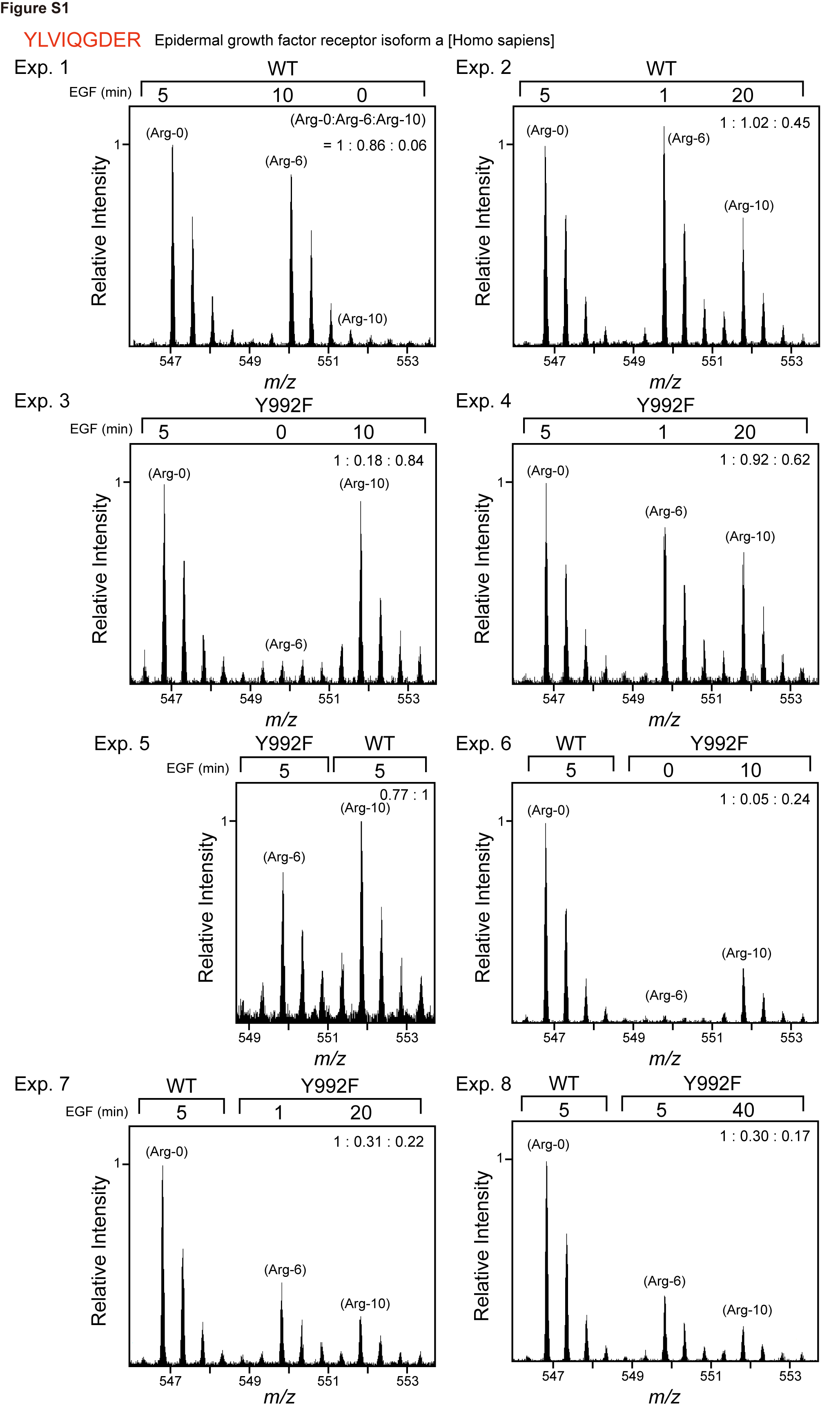

Supplement: Figure S1 — Representative mass spectra of SILAC-encoded peptide. Mass spectra of the SILAC-encoded peptide YLVIQGDER from the epidermal growth factor receptor isoform a observed in each experiment are shown. In experiments 1–5, the SILAC-encoded cell lysates were mixed in a ratio of 1∶1∶1 (Arg-0∶Arg-6∶Arg-10), while in experiments 6–8, 5 min of EGF-stimulated cell lysate from Arg-0-encoded WT cells was mixed with Y992F cell lysates in a ratio of 2∶1∶1 (Arg-0∶Arg-6∶Arg-10) to ensure the identification of WT-enriched peptides. (0.82 MB TIF) [file pone.0013926.s001.tif]

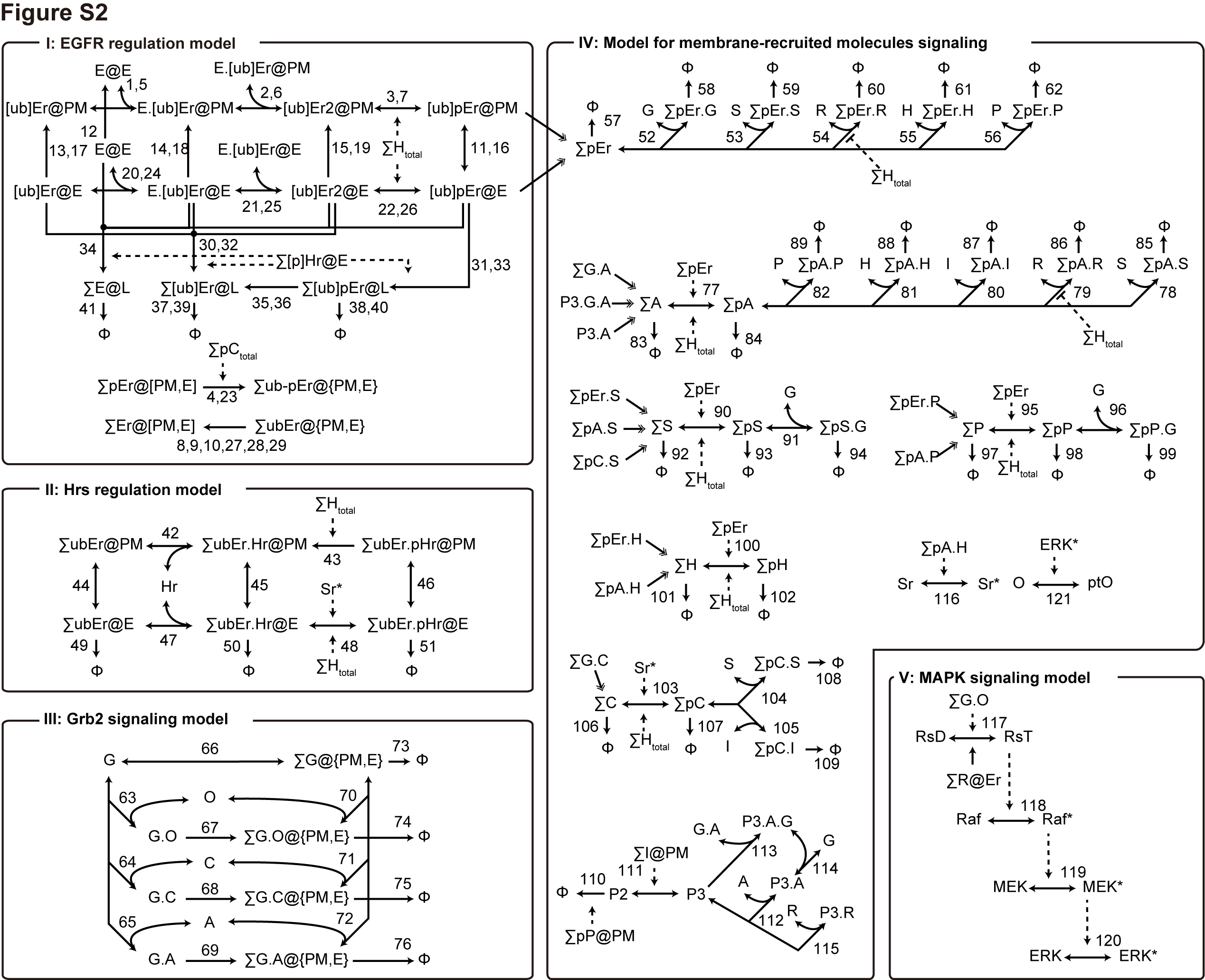

Supplement: Figure S2 — The reaction scheme of the EGFR signal transduction pathway. Single- and double-sided solid-line arrows denote irreversible and reversible state changes, respectively. Dashed-line arrows denote catalytic reactions or indirect association. Double solid-head arrows denote summation into a sigma-state. E, EGF; Er, EGFR; Er2, EGFR dimer; G, Grb2; S, Shc; R, RasGAP; H, Shp2; P, Plcγ1; O, Sos; C, Cbl; A, Gab1; I, PI3K; Hr, Hrs; Sr, Src; P2, PIP2; P3, PIP3; RsD, RasGDP; RsT, RasGTP; Φ, degradation; @EX, at extracellular compartment; @PM, at plasma membrane compartment; @E, at endosomal compartment; @L, at lysosomal compartment. Sigma denotes summation of each state; p denotes tyrosine phosphorylation; ub denotes ubiquitination; pt denotes serine/threonine phosphorylation; * denotes activation; and a dot denotes binding. [ ] denotes additional modification of the molecule. {,} denotes alternative condition of the molecule. The numbers attached to the arrows represent the reaction indexes indicated in Table S2B. (0.78 MB TIF) [file pone.0013926.s002.tif]

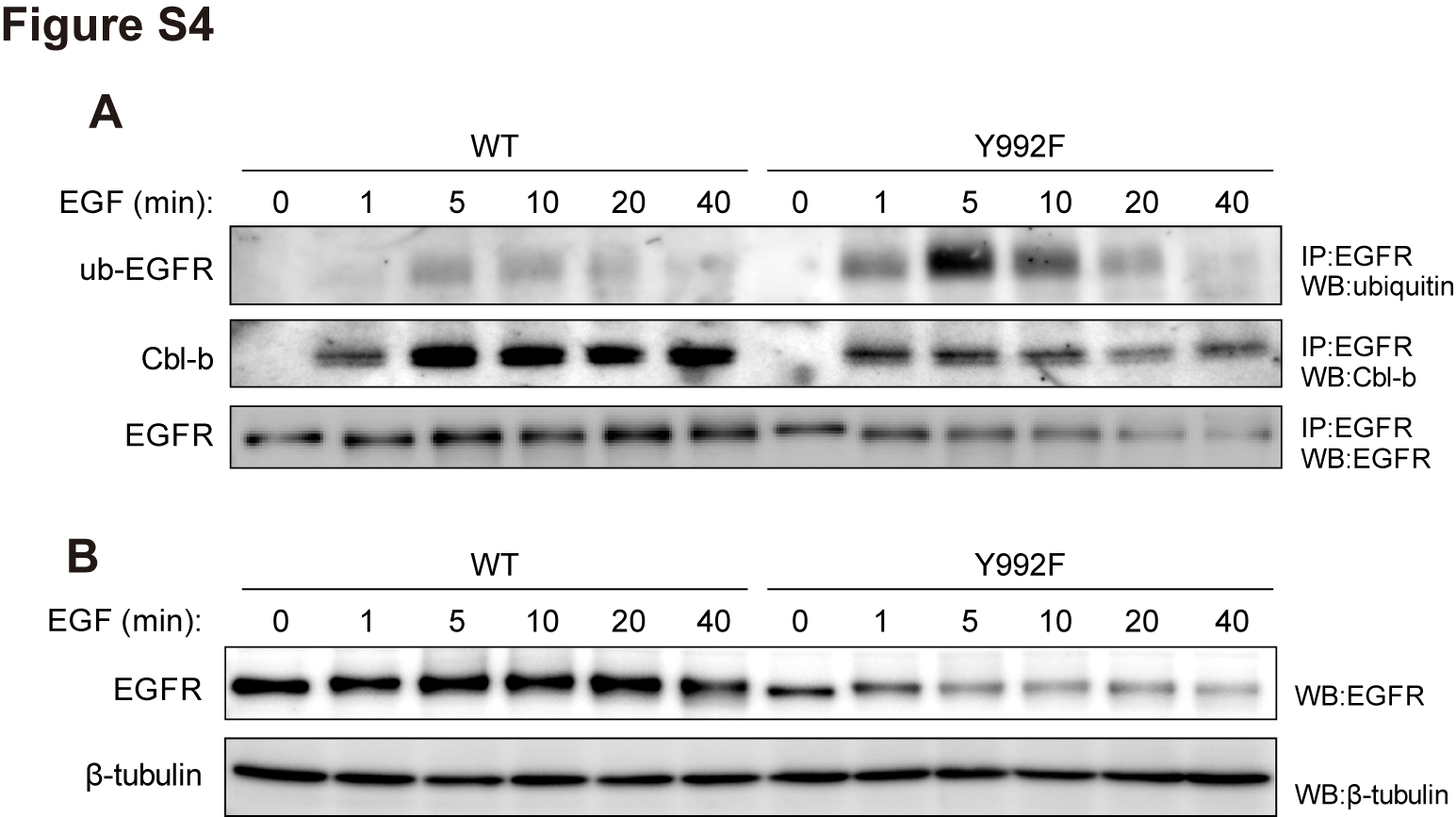

Supplement: Figure S4 — Western blot analysis of the EGF signaling molecules. A. The temporal dynamics of EGFR-bound Cbl-b and ubiquitinated EGFR. WT and Y992F cells were stimulated with 150 ng/ml of EGF for the time intervals indicated. Extracted proteins were normalized to the initial expression amount of EGFR, and then subjected to EGFR immunoprecipitation. Immunoblotting was performed to detect ubiquitinated EGFR and co-immunoprecipitated Cbl-b. B. Measurement of EGF-induced degradation of EGFR. WT and Y992F cells were stimulated with 150 ng/ml of EGF for the time intervals indicated. Extracted protein samples were dissolved by SDS-PAGE probed using anti-EGFR and anti-β-tubulin antibodies as a loading control. (0.66 MB TIF) [file pone.0013926.s004.tif]

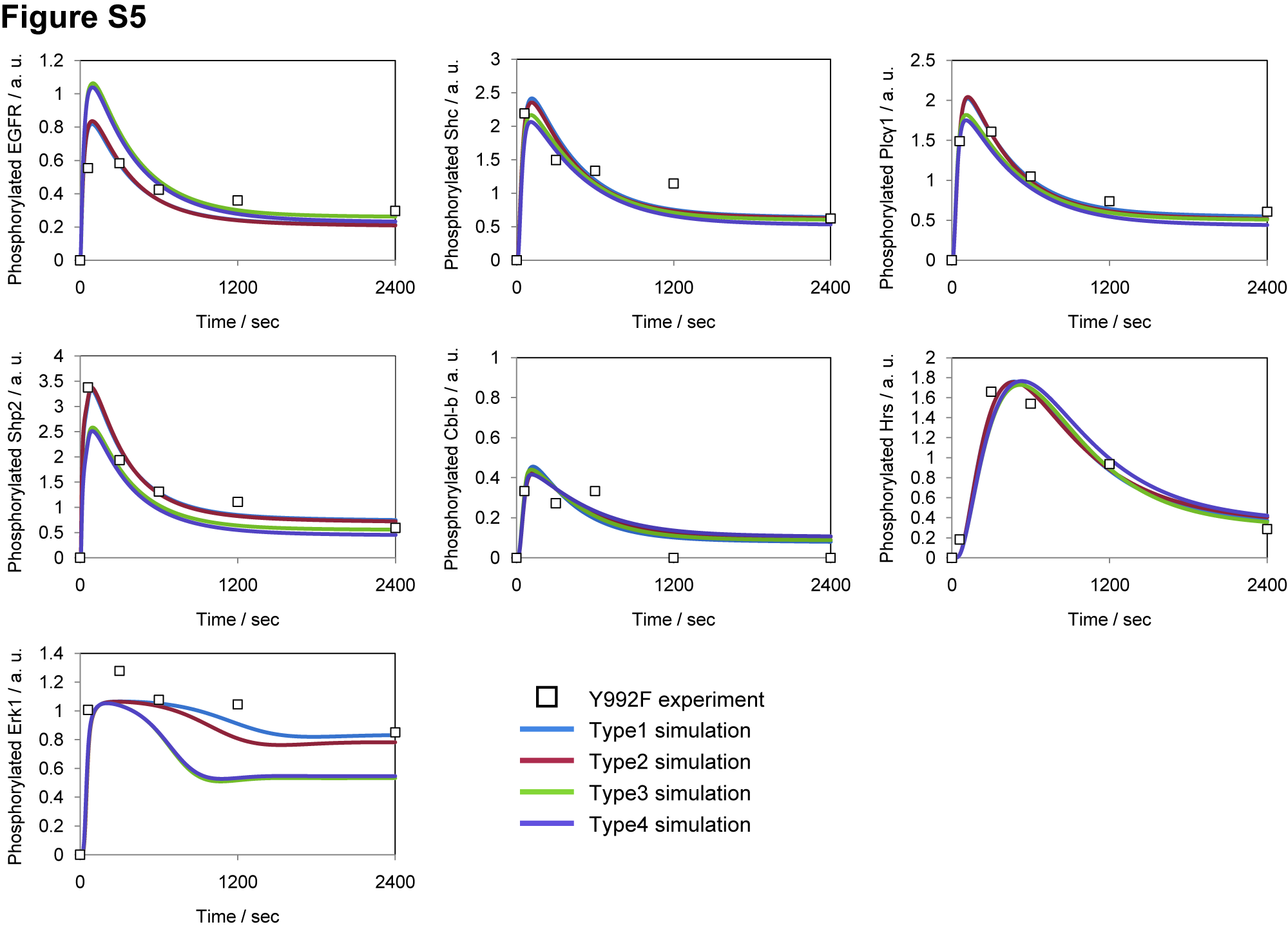

Supplement: Figure S5 — The simulation results of the best model estimated using the different combinations of parameters (Types 1–4). The solid lines represent the simulation results of the model corresponding to each parameter type indicated in Figure 8. The squares represent the experimental data on the Y992F cells. (1.06 MB TIF) [file pone.0013926.s005.tif]
