# Supplementary material for: Phosphoproteomics-Based Modeling Defines the Regulatory Mechanism Underlying Aberrant EGFR Signaling
Source: PLoS One. 2010 Nov 10;5(11):e13926. doi: 10.1371/journal.pone.0013926 (PMC2978091; doi:10.1371/journal.pone.0013926)

Figure S3

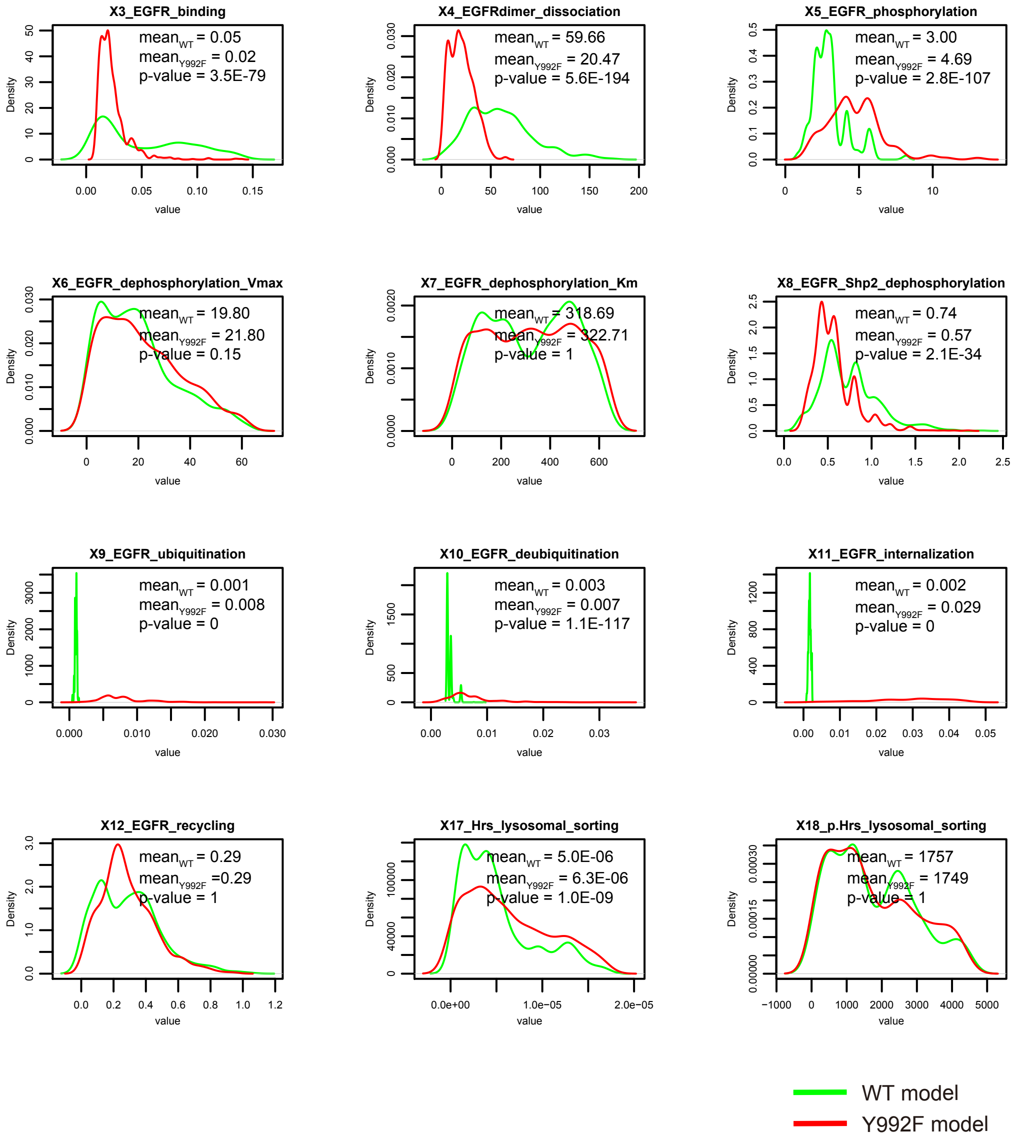
X3_EGFR_binding
mean$_{WT}$ = 0.05
mean$_{Y992F}$ = 0.02
p-value = 3.5E-79
X4_EGFRdimer_dissociation
mean$_{WT}$ = 59.66
mean$_{Y992F}$ = 20.47
p-value = 5.6E-194
X5_EGFR_phosphorylation
mean$_{WT}$ = 3.00
mean$_{Y992F}$ = 4.69
p-value = 2.8E-107
X6_EGFR_dephosphorylation_Vmax
mean$_{WT}$ = 19.80
mean$_{Y992F}$ = 21.80
p-value = 0.15
X7_EGFR_dephosphorylation_Km
mean$_{WT}$ = 318.69
mean$_{Y992F}$ = 322.71
p-value = 1
X8_EGFR_Shp2_dephosphorylation
mean$_{WT}$ = 0.74
mean$_{Y992F}$ = 0.57
p-value = 2.1E-34
X9_EGFR_ubiquitination
mean$_{WT}$ = 0.001
mean$_{Y992F}$ = 0.008
p-value = 0
X10_EGFR_deubiquitination
mean$_{WT}$ = 0.003
mean$_{Y992F}$ = 0.007
p-value = 1.1E-117
X11_EGFR_internalization
mean$_{WT}$ = 0.002
mean$_{Y992F}$ = 0.029
p-value = 0
X12_EGFR_recycling
mean$_{WT}$ = 0.29
mean$_{Y992F}$ =0.29
p-value = 1
X17_Hrs_lysosomal_sorting
mean$_{WT}$ = 5.0E-06
mean$_{Y992F}$ = 6.3E-06
p-value = 1.0E-09
X18_p.Hrs_lysosomal_sorting
mean$_{WT}$ = 1757
mean$_{Y992F}$ = 1749
p-value = 1
Density
value
WT model
Y992F model

Figure S3 continued

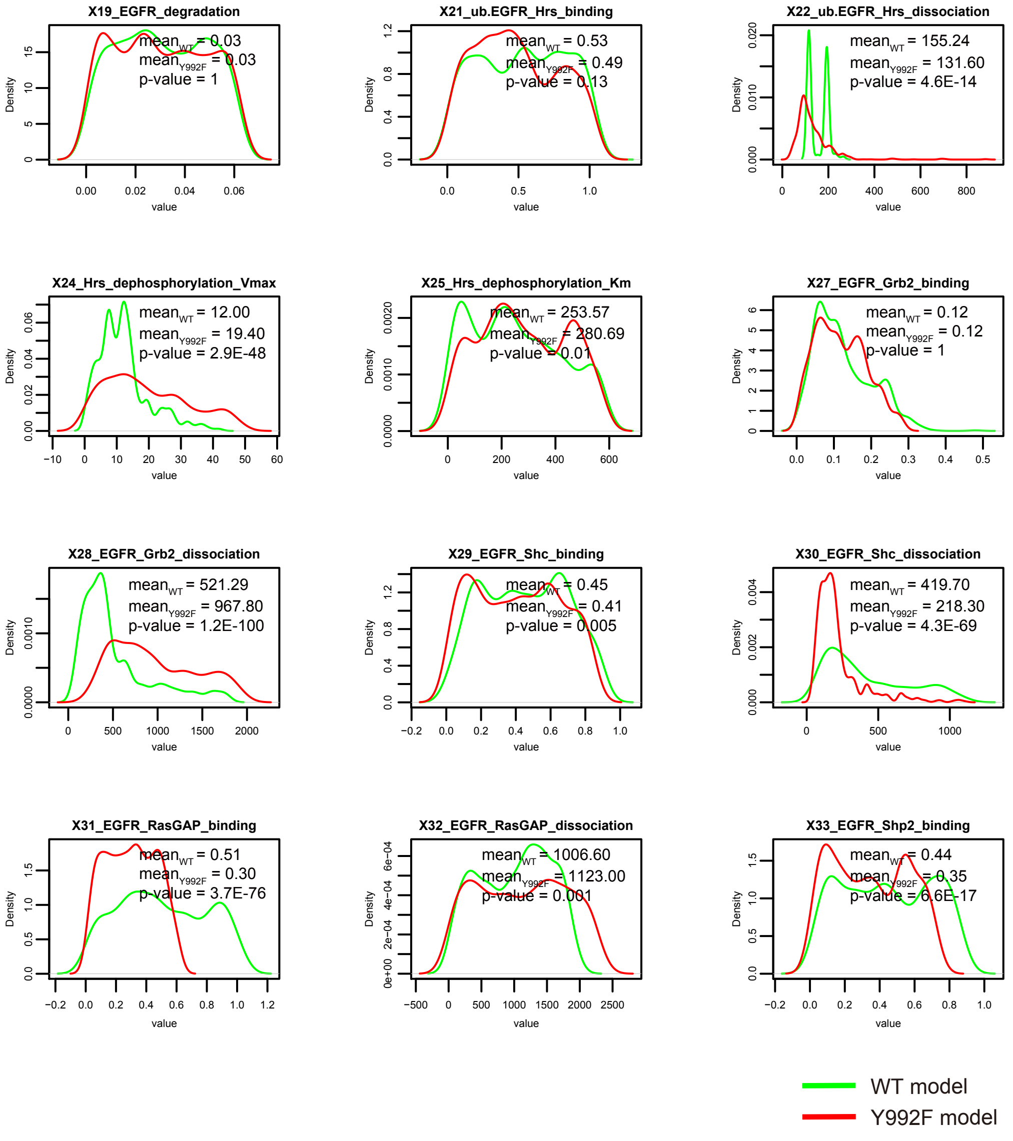

Figure S3 continued

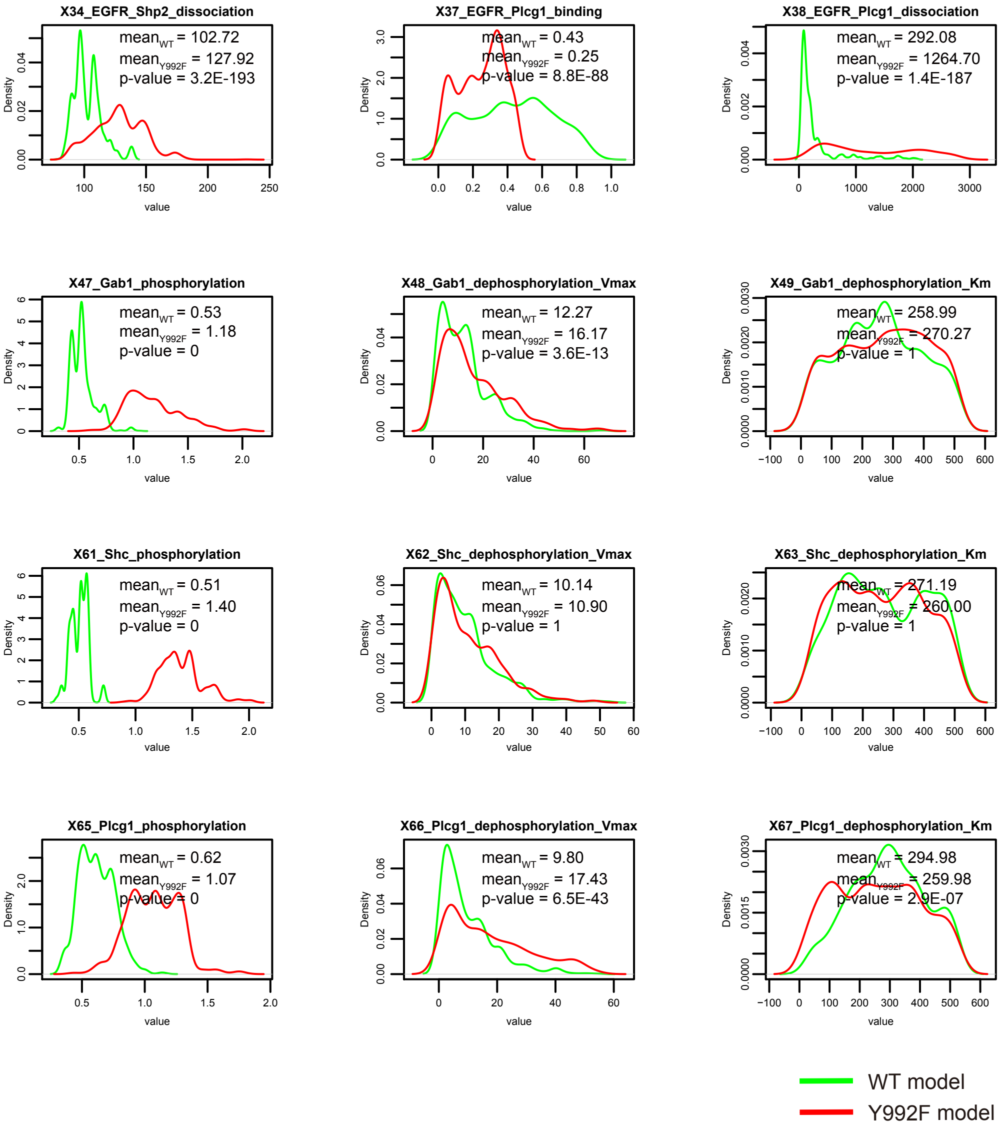

Figure S3 continued

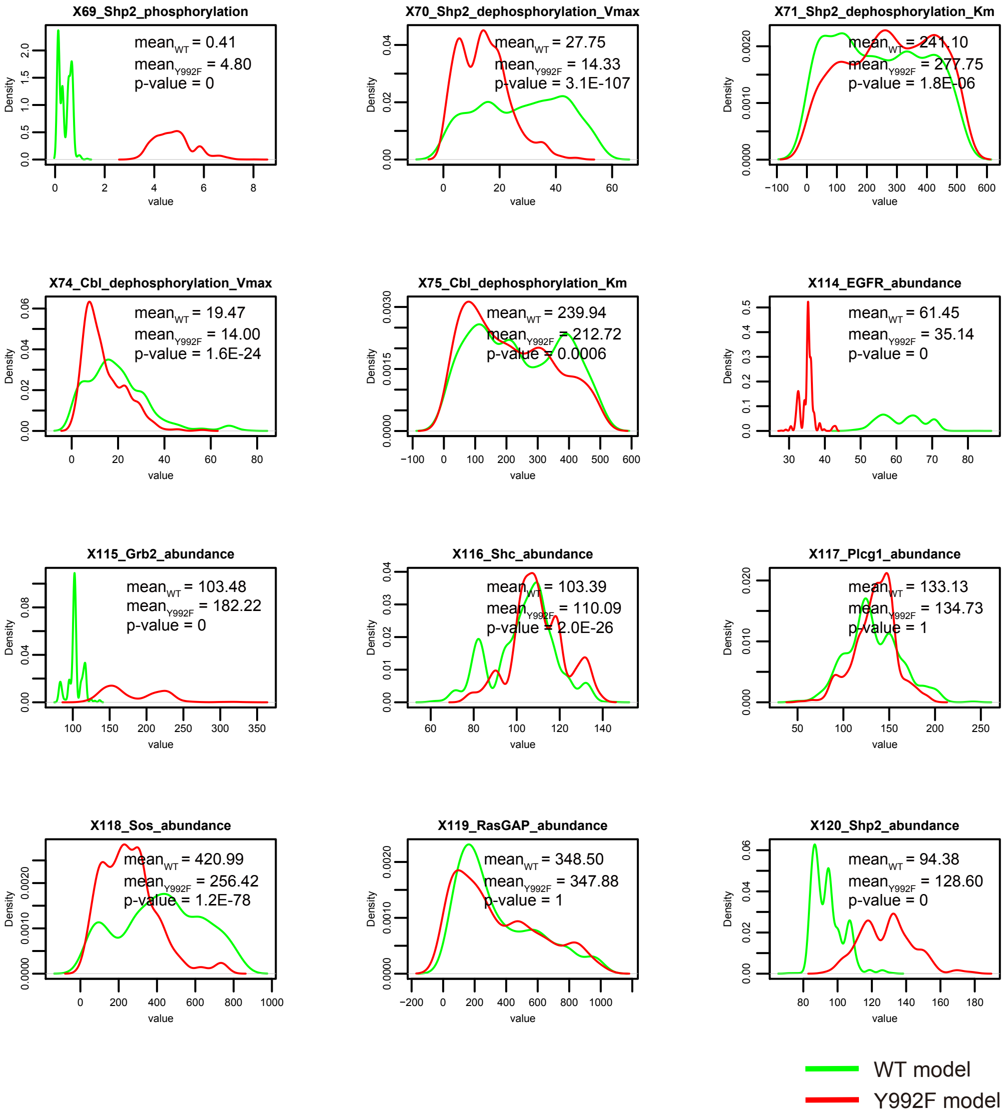

Figure S3 continued

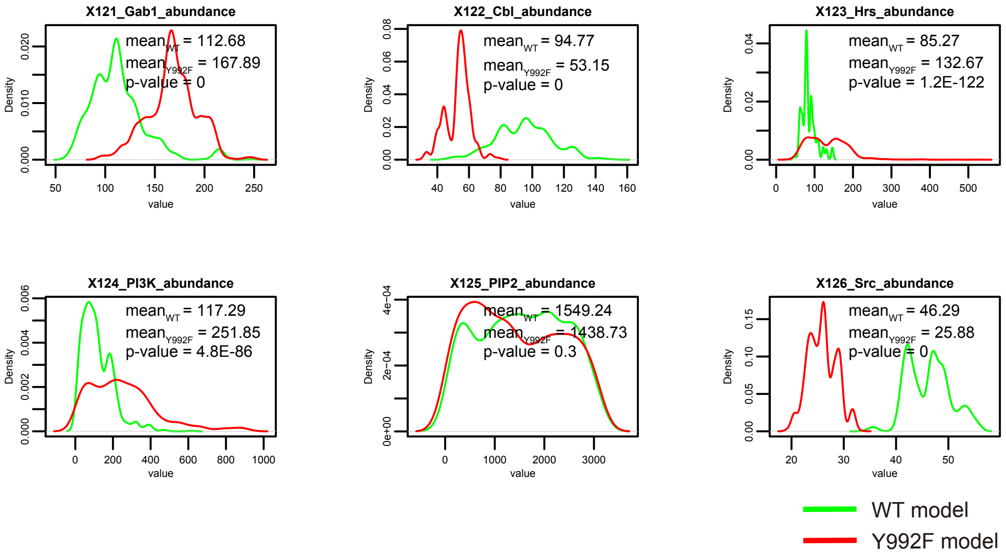

Supplement: Figure S3 — Parameter distributions of the WT and Y992F models. Probability densities of the model parameters were estimated using kernel density estimation based on an ensemble of WT and Y992F models. Mean of each ensemble and p-value obtained from unpaired t-test adjusted by Bonferroni method were indicated. (1.37 MB PDF) [file pone.0013926.s003.pdf]
