# Supplementary material for: Phosphoproteomics-Based Modeling Defines the Regulatory Mechanism Underlying Aberrant EGFR Signaling
Source: PLoS One. 2010 Nov 10;5(11):e13926. doi: 10.1371/journal.pone.0013926 (PMC2978091; doi:10.1371/journal.pone.0013926)
Supplement: Material S1 — Supplementary Materials and Methods (0.06 MB DOC) [file pone.0013926.s011.doc]

**Supplementary Materials and Methods**

***Construction of the EGFR signal transduction model—***The EGFR model, which consists of five pathways (Pathways I-V), is constructed based on the HFPNe architecture by extending and modifying previously reported EGFR model [1, 2] (Figure S2). In pathway I, EGFR activation, autophosphorylation, location change, and degradation were represented. In pathway II, the recruitment of Hrs to EGFR and the subsequent phosphorylation process were modeled. Pathway III is the model for the Grb2 binding process through the SH3 domain in both the cytoplasmic and membrane compartments. Pathway IV is for the recruitment of the adaptor molecules to the membrane and the subsequent reactions at the plasma membrane and endosomal compartment. Pathway V is described for MAPK cascade initiated by Ras activation.

Pathway I represents the detailed state changes of EGFR upon EGF stimulation. Binding of EGF to EGFR leads to receptor dimer formation that results in EGFR kinase activation. Activated EGFR phosphorylates specific tyrosine residues on the COOH-terminal of EGFR and is then rapidly internalized [3]. Internalized EGFR is either shuttled back to the plasma membrane or transported into the late endosomes. The EGFR in the late endosomes is further sorted to lysosomes for degradation or recycled back to the cell surface. Although EGFR trafficking is achieved through multi-step processes, macroscopic receptor dynamics can be modeled in a simpler way without a loss of the important features of the trafficking [4]. Thus, in our model, we do not distinguish between early and late endosomes; rather we treat them as one compartment. In the endosomal compartment, receptors can also bind to EGF and be activated, even with a slight decrease in binding affinity [5].

In pathway II, the recruitment of Hrs to EGFR and the subsequent phosphorylation process are modeled. At the step of receptor targeting to the lysosome from the endosome, ubiquitin-binding endosomal protein machinery is required [6, 7]. Since, it is reported that the depletion of Hrs inhibits lysosomal sorting of EGFR and enhances its recycling back to the plasma membrane, Hrs is considered to be one of the key molecules of such machinery [8, 9]. In the endosome fraction, Hrs binds to ubiquitinated EGFR and is phosphorylated mainly by Src family kinases (SFKs)　[10], up-regulating its lysosomal targeting activity [11]. Moreover, Stern *et al* reported that Hrs dephosphorylation rate is correlated with EGFR degradation rate. Based on the above evidence, we treat Hrs as a representative of endosomal protein machinery. In our model, Hrs can bind to ubiquitinated EGFR independent of the binding status of any other proteins that bind to pEGFR for simplification.

Pathway III is the model for Grb2 binding process through the SH3 domain in both cytoplasmic and membrane compartments. Grb2 is known to constantly associate with Gab1 [12], Cbl [13], and Sos [14] through the SH3 domain without EGF stimulation. Grb2 also binds to pEGFR, phosphorylated Shc (pShc), and phosphorylated Plcγ1 (pPlcγ1) through SH2 domain [15]. In our model, SH3 domain-mediated binding and SH2 domain-mediated binding are not influenced by each other.

Pathway IV is for the recruitment of the adaptor molecules to the membrane and the subsequent reactions at the plasma membrane and the endosomal compartment. Membrane recruitment of the downstream molecules is initiated by pEGFR or phosphatidylinositol-3,4,5-trisphosphate (PIP_3_) in our model. Grb2, Shc, Plcγ1 [16], Shp2 [17], and RasGAP bind to pEGFR, while Gab1 and RasGAP associate with PIP_3_ [18]. In our model, Cbl binds to EGFR indirectly through Grb2 for simplification because the depletion of Grb2 reduces the recruitment of Cbl to EGFR [19]. Membrane-recruited molecules are phosphorylated by either EGFR or SFKs. Since the contribution of each kinase to target protein phosphorylation is poorly understood, EGFR is defined as the main kinase for Gab1, Shp2, Plcγ1, and Shc except Cbl and Hrs according to the evidence that inhibition of SFKs reduces phosphorylation of both the proteins [10, 20]. Phosphorylated molecules serve as adaptors for other molecules such as pGab1 for Shc, Shp2, Plcγ1, and PI3K [21], pShc and pPlcγ1 for Grb2 [15], and phosphorylated Cbl for Shc and PI3K [20]. Phosphorylation of Cbl and Plcγ1 up-regulates the ubiquitin ligase activity and the phospholipase activity, respectively, whereas binding of PI3K to phosphoproteins up-regulates its kinase activity. Because phosphatidylinositol-4,5-bisphosphate (PIP_2_) is a plasma membrane lipid, we calculate the amount of pPlcγ1 and activated PI3K located at the plasma membrane in the model to describe the reactions with PIP_2_. Phosphatase activity of Shp2 is increased by its binding to phosphoproteins or its phosphorylation. Activated Shp2 acts as a positive effector for Src activation [22] and selectively dephosphorylates the RasGAP binding site of EGFR and Gab1 [23]. Thus, we define Shp2 as a representative of EGF-induced phosphatases and also a specific phosphatase for Src activation and RasGAP binding sites. Regarding the behavior of the molecules recruited through EGFR, we assume that the complex bound to EGFR dissociates into each single molecule during the EGFR translocation to the lysosome. In each sigma state, which is the summation of the molecules at the plasma membrane and endosomal compartments, we calculate the ratio of EGFR-driven recruitment to PIP3-driven recruitment and deprived the molecules recruited through EGFR from the sigma state according to the EGFR translocation rate to the lysosomal compartment.

Pathway V is described for MAPK cascade initiated by Ras activation. RasGAP can localize at the membrane through EGFR or PIP_3_. Although RasGAP is mostly recruited to the membrane through PIP_3_, it cannot accelerate the turnover of GTP hydrolysis of Ras [18]. Thus, we describe only the RasGAP recruited through EGFR as active in our model. Each reaction in the Ras-MAPK pathway is modeled as a one-step reaction for simplification according to our previous study [1].

***Parameter estimation—***First we performed manual tuning of the parameters for WT model as follows. The abundance of EGFR was calculated by assuming that the amount of EGFR-bound EGF equals the amount of EGFR [24], where 1 pl is employed for the cell volume. The abundance of other signaling molecules is determined within a reasonable range according to previous models. The initial values for binding rate constants and dissociation constants for protein-protein interaction are unified to 0.1 nM^-1^s^-1^ and 100 nM, respectively. The parameters related to internalization and degradation of EGFR were manually adjusted to prevent excess degradation of total EGFR within an hour after EGF stimulation on the basis of the literature [26]. The parameters for the binding process of PIP_3_ to RasGAP were adjusted based on the fact that Sos accumulates at the plasma membrane faster than RasGAP [27]. The parameters regarding PIP_3_ production and binding of Gab1 to PIP_3_ were tuned to prevent bistable behavior of Gab1 phosphorylation upon low EGF stimulation caused by the feed-forward loop between Gab1 and PIP_3_. Then, we estimated global and local distributions of parameters using a sequential Monte Carlo method known as a particle filter [25] to generate the phosphorylation dynamics of EGFR, Shc, Plcγ1, Hrs, Cbl-b, Shp2, and ERK1 in the WT and Y992F cells. In the global parameter optimization process we performed a stepwise calculation to estimate the distribution of the model parameters. At each calculation step, 100,352 particles were generated according to log-normal distribution with base two (mean; initial value of each parameter, standard deviation; 0.035 × initial value of each parameter) and the values of the parameters were updated according to those newly estimated in a repeated manner. Our preliminary parameter optimization experiments revealed that the likelihood of the WT and Y992F models were stably converged over the 3 and 60 steps of calculations, respectively. Then, we performed three steps of optimization for the WT model and 10 independent parameter estimation experiments with 60 steps for the Y992F model. In the local optimization process, 1,000 particles were generated from the distribution that ranges from a minimum of p^i^_WT_ × 0.1 and p^i^_Y992F_ × 0.1 to a maximum of p^i^_WT_ × 10 and p^i^_Y992F_ × 10 regarding the i th parameter, while other parameters were generated according to the probabilistic distributions obtained by the global optimization process. We adopted 0.3 as the sigma value in likelihood calculation.

**Supplementary References**

1. Tasaki S, Nagasaki M, Oyama M, Hata H, Ueno K, et al. (2006) Modeling and estimation of dynamic EGFR pathway by data assimilation approach using time series proteomic data. Genome Inform 17: 226-238.

2. Birtwistle MR, Hatakeyama M, Yumoto N, Ogunnaike BA, Hoek JB, et al. (2007) Ligand-dependent responses of the ErbB signaling network: experimental and modeling analyses. Mol Syst Biol 3: 144.

3. Wiley HS (2003) Trafficking of the ErbB receptors and its influence on signaling. Exp Cell Res 284: 78-88.

4. Lund KA, Opresko LK, Starbuck C, Walsh BJ, Wiley HS (1990) Quantitative analysis of the endocytic system involved in hormone-induced receptor internalization. J Biol Chem 265: 15713-15723.

5. Ebner R, Derynck R (1991) Epidermal growth factor and transforming growth factor-alpha: differential intracellular routing and processing of ligand-receptor complexes. Cell Regul 2: 599-612.

6. Williams RL, Urbé S (2007) The emerging shape of the ESCRT machinery. Nat Rev Mol Cell Biol 8: 355-368.

7. Komada M, Kitamura N (2005) The Hrs/STAM complex in the downregulation of receptor tyrosine kinases. J Biochem 137: 1-8.

8. Raiborg C, Malerød L, Pedersen NM, Stenmark H (2008) Differential functions of Hrs and ESCRT proteins in endocytic membrane trafficking. Exp Cell Res 314: 801-813.

9. Lu Q, Hope LW, Brasch M, Reinhard C, Cohen SN (2003) TSG101 interaction with HRS mediates endosomal trafficking and receptor down-regulation. Proc. Natl Acad Sci U S A 100: 7626-7631.

10. Bache KG, Raiborg C, Mehlum, A, Madshus IH, Stenmark H (2002) Phosphorylation of Hrs downstream of the epidermal growth factor receptor. Eur J Biochem 269: 3881-3887.

11. Stern KA, Visser Smit GD, Place TL, Winistorfer S, Piper RC, et al. (2007) Epidermal growth factor receptor fate is controlled by Hrs tyrosine phosphorylation sites that regulate Hrs degradation. Mol Cell Biol 27: 888-898.

12. Lock LS, Royal I, Naujokas MA, Park M (2000) Identification of an atypical Grb2 carboxyl-terminal SH3 domain binding site in Gab docking proteins reveals Grb2-dependent and -independent recruitment of Gab1 to receptor tyrosine kinases. J Biol Chem 275: 31536-31545.

13. Ettenberg SA, Keane MM, Nau MM, Frankel M, Wang LM, et al. (1999) cbl-b inhibits epidermal growth factor receptor signaling. Oncogene 18: 1855-1866.

14. Takeuchi Y, Pausawasdi N, Todisco A (1999) Carbachol activates ERK2 in isolated gastric parietal cells via multiple signaling pathways. Am J Physiol 276: 1484-1492.

15. Pei Z, Maloney JA, Yang L, Williamson JR (1997) A new function for phospholipase C-gamma1: coupling to the adaptor protein GRB2. Arch Biochem Biophys 345: 103-110.

16. Rotin D, Margolis B, Mohammadi M, Daly RJ, Daum G, et al. (1992) SH2 domains prevent tyrosine dephosphorylation of the EGF receptor: identification of Tyr992 as the high-affinity binding site for　SH2 domains of phospholipase C gamma. EMBO J 11: 559-567.

17. Agazie YM, Hayman MJ (2003) Molecular mechanism for a role of SHP2 in epidermal growth factor receptor signaling. Mol Cell Biol 23: 7875-7886.

18. Lockyer PJ, Wennström S, Kupzig S, Venkateswarlu K, Downward J, et al. (1999) Identification of the ras GTPase-activating protein GAP1(m) as a phosphatidylinositol-3,4,5-trisphosphate-binding protein in vivo. Curr Biol 9: 265-268.

19. Swaminathan G, Tsygankov AY (2006) The Cbl family proteins: ring leaders in regulation of cell signaling. J Cell Physiol 209: 21-43.

20. Thien CB, Langdon WY (2001) Cbl: many adaptations to regulate protein tyrosine kinases. Nat Rev Mol Cell Biol 2: 294-307.

21. Holgado-Madruga M, Emlet DR, Moscatello DK, Godwin AK, Wong AJ (1996) A Grb2-associated docking protein in EGF- and insulin-receptor signalling. Nature 379: 560-564.

22. Ren Y, Meng S, Mei L, Zhao ZJ, Jove R, et al. (2004) Roles of Gab1 and SHP2 in paxillin tyrosine dephosphorylation and Src activation in response to epidermal growth factor. J Biol Chem 279: 8497-8505.

23. Montagner A, Yart A, Dance M, Perret B, Salles JP, et al. (2005) A novel role for Gab1 and SHP2 in epidermal growth factor-induced Ras activation. J Biol Chem 280: 5350-5360.

24. Gotoh N, Tojo A, Muroya K, Hashimoto Y, Hattori S, et al. (1994) Epidermal growth factor-receptor mutant lacking the autophosphorylation sites induces phosphorylation of Shc protein and Shc-Grb2/ASH association and retains mitogenic activity. Proc Natl Acad Sci U S A 91: 167-171.

25. Nagasaki M, Yamaguchi R, Yoshida R, Imoto S, Doi A, et al. (2006) Genomic data assimilation for estimating hybrid functional Petri net from time-course gene expression data. Genome Inform 17: 46-61.

26. Melikova MS, Kondratov KA, Kornilova ES (2006) Two different stages of epidermal growth factor (EGF) receptor endocytosis are sensitive to free ubiquitin depletion produced by proteasome inhibitor MG132. Cell Biol Int 30: 31-43.

27. Sasagawa S, Ozaki Y, Fujita K, Kuroda S (2005) Prediction and validation of the distinct dynamics of transient and sustained ERK activation. Nat Cell Biol 7: 365-373.

28. Kholodenko BN, Demin OV, Moehren G, Hoek JB (1999) Quantification of short term signaling by the epidermal growth factor receptor. J Biol Chem 274: 30169-30181.

29. Moehren G, Markevich N, Demin O, Kiyatkin A, Goryanin I, et al. (2002) Temperature dependence of the epidermal growth factor receptor signaling network can be accounted for by a kinetic model. Biochemistry 41: 306-320.

30. Resat H, Ewald JA, Dixon DA, Wiley HS (2003) An integrated model of epidermal growth factor receptor trafficking and signal transduction. Biophys J 85: 730-743.

31. Hatakeyama M, Kimura S, Naka T, Kawasaki T, Yumoto N, et al. (2003) A computational model on the modulation of mitogen-activated protein kinase (MAPK) and Akt pathways in heregulin-induced ErbB signalling. Biochem J 373: 451-463.

32. Markevich NI, Moehren G, Demin OV, Kiyatkin A, Hoek JB, et al. (2004) Signal processing at the Ras circuit: what shapes Ras activation patterns? Syst Biol 1: 104-113.

33. Fujioka A, Terai K, Itoh RE, Aoki K, Nakamura T, et al. (2006) Dynamics of the Ras/ERK MAPK cascade as monitored by fluorescent probes. J Biol Chem 281: 8917-8926.
