# Supplementary material for: Phosphoproteomics-Based Modeling Defines the Regulatory Mechanism Underlying Aberrant EGFR Signaling
Source: PLoS One. 2010 Nov 10;5(11):e13926. doi: 10.1371/journal.pone.0013926 (PMC2978091; doi:10.1371/journal.pone.0013926)

Peptide View

MS/MS Fragmentation of ADVSVDQSKLPGVK
Found in gi|71043970|ref|NP_001020786.1|, Ymer protein isoform 2 [Mus musculus]

Match to Query 462: 1483.799848 from(742.907200,2+)

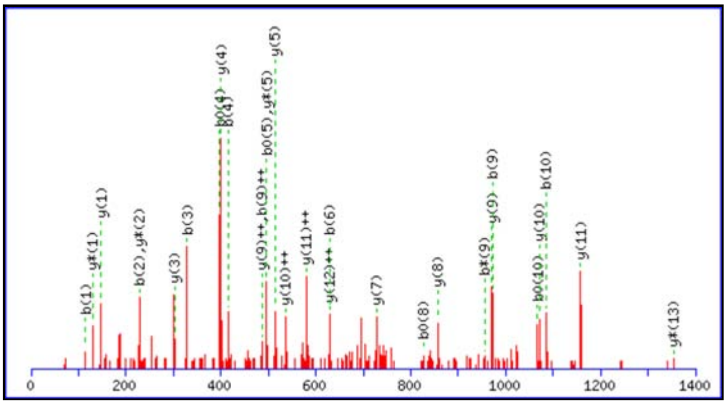

Monoisotopic mass of neutral peptide Mr(calc): 1483.7882
Variable modifications:
N-term : N-Acetyl (Protein)
Ions Score: 55 **Expect:** 0.0049
Matches (Bold Red): 30/130 fragment ions using 50 most intense peaks

#	b	b^{++}	b*	b^{*++}	b^0	b^{0++}	Seq.	y	y^{++}	y*	y^{*++}	y^0	y^{0++}	#
1	**114.0549**	57.5311					A							14
2	**229.0819**	115.0446			211.0713	106.0393	D	1371.7478	686.3775	**1354.7212**	677.8643	1353.7372	677.3723	13
3	**328.1503**	164.5788			310.1397	155.5735	V	1256.7209	**628.8641**	1239.6943	620.3508	1238.7103	619.8588	12
4	**415.1823**	208.0948			**397.1718**	199.0895	S	**1157.6524**	**579.3299**	1140.6259	570.8166	1139.6419	570.3246	11
5	514.2507	257.6290			**496.2402**	248.6237	V	**1070.6204**	**535.8138**	1053.5939	527.3006	1052.6098	526.8086	10
6	**629.2777**	315.1425			611.2671	306.1372	D	**971.5520**	**486.2796**	954.5255	477.7664	953.5414	477.2744	9
7	757.3363	379.1718	740.3097	370.6585	739.3257	370.1665	Q	**856.5251**	428.7662	839.4985	420.2529	838.5145	419.7609	8
8	844.3683	422.6878	827.3417	414.1745	**826.3577**	413.6825	S	**728.4665**	364.7369	711.4399	356.2236	710.4559	355.7316	7
9	**972.4632**	**486.7353**	**955.4367**	478.2220	954.4527	477.7300	K	641.4345	321.2209	624.4079	312.7076			6
10	**1085.5473**	543.2773	1068.5208	534.7640	**1067.5367**	534.2720	L	**513.3395**	257.1734	**496.3129**	248.6601			5
11	1182.6001	591.8037	1165.5735	583.2904	1164.5895	582.7984	P	**400.2554**	200.6314	383.2289	192.1181			4
12	1239.6215	620.3144	1222.5950	611.8011	1221.6110	611.3091	G	**303.2027**	152.1050	286.1761	143.5917			3
13	1338.6899	669.8486	1321.6634	661.3353	1320.6794	660.8433	V	246.1812	123.5942	**229.1547**	115.0810			2
14							K	**147.1128**	74.0600	**130.0863**	65.5468			1

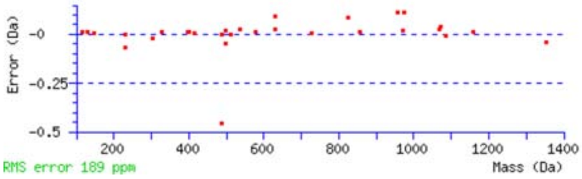

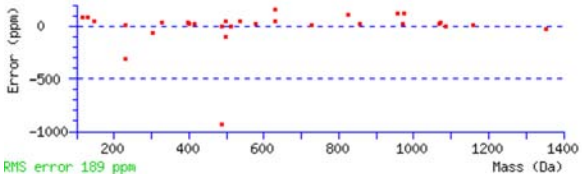

Peptide View

MS/MS Fragmentation of ALMVEWTDEFKNDPQLSLISAMIK
Found in gi|6755668|ref|NP_035614.1|, signal transducing adaptor molecule (SH3 domain and ITAM motif) 1 [Mus musculus]

Match to Query 891: 2778.630072 from(927.217300,3+)

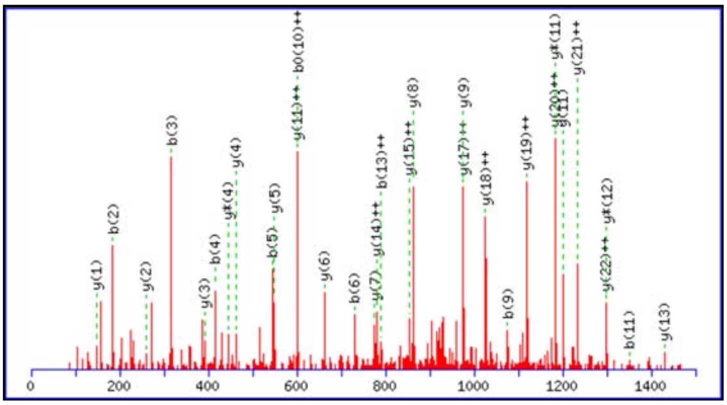

Monoisotopic mass of neutral peptide Mr(calc): 2778.3972
Ions Score: 53 Expect: 0.037
Matches (Bold Red): 32/240 fragment ions using 88 most intense peaks

#	b	b^{++}	b*	b^{*++}	b^{0}	b^{0++}	Seq.	y	y^{++}	y*	y^{*++}	y^{0}	y^{0++}	#
1	72.0444	36.5258					A							24
2	**185.1284**	93.0679					L	2708.3673	1354.6873	2691.3408	1346.1740	2690.3568	1345.6820	23
3	**316.1689**	158.5881					M	2595.2833	**1298.1453**	2578.2567	1289.6320	2577.2727	1289.1400	22
4	**415.2373**	208.1223					V	2464.2428	**1232.6250**	2447.2162	1224.1118	2446.2322	1223.6198	21
5	**544.2799**	272.6436			526.2694	263.6383	E	2365.1744	**1183.0908**	2348.1478	1174.5776	2347.1638	1174.0855	20
6	**730.3592**	365.6833			712.3487	356.6780	W	2236.1318	**1118.5695**	2219.1052	1110.0563	2218.1212	1109.5643	19
7	831.4069	416.2071			813.3964	407.2018	T	2050.0525	**1025.5299**	2033.0259	1017.0166	2032.0419	1016.5246	18
8	946.4339	473.7206			928.4233	464.7153	D	1949.0048	**975.0060**	1931.9783	966.4928	1930.9942	966.0008	17
9	**1075.4764**	538.2419			1057.4659	529.2366	E	1833.9779	917.4926	1816.9513	908.9793	1815.9673	908.4873	16
10	1222.5449	611.7761			1204.5343	**602.7708**	F	1704.9353	**852.9713**	1687.9087	844.4580	1686.9247	843.9660	15
11	**1350.6398**	675.8235	1333.6133	667.3103	1332.6293	666.8183	K	1557.8669	**779.4371**	1540.8403	770.9238	1539.8563	770.4318	14
12	1464.6827	732.8450	1447.6562	724.3317	1446.6722	723.8397	N	**1429.7719**	715.3896	1412.7454	706.8763	1411.7613	706.3843	13
13	1579.7097	**790.3585**	1562.6831	781.8452	1561.6991	781.3532	D	1315.7290	658.3681	**1298.7024**	649.8548	1297.7184	649.3628	12
14	1676.7624	838.8849	1659.7359	830.3716	1658.7519	829.8796	P	**1200.7020**	**600.8547**	**1183.6755**	592.3414	1182.6915	591.8494	11
15	1804.8210	902.9142	1787.7945	894.4009	1786.8105	893.9089	Q	1103.6493	552.3283	1086.6227	543.8150	1085.6387	543.3230	10
16	1917.9051	959.4562	1900.8785	950.9429	1899.8945	950.4509	L	**975.5907**	488.2990	958.5641	479.7857	957.5801	479.2937	9
17	2004.9371	1002.9722	1987.9106	994.4589	1986.9266	993.9669	S	**862.5066**	431.7570	845.4801	423.2437	844.4961	422.7517	8
18	2118.0212	1059.5142	2100.9946	1051.0010	2100.0106	1050.5089	L	**775.4746**	388.2409	758.4481	379.7277	757.4640	379.2357	7
19	2231.1052	1116.0563	2214.0787	1107.5430	2213.0947	1107.0510	I	**662.3905**	331.6989	645.3640	323.1856	644.3800	322.6936	6
20	2318.1373	1159.5723	2301.1107	1151.0590	2300.1267	1150.5670	S	**549.3065**	275.1569	532.2799	266.6436	531.2959	266.1516	5
21	2389.1744	1195.0908	2372.1478	1186.5776	2371.1638	1186.0855	A	**462.2745**	231.6409	**445.2479**	223.1276			4
22	2520.2149	1260.6111	2503.1883	1252.0978	2502.2043	1251.6058	M	**391.2373**	196.1223	374.2108	187.6090			3
23	2633.2989	1317.1531	2616.2724	1308.6398	2615.2884	1308.1478	I	**260.1969**	130.6021	243.1703	122.0888			2
24							K	**147.1128**	74.0600	130.0863	65.5468			1

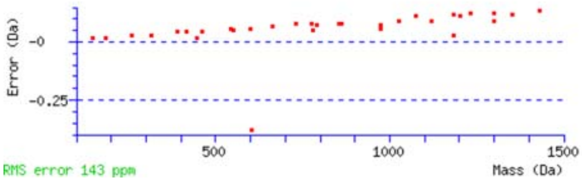

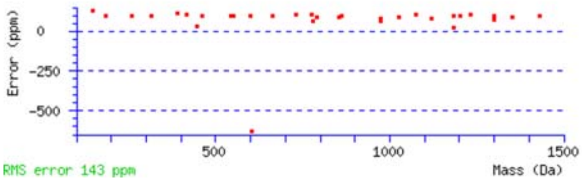

Peptide View

MS/MS Fragmentation of AMSTTSVTSSQPGK
Found in gi|7304993|ref|NP_038838.1|, drebrin-like [Mus musculus]

Match to Query 378: 1380.720648 from(691.367600,2+)

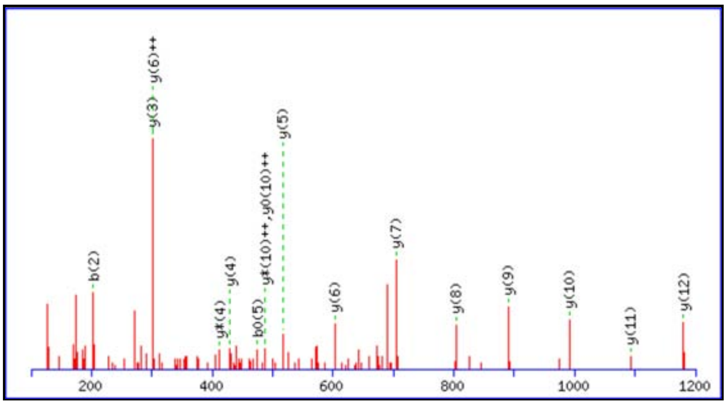

Monoisotopic mass of neutral peptide Mr(calc): 1380.6555
Ions Score: 90 **Expect:** 1.6e-006
Matches (**Bold Red**): 16/124 fragment ions using 18 most intense peaks

#	b	b^{++}	b*	$b*^{++}$	b^0	b^{0++}	Seq.	y	y^{++}	y*	$y*^{++}$	y^0	y^{0++}	#
1	72.0444	36.5258					A							14
2	**203.0849**	102.0461					M	1310.6257	655.8165	1293.5991	647.3032	1292.6151	646.8112	13
3	290.1169	145.5621			272.1063	136.5568	S	**1179.5852**	590.2962	1162.5586	581.7829	1161.5746	581.2909	12
4	391.1646	196.0859			373.1540	187.0806	T	**1092.5531**	546.7802	1075.5266	538.2669	1074.5426	537.7749	11
5	492.2123	246.6098			**474.2017**	237.6045	T	**991.5055**	496.2564	974.4789	**487.7431**	973.4949	**487.2511**	10
6	579.2443	290.1258			561.2337	281.1205	S	**890.4578**	445.7325	873.4312	437.2193	872.4472	436.7272	9
7	678.3127	339.6600			660.3021	330.6547	V	**803.4258**	402.2165	786.3992	393.7032	785.4152	393.2112	8
8	779.3604	390.1838			761.3498	381.1785	T	**704.3573**	352.6823	687.3308	344.1690	686.3468	343.6770	7
9	866.3924	433.6998			848.3818	424.6946	S	**603.3097**	**302.1585**	586.2831	293.6452	585.2991	293.1532	6
10	953.4244	477.2159			935.4139	468.2106	S	**516.2776**	258.6425	499.2511	250.1292	498.2671	249.6372	5
11	1081.4830	541.2451	1064.4565	532.7319	1063.4725	532.2399	Q	**429.2456**	215.1264	**412.2191**	206.6132			4
12	1178.5358	589.7715	1161.5092	581.2583	1160.5252	580.7662	P	**301.1870**	151.0971	284.1605	142.5839			3
13	1235.5572	618.2823	1218.5307	609.7690	1217.5467	609.2770	G	204.1343	102.5708	187.1077	94.0575			2
14							K	147.1128	74.0600	130.0863	65.5468			1

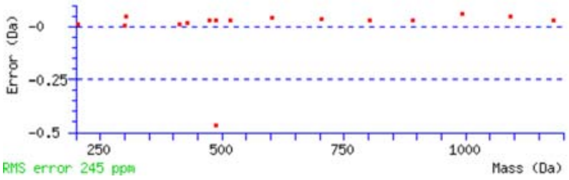

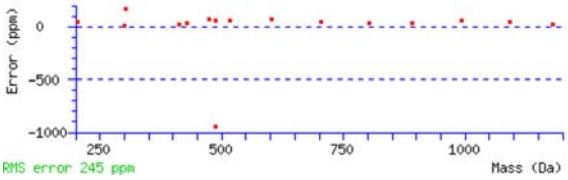

Peptide View

MS/MS Fragmentation of **ARPLEEALDVIVSTFHK**
Found in **gi|33859624|ref|NP_035441.1|**, S100 calcium binding protein A4 [Mus musculus]

Match to Query 485: 1976.159472 from(659.727100,3+)

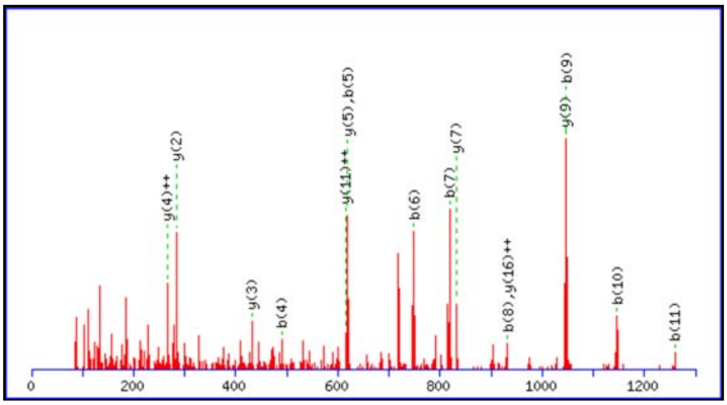

Monoisotopic mass of neutral peptide Mr(calc): 1976.0859
Variable modifications:
N-term : N-Acetyl (Protein)
R2 : Arginine-13C615N4 (R-full)
Ions Score: 64 Expect: 0.0016
Matches (**Bold Red**): 16/176 fragment ions using 24 most intense peaks

#	b	b^{++}	b*	b^{*++}	b^0	b^{0++}	Seq.	y	y^{++}	y*	y^{*++}	y^0	y^{0++}	#
1	114.0549	57.5311					A							17
2	280.1896	140.5984	263.1631	132.0852			R	1864.0455	**932.5264**	1847.0189	924.0131	1846.0349	923.5211	16
3	377.2424	189.1248	360.2158	180.6115			P	1697.9108	849.4590	1680.8843	840.9458	1679.9003	840.4538	15
4	**490.3264**	245.6669	473.2999	237.1536			L	1600.8581	800.9327	1583.8315	792.4194	1582.8475	791.9274	14
5	**619.3690**	310.1881	602.3425	301.6749	601.3585	301.1829	E	1487.7740	744.3906	1470.7475	735.8774	1469.7634	735.3854	13
6	**748.4116**	374.7094	731.3851	366.1962	730.4010	365.7042	E	1358.7314	679.8693	1341.7049	671.3561	1340.7208	670.8641	12
7	**819.4487**	410.2280	802.4222	401.7147	801.4382	401.2227	A	1229.6888	**615.3480**	1212.6623	606.8348	1211.6783	606.3428	11
8	**932.5328**	466.7700	915.5062	458.2568	914.5222	457.7647	L	1158.6517	579.8295	1141.6252	571.3162	1140.6411	570.8242	10
9	**1047.5597**	524.2835	1030.5332	515.7702	1029.5492	515.2782	D	**1045.5677**	523.2875	1028.5411	514.7742	1027.5571	514.2822	9
10	**1146.6281**	573.8177	1129.6016	565.3044	1128.6176	564.8124	V	930.5407	465.7740	913.5142	457.2607	912.5301	456.7687	8
11	**1259.7122**	630.3597	1242.6856	621.8465	1241.7016	621.3545	I	**831.4723**	416.2398	814.4458	407.7265	813.4617	407.2345	7
12	1358.7806	679.8939	1341.7541	671.3807	1340.7700	670.8887	V	718.3882	359.6978	701.3617	351.1845	700.3777	350.6925	6
13	1445.8126	723.4100	1428.7861	714.8967	1427.8021	714.4047	S	**619.3198**	310.1636	602.2933	301.6503	601.3093	301.1583	5
14	1546.8603	773.9338	1529.8338	765.4205	1528.8497	764.9285	T	532.2878	**266.6475**	515.2613	258.1343	514.2772	257.6423	4
15	1693.9287	847.4680	1676.9022	838.9547	1675.9182	838.4627	F	**431.2401**	216.1237	414.2136	207.6104			3
16	1830.9876	915.9975	1813.9611	907.4842	1812.9771	906.9922	H	**284.1717**	142.5895	267.1452	134.0762			2
17							K	147.1128	74.0600	130.0863	65.5468			1

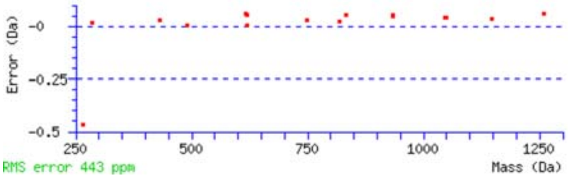

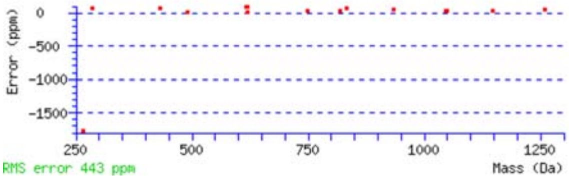

Peptide View

MS/MS Fragmentation of **AVDLLNYTQWFPIVIFFNPDSR**
Found in **gi|7549795|ref|NP_035727.1|**, tight junction protein 2 [Mus musculus]

Match to Query 786: 2660.383248 from(1331.198900,2+)

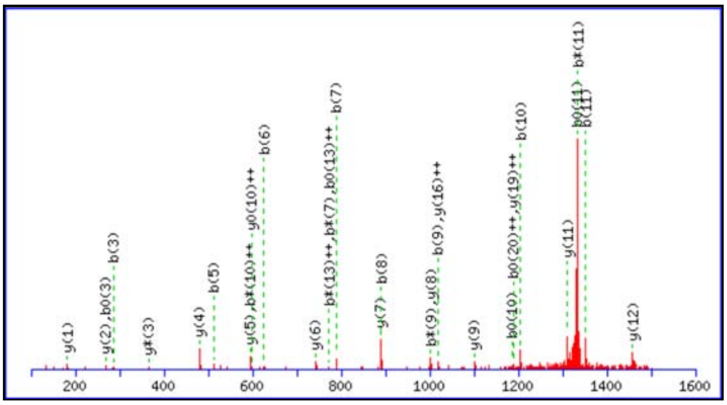

Monoisotopic mass of neutral peptide Mr(calc): 2660.3734
Variable modifications:
R22 : Arginine-13C6 (R-13C6)
Ions Score: 57 Expect: 0.016
Matches (**Bold Red**): 32/236 fragment ions using 66 most intense peaks

#	b	b^{++}	b*	$b*^{++}$	b^0	b^{0++}	Seq.	y	y^{++}	y*	$y*^{++}$	y^0	y^{0++}	#
1	72.0444	36.5258					A							22
2	171.1128	86.0600					V	2590.3436	1295.6754	2573.3170	1287.1621	2572.3330	1286.6701	21
3	**286.1397**	143.5735			**268.1292**	134.5682	D	2491.2751	1246.1412	2474.2486	1237.6279	2473.2646	1237.1359	20
4	399.2238	200.1155			381.2132	191.1103	L	2376.2482	**1188.6277**	2359.2217	1180.1145	2358.2376	1179.6225	19
5	**512.3079**	256.6576			494.2973	247.6523	L	2263.1641	1132.0857	2246.1376	1123.5724	2245.1536	1123.0804	18
6	**626.3508**	313.6790	609.3242	305.1658	608.3402	304.6737	N	2150.0801	1075.5437	2133.0535	1067.0304	2132.0695	1066.5384	17
7	**789.4141**	395.2107	**772.3876**	386.6974	771.4036	386.2054	Y	2036.0372	**1018.5222**	2019.0106	1010.0089	2018.0266	1009.5169	16
8	**890.4618**	445.7345	873.4352	437.2213	872.4512	436.7293	T	1872.9738	936.9906	1855.9473	928.4773	1854.9633	927.9853	15
9	**1018.5204**	509.7638	**1001.4938**	501.2506	1000.5098	500.7585	Q	1771.9261	886.4667	1754.8996	877.9534	1753.9156	877.4614	14
10	**1204.5997**	602.8035	1187.5731	**594.2902**	**1186.5891**	593.7982	W	1643.8676	822.4374	1626.8410	813.9241	1625.8570	813.4321	13
11	**1351.6681**	676.3377	**1334.6415**	667.8244	**1333.6575**	667.3324	F	**1457.7883**	729.3978	1440.7617	720.8845	1439.7777	720.3925	12
12	1448.7209	724.8641	1431.6943	716.3508	1430.7103	715.8588	P	**1310.7198**	655.8636	1293.6933	647.3503	1292.7093	646.8583	11
13	1561.8049	781.4061	1544.7784	**772.8928**	1543.7944	**772.4008**	I	1213.6671	607.3372	1196.6405	598.8239	1195.6565	**598.3319**	10
14	1660.8733	830.9403	1643.8468	822.4270	1642.8628	821.9350	V	**1100.5830**	550.7952	1083.5565	542.2819	1082.5725	541.7899	9
15	1773.9574	887.4823	1756.9308	878.9691	1755.9468	878.4770	I	**1001.5146**	501.2609	984.4881	492.7477	983.5041	492.2557	8
16	1921.0258	961.0165	1903.9992	952.5033	1903.0152	952.0113	F	**888.4306**	444.7189	871.4040	436.2056	870.4200	435.7136	7
17	2068.0942	1034.5507	2051.0677	1026.0375	2050.0836	1025.5455	F	**741.3621**	371.1847	724.3356	362.6714	723.3516	362.1794	6
18	2182.1371	1091.5722	2165.1106	1083.0589	2164.1266	1082.5669	N	**594.2937**	297.6505	577.2672	289.1372	576.2832	288.6452	5
19	2279.1899	1140.0986	2262.1633	1131.5853	2261.1793	1131.0933	P	**480.2508**	240.6290	463.2243	232.1158	462.2402	231.6238	4
20	2394.2168	1197.6121	2377.1903	1189.0988	2376.2063	**1188.6068**	D	383.1980	192.1027	**366.1715**	183.5894	365.1875	183.0974	3
21	2481.2489	1241.1281	2464.2223	1232.6148	2463.2383	1232.1228	S	**268.1711**	134.5892	251.1446	126.0759	250.1605	125.5839	2
22							R	**181.1391**	91.0732	164.1125	82.5599			1

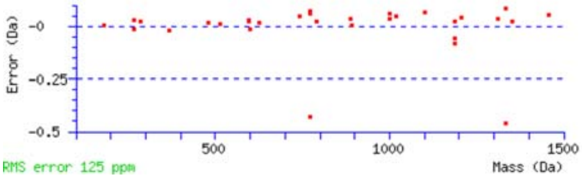

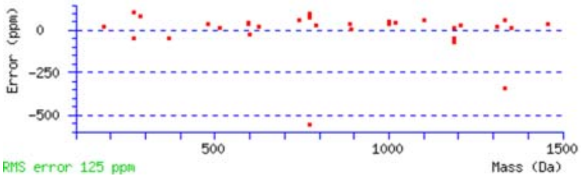

Peptide View

MS/MS Fragmentation of **EFATLIIDILSEAK**
Found in **gi|51921285|ref|NP_001004144.1|**, G protein-coupled receptor kinase-interactor 1 [Mus musculus]

Match to Query 338: 1561.937048 from(781.975800,2+)

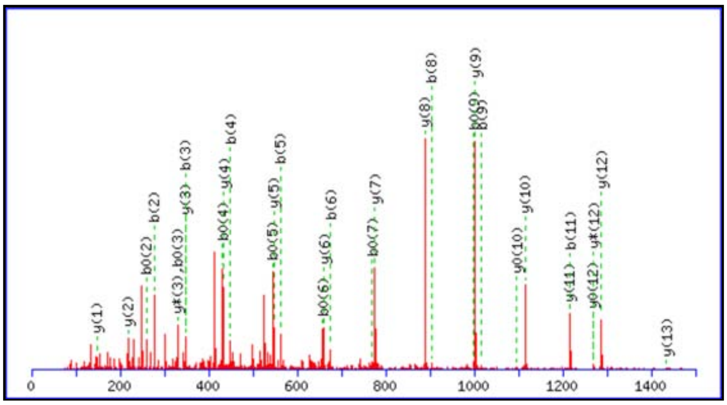

Monoisotopic mass of neutral peptide Mr(calc): 1561.8603
Ions Score: 68 Expect: 0.00033
Matches (**Bold Red**): 32/126 fragment ions using 90 most intense peaks

#	b	b^{++}	b^0	b^{0++}	Seq.	y	y^{++}	y*	y^{*++}	y^0	y^{0++}	#
1	130.0499	65.5286	112.0393	56.5233	E							14
2	**277.1183**	139.0628	**259.1077**	130.0575	F	**1433.8250**	717.4161	1416.7984	708.9028	1415.8144	708.4108	13
3	**348.1554**	174.5813	**330.1448**	165.5760	A	**1286.7566**	643.8819	**1269.7300**	635.3686	**1268.7460**	634.8766	12
4	**449.2031**	225.1052	**431.1925**	216.0999	T	**1215.7195**	608.3634	1198.6929	599.8501	1197.7089	599.3581	11
5	**562.2871**	281.6472	**544.2766**	272.6419	L	**1114.6718**	557.8395	1097.6452	549.3262	**1096.6612**	548.8342	10
6	**675.3712**	338.1892	**657.3606**	329.1839	I	**1001.5877**	501.2975	984.5612	492.7842	983.5771	492.2922	9
7	788.4552	394.7313	**770.4447**	385.7260	I	**888.5037**	444.7555	871.4771	436.2422	870.4931	435.7502	8
8	**903.4822**	452.2447	885.4716	443.2394	D	**775.4196**	388.2134	758.3930	379.7002	757.4090	379.2082	7
9	**1016.5662**	508.7868	**998.5557**	499.7815	I	**660.3927**	330.7000	643.3661	322.1867	642.3821	321.6947	6
10	1129.6503	565.3288	1111.6397	556.3235	L	**547.3086**	274.1579	530.2820	265.6447	529.2980	265.1527	5
11	**1216.6823**	608.8448	1198.6718	599.8395	S	**434.2245**	217.6159	417.1980	209.1026	416.2140	208.6106	4
12	1345.7249	673.3661	1327.7144	664.3608	E	**347.1925**	174.0999	**330.1660**	165.5866	329.1819	165.0946	3
13	1416.7620	708.8847	1398.7515	699.8794	A	**218.1499**	109.5786	201.1234	101.0653			2
14					K	**147.1128**	74.0600	130.0863	65.5468			1

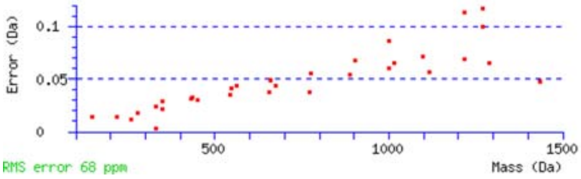

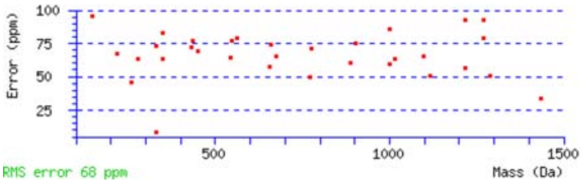

Peptide View

MS/MS Fragmentation of **ELFALDLVTGVLTIK**
Found in **gi|18087747|ref|NP_291059.1|**, protocadherin gamma subfamily C, 3 [Mus musculus]

Match to Query 540: 1630.984648 from(816.499600,2+)

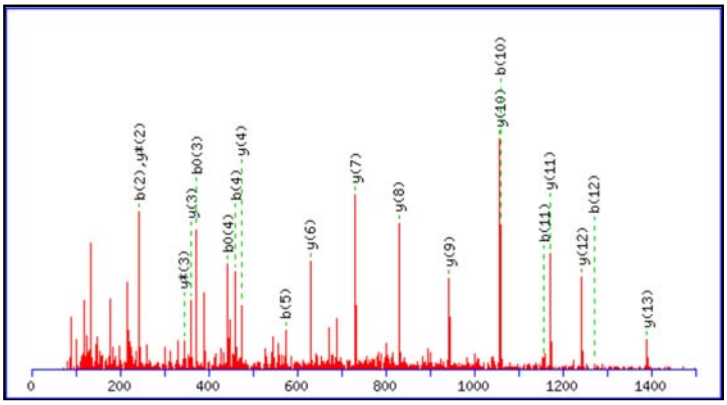

Monoisotopic mass of neutral peptide Mr(calc): 1630.9545
Ions Score: 70 Expect: 0.00025
Matches (**Bold Red**): 20/136 fragment ions using 43 most intense peaks

#	b	b^{++}	b^{0}	b^{0++}	Seq.	y	y^{++}	y*	$y*^{++}$	y^{0}	y^{0++}	#
1	130.0499	65.5286	112.0393	56.5233	E							15
2	**243.1339**	122.0706	225.1234	113.0653	L	1502.9192	751.9632	1485.8927	743.4500	1484.9086	742.9580	14
3	390.2023	195.6048	**372.1918**	186.5995	F	**1389.8351**	695.4212	1372.8086	686.9079	1371.8246	686.4159	13
4	**461.2394**	231.1234	**443.2289**	222.1181	A	**1242.7667**	621.8870	1225.7402	613.3737	1224.7562	612.8817	12
5	**574.3235**	287.6654	556.3129	278.6601	L	**1171.7296**	586.3684	1154.7031	577.8552	1153.7191	577.3632	11
6	689.3504	345.1789	671.3399	336.1736	D	**1058.6456**	529.8264	1041.6190	521.3131	1040.6350	520.8211	10
7	802.4345	401.7209	784.4239	392.7156	L	**943.6186**	472.3129	926.5921	463.7997	925.6081	463.3077	9
8	901.5029	451.2551	883.4924	442.2498	V	**830.5346**	415.7709	813.5080	407.2576	812.5240	406.7656	8
9	1002.5506	501.7789	984.5400	492.7737	T	**731.4662**	366.2367	714.4396	357.7234	713.4556	357.2314	7
10	**1059.5721**	530.2897	1041.5615	521.2844	G	**630.4185**	315.7129	613.3919	307.1996	612.4079	306.7076	6
11	**1158.6405**	579.8239	1140.6299	570.8186	V	573.3970	287.2021	556.3705	278.6889	555.3864	278.1969	5
12	**1271.7245**	636.3659	1253.7140	627.3606	L	**474.3286**	237.6679	457.3021	229.1547	456.3180	228.6627	4
13	1372.7722	686.8897	1354.7616	677.8845	T	**361.2445**	181.1259	**344.2180**	172.6126	343.2340	172.1206	3
14	1485.8563	743.4318	1467.8457	734.4265	I	260.1969	130.6021	**243.1703**	122.0888			2
15					K	147.1128	74.0600	130.0863	65.5468			1

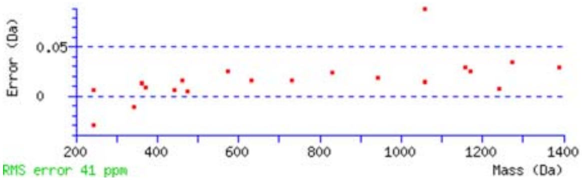

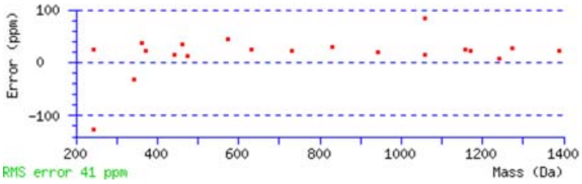

Peptide View

MS/MS Fragmentation of EQFALAMYFIQQK
Found in gi|6679669|ref|NP_031970.1|, epidermal growth factor receptor pathway substrate 15, related [Mus musculus]

Match to Query 924: 1615.898248 from(808.956400,2+)

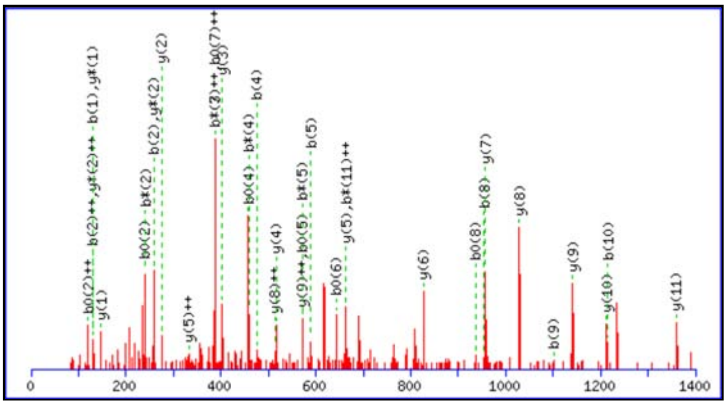

Monoisotopic mass of neutral peptide Mr(calc): 1615.8068
Ions Score: 51 Expect: 0.015
Matches (**Bold Red**): 39/118 fragment ions using 87 most intense peaks

#	b	b^{++}	b*	b^{*++}	b^0	b^{0++}	Seq.	y	y^{++}	y*	y^{*++}	#
1	**130.0499**	65.5286			112.0393	56.5233	E					13
2	**258.1084**	**129.5579**	**241.0819**	121.0446	**240.0979**	**120.5526**	Q	1487.7715	744.3894	1470.7450	735.8761	12
3	405.1769	203.0921	**388.1503**	194.5788	**387.1663**	194.0868	F	**1359.7129**	680.3601	1342.6864	671.8468	11
4	**476.2140**	238.6106	**459.1874**	230.0973	**458.2034**	229.6053	A	**1212.6445**	606.8259	1195.6180	598.3126	10
5	**589.2980**	295.1527	**572.2715**	286.6394	**571.2875**	286.1474	L	**1141.6074**	**571.3073**	1124.5809	562.7941	9
6	660.3351	330.6712	643.3086	322.1579	**642.3246**	321.6659	A	**1028.5234**	**514.7653**	1011.4968	506.2520	8
7	791.3756	396.1914	774.3491	**387.6782**	773.3651	**387.1862**	M	**957.4862**	479.2468	940.4597	470.7335	7
8	**954.4389**	477.7231	937.4124	469.2098	**936.4284**	468.7178	Y	**826.4458**	413.7265	809.4192	405.2132	6
9	**1101.5074**	551.2573	1084.4808	542.7440	1083.4968	542.2520	F	**663.3824**	**332.1949**	646.3559	323.6816	5
10	**1214.5914**	607.7993	1197.5649	599.2861	1196.5809	598.7941	I	**516.3140**	258.6606	499.2875	250.1474	4
11	1342.6500	671.8286	1325.6234	**663.3154**	1324.6394	662.8234	Q	**403.2300**	202.1186	386.2034	193.6053	3
12	1470.7086	735.8579	1453.6820	727.3447	1452.6980	726.8526	Q	**275.1714**	138.0893	**258.1448**	**129.5761**	2
13							K	**147.1128**	74.0600	**130.0863**	65.5468	1

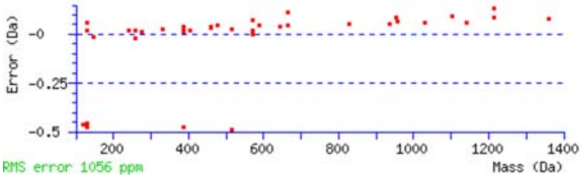

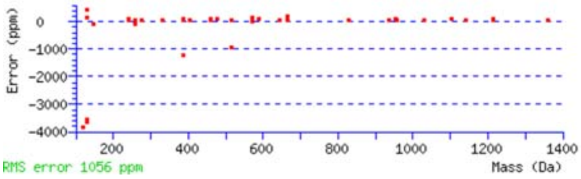

Peptide View

MS/MS Fragmentation of FFEVILIDPFHK
Found in gi|94385783|ref|XP_001004374.1|, PREDICTED: similar to ribosomal protein L15 [Mus musculus]

Match to Query 807: 1503.900648 from(752.957600,2+)

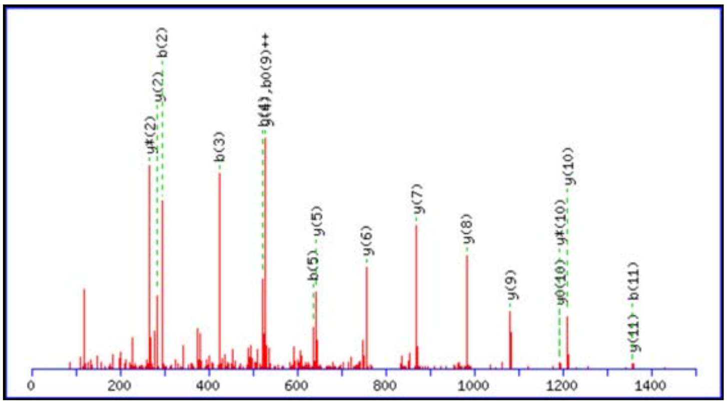

Monoisotopic mass of neutral peptide Mr(calc): 1503.8125
Ions Score: 60 **Expect:** 0.0018
Matches (Bold Red): 18/98 fragment ions using 40 most intense peaks

#	b	b^{++}	b^{0}	b^{0++}	Seq.	y	y^{++}	y*	$y*^{++}$	y^{0}	y^{0++}	#
1	148.0757	74.5415			F							12
2	**295.1441**	148.0757			F	**1357.7514**	679.3793	1340.7249	670.8661	1339.7408	670.3741	11
3	**424.1867**	212.5970	406.1761	203.5917	E	**1210.6830**	605.8451	**1193.6565**	597.3319	**1192.6724**	596.8399	10
4	**523.2551**	262.1312	505.2445	253.1259	V	**1081.6404**	541.3238	1064.6139	532.8106	1063.6298	532.3186	9
5	**636.3392**	318.6732	618.3286	309.6679	I	**982.5720**	491.7896	965.5455	483.2764	964.5614	482.7844	8
6	749.4232	375.2152	731.4127	366.2100	L	**869.4879**	435.2476	852.4614	426.7343	851.4774	426.2423	7
7	862.5073	431.7573	844.4967	422.7520	I	**756.4039**	378.7056	739.3773	370.1923	738.3933	369.7003	6
8	977.5342	489.2707	959.5237	480.2655	D	**643.3198**	322.1635	626.2933	313.6503	625.3093	313.1583	5
9	1074.5870	537.7971	1056.5764	**528.7918**	P	**528.2929**	264.6501	511.2663	256.1368			4
10	1221.6554	611.3313	1203.6448	602.3260	F	431.2401	216.1237	414.2136	207.6104			3
11	**1358.7143**	679.8608	1340.7037	670.8555	H	**284.1717**	142.5895	**267.1452**	134.0762			2
12					K	147.1128	74.0600	130.0863	65.5468			1

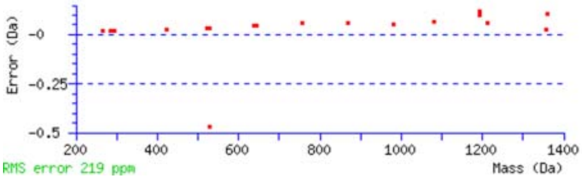

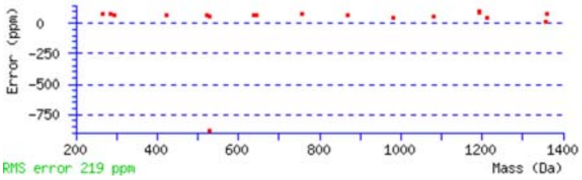

Peptide View

MS/MS Fragmentation of FGSAIPIPSLPDK
Found in gi|29568084|ref|NP_079940.2|, sorting nexin 9 [Mus musculus]

Match to Query 224: 1340.792648 from(671.403600,2+)

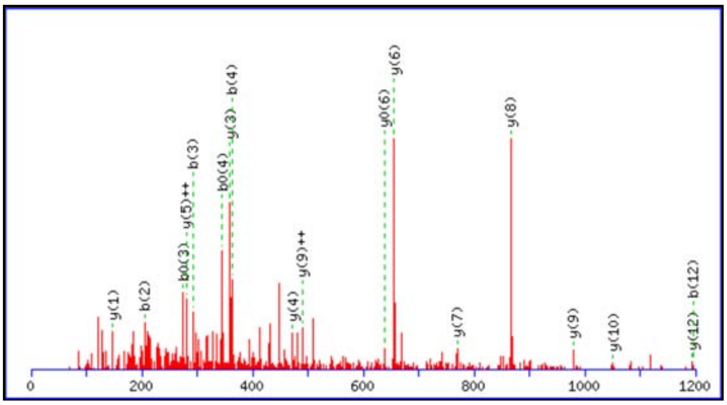

Monoisotopic mass of neutral peptide Mr(calc): 1340.7340
Ions Score: 46 Expect: 0.033
Matches (**Bold Red**): 18/114 fragment ions using 42 most intense peaks

#	b	b^{++}	b^{0}	b^{0++}	Seq.	y	y^{++}	y*	y^{*++}	y^{0}	y^{0++}	#
1	148.0757	74.5415			F							13
2	**205.0971**	103.0522			G	**1194.6728**	597.8401	1177.6463	589.3268	1176.6623	588.8348	12
3	**292.1292**	146.5682	**274.1186**	137.5629	S	1137.6514	569.3293	1120.6248	560.8160	1119.6408	560.3240	11
4	**363.1663**	182.0868	**345.1557**	173.0815	A	**1050.6193**	525.8133	1033.5928	517.3000	1032.6088	516.8080	10
5	476.2503	238.6288	458.2398	229.6235	I	**979.5822**	**490.2948**	962.5557	481.7815	961.5717	481.2895	9
6	573.3031	287.1552	555.2925	278.1499	P	**866.4982**	433.7527	849.4716	425.2394	848.4876	424.7474	8
7	686.3872	343.6972	668.3766	334.6919	I	**769.4454**	385.2263	752.4189	376.7131	751.4348	376.2211	7
8	783.4399	392.2236	765.4294	383.2183	P	**656.3614**	328.6843	639.3348	320.1710	**638.3508**	319.6790	6
9	870.4720	435.7396	852.4614	426.7343	S	559.3086	**280.1579**	542.2820	271.6447	541.2980	271.1527	5
10	983.5560	492.2816	965.5455	483.2764	L	**472.2766**	236.6419	455.2500	228.1286	454.2660	227.6366	4
11	1080.6088	540.8080	1062.5982	531.8027	P	**359.1925**	180.0999	342.1660	171.5866	341.1819	171.0946	3
12	**1195.6357**	598.3215	1177.6252	589.3162	D	262.1397	131.5735	245.1132	123.0602	244.1292	122.5682	2
13					K	**147.1128**	74.0600	130.0863	65.5468			1

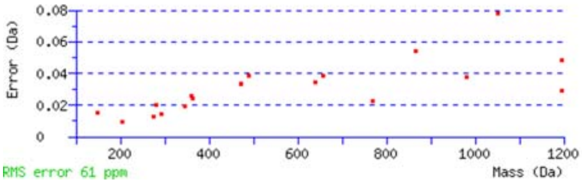

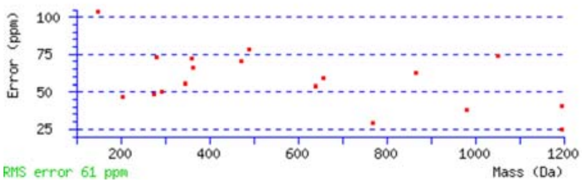

Peptide View

MS/MS Fragmentation of FGVEQDVDMVFASFIR
Found in gi|31981562|ref|NP_035229.2|, pyruvate kinase 3 [Mus musculus]

Match to Query 611: 1858.988248 from(930.501400,2+)

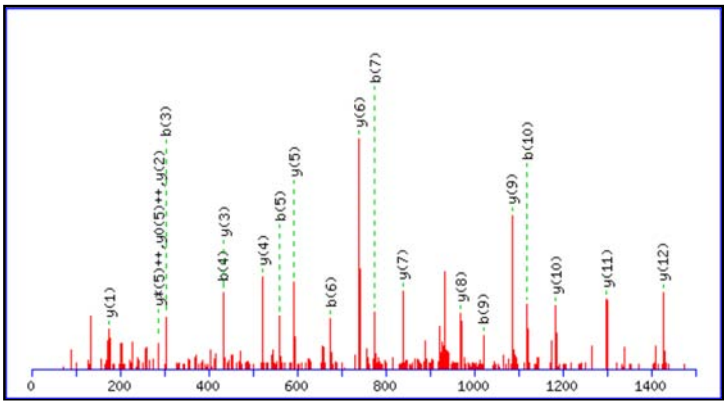

Monoisotopic mass of neutral peptide Mr(calc): 1858.8923
Ions Score: 88 Expect: 5.1e-006
Matches (Bold Red): 21/160 fragment ions using 37 most intense peaks

#	b	b^{++}	b*	b^{*++}	b^{0}	b^{0++}	Seq.	y	y^{++}	y*	y^{*++}	y^{0}	y^{0++}	#
1	148.0757	74.5415					F							16
2	205.0971	103.0522					G	1712.8312	856.9192	1695.8046	848.4060	1694.8206	847.9140	15
3	**304.1656**	152.5864					V	1655.8097	828.4085	1638.7832	819.8952	1637.7992	819.4032	14
4	**433.2081**	217.1077			415.1976	208.1024	E	1556.7413	778.8743	1539.7148	770.3610	1538.7308	769.8690	13
5	**561.2667**	281.1370	544.2402	272.6237	543.2562	272.1317	Q	**1427.6987**	714.3530	1410.6722	705.8397	1409.6882	705.3477	12
6	**676.2937**	338.6505	659.2671	330.1372	658.2831	329.6452	D	**1299.6402**	650.3237	1282.6136	641.8104	1281.6296	641.3184	11
7	**775.3621**	388.1847	758.3355	379.6714	757.3515	379.1794	V	**1184.6132**	592.8102	1167.5867	584.2970	1166.6026	583.8050	10
8	890.3890	445.6981	873.3625	437.1849	872.3785	436.6929	D	**1085.5448**	543.2760	1068.5183	534.7628	1067.5342	534.2708	9
9	**1021.4295**	511.2184	1004.4029	502.7051	1003.4189	502.2131	M	**970.5179**	485.7626	953.4913	477.2493	952.5073	476.7573	8
10	**1120.4979**	560.7526	1103.4714	552.2393	1102.4873	551.7473	V	**839.4774**	420.2423	822.4508	411.7291	821.4668	411.2370	7
11	1267.5663	634.2868	1250.5398	625.7735	1249.5558	625.2815	F	**740.4090**	370.7081	723.3824	362.1948	722.3984	361.7028	6
12	1338.6034	669.8054	1321.5769	661.2921	1320.5929	660.8001	A	**593.3406**	297.1739	576.3140	**288.6606**	575.3300	**288.1686**	5
13	1425.6355	713.3214	1408.6089	704.8081	1407.6249	704.3161	S	**522.3035**	261.6554	505.2769	253.1421	504.2929	252.6501	4
14	1572.7039	786.8556	1555.6773	778.3423	1554.6933	777.8503	F	**435.2714**	218.1393	418.2449	209.6261			3
15	1685.7879	843.3976	1668.7614	834.8843	1667.7774	834.3923	I	**288.2030**	144.6051	271.1765	136.0919			2
16							R	**175.1190**	88.0631	158.0924	79.5498			1

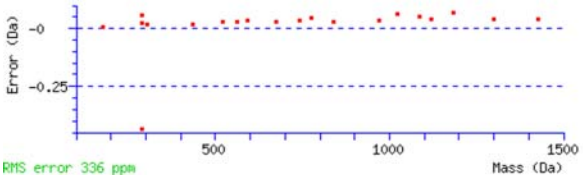

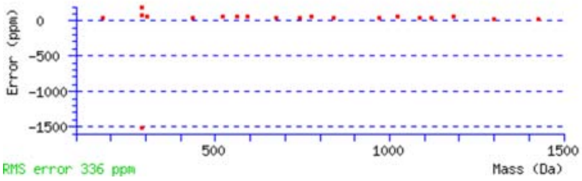

Peptide View

MS/MS Fragmentation of FIENLLASDGDFWIGLK
Found in gi|94385870|ref|XP_922372.2|, PREDICTED: similar to layilin [Mus musculus]

Match to Query 468: 1937.087048 from(969.550800,2+)

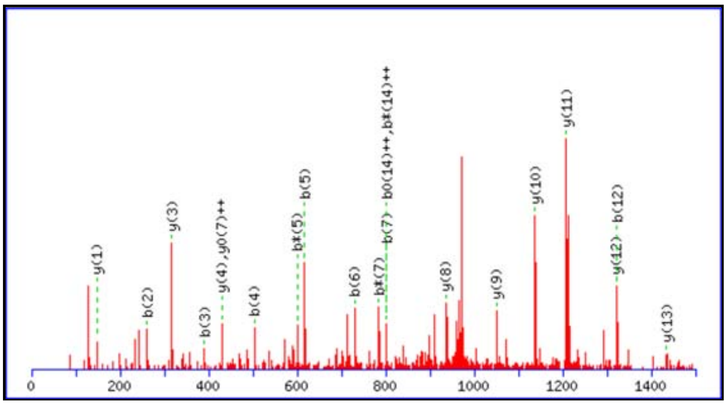

Monoisotopic mass of neutral peptide Mr(calc): 1936.9934
Ions Score: 53 Expect: 0.018
Matches (Bold Red): 21/170 fragment ions using 52 most intense peaks

#	b	b^{++}	b*	b^{*++}	b^0	b^{0++}	Seq.	y	y^{++}	y*	y^{*++}	y^0	y^{0++}	#
1	148.0757	74.5415					F							17
2	**261.1597**	131.0835					I	1790.9323	895.9698	1773.9057	887.4565	1772.9217	886.9645	16
3	**390.2023**	195.6048			372.1918	186.5995	E	1677.8482	839.4277	1660.8217	830.9145	1659.8377	830.4225	15
4	**504.2453**	252.6263	487.2187	244.1130	486.2347	243.6210	N	1548.8056	774.9065	1531.7791	766.3932	1530.7951	765.9012	14
5	**617.3293**	309.1683	**600.3028**	300.6550	599.3188	300.1630	L	**1434.7627**	717.8850	1417.7362	709.3717	1416.7521	708.8797	13
6	**730.4134**	365.7103	713.3868	357.1971	712.4028	356.7050	L	**1321.6786**	661.3430	1304.6521	652.8297	1303.6681	652.3377	12
7	**801.4505**	401.2289	**784.4239**	392.7156	783.4399	392.2236	A	**1208.5946**	604.8009	1191.5680	596.2877	1190.5840	595.7956	11
8	888.4825	444.7449	871.4560	436.2316	870.4720	435.7396	S	**1137.5575**	569.2824	1120.5309	560.7691	1119.5469	560.2771	10
9	1003.5095	502.2584	986.4829	493.7451	985.4989	493.2531	D	**1050.5254**	525.7664	1033.4989	517.2531	1032.5149	516.7611	9
10	1060.5309	530.7691	1043.5044	522.2558	1042.5204	521.7638	G	**935.4985**	468.2529	918.4720	459.7396	917.4879	459.2476	8
11	1175.5579	588.2826	1158.5313	579.7693	1157.5473	579.2773	D	878.4770	439.7422	861.4505	431.2289	860.4665	**430.7369**	7
12	**1322.6263**	661.8168	1305.5997	653.3035	1304.6157	652.8115	F	763.4501	382.2287	746.4236	373.7154			6
13	1508.7056	754.8564	1491.6790	746.3432	1490.6950	745.8511	W	616.3817	308.6945	599.3551	300.1812			5
14	1621.7896	811.3985	1604.7631	**802.8852**	1603.7791	**802.3932**	I	**430.3024**	215.6548	413.2758	207.1416			4
15	1678.8111	839.9092	1661.7846	831.3959	1660.8005	830.9039	G	**317.2183**	159.1128	300.1918	150.5995			3
16	1791.8952	896.4512	1774.8686	887.9379	1773.8846	887.4459	L	260.1969	130.6021	243.1703	122.0888			2
17							K	**147.1128**	74.0600	130.0863	65.5468			1

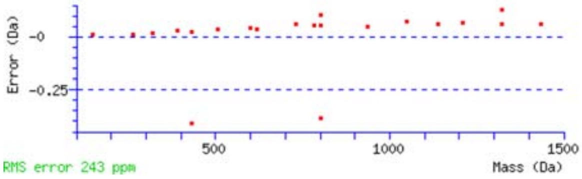

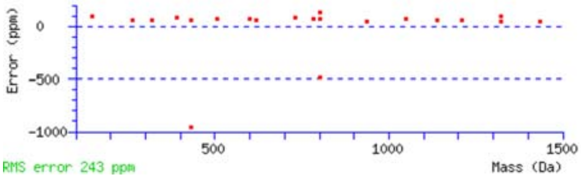

Peptide View

MS/MS Fragmentation of FINMFAVLDELK
Found in gi|84370256|ref|NP_598530.2|, cytoplasmic FMR1 interacting protein 2 [Mus musculus]

Match to Query 288: 1438.826848 from(720.420700,2+)

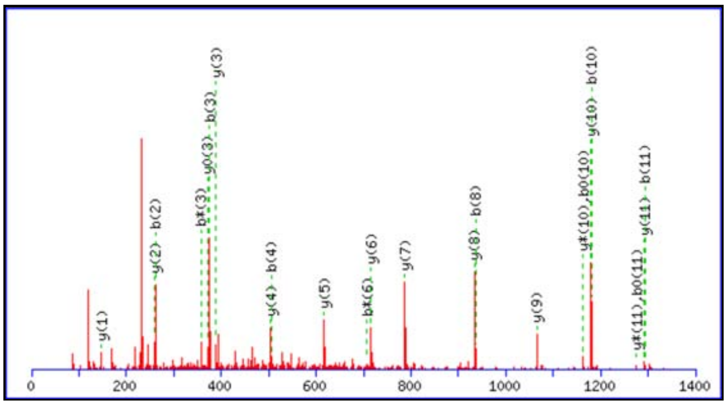

Monoisotopic mass of neutral peptide Mr(calc): 1438.7530
Ions Score: 65 **Expect:** 0.00052
Matches (**Bold Red**): 24/108 fragment ions using 57 most intense peaks

#	b	b^{++}	b*	b^{*++}	b^{0}	b^{0++}	Seq.	y	y^{++}	y*	y^{*++}	y^{0}	y^{0++}	#
1	148.0757	74.5415					F							12
2	**261.1597**	131.0835					I	**1292.6919**	646.8496	**1275.6653**	638.3363	1274.6813	637.8443	11
3	**375.2027**	188.1050	**358.1761**	179.5917			N	**1179.6078**	590.3075	**1162.5812**	581.7943	1161.5972	581.3023	10
4	**506.2432**	253.6252	489.2166	245.1119			M	**1065.5649**	533.2861	1048.5383	524.7728	1047.5543	524.2808	9
5	653.3116	327.1594	636.2850	318.6461			F	**934.5244**	467.7658	917.4978	459.2526	916.5138	458.7605	8
6	724.3487	362.6780	**707.3221**	354.1647			A	**787.4560**	394.2316	770.4294	385.7183	769.4454	385.2263	7
7	823.4171	412.2122	806.3905	403.6989			V	**716.4189**	358.7131	699.3923	350.1998	698.4083	349.7078	6
8	**936.5011**	468.7542	919.4746	460.2409			L	**617.3505**	309.1789	600.3239	300.6656	599.3399	300.1736	5
9	1051.5281	526.2677	1034.5015	517.7544	1033.5175	517.2624	D	**504.2664**	252.6368	487.2398	244.1236	486.2558	243.6316	4
10	**1180.5707**	590.7890	1163.5441	582.2757	**1162.5601**	581.7837	E	**389.2395**	195.1234	372.2129	186.6101	**371.2289**	186.1181	3
11	**1293.6547**	647.3310	1276.6282	638.8177	**1275.6442**	638.3257	L	**260.1969**	130.6021	243.1703	122.0888			2
12							K	**147.1128**	74.0600	130.0863	65.5468			1

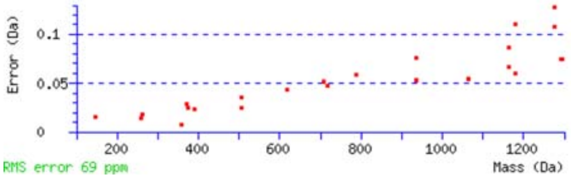

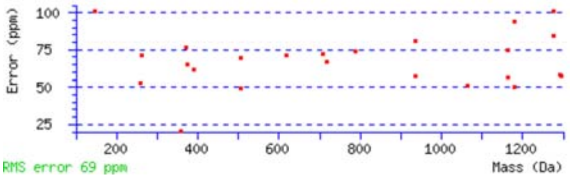

Peptide View

MS/MS Fragmentation of FLDGELPLDSFIDVYQSK
Found in gi|29244484|ref|NP_808544.1|, vacuolar protein sorting 37B [Mus musculus]

Match to Query 685: 2085.053848 from(1043.534200,2+)

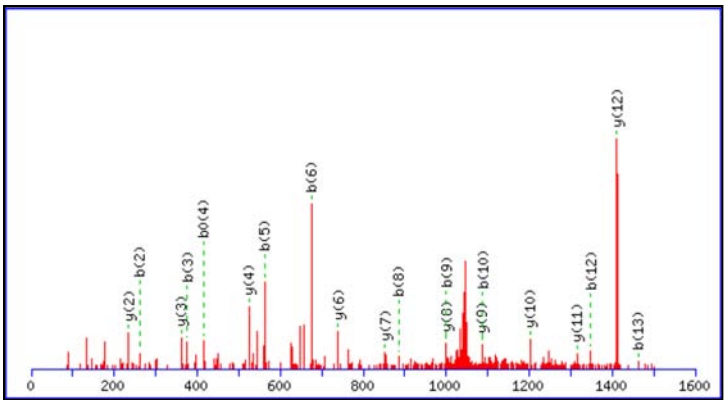

Monoisotopic mass of neutral peptide Mr(calc): 2085.0306
Ions Score: 57 **Expect:** 0.011
Matches (**Bold Red**): 20/168 fragment ions using 75 most intense peaks

#	b	b^{++}	b*	b*$^{++}$	b^{0}	b^{0++}	Seq.	y	y^{++}	y*	y*$^{++}$	y^{0}	y^{0++}	#
1	148.0757	74.5415					F							18
2	**261.1597**	131.0835					L	1938.9695	969.9884	1921.9429	961.4751	1920.9589	960.9831	17
3	**376.1867**	188.5970			358.1761	179.5917	D	1825.8854	913.4463	1808.8589	904.9331	1807.8748	904.4411	16
4	433.2081	217.1077			**415.1976**	208.1024	G	1710.8585	855.9329	1693.8319	847.4196	1692.8479	846.9276	15
5	**562.2507**	281.6290			544.2402	272.6237	E	1653.8370	827.4221	1636.8105	818.9089	1635.8264	818.4169	14
6	**675.3348**	338.1710			657.3242	329.1658	L	1524.7944	762.9008	1507.7679	754.3876	1506.7838	753.8956	13
7	772.3876	386.6974			754.3770	377.6921	P	**1411.7104**	706.3588	1394.6838	697.8455	1393.6998	697.3535	12
8	**885.4716**	443.2394			867.4611	434.2342	L	**1314.6576**	657.8324	1297.6310	649.3192	1296.6470	648.8272	11
9	**1000.4986**	500.7529			982.4880	491.7476	D	**1201.5735**	601.2904	1184.5470	592.7771	1183.5630	592.2851	10
10	**1087.5306**	544.2689			1069.5200	535.2636	S	**1086.5466**	543.7769	1069.5200	535.2637	1068.5360	534.7717	9
11	1234.5990	617.8031			1216.5884	608.7979	F	**999.5146**	500.2609	982.4880	491.7476	981.5040	491.2556	8
12	**1347.6831**	674.3452			1329.6725	665.3399	I	**852.4462**	426.7267	835.4196	418.2134	834.4356	417.7214	7
13	**1462.7100**	731.8586			1444.6994	722.8534	D	**739.3621**	370.1847	722.3355	361.6714	721.3515	361.1794	6
14	1561.7784	781.3928			1543.7678	772.3876	V	624.3352	312.6712	607.3086	304.1579	606.3246	303.6659	5
15	1724.8417	862.9245			1706.8312	853.9192	Y	**525.2667**	263.1370	508.2402	254.6237	507.2562	254.1317	4
16	1852.9003	926.9538	1835.8738	918.4405	1834.8898	917.9485	Q	**362.2034**	181.6053	345.1769	173.0921	344.1928	172.6001	3
17	1939.9323	970.4698	1922.9058	961.9565	1921.9218	961.4645	S	**234.1448**	117.5761	217.1183	109.0628	216.1343	108.5708	2
18							K	147.1128	74.0600	130.0863	65.5468			1

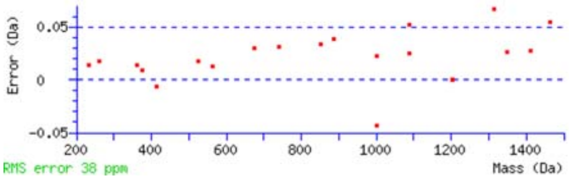

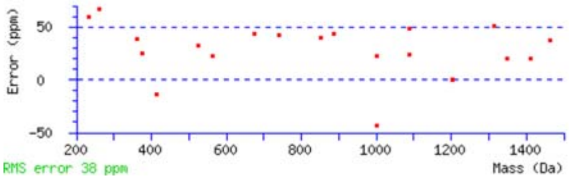

Peptide View

MS/MS Fragmentation of FYTNPSYFFDLWK
Found in gi|13994209|ref|NP_114083.1|, WASP family 1 [Mus musculus]

Match to Query 650: 1726.949048 from(864.481800,2+)

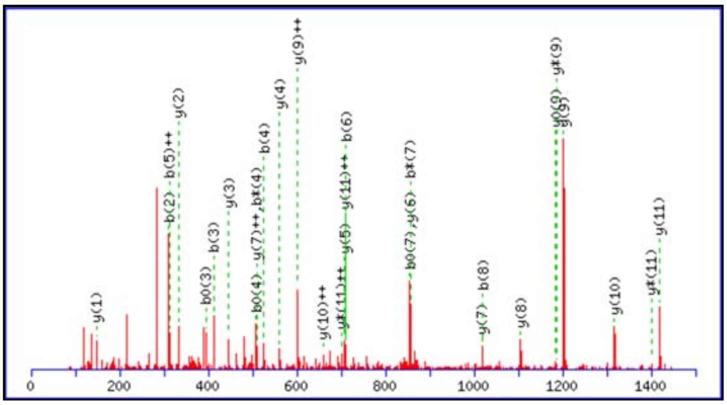

Monoisotopic mass of neutral peptide Mr(calc): 1726.8031
Ions Score: 49 Expect: 0.032
Matches (**Bold Red**): 30/128 fragment ions using 77 most intense peaks

#	b	b^{++}	b*	b^{*++}	b^{0}	b^{0++}	Seq.	y	y^{++}	y*	y^{*++}	y^{0}	y^{0++}	#
1	148.0757	74.5415					F							13
2	**311.1390**	156.0731					Y	1580.7420	790.8746	1563.7154	782.3614	1562.7314	781.8694	12
3	**412.1867**	206.5970			**394.1761**	197.5917	T	**1417.6787**	**709.3430**	**1400.6521**	**700.8297**	1399.6681	700.3377	11
4	**526.2296**	263.6185	**509.2031**	255.1052	**508.2191**	254.6132	N	**1316.6310**	**658.8191**	1299.6044	650.3059	1298.6204	649.8138	10
5	623.2824	**312.1448**	606.2558	303.6316	605.2718	303.1395	P	**1202.5881**	**601.7977**	**1185.5615**	593.2844	**1184.5775**	592.7924	9
6	**710.3144**	355.6608	693.2879	347.1476	692.3039	346.6556	S	**1105.5353**	553.2713	1088.5087	544.7580	1087.5247	544.2660	8
7	873.3777	437.1925	**856.3512**	428.6792	**855.3672**	428.1872	Y	**1018.5033**	**509.7553**	1001.4767	501.2420	1000.4927	500.7500	7
8	**1020.4462**	510.7267	1003.4196	502.2134	1002.4356	501.7214	F	**855.4399**	428.2236	838.4134	419.7103	837.4294	419.2183	6
9	1167.5146	584.2609	1150.4880	575.7476	1149.5040	575.2556	F	**708.3715**	354.6894	691.3450	346.1761	690.3610	345.6841	5
10	1282.5415	641.7744	1265.5150	633.2611	1264.5309	632.7691	D	**561.3031**	281.1552	544.2766	272.6419	543.2925	272.1499	4
11	1395.6256	698.3164	1378.5990	689.8031	1377.6150	689.3111	L	**446.2762**	223.6417	429.2496	215.1284			3
12	1581.7049	791.3561	1564.6783	782.8428	1563.6943	782.3508	W	**333.1921**	167.0997	316.1656	158.5864			2
13							K	**147.1128**	74.0600	130.0863	65.5468			1

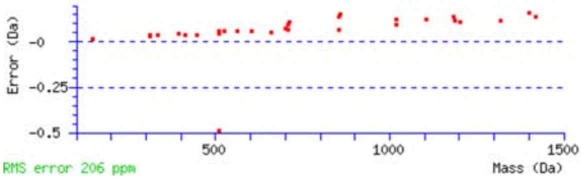

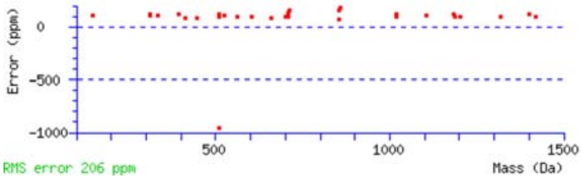

Peptide View

MS/MS Fragmentation of GLTVVQAFQELVEPK
Found in gi|19882207|ref|NP_608219.1|, farnesyl diphosphate synthetase [Mus musculus]

Match to Query 381: 1656.988248 from(829.501400,2+)

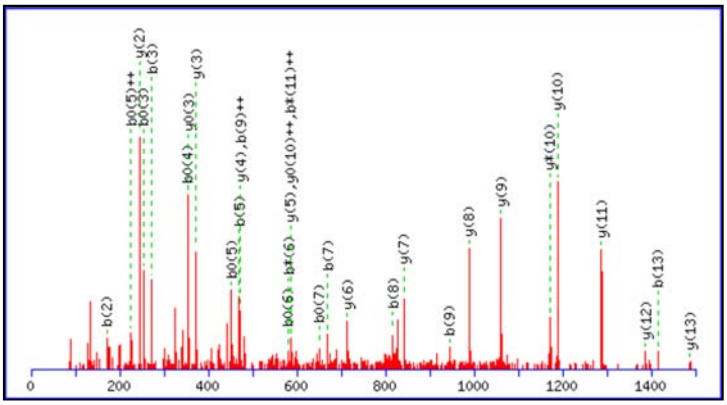

Monoisotopic mass of neutral peptide Mr(calc): 1656.9086
Ions Score: 88 **Expect:** 4e-006
Matches (Bold Red): 31/150 fragment ions using 49 most intense peaks

#	b	b^{++}	b*	b^{*++}	b^{0}	b^{0++}	Seq.	y	y^{++}	y*	y^{*++}	y^{0}	y^{0++}	#
1	58.0287	29.5180					G							15
2	**171.1128**	86.0600					L	1600.8945	800.9509	1583.8679	792.4376	1582.8839	791.9456	14
3	**272.1605**	136.5839			**254.1499**	127.5786	T	**1487.8104**	744.4088	1470.7838	735.8956	1469.7998	735.4036	13
4	371.2289	186.1181			**353.2183**	177.1128	V	**1386.7627**	693.8850	1369.7362	685.3717	1368.7521	684.8797	12
5	**470.2973**	235.6523			**452.2867**	**226.6470**	V	**1287.6943**	644.3508	1270.6678	635.8375	1269.6837	635.3455	11
6	598.3559	299.6816	**581.3293**	291.1683	**580.3453**	290.6763	Q	**1188.6259**	594.8166	**1171.5993**	586.3033	1170.6153	**585.8113**	10
7	**669.3930**	335.2001	652.3664	326.6869	**651.3824**	326.1948	A	**1060.5673**	530.7873	1043.5408	522.2740	1042.5567	521.7820	9
8	**816.4614**	408.7343	799.4348	400.2211	798.4508	399.7291	F	**989.5302**	495.2687	972.5037	486.7555	971.5196	486.2635	8
9	**944.5200**	**472.7636**	927.4934	464.2504	926.5094	463.7583	Q	**842.4618**	421.7345	825.4352	413.2213	824.4512	412.7293	7
10	1073.5626	537.2849	1056.5360	528.7716	1055.5520	528.2796	E	**714.4032**	357.7052	697.3767	349.1920	696.3926	348.7000	6
11	1186.6466	593.8270	1169.6201	**585.3137**	1168.6361	584.8217	L	**585.3606**	293.1839	568.3341	284.6707	567.3501	284.1787	5
12	1285.7150	643.3612	1268.6885	634.8479	1267.7045	634.3559	V	**472.2766**	236.6419	455.2500	228.1286	454.2660	227.6366	4
13	**1414.7576**	707.8825	1397.7311	699.3692	1396.7471	698.8772	E	**373.2082**	187.1077	356.1816	178.5944	**355.1976**	178.1024	3
14	1511.8104	756.4088	1494.7838	747.8956	1493.7998	747.4035	P	**244.1656**	122.5864	227.1390	114.0731			2
15							K	147.1128	74.0600	130.0863	65.5468			1

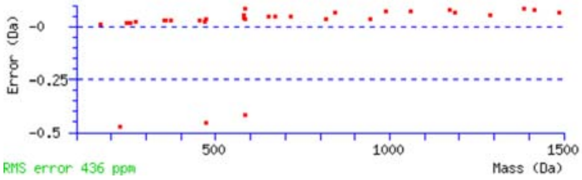

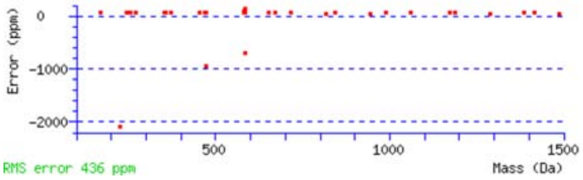

Peptide View

MS/MS Fragmentation of GMGGAFVLVLYDEIK
Found in gi|22094075|ref|NP_031477.1|, solute carrier family 25, member 5 [Mus musculus]

Match to Query 362: 1610.915648 from(806.465100,2+)

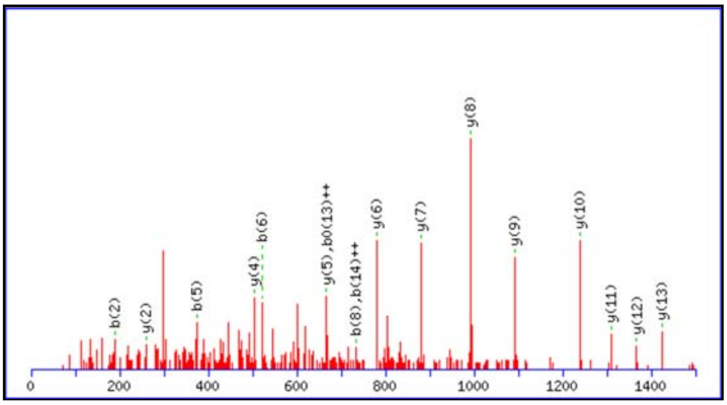

Monoisotopic mass of neutral peptide Mr(calc): 1610.8377
Ions Score: 73 **Expect:** 0.00011
Matches (**Bold Red**): 17/114 fragment ions using 32 most intense peaks

#	b	b++	b0	b0++	Seq.	y	y++	y*	y*++	y0	y0++	#
1	58.0287	29.5180			G							15
2	**189.0692**	95.0382			M	1554.8236	777.9154	1537.7970	769.4022	1536.8130	768.9101	14
3	246.0907	123.5490			G	**1423.7831**	712.3952	1406.7566	703.8819	1405.7725	703.3899	13
4	303.1121	152.0597			G	**1366.7616**	683.8845	1349.7351	675.3712	1348.7511	674.8792	12
5	**374.1492**	187.5783			A	**1309.7402**	655.3737	1292.7136	646.8605	1291.7296	646.3684	11
6	**521.2177**	261.1125			F	**1238.7031**	619.8552	1221.6765	611.3419	1220.6925	610.8499	10
7	620.2861	310.6467			V	**1091.6347**	546.3210	1074.6081	537.8077	1073.6241	537.3157	9
8	**733.3701**	367.1887			L	**992.5663**	496.7868	975.5397	488.2735	974.5557	487.7815	8
9	832.4385	416.7229			V	**879.4822**	440.2447	862.4556	431.7315	861.4716	431.2395	7
10	945.5226	473.2649			L	**780.4138**	390.7105	763.3872	382.1973	762.4032	381.7052	6
11	1108.5859	554.7966			Y	**667.3297**	334.1685	650.3032	325.6552	649.3192	325.1632	5
12	1223.6129	612.3101	1205.6023	603.3048	D	**504.2664**	252.6368	487.2398	244.1236	486.2558	243.6316	4
13	1352.6555	676.8314	1334.6449	**667.8261**	E	389.2395	195.1234	372.2129	186.6101	371.2289	186.1181	3
14	1465.7395	**733.3734**	1447.7290	724.3681	I	**260.1969**	130.6021	243.1703	122.0888			2
15					K	147.1128	74.0600	130.0863	65.5468			1

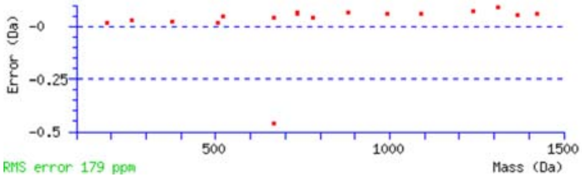

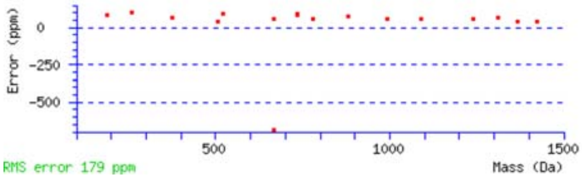

Peptide View

MS/MS Fragmentation of **GSLDTMLGLLQSDLSR**
Found in **gi|6678313|ref|NP_033391.1|**, transforming growth factor beta 1 induced transcript 1 [Mus musculus]

Match to Query 581: 1714.903448 from(858.459000,2+)

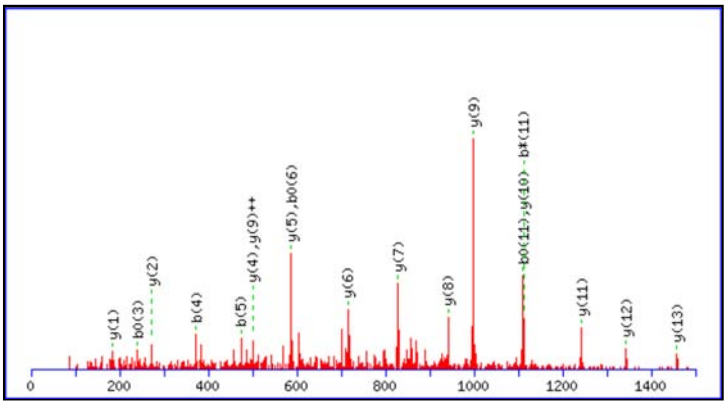

Monoisotopic mass of neutral peptide Mr(calc): 1714.9052
Variable modifications:
R16 : Arginine-13C615N4 (R-full)
Ions Score: 135 **Expect:** 8.7e-011
Matches (**Bold Red**): 19/156 fragment ions using 17 most intense peaks

#	b	b^{++}	b*	b^{*++}	b^0	b^{0++}	Seq.	y	y^{++}	y*	y^{*++}	y^0	y^{0++}	#
1	58.0287	29.5180					G							16
2	145.0608	73.0340			127.0502	64.0287	S	1658.8910	829.9491	1641.8644	821.4359	1640.8804	820.9438	15
3	258.1448	129.5761			**240.1343**	120.5708	L	1571.8589	786.4331	1554.8324	777.9198	1553.8484	777.4278	14
4	**373.1718**	187.0895			355.1612	178.0842	D	**1458.7749**	729.8911	1441.7483	721.3778	1440.7643	720.8858	13
5	**474.2194**	237.6134			456.2089	228.6081	T	**1343.7479**	672.3776	1326.7214	663.8643	1325.7374	663.3723	12
6	605.2599	303.1336			**587.2494**	294.1283	M	**1242.7003**	621.8538	1225.6737	613.3405	1224.6897	612.8485	11
7	718.3440	359.6756			700.3334	350.6703	L	**1111.6598**	556.3335	1094.6332	547.8203	1093.6492	547.3282	10
8	775.3654	388.1864			757.3549	379.1811	G	**998.5757**	**499.7915**	981.5492	491.2782	980.5652	490.7862	9
9	888.4495	444.7284			870.4389	435.7231	L	**941.5543**	471.2808	924.5277	462.7675	923.5437	462.2755	8
10	1001.5336	501.2704			983.5230	492.2651	L	**828.4702**	414.7387	811.4437	406.2255	810.4596	405.7335	7
11	1129.5921	565.2997	**1112.5656**	556.7864	**1111.5816**	556.2944	Q	**715.3861**	358.1967	698.3596	349.6834	697.3756	349.1914	6
12	1216.6242	608.8157	1199.5976	600.3025	1198.6136	599.8104	S	**587.3276**	294.1674	570.3010	285.6541	569.3170	285.1621	5
13	1331.6511	666.3292	1314.6246	657.8159	1313.6406	657.3239	D	**500.2955**	250.6514	483.2690	242.1381	482.2850	241.6461	4
14	1444.7352	722.8712	1427.7086	714.3580	1426.7246	713.8659	L	385.2686	193.1379	368.2420	184.6247	367.2580	184.1327	3
15	1531.7672	766.3872	1514.7407	757.8740	1513.7566	757.3820	S	**272.1845**	136.5959	255.1580	128.0826	254.1740	127.5906	2
16							R	**185.1525**	93.0799	168.1260	84.5666			1

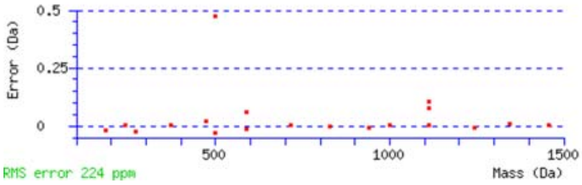

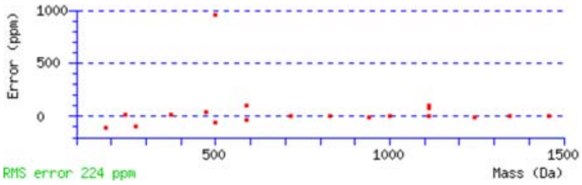

Peptide View

MS/MS Fragmentation of **GVFVSAFFSLLQTLSVK**
Found in **gi|31982724|ref|NP_058056.2|**, MYB binding protein (P160) 1a [Mus musculus]

Match to Query 1035: 1842.164848 from(922.089700,2+)

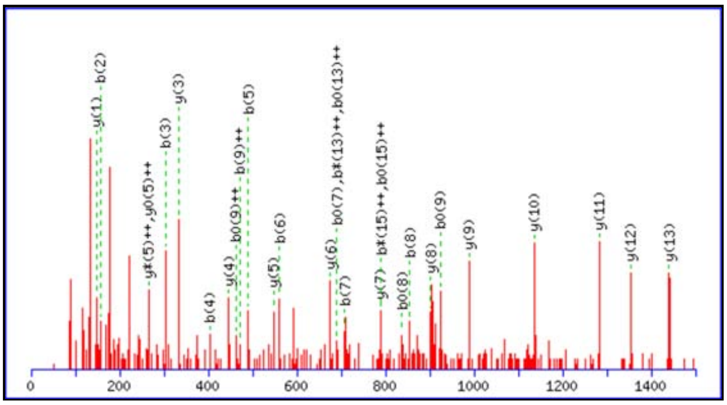

Monoisotopic mass of neutral peptide Mr(calc): 1842.0290
Ions Score: 51 **Expect:** 0.034
Matches (**Bold Red**): 30/158 fragment ions using 79 most intense peaks

#	b	b^{++}	b*	b^{*++}	b^{0}	b^{0++}	Seq.	y	y^{++}	y*	y^{*++}	y^{0}	y^{0++}	#
1	58.0287	29.5180					G							17
2	**157.0971**	79.0522					V	1786.0149	893.5111	1768.9884	884.9978	1768.0043	884.5058	16
3	**304.1656**	152.5864					F	1686.9465	843.9769	1669.9199	835.4636	1668.9359	834.9716	15
4	**403.2340**	202.1206					V	1539.8781	770.4427	1522.8515	761.9294	1521.8675	761.4374	14
5	**490.2660**	245.6366			472.2554	236.6314	S	**1440.8097**	720.9085	1423.7831	712.3952	1422.7991	711.9032	13
6	**561.3031**	281.1552			543.2925	272.1499	A	**1353.7776**	677.3925	1336.7511	668.8792	1335.7671	668.3872	12
7	**708.3715**	354.6894			**690.3610**	345.6841	F	**1282.7405**	641.8739	1265.7140	633.3606	1264.7300	632.8686	11
8	**855.4399**	428.2236			**837.4294**	419.2183	F	**1135.6721**	568.3397	1118.6456	559.8264	1117.6616	559.3344	10
9	942.4720	**471.7396**			**924.4614**	**462.7343**	S	**988.6037**	494.8055	971.5772	486.2922	970.5931	485.8002	9
10	1055.5560	528.2816			1037.5455	519.2764	L	**901.5717**	451.2895	884.5451	442.7762	883.5611	442.2842	8
11	1168.6401	584.8237			1150.6295	575.8184	L	**788.4876**	394.7474	771.4611	386.2342	770.4771	385.7422	7
12	1296.6987	648.8530	1279.6721	640.3397	1278.6881	639.8477	Q	**675.4036**	338.2054	658.3770	329.6921	657.3930	329.2001	6
13	1397.7463	699.3768	1380.7198	**690.8635**	1379.7358	**690.3715**	T	**547.3450**	274.1761	530.3184	**265.6629**	529.3344	**265.1708**	5
14	1510.8304	755.9188	1493.8038	747.4056	1492.8198	746.9136	L	**446.2973**	223.6523	429.2708	215.1390	428.2867	214.6470	4
15	1597.8624	799.4349	1580.8359	**790.9216**	1579.8519	**790.4296**	S	**333.2132**	167.1103	316.1867	158.5970	315.2027	158.1050	3
16	1696.9308	848.9691	1679.9043	840.4558	1678.9203	839.9638	V	246.1812	123.5942	229.1547	115.0810			2
17							K	**147.1128**	74.0600	130.0863	65.5468			1

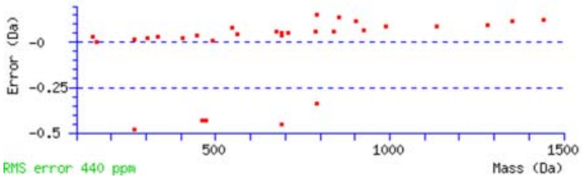

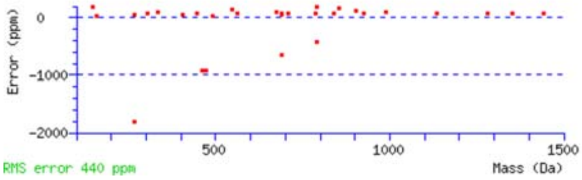

Peptide View

MS/MS Fragmentation of HALIIYDDLSK
Found in gi|6680748|ref|NP_031531.1|, ATP synthase, H+ transporting, mitochondrial F1 complex, alpha subunit, isoform 1 [Mus musculus]

Match to Query 316: 1286.680248 from(644.347400,2+)

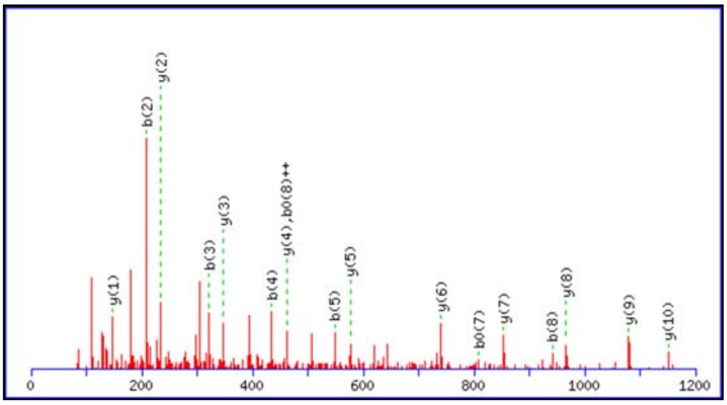

Monoisotopic mass of neutral peptide Mr(calc): 1286.6870
Ions Score: 70 **Expect:** 0.00013
Matches (Bold Red): 17/86 fragment ions using 33 most intense peaks

#	b	b^{++}	b^0	b^{0++}	Seq.	y	y^{++}	y*	y^{*++}	y^0	y^{0++}	#
1	138.0662	69.5367			H							11
2	**209.1033**	105.0553			A	**1150.6354**	575.8213	1133.6088	567.3081	1132.6248	566.8161	10
3	**322.1874**	161.5973			L	**1079.5983**	540.3028	1062.5717	531.7895	1061.5877	531.2975	9
4	**435.2714**	218.1393			I	**966.5142**	483.7607	949.4877	475.2475	948.5037	474.7555	8
5	**548.3555**	274.6814			I	**853.4302**	427.2187	836.4036	418.7054	835.4196	418.2134	7
6	711.4188	356.2130			Y	**740.3461**	370.6767	723.3196	362.1634	722.3355	361.6714	6
7	826.4457	413.7265	**808.4352**	404.7212	D	**577.2828**	289.1450	560.2562	280.6317	559.2722	280.1397	5
8	**941.4727**	471.2400	923.4621	**462.2347**	D	**462.2558**	231.6316	445.2293	223.1183	444.2453	222.6263	4
9	1054.5567	527.7820	1036.5462	518.7767	L	**347.2289**	174.1181	330.2023	165.6048	329.2183	165.1128	3
10	1141.5888	571.2980	1123.5782	562.2927	S	**234.1448**	117.5761	217.1183	109.0628	216.1343	108.5708	2
11					K	**147.1128**	74.0600	130.0863	65.5468			1

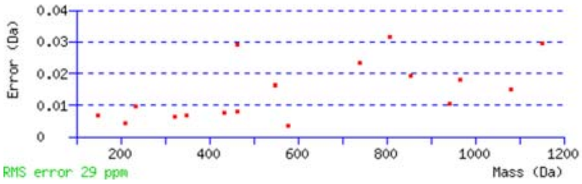

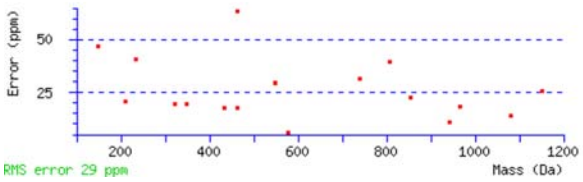

Peptide View

MS/MS Fragmentation of **HLIPAANTGESK**
Found in **gi|31981925|ref|NP_033562.2|**, tyrosine 3-monooxygenase/tryptophan 5-monooxygenase activation protein, epsilon polypeptide [Mus mu

Match to Query 264: 1236.699248 from(619.356900,2+)

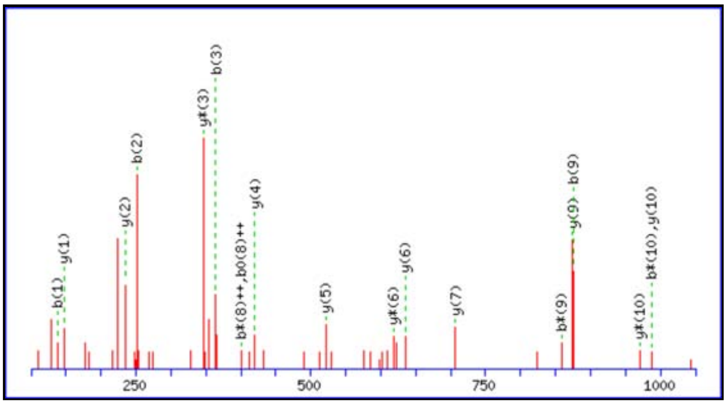

Monoisotopic mass of neutral peptide Mr(calc): 1236.6462
Ions Score: 62 **Expect:** 0.00089
Matches (Bold Red): 19/104 fragment ions using 27 most intense peaks

#	b	b^{++}	b*	b^{*++}	b^0	b^{0++}	Seq.	y	y^{++}	y*	y^{*++}	y^0	y^{0++}	#
1	**138.0662**	69.5367					H							12
2	**251.1502**	126.0788					L	1100.5946	550.8009	1083.5680	542.2877	1082.5840	541.7957	11
3	**364.2343**	182.6208					I	**987.5105**	494.2589	**970.4840**	485.7456	969.5000	485.2536	10
4	461.2871	231.1472					P	**874.4265**	437.7169	857.3999	429.2036	856.4159	428.7116	9
5	532.3242	266.6657					A	777.3737	389.1905	760.3472	380.6772	759.3631	380.1852	8
6	603.3613	302.1843					A	**706.3366**	353.6719	689.3101	345.1587	688.3260	344.6667	7
7	717.4042	359.2057	700.3777	350.6925			N	**635.2995**	318.1534	**618.2729**	309.6401	617.2889	309.1481	6
8	818.4519	409.7296	801.4253	**401.2163**	800.4413	**400.7243**	T	**521.2566**	261.1319	504.2300	252.6186	503.2460	252.1266	5
9	**875.4734**	438.2403	**858.4468**	429.7270	857.4628	429.2350	G	**420.2089**	210.6081	403.1823	202.0948	402.1983	201.6028	4
10	1004.5159	502.7616	**987.4894**	494.2483	986.5054	493.7563	E	363.1874	182.0973	**346.1609**	173.5841	345.1769	173.0921	3
11	1091.5480	546.2776	1074.5214	537.7644	1073.5374	537.2723	S	**234.1448**	117.5761	217.1183	109.0628	216.1343	108.5708	2
12							K	**147.1128**	74.0600	130.0863	65.5468			1

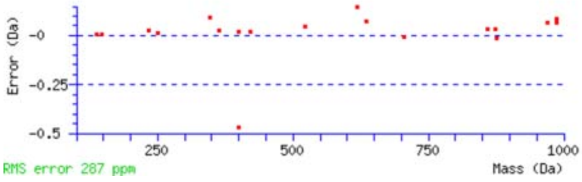

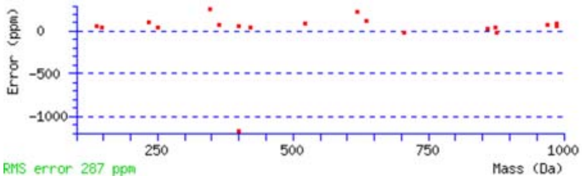

Peptide View

MS/MS Fragmentation of **HPTQLANSGLK**
Found in **gi|6753856|ref|NP_034336.1|**, fibroblast growth factor receptor 1 [Mus musculus]

Match to Query 220: 1164.638848 from(583.326700,2+)

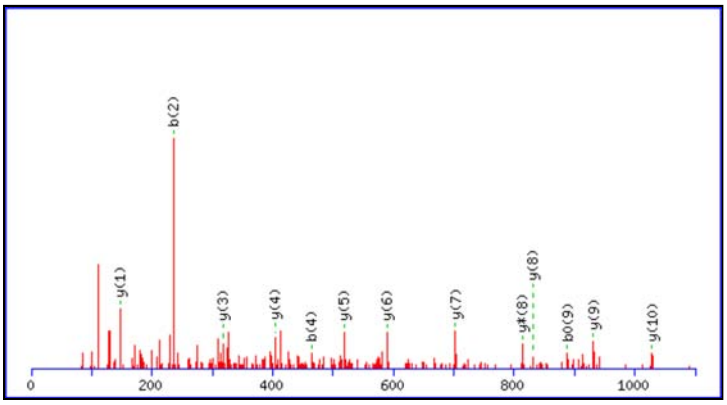

Monoisotopic mass of neutral peptide Mr(calc): 1164.6251
Ions Score: 48 **Expect:** 0.021
Matches (**Bold Red**): 13/104 fragment ions using 32 most intense peaks

#	b	b^{++}	b*	b^{*++}	b^{0}	b^{0++}	Seq.	y	y^{++}	y*	y^{*++}	y^{0}	y^{0++}	#
1	138.0662	69.5367					H							11
2	**235.1189**	118.0631					P	**1028.5735**	514.7904	1011.5469	506.2771	1010.5629	505.7851	10
3	336.1666	168.5870			318.1561	159.5817	T	**931.5207**	466.2640	914.4942	457.7507	913.5101	457.2587	9
4	**464.2252**	232.6162	447.1987	224.1030	446.2146	223.6110	Q	**830.4730**	415.7402	**813.4465**	407.2269	812.4625	406.7349	8
5	577.3093	289.1583	560.2827	280.6450	559.2987	280.1530	L	**702.4145**	351.7109	685.3879	343.1976	684.4039	342.7056	7
6	648.3464	324.6768	631.3198	316.1636	630.3358	315.6715	A	**589.3304**	295.1688	572.3038	286.6556	571.3198	286.1636	6
7	762.3893	381.6983	745.3628	373.1850	744.3787	372.6930	N	**518.2933**	259.6503	501.2667	251.1370	500.2827	250.6450	5
8	849.4213	425.2143	832.3948	416.7010	831.4108	416.2090	S	**404.2504**	202.6288	387.2238	194.1155	386.2398	193.6235	4
9	906.4428	453.7250	889.4162	445.2118	**888.4322**	444.7198	G	**317.2183**	159.1128	300.1918	150.5995			3
10	1019.5269	510.2671	1002.5003	501.7538	1001.5163	501.2618	L	260.1969	130.6021	243.1703	122.0888			2
11							K	**147.1128**	74.0600	130.0863	65.5468			1

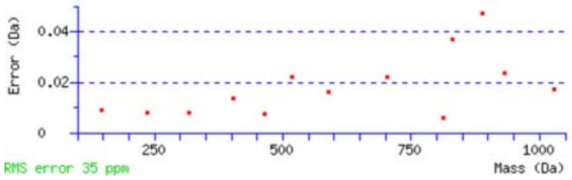

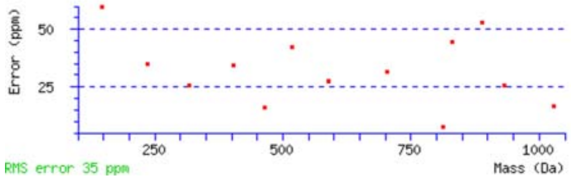

Peptide View

MS/MS Fragmentation of IKEEYEVAEMGAPHGSASVR
Found in gi|21539593|ref|NP_079654.1|, interferon induced transmembrane protein 3 [Mus musculus]

Match to Query 687: 2169.104472 from(724.042100,3+)

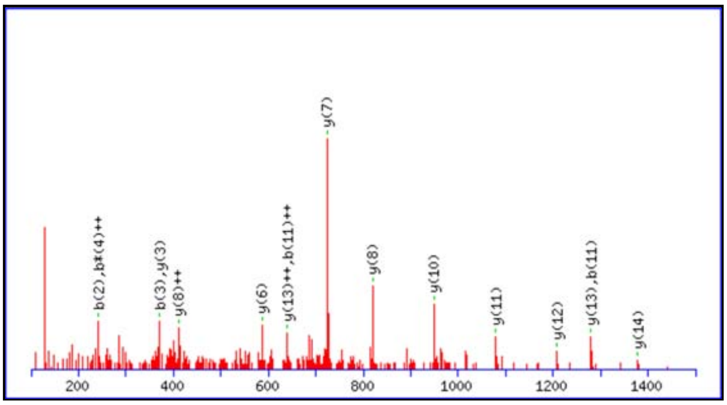

Monoisotopic mass of neutral peptide Mr(calc): 2169.0652
Variable modifications:
R20 : Arginine-13C615N4 (R-full)
Ions Score: 75 Expect: 0.00019
Matches (**Bold Red**): 16/218 fragment ions using 17 most intense peaks

#	b	b^{++}	b*	b^{*++}	b^{0}	b^{0++}	Seq.	y	y^{++}	y*	y^{*++}	y^{0}	y^{0++}	#
1	114.0913	57.5493					I							20
2	**242.1863**	121.5968	225.1597	113.0835			K	2056.9884	1028.9979	2039.9619	1020.4846	2038.9779	1019.9926	19
3	**371.2289**	186.1181	354.2023	177.6048	353.2183	177.1128	E	1928.8935	964.9504	1911.8669	956.4371	1910.8829	955.9451	18
4	500.2715	250.6394	483.2449	**242.1261**	482.2609	241.6341	E	1799.8509	900.4291	1782.8243	891.9158	1781.8403	891.4238	17
5	663.3348	332.1710	646.3083	323.6578	645.3242	323.1658	Y	1670.8083	835.9078	1653.7817	827.3945	1652.7977	826.9025	16
6	792.3774	396.6923	775.3508	388.1791	774.3668	387.6871	E	1507.7450	754.3761	1490.7184	745.8628	1489.7344	745.3708	15
7	891.4458	446.2265	874.4193	437.7133	873.4352	437.2213	V	**1378.7024**	689.8548	1361.6758	681.3416	1360.6918	680.8495	14
8	962.4829	481.7451	945.4564	473.2318	944.4724	472.7398	A	**1279.6340**	**640.3206**	1262.6074	631.8073	1261.6234	631.3153	13
9	1091.5255	546.2664	1074.4990	537.7531	1073.5149	537.2611	E	**1208.5969**	604.8021	1191.5703	596.2888	1190.5863	595.7968	12
10	1222.5660	611.7866	1205.5394	603.2734	1204.5554	602.7813	M	**1079.5543**	540.2808	1062.5277	531.7675	1061.5437	531.2755	11
11	**1279.5874**	**640.2974**	1262.5609	631.7841	1261.5769	631.2921	G	**948.5138**	474.7605	931.4872	466.2473	930.5032	465.7552	10
12	1350.6246	675.8159	1333.5980	667.3026	1332.6140	666.8106	A	891.4923	446.2498	874.4658	437.7365	873.4818	437.2445	9
13	1447.6773	724.3423	1430.6508	715.8290	1429.6668	715.3370	P	**820.4552**	**410.7312**	803.4287	402.2180	802.4447	401.7260	8
14	1584.7362	792.8718	1567.7097	784.3585	1566.7257	783.8665	H	**723.4025**	362.2049	706.3759	353.6916	705.3919	353.1996	7
15	1641.7577	821.3825	1624.7311	812.8692	1623.7471	812.3772	G	**586.3435**	293.6754	569.3170	285.1621	568.3330	284.6701	6
16	1728.7897	864.8985	1711.7632	856.3852	1710.7792	855.8932	S	529.3221	265.1647	512.2955	256.6514	511.3115	256.1594	5
17	1799.8268	900.4171	1782.8003	891.9038	1781.8163	891.4118	A	442.2901	221.6487	425.2635	213.1354	424.2795	212.6434	4
18	1886.8589	943.9331	1869.8323	935.4198	1868.8483	934.9278	S	**371.2529**	186.1301	354.2264	177.6168	353.2424	177.1248	3
19	1985.9273	993.4673	1968.9007	984.9540	1967.9167	984.4620	V	284.2209	142.6141	267.1944	134.1008			2
20							R	185.1525	93.0799	168.1260	84.5666			1

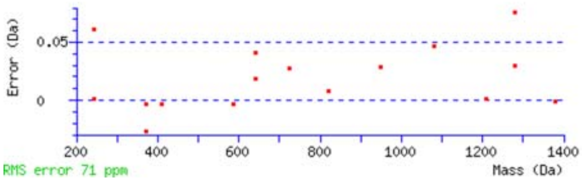

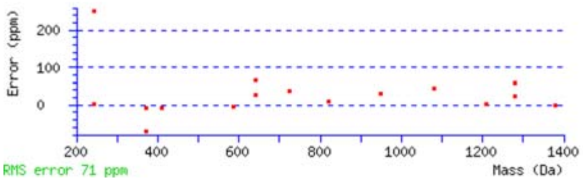

Peptide View

MS/MS Fragmentation of **ILADLEDYLNELWEDKEGK**
Found in **gi|22203755|ref|NP_666312.1|**, eukaryotic translation initiation factor 3, subunit 8 [Mus musculus]

Match to Query 1195: 2292.253272 from(765.091700,3+)

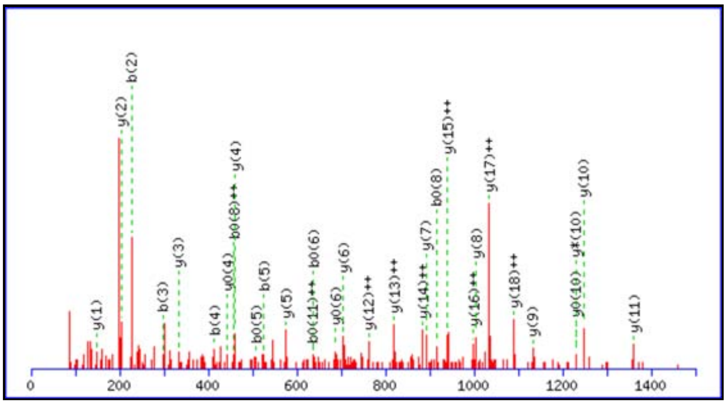

Monoisotopic mass of neutral peptide Mr(calc): 2292.1161
Ions Score: 51 **Expect:** 0.049
Matches (**Bold Red**): 31/188 fragment ions using 94 most intense peaks

#	b	b^{++}	b*	b^{*++}	b^{0}	b^{0++}	Seq.	y	y^{++}	y*	y^{*++}	y^{0}	y^{0++}	#
1	114.0913	57.5493					I							19
2	**227.1754**	114.0913					L	2180.0393	**1090.5233**	2163.0128	1082.0100	2162.0288	1081.5180	18
3	**298.2125**	149.6099					A	2066.9553	**1033.9813**	2049.9287	1025.4680	2048.9447	1024.9760	17
4	**413.2394**	207.1234			395.2289	198.1181	D	1995.9182	**998.4627**	1978.8916	989.9494	1977.9076	989.4574	16
5	**526.3235**	263.6654			**508.3129**	254.6601	L	1880.8912	**940.9492**	1863.8647	932.4360	1862.8806	931.9440	15
6	655.3661	328.1867			**637.3555**	319.1814	E	1767.8072	**884.4072**	1750.7806	875.8939	1749.7966	875.4019	14
7	770.3930	385.7002			752.3825	376.6949	D	1638.7646	**819.8859**	1621.7380	811.3726	1620.7540	810.8806	13
8	933.4564	467.2318			**915.4458**	**458.2265**	Y	1523.7376	**762.3724**	1506.7111	753.8592	1505.7271	753.3672	12
9	1046.5404	523.7739			1028.5299	514.7686	L	**1360.6743**	680.8408	1343.6477	672.3275	1342.6637	671.8355	11
10	1160.5834	580.7953	1143.5568	572.2820	1142.5728	571.7900	N	**1247.5902**	624.2988	**1230.5637**	615.7855	**1229.5797**	615.2935	10
11	1289.6259	645.3166	1272.5994	636.8033	1271.6154	**636.3113**	E	**1133.5473**	567.2773	1116.5208	558.7640	1115.5367	558.2720	9
12	1402.7100	701.8586	1385.6835	693.3454	1384.6994	692.8534	L	**1004.5047**	502.7560	987.4782	494.2427	986.4941	493.7507	8
13	1588.7893	794.8983	1571.7628	786.3850	1570.7788	785.8930	W	**891.4207**	446.2140	874.3941	437.7007	873.4101	437.2087	7
14	1717.8319	859.4196	1700.8054	850.9063	1699.8213	850.4143	E	**705.3413**	353.1743	688.3148	344.6610	**687.3308**	344.1690	6
15	1832.8588	916.9331	1815.8323	908.4198	1814.8483	907.9278	D	**576.2988**	288.6530	559.2722	280.1397	558.2882	279.6477	5
16	1960.9538	980.9805	1943.9273	972.4673	1942.9432	971.9753	K	**461.2718**	231.1395	444.2453	222.6263	**443.2612**	222.1343	4
17	2089.9964	1045.5018	2072.9698	1036.9886	2071.9858	1036.4966	E	**333.1769**	167.0921	316.1503	158.5788	315.1663	158.0868	3
18	2147.0179	1074.0126	2129.9913	1065.4993	2129.0073	1065.0073	G	**204.1343**	102.5708	187.1077	94.0575			2
19							K	**147.1128**	74.0600	130.0863	65.5468			1

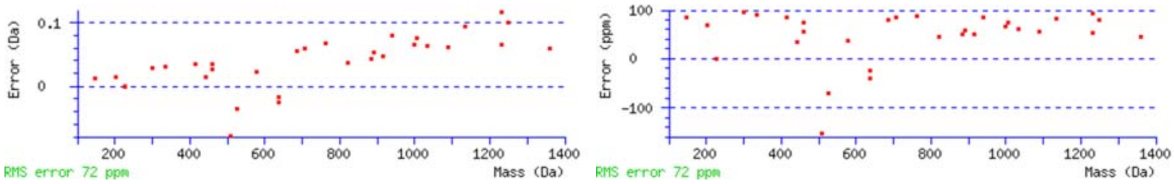

Peptide View

MS/MS Fragmentation of **ILTFDQLALESPK**
Found in **gi|83699424|ref|NP_033103.2|**, ribosomal protein L18 [Mus musculus]

Match to Query 308: 1473.879648 from(737.947100,2+)

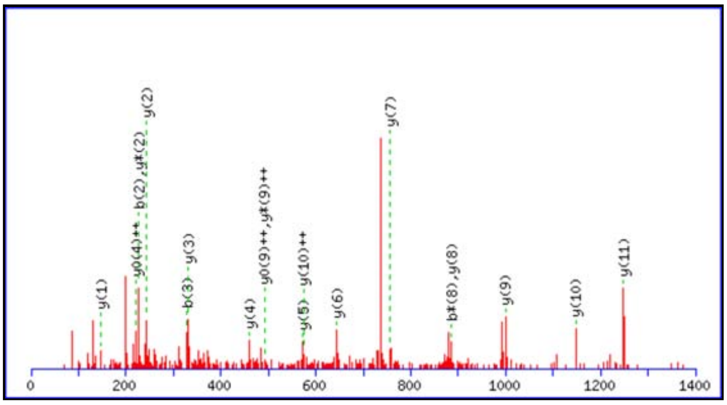

Monoisotopic mass of neutral peptide Mr(calc): 1473.8079
Ions Score: 46 **Expect:** 0.048
Matches (**Bold Red**): 19/126 fragment ions using 63 most intense peaks

#	b	b^{++}	b*	b^{*++}	b^{0}	b^{0++}	Seq.	y	y^{++}	y*	y^{*++}	y^{0}	y^{0++}	#
1	114.0913	57.5493					I							13
2	**227.1754**	114.0913					L	1361.7311	681.3692	1344.7045	672.8559	1343.7205	672.3639	12
3	**328.2231**	164.6152			310.2125	155.6099	T	**1248.6470**	624.8271	1231.6205	616.3139	1230.6365	615.8219	11
4	475.2915	238.1494			457.2809	229.1441	F	**1147.5993**	**574.3033**	1130.5728	565.7900	1129.5888	565.2980	10
5	590.3184	295.6629			572.3079	286.6576	D	**1000.5309**	500.7691	983.5044	**492.2558**	982.5204	**491.7638**	9
6	718.3770	359.6921	701.3505	351.1789	700.3664	350.6869	Q	**885.5040**	443.2556	868.4774	434.7424	867.4934	434.2504	8
7	831.4611	416.2342	814.4345	407.7209	813.4505	407.2289	L	**757.4454**	379.2263	740.4189	370.7131	739.4348	370.2211	7
8	902.4982	451.7527	**885.4716**	443.2395	884.4876	442.7474	A	**644.3614**	322.6843	627.3348	314.1710	626.3508	313.6790	6
9	1015.5822	508.2948	998.5557	499.7815	997.5717	499.2895	L	**573.3242**	287.1658	556.2977	278.6525	555.3137	278.1605	5
10	1144.6248	572.8161	1127.5983	564.3028	1126.6143	563.8108	E	**460.2402**	230.6237	443.2136	222.1105	442.2296	**221.6184**	4
11	1231.6569	616.3321	1214.6303	607.8188	1213.6463	607.3268	S	**331.1976**	166.1024	314.1710	157.5892	313.1870	157.0972	3
12	1328.7096	664.8584	1311.6831	656.3452	1310.6991	655.8532	P	**244.1656**	122.5864	**227.1390**	114.0731			2
13							K	**147.1128**	74.0600	130.0863	65.5468			1

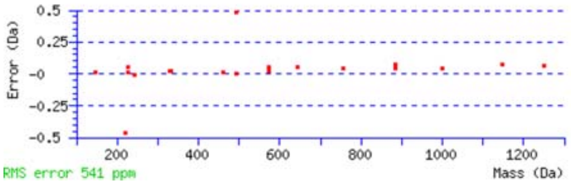

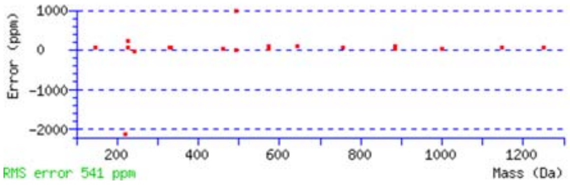

Peptide View

MS/MS Fragmentation of **IQDALSTVLQYAEDVLSGK**
Found in **gi|21313620|ref|NP_079620.1|**, eukaryotic translation initiation factor 3, subunit 5 (epsilon) [Mus musculus]

Match to Query 1120: 2049.219648 from(1025.617100,2+)

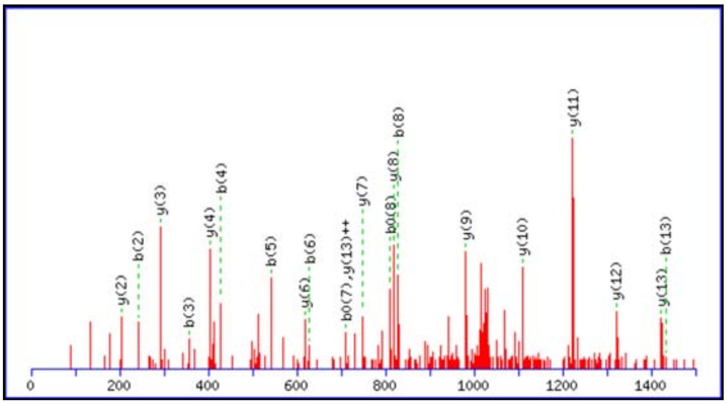

Monoisotopic mass of neutral peptide Mr(calc): 2049.0629
Ions Score: 81 **Expect:** 4e-005
Matches (**Bold Red**): 21/206 fragment ions using 41 most intense peaks

#	b	b^{++}	b*	b^{*++}	b^{0}	b^{0++}	Seq.	y	y^{++}	y*	y^{*++}	y^{0}	y^{0++}	#
1	114.0913	57.5493					I							19
2	**242.1499**	121.5786	225.1234	113.0653			Q	1936.9862	968.9967	1919.9596	960.4835	1918.9756	959.9914	18
3	**357.1769**	179.0921	340.1503	170.5788	339.1663	170.0868	D	1808.9276	904.9674	1791.9011	896.4542	1790.9170	895.9622	17
4	**428.2140**	214.6106	411.1874	206.0973	410.2034	205.6053	A	1693.9007	847.4540	1676.8741	838.9407	1675.8901	838.4487	16
5	**541.2980**	271.1527	524.2715	262.6394	523.2875	262.1474	L	1622.8636	811.9354	1605.8370	803.4221	1604.8530	802.9301	15
6	**628.3301**	314.6687	611.3035	306.1554	610.3195	305.6634	S	1509.7795	755.3934	1492.7529	746.8801	1491.7689	746.3881	14
7	729.3777	365.1925	712.3512	356.6792	**711.3672**	356.1872	T	**1422.7475**	**711.8774**	1405.7209	703.3641	1404.7369	702.8721	13
8	**828.4461**	414.7267	811.4196	406.2134	**810.4356**	405.7214	V	**1321.6998**	661.3535	1304.6732	652.8403	1303.6892	652.3482	12
9	941.5302	471.2687	924.5037	462.7555	923.5196	462.2635	L	**1222.6314**	611.8193	1205.6048	603.3060	1204.6208	602.8140	11
10	1069.5888	535.2980	1052.5622	526.7848	1051.5782	526.2927	Q	**1109.5473**	555.2773	1092.5208	546.7640	1091.5367	546.2720	10
11	1232.6521	616.8297	1215.6256	608.3164	1214.6416	607.8244	Y	**981.4887**	491.2480	964.4622	482.7347	963.4782	482.2427	9
12	1303.6892	652.3483	1286.6627	643.8350	1285.6787	643.3430	A	**818.4254**	409.7163	801.3989	401.2031	800.4148	400.7111	8
13	**1432.7318**	716.8695	1415.7053	708.3563	1414.7213	707.8643	E	**747.3883**	374.1978	730.3617	365.6845	729.3777	365.1925	7
14	1547.7588	774.3830	1530.7322	765.8697	1529.7482	765.3777	D	**618.3457**	309.6765	601.3192	301.1632	600.3351	300.6712	6
15	1646.8272	823.9172	1629.8006	815.4039	1628.8166	814.9119	V	503.3188	252.1630	486.2922	243.6497	485.3082	243.1577	5
16	1759.9112	880.4593	1742.8847	871.9460	1741.9007	871.4540	L	**404.2504**	202.6288	387.2238	194.1155	386.2398	193.6235	4
17	1846.9433	923.9753	1829.9167	915.4620	1828.9327	914.9700	S	**291.1663**	146.0868	274.1397	137.5735	273.1557	137.0815	3
18	1903.9647	952.4860	1886.9382	943.9727	1885.9542	943.4807	G	**204.1343**	102.5708	187.1077	94.0575			2
19							K	147.1128	74.0600	130.0863	65.5468			1

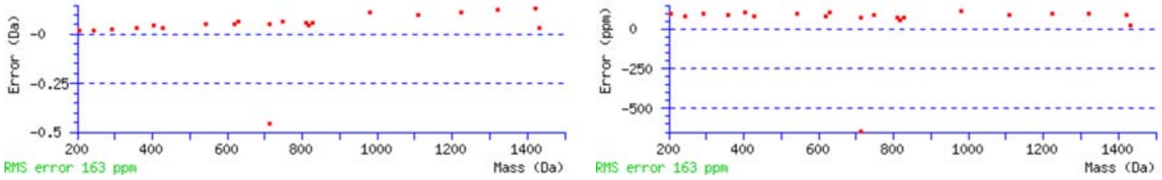

Peptide View

MS/MS Fragmentation of IQSLVGLSWIDWLK
Found in gi|29789235|ref|NP_083370.1|, phosphatidylinositol 3-kinase, catalytic, beta polypeptide [Mus musculus]

Match to Query 553: 1656.942448 from(829.478500,2+)

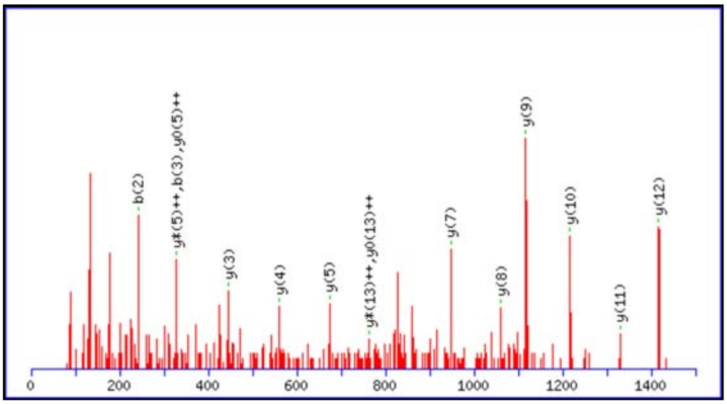

Monoisotopic mass of neutral peptide Mr(calc): 1656.9239
Ions Score: 81 **Expect:** 1.9e-005
Matches (**Bold Red**): 15/144 fragment ions using 21 most intense peaks

#	b	b^{++}	b*	b^{*++}	b^0	b^{0++}	Seq.	y	y^{++}	y*	y^{*++}	y^0	y^{0++}	#
1	114.0913	57.5493					I							14
2	**242.1499**	121.5786	225.1234	113.0653			Q	1544.8471	772.9272	1527.8206	**764.4139**	1526.8365	**763.9219**	13
3	**329.1819**	165.0946	312.1554	156.5813	311.1714	156.0893	S	**1416.7885**	708.8979	1399.7620	700.3846	1398.7780	699.8926	12
4	442.2660	221.6366	425.2395	213.1234	424.2554	212.6314	L	**1329.7565**	665.3819	1312.7300	656.8686	1311.7459	656.3766	11
5	541.3344	271.1708	524.3079	262.6576	523.3239	262.1656	V	**1216.6724**	608.8399	1199.6459	600.3266	1198.6619	599.8346	10
6	598.3559	299.6816	581.3293	291.1683	580.3453	290.6763	G	**1117.6040**	559.3057	1100.5775	550.7924	1099.5935	550.3004	9
7	711.4399	356.2236	694.4134	347.7103	693.4294	347.2183	L	**1060.5826**	530.7949	1043.5560	522.2816	1042.5720	521.7896	8
8	798.4720	399.7396	781.4454	391.2263	780.4614	390.7343	S	**947.4985**	474.2529	930.4720	465.7396	929.4879	465.2476	7
9	984.5513	492.7793	967.5247	484.2660	966.5407	483.7740	W	860.4665	430.7369	843.4399	422.2236	842.4559	421.7316	6
10	1097.6353	549.3213	1080.6088	540.8080	1079.6248	540.3160	I	**674.3872**	337.6972	657.3606	**329.1839**	656.3766	**328.6919**	5
11	1212.6623	606.8348	1195.6357	598.3215	1194.6517	597.8295	D	**561.3031**	281.1552	544.2766	272.6419	543.2925	272.1499	4
12	1398.7416	699.8744	1381.7150	691.3612	1380.7310	690.8691	W	**446.2762**	223.6417	429.2496	215.1284			3
13	1511.8256	756.4165	1494.7991	747.9032	1493.8151	747.4112	L	260.1969	130.6021	243.1703	122.0888			2
14							K	147.1128	74.0600	130.0863	65.5468			1

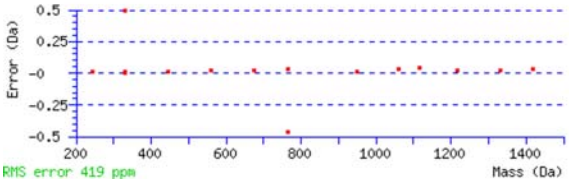

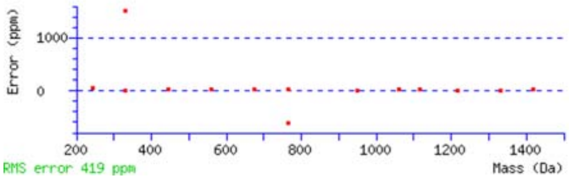

Peptide View

MS/MS Fragmentation of ISNNNNGLTEVLK
Found in gi|27370424|ref|NP_766512.1|, hypothetical protein LOC244895 [Mus musculus]

Match to Query 270: 1414.802048 from(708.408300,2+)

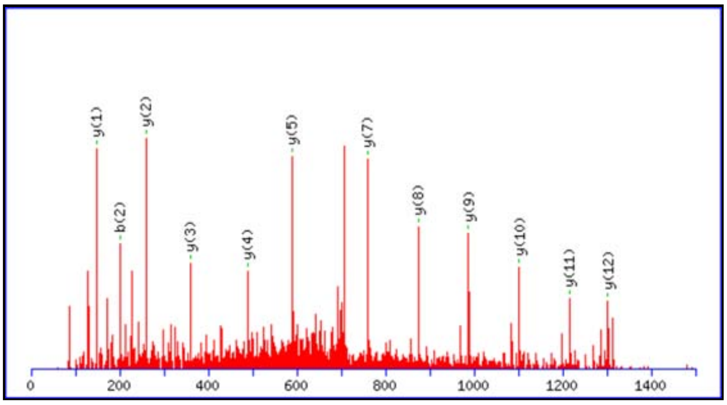

Monoisotopic mass of neutral peptide Mr(calc): 1414.7415
Ions Score: 110 **Expect:** 1.8e-008
Matches (**Bold Red**): 12/132 fragment ions using 24 most intense peaks

#	b	b^{++}	b*	b^{*++}	b^{0}	b^{0++}	Seq.	y	y^{++}	y*	y^{*++}	y^{0}	y^{0++}	#
1	114.0913	57.5493					I							13
2	**201.1234**	101.0653			183.1128	92.0600	S	**1302.6648**	651.8360	1285.6383	643.3228	1284.6542	642.8308	12
3	315.1663	158.0868	298.1397	149.5735	297.1557	149.0815	N	**1215.6328**	608.3200	1198.6062	599.8068	1197.6222	599.3147	11
4	429.2092	215.1083	412.1827	206.5950	411.1987	206.1030	N	**1101.5899**	551.2986	1084.5633	542.7853	1083.5793	542.2933	10
5	543.2522	272.1297	526.2256	263.6164	525.2416	263.1244	N	**987.5469**	494.2771	970.5204	485.7638	969.5364	485.2718	9
6	657.2951	329.1512	640.2685	320.6379	639.2845	320.1459	N	**873.5040**	437.2556	856.4774	428.7424	855.4934	428.2504	8
7	714.3165	357.6619	697.2900	349.1486	696.3060	348.6566	G	**759.4611**	380.2342	742.4345	371.7209	741.4505	371.2289	7
8	827.4006	414.2039	810.3741	405.6907	809.3900	405.1987	L	702.4396	351.7234	685.4131	343.2102	684.4290	342.7182	6
9	928.4483	464.7278	911.4217	456.2145	910.4377	455.7225	T	**589.3555**	295.1814	572.3290	286.6681	571.3450	286.1761	5
10	1057.4909	529.2491	1040.4643	520.7358	1039.4803	520.2438	E	**488.3079**	244.6576	471.2813	236.1443	470.2973	235.6523	4
11	1156.5593	578.7833	1139.5327	570.2700	1138.5487	569.7780	V	**359.2653**	180.1363	342.2387	171.6230			3
12	1269.6433	635.3253	1252.6168	626.8120	1251.6328	626.3200	L	**260.1969**	130.6021	243.1703	122.0888			2
13							K	**147.1128**	74.0600	130.0863	65.5468			1

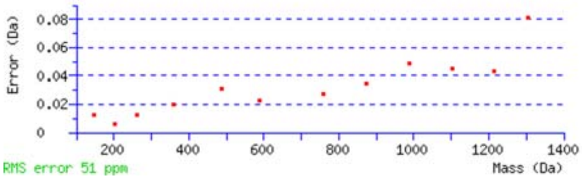

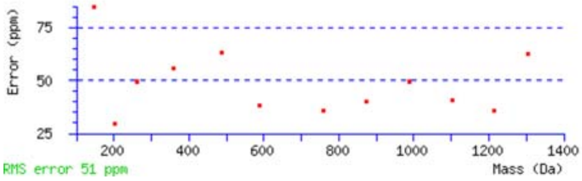

Peptide View

MS/MS Fragmentation of **KLEAAATALATK**
Found in **gi|30794450|ref|NP_077174.1|**, ribosomal protein L4 [Mus musculus]

Match to Query 213: 1186.750848 from(594.382700,2+)

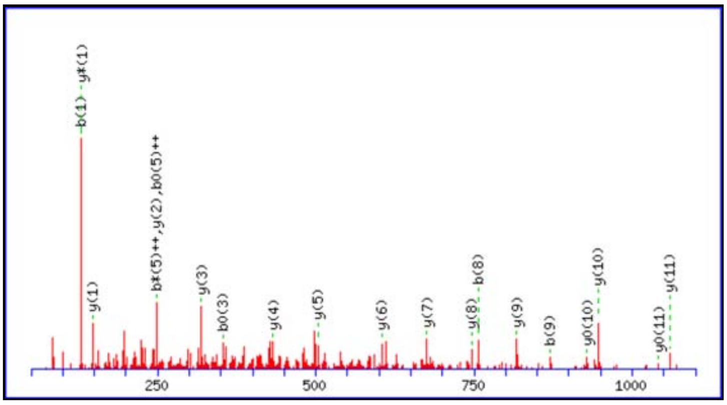

Monoisotopic mass of neutral peptide Mr(calc): 1186.6921
Ions Score: 92 **Expect:** 8.8e-007
Matches (**Bold Red**): 21/126 fragment ions using 34 most intense peaks

#	b	b^{++}	b*	b^{*++}	b^0	b^{0++}	Seq.	y	y^{++}	y*	y^{*++}	y^0	y^{0++}	#
1	**129.1022**	65.0548	112.0757	56.5415			K							12
2	242.1863	121.5968	225.1597	113.0835			L	**1059.6044**	530.3058	1042.5779	521.7926	**1041.5939**	521.3006	11
3	371.2289	186.1181	354.2023	177.6048	**353.2183**	177.1128	E	**946.5204**	473.7638	929.4938	465.2505	**928.5098**	464.7585	10
4	442.2660	221.6366	425.2394	213.1234	424.2554	212.6314	A	**817.4778**	409.2425	800.4512	400.7292	799.4672	400.2372	9
5	513.3031	257.1552	496.2766	**248.6419**	495.2925	**248.1499**	A	**746.4407**	373.7240	729.4141	365.2107	728.4301	364.7187	8
6	584.3402	292.6737	567.3137	284.1605	566.3297	283.6685	A	**675.4036**	338.2054	658.3770	329.6921	657.3930	329.2001	7
7	685.3879	343.1976	668.3613	334.6843	667.3773	334.1923	T	**604.3664**	302.6869	587.3399	294.1736	586.3559	293.6816	6
8	**756.4250**	378.7161	739.3985	370.2029	738.4144	369.7109	A	**503.3188**	252.1630	486.2922	243.6497	485.3082	243.1577	5
9	**869.5091**	435.2582	852.4825	426.7449	851.4985	426.2529	L	**432.2817**	216.6445	415.2551	208.1312	414.2711	207.6392	4
10	940.5462	470.7767	923.5196	462.2635	922.5356	461.7714	A	**319.1976**	160.1024	302.1710	151.5892	301.1870	151.0972	3
11	**1041.5939**	521.3006	1024.5673	512.7873	1023.5833	512.2953	T	**248.1605**	124.5839	231.1339	116.0706	230.1499	115.5786	2
12							K	**147.1128**	74.0600	**130.0863**	65.5468			1

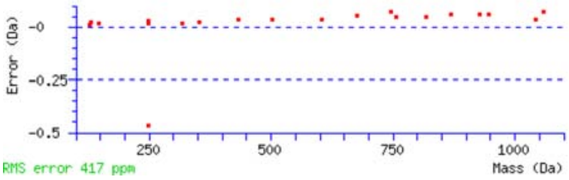

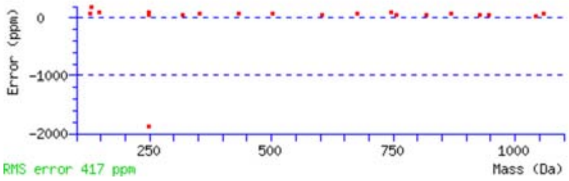

Peptide View

MS/MS Fragmentation of **KLQEEELLATHVDMR**
Found in **gi|71043975|ref|NP_080478.2|**, Ymer protein isoform 1 [Mus musculus]

Match to Query 595: 1810.970772 from(604.664200,3+)

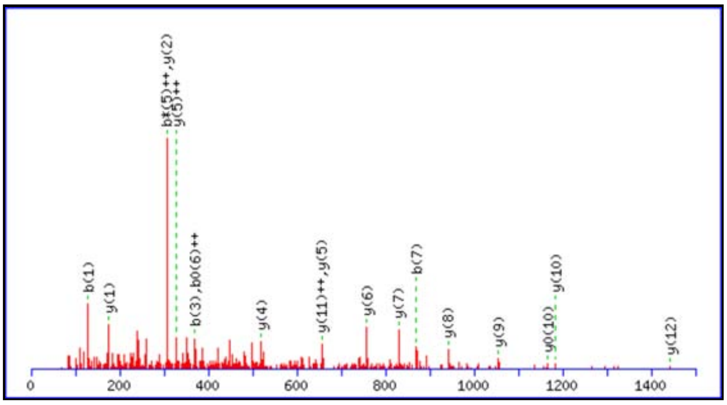

Monoisotopic mass of neutral peptide Mr(calc): 1810.9247
Ions Score: 84 **Expect:** 1.1e-005
Matches (Bold Red): 18/158 fragment ions using 22 most intense peaks

#	b	b^{++}	b*	b^{*++}	b^0	b^{0++}	Seq.	y	y^{++}	y*	y^{*++}	y^0	y^{0++}	#
1	**129.1022**	65.0548	112.0757	56.5415			K							15
2	242.1863	121.5968	225.1597	113.0835			L	1683.8370	842.4221	1666.8105	833.9089	1665.8264	833.4169	14
3	**370.2449**	185.6261	353.2183	177.1128			Q	1570.7530	785.8801	1553.7264	777.3668	1552.7424	776.8748	13
4	499.2875	250.1474	482.2609	241.6341	481.2769	241.1421	E	**1442.6944**	721.8508	1425.6678	713.3375	1424.6838	712.8455	12
5	628.3301	314.6687	611.3035	**306.1554**	610.3195	305.6634	E	1313.6518	**657.3295**	1296.6252	648.8163	1295.6412	648.3242	11
6	757.3726	379.1900	740.3461	370.6767	739.3621	**370.1847**	E	**1184.6092**	592.8082	1167.5826	584.2950	**1166.5986**	583.8030	10
7	**870.4567**	435.7320	853.4302	427.2187	852.4461	426.7267	L	**1055.5666**	528.2869	1038.5401	519.7737	1037.5560	519.2817	9
8	983.5408	492.2740	966.5142	483.7607	965.5302	483.2687	L	**942.4825**	471.7449	925.4560	463.2316	924.4720	462.7396	8
9	1054.5779	527.7926	1037.5513	519.2793	1036.5673	518.7873	A	**829.3985**	415.2029	812.3719	406.6896	811.3879	406.1976	7
10	1155.6256	578.3164	1138.5990	569.8031	1137.6150	569.3111	T	**758.3614**	379.6843	741.3348	371.1710	740.3508	370.6790	6
11	1292.6845	646.8459	1275.6579	638.3326	1274.6739	637.8406	H	**657.3137**	**329.1605**	640.2871	320.6472	639.3031	320.1552	5
12	1391.7529	696.3801	1374.7263	687.8668	1373.7423	687.3748	V	**520.2548**	260.6310	503.2282	252.1178	502.2442	251.6257	4
13	1506.7798	753.8935	1489.7533	745.3803	1488.7693	744.8883	D	421.1864	211.0968	404.1598	202.5835	403.1758	202.0915	3
14	1637.8203	819.4138	1620.7937	810.9005	1619.8097	810.4085	M	**306.1594**	153.5834	289.1329	145.0701			2
15							R	**175.1190**	88.0631	158.0924	79.5498			1

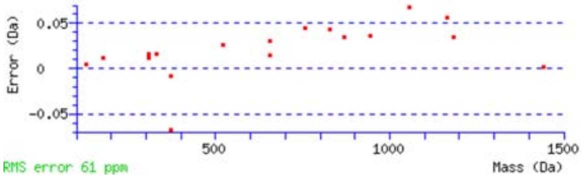

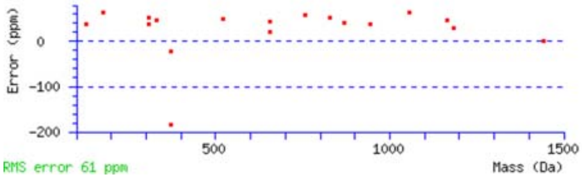

Peptide View

MS/MS Fragmentation of KLWMSLGDSWVK
Found in gi|75677397|ref|NP_031971.2|, epidermal growth factor receptor pathway substrate 8 [Mus musculus]

Match to Query 445: 1448.758248 from(725.386400,2+)

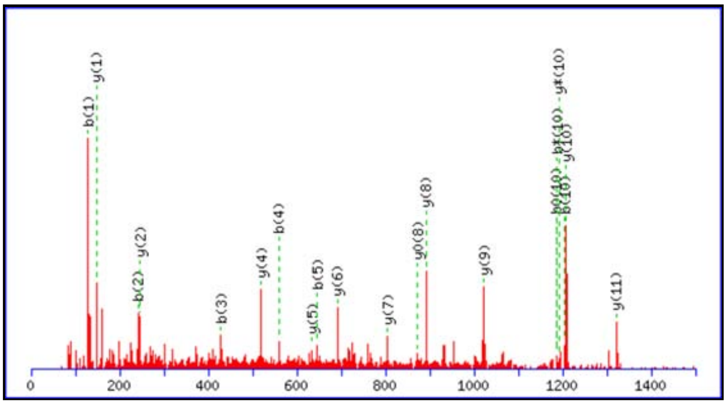

Monoisotopic mass of neutral peptide Mr(calc): 1448.7486
Ions Score: 66 **Expect:** 0.00042
Matches (**Bold Red**): 20/118 fragment ions using 52 most intense peaks

#	b	b^{++}	b*	b^{*++}	b^{0}	b^{0++}	Seq.	y	y^{++}	y*	y^{*++}	y^{0}	y^{0++}	#
1	**129.1022**	65.0548	112.0757	56.5415			K							12
2	**242.1863**	121.5968	225.1597	113.0835			L	**1321.6609**	661.3341	1304.6343	652.8208	1303.6503	652.3288	11
3	**428.2656**	214.6364	411.2391	206.1232			W	**1208.5768**	604.7921	**1191.5503**	596.2788	1190.5663	595.7868	10
4	**559.3061**	280.1567	542.2795	271.6434			M	**1022.4975**	511.7524	1005.4710	503.2391	1004.4870	502.7471	9
5	**646.3381**	323.6727	629.3116	315.1594	628.3276	314.6674	S	**891.4570**	446.2322	874.4305	437.7189	**873.4465**	437.2269	8
6	759.4222	380.2147	742.3956	371.7015	741.4116	371.2094	L	**804.4250**	402.7161	787.3985	394.2029	786.4144	393.7109	7
7	816.4436	408.7255	799.4171	400.2122	798.4331	399.7202	G	**691.3410**	346.1741	674.3144	337.6608	673.3304	337.1688	6
8	931.4706	466.2389	914.4440	457.7257	913.4600	457.2336	D	**634.3195**	317.6634	617.2929	309.1501	616.3089	308.6581	5
9	1018.5026	509.7549	1001.4761	501.2417	1000.4920	500.7497	S	**519.2926**	260.1499	502.2660	251.6366	501.2820	251.1446	4
10	**1204.5819**	602.7946	**1187.5554**	594.2813	**1186.5714**	593.7893	W	432.2605	216.6339	415.2340	208.1206			3
11	1303.6503	652.3288	1286.6238	643.8155	1285.6398	643.3235	V	**246.1812**	123.5942	229.1547	115.0810			2
12							K	**147.1128**	74.0600	130.0863	65.5468			1

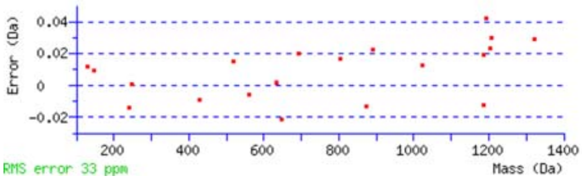

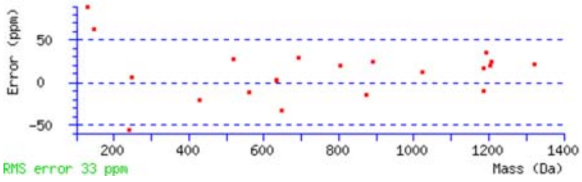

Peptide View

MS/MS Fragmentation of **KPLFGEIGHTIMNLLVDLR**
Found in **gi|27734996|ref|NP_775568.1|**, zinc finger, FYVE domain containing 16 [Mus musculus]

Match to Query 534: 2171.328072 from(724.783300,3+)

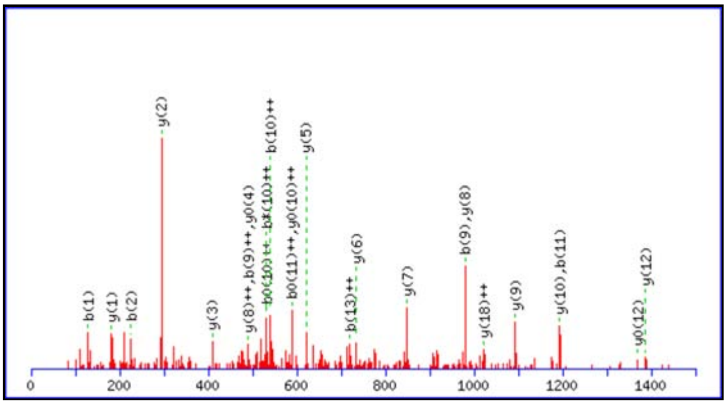

Monoisotopic mass of neutral peptide Mr(calc): 2171.2231
Variable modifications:
R19 : Arginine-13C6 (R-13C6)
Ions Score: 64 Expect: 0.0021
Matches (**Bold Red**): 25/202 fragment ions using 37 most intense peaks

#	b	b^{++}	b^{*}	b^{*++}	b^{0}	b^{0++}	Seq.	y	y^{++}	y^{*}	y^{*++}	y^{0}	y^{0++}	#
1	**129.1022**	65.0548	112.0757	56.5415			K							19
2	**226.1550**	113.5811	209.1284	105.0679			P	2044.1355	**1022.5714**	2027.1089	1014.0581	2026.1249	1013.5661	18
3	339.2391	170.1232	322.2125	161.6099			L	1947.0827	974.0450	1930.0562	965.5317	1929.0721	965.0397	17
4	486.3075	243.6574	469.2809	235.1441			F	1833.9986	917.5030	1816.9721	908.9897	1815.9881	908.4977	16
5	543.3289	272.1681	526.3024	263.6548			G	1686.9302	843.9688	1669.9037	835.4555	1668.9197	834.9635	15
6	672.3715	336.6894	655.3450	328.1761	654.3610	327.6841	E	1629.9088	815.4580	1612.8822	806.9448	1611.8982	806.4527	14
7	785.4556	393.2314	768.4290	384.7182	767.4450	384.2261	I	1500.8662	750.9367	1483.8396	742.4235	1482.8556	741.9314	13
8	842.4770	421.7422	825.4505	413.2289	824.4665	412.7369	G	**1387.7821**	694.3947	1370.7556	685.8814	**1369.7716**	685.3894	12
9	**979.5359**	**490.2716**	962.5094	481.7583	961.5254	481.2663	H	1330.7607	665.8840	1313.7341	657.3707	1312.7501	656.8787	11
10	1080.5836	**540.7955**	1063.5571	**532.2822**	1062.5731	**531.7902**	T	**1193.7018**	597.3545	1176.6752	588.8412	1175.6912	**588.3492**	10
11	**1193.6677**	597.3375	1176.6411	588.8242	1175.6571	**588.3322**	I	**1092.6541**	546.8307	1075.6275	538.3174	1074.6435	537.8254	9
12	1324.7082	662.8577	1307.6816	654.3444	1306.6976	653.8524	M	**979.5700**	**490.2886**	962.5435	481.7754	961.5595	481.2834	8
13	1438.7511	**719.8792**	1421.7245	711.3659	1420.7405	710.8739	N	**848.5295**	424.7684	831.5030	416.2551	830.5190	415.7631	7
14	1551.8352	776.4212	1534.8086	767.9079	1533.8246	767.4159	L	**734.4866**	367.7469	717.4601	359.2337	716.4760	358.7417	6
15	1664.9192	832.9632	1647.8927	824.4500	1646.9087	823.9580	L	**621.4025**	311.2049	604.3760	302.6916	603.3920	302.1996	5
16	1763.9876	882.4975	1746.9611	873.9842	1745.9771	873.4922	V	508.3185	254.6629	491.2919	246.1496	**490.3079**	245.6576	4
17	1879.0146	940.0109	1861.9880	931.4976	1861.0040	931.0056	D	**409.2501**	205.1287	392.2235	196.6154	391.2395	196.1234	3
18	1992.0986	996.5530	1975.0721	988.0397	1974.0881	987.5477	L	**294.2231**	147.6152	277.1966	139.1019			2
19							R	**181.1391**	91.0732	164.1125	82.5599			1

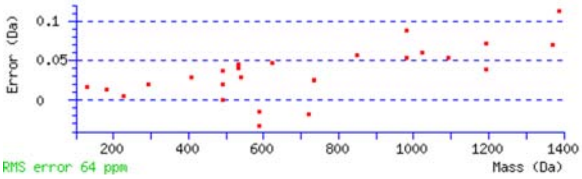

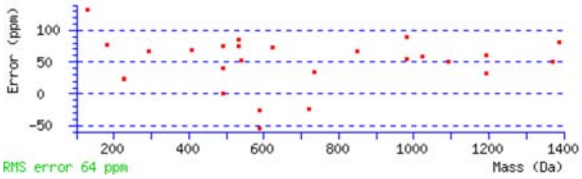

MS/MS Fragmentation of KSEIEYYAMLAK
Found in gi|6677783|ref|NP_033109.1|, ribosomal protein L30 [Mus musculus]

Match to Query 470: 1444.820648 from(723.417600,2+)

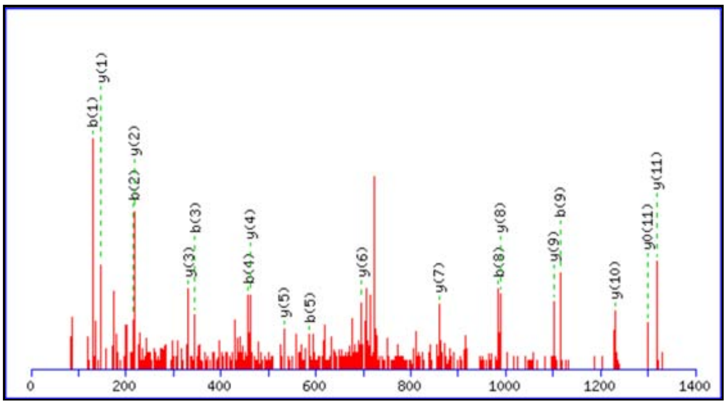

Monoisotopic mass of neutral peptide Mr(calc): 1444.7271
Ions Score: 74 Expect: 6.4e-005
Matches (Bold Red): 20/116 fragment ions using 32 most intense peaks

#	b	b^{++}	b*	b^{*++}	b^0	b^{0++}	Seq.	y	y^{++}	y*	y^{*++}	y^0	y^{0++}	#
1	**129.1022**	65.0548	112.0757	56.5415			K							12
2	**216.1343**	108.5708	199.1077	100.0575	198.1237	99.5655	S	**1317.6395**	659.3234	1300.6129	650.8101	**1299.6289**	650.3181	11
3	**345.1769**	173.0921	328.1503	164.5788	327.1663	164.0868	E	**1230.6075**	615.8074	1213.5809	607.2941	1212.5969	606.8021	10
4	**458.2609**	229.6341	441.2344	221.1208	440.2504	220.6288	I	**1101.5649**	551.2861	1084.5383	542.7728	1083.5543	542.2808	9
5	**587.3035**	294.1554	570.2770	285.6421	569.2929	285.1501	E	**988.4808**	494.7440	971.4543	486.2308	970.4702	485.7388	8
6	750.3668	375.6871	733.3403	367.1738	732.3563	366.6818	Y	**859.4382**	430.2227	842.4117	421.7095			7
7	913.4302	457.2187	896.4036	448.7054	895.4196	448.2134	Y	**696.3749**	348.6911	679.3483	340.1778			6
8	**984.4673**	492.7373	967.4407	484.2240	966.4567	483.7320	A	**533.3116**	267.1594	516.2850	258.6461			5
9	**1115.5078**	558.2575	1098.4812	549.7442	1097.4972	549.2522	M	**462.2745**	231.6409	445.2479	223.1276			4
10	1228.5918	614.7995	1211.5653	606.2863	1210.5813	605.7943	L	**331.2340**	166.1206	314.2074	157.6073			3
11	**1299.6289**	650.3181	1282.6024	641.8048	1281.6184	641.3128	A	**218.1499**	109.5786	201.1234	101.0653			2
12							K	**147.1128**	74.0600	130.0863	65.5468			1

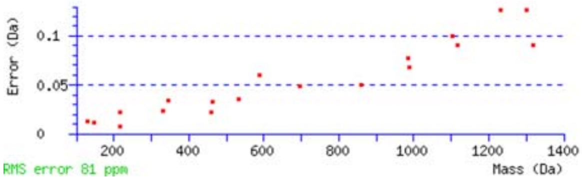

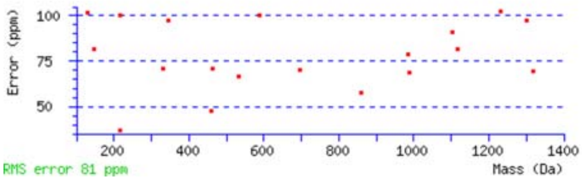

Peptide View

MS/MS Fragmentation of **LGANSLLDLVVFGR**
Found in **gi|54607098|ref|NP_075770.1|**, succinate dehydrogenase Fp subunit [Mus musculus]

Match to Query 459: 1482.862048 from(742.438300,2+)

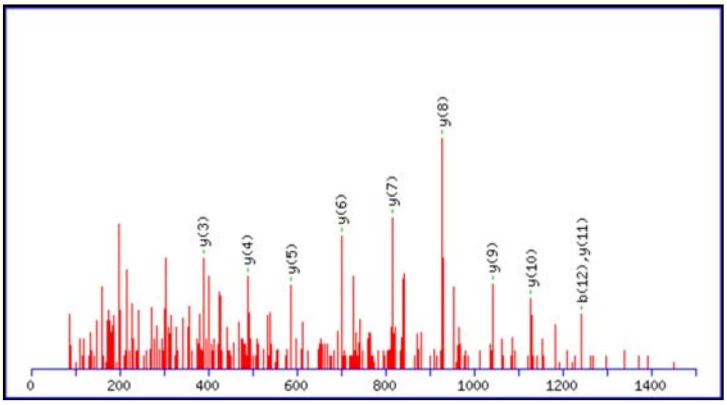

Monoisotopic mass of neutral peptide Mr(calc): 1482.8686
Variable modifications:
R14 : Arginine-13C615N4 (R-full)
Ions Score: 69 **Expect:** 0.00024
Matches (**Bold Red**): 10/130 fragment ions using 18 most intense peaks

#	b	b^{++}	b*	b^{*++}	b^0	b^{0++}	Seq.	y	y^{++}	y*	y^{*++}	y^0	y^{0++}	#
1	114.0913	57.5493					L							14
2	171.1128	86.0600					G	1370.7918	685.8996	1353.7653	677.3863	1352.7813	676.8943	13
3	242.1499	121.5786					A	1313.7704	657.3888	1296.7438	648.8756	1295.7598	648.3835	12
4	356.1928	178.6001	339.1663	170.0868			N	**1242.7333**	621.8703	1225.7067	613.3570	1224.7227	612.8650	11
5	443.2249	222.1161	426.1983	213.6028	425.2143	213.1108	S	**1128.6903**	564.8488	1111.6638	556.3355	1110.6798	555.8435	10
6	556.3089	278.6581	539.2824	270.1448	538.2984	269.6528	L	**1041.6583**	521.3328	1024.6318	512.8195	1023.6478	512.3275	9
7	669.3930	335.2001	652.3664	326.6869	651.3824	326.1948	L	**928.5743**	464.7908	911.5477	456.2775	910.5637	455.7855	8
8	784.4199	392.7136	767.3934	384.2003	766.4094	383.7083	D	**815.4902**	408.2487	798.4636	399.7355	797.4796	399.2435	7
9	897.5040	449.2556	880.4774	440.7424	879.4934	440.2503	L	**700.4633**	350.7353	683.4367	342.2220			6
10	996.5724	498.7898	979.5458	490.2766	978.5618	489.7846	V	**587.3792**	294.1932	570.3526	285.6800			5
11	1095.6408	548.3240	1078.6143	539.8108	1077.6302	539.3188	V	**488.3108**	244.6590	471.2842	236.1458			4
12	**1242.7092**	621.8582	1225.6827	613.3450	1224.6987	612.8530	F	**389.2424**	195.1248	372.2158	186.6116			3
13	1299.7307	650.3690	1282.7041	641.8557	1281.7201	641.3637	G	242.1740	121.5906	225.1474	113.0773			2
14							R	185.1525	93.0799	168.1260	84.5666			1

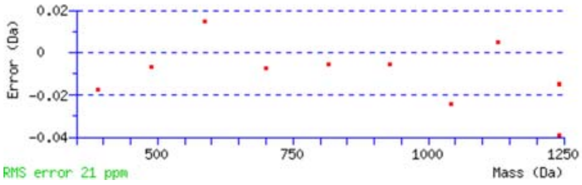

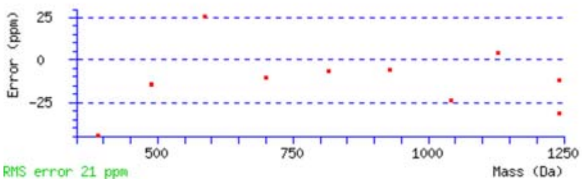

Peptide View

MS/MS Fragmentation of LIEEVHAVVTVR
Found in gi|38348472|ref|NP_941015.1|, adaptor-related protein complex 2, sigma 1 subunit [Mus musculus]

Match to Query 362: 1363.826848 from(682.920700,2+)

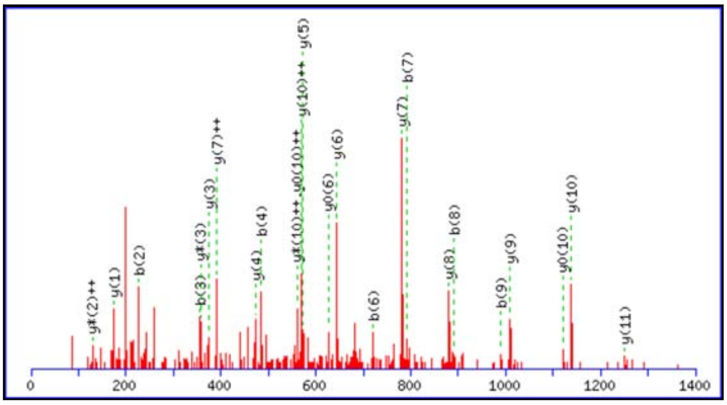

Monoisotopic mass of neutral peptide Mr(calc): 1363.7823
Ions Score: 61 **Expect:** 0.0014
Matches (**Bold Red**): 25/102 fragment ions using 39 most intense peaks

#	b	b^{++}	b^{0}	b^{0++}	Seq.	y	y^{++}	y*	$y*^{++}$	y^{0}	y^{0++}	#
1	114.0913	57.5493			L							12
2	**227.1754**	114.0913			I	**1251.7055**	626.3564	1234.6790	617.8431	1233.6950	617.3511	11
3	**356.2180**	178.6126	338.2074	169.6073	E	**1138.6215**	**569.8144**	1121.5949	**561.3011**	**1120.6109**	**560.8091**	10
4	**485.2606**	243.1339	467.2500	234.1286	E	**1009.5789**	505.2931	992.5523	496.7798	991.5683	496.2878	9
5	584.3290	292.6681	566.3184	283.6628	V	**880.5363**	440.7718	863.5097	432.2585	862.5257	431.7665	8
6	**721.3879**	361.1976	703.3773	352.1923	H	**781.4679**	**391.2376**	764.4413	382.7243	763.4573	382.2323	7
7	**792.4250**	396.7161	774.4144	387.7109	A	**644.4090**	322.7081	627.3824	314.1948	**626.3984**	313.7028	6
8	**891.4934**	446.2503	873.4829	437.2451	V	**573.3719**	287.1896	556.3453	278.6763	555.3613	278.1843	5
9	**990.5618**	495.7846	972.5513	486.7793	V	**474.3035**	237.6554	457.2769	229.1421	456.2929	228.6501	4
10	1091.6095	546.3084	1073.5989	537.3031	T	**375.2350**	188.1212	**358.2085**	179.6079	357.2245	179.1159	3
11	1190.6779	595.8426	1172.6674	586.8373	V	274.1874	137.5973	257.1608	**129.0840**			2
12					R	**175.1190**	88.0631	158.0924	79.5498			1

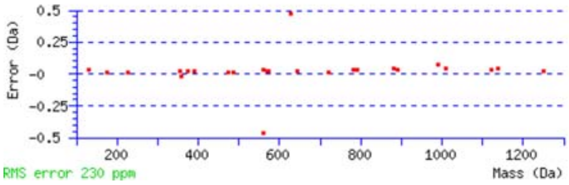

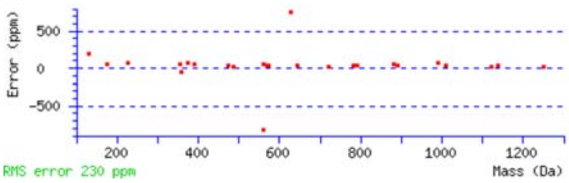

Peptide View

MS/MS Fragmentation of **LIGQIVSSITASLR**
Found in **gi|6755901|ref|NP_035783.1|**, tubulin, alpha 1 [Mus musculus]

Match to Query 297: 1456.936648 from(729.475600,2+)

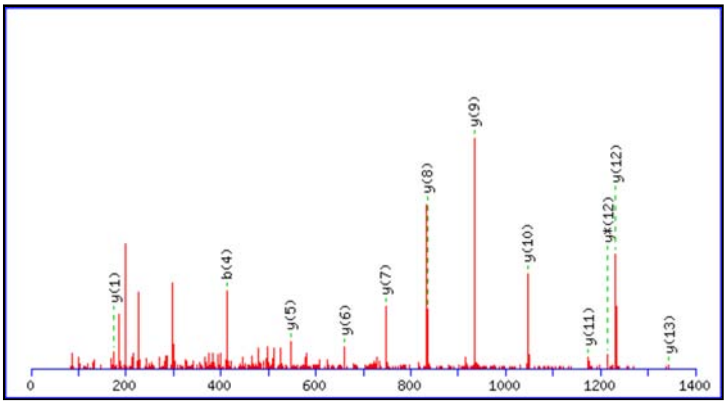

Monoisotopic mass of neutral peptide Mr(calc): 1456.8613
Ions Score: 93 **Expect:** 1.1e-006
Matches (**Bold Red**): 12/134 fragment ions using 20 most intense peaks

#	b	b^{++}	b^{*}	b^{*++}	b^{0}	b^{0++}	Seq.	y	y^{++}	y^{*}	y^{*++}	y^{0}	y^{0++}	#
1	114.0913	57.5493					L							14
2	227.1754	114.0913					I	**1344.7845**	672.8959	1327.7580	664.3826	1326.7740	663.8906	13
3	284.1969	142.6021					G	**1231.7005**	616.3539	**1214.6739**	607.8406	1213.6899	607.3486	12
4	**412.2554**	206.6314	395.2289	198.1181			Q	**1174.6790**	587.8431	1157.6525	579.3299	1156.6684	578.8379	11
5	525.3395	263.1734	508.3129	254.6601			I	**1046.6204**	523.8138	1029.5939	515.3006	1028.6099	514.8086	10
6	624.4079	312.7076	607.3814	304.1943			V	**933.5364**	467.2718	916.5098	458.7585	915.5258	458.2665	9
7	711.4399	356.2236	694.4134	347.7103	693.4294	347.2183	S	**834.4680**	417.7376	817.4414	409.2243	816.4574	408.7323	8
8	798.4720	399.7396	781.4454	391.2263	780.4614	390.7343	S	**747.4359**	374.2216	730.4094	365.7083	729.4254	365.2163	7
9	911.5560	456.2817	894.5295	447.7684	893.5455	447.2764	I	**660.4039**	330.7056	643.3773	322.1923	642.3933	321.7003	6
10	1012.6037	506.8055	995.5772	498.2922	994.5931	497.8002	T	**547.3198**	274.1636	530.2933	265.6503	529.3093	265.1583	5
11	1083.6408	542.3240	1066.6143	533.8108	1065.6303	533.3188	A	446.2722	223.6397	429.2456	215.1264	428.2616	214.6344	4
12	1170.6728	585.8401	1153.6463	577.3268	1152.6623	576.8348	S	375.2350	188.1212	358.2085	179.6079	357.2245	179.1159	3
13	1283.7569	642.3821	1266.7304	633.8688	1265.7463	633.3768	L	288.2030	144.6051	271.1765	136.0919			2
14							R	**175.1190**	88.0631	158.0924	79.5498			1

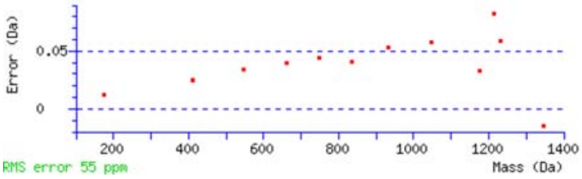

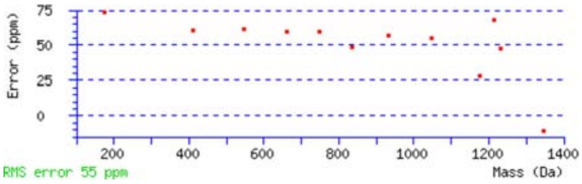

Peptide View

MS/MS Fragmentation of LLQDSVDFSLADAINTEFK
Found in gi|31982755|ref|NP_035831.2|, vimentin [Mus musculus]

Match to Query 760: 2125.234248 from(1063.624400,2+)

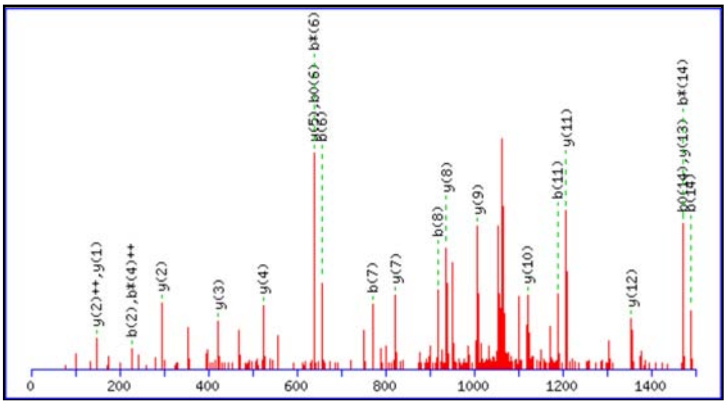

Monoisotopic mass of neutral peptide Mr(calc): 2125.0579
Ions Score: 105 Expect: 1.5e-007
Matches (Bold Red): 24/202 fragment ions using 27 most intense peaks

#	b	b^{++}	b*	$b*^{++}$	b^0	b^{0++}	Seq.	y	y^{++}	y*	$y*^{++}$	y^0	y^{0++}	#
1	114.0913	57.5493					L							19
2	**227.1754**	114.0913					L	2012.9811	1006.9942	1995.9545	998.4809	1994.9705	997.9889	18
3	355.2340	178.1206	338.2074	169.6074			Q	1899.8970	950.4522	1882.8705	941.9389	1881.8865	941.4469	17
4	470.2609	235.6341	453.2344	**227.1208**	452.2504	226.6288	D	1771.8385	886.4229	1754.8119	877.9096	1753.8279	877.4176	16
5	557.2929	279.1501	540.2664	270.6368	539.2824	270.1448	S	1656.8115	828.9094	1639.7850	820.3961	1638.8009	819.9041	15
6	**656.3614**	328.6843	**639.3348**	320.1710	**638.3508**	319.6790	V	1569.7795	785.3934	1552.7529	776.8801	1551.7689	776.3881	14
7	**771.3883**	386.1978	754.3617	377.6845	753.3777	377.1925	D	**1470.7111**	735.8592	1453.6845	727.3459	1452.7005	726.8539	13
8	**918.4567**	459.7320	901.4302	451.2187	900.4461	450.7267	F	**1355.6841**	678.3457	1338.6576	669.8324	1337.6736	669.3404	12
9	1005.4887	503.2480	988.4622	494.7347	987.4782	494.2427	S	**1208.6157**	604.8115	1191.5892	596.2982	1190.6052	595.8062	11
10	1118.5728	559.7900	1101.5462	551.2768	1100.5622	550.7848	L	**1121.5837**	561.2955	1104.5571	552.7822	1103.5731	552.2902	10
11	**1189.6099**	595.3086	1172.5834	586.7953	1171.5993	586.3033	A	**1008.4996**	504.7535	991.4731	496.2402	990.4891	495.7482	9
12	1304.6368	652.8221	1287.6103	644.3088	1286.6263	643.8168	D	**937.4625**	469.2349	920.4360	460.7216	919.4520	460.2296	8
13	1375.6740	688.3406	1358.6474	679.8273	1357.6634	679.3353	A	**822.4356**	411.7214	805.4090	403.2082	804.4250	402.7161	7
14	**1488.7580**	744.8826	**1471.7315**	736.3694	**1470.7475**	735.8774	I	751.3985	376.2029	734.3719	367.6896	733.3879	367.1976	6
15	1602.8009	801.9041	1585.7744	793.3908	1584.7904	792.8988	N	**638.3144**	319.6608	621.2879	311.1476	620.3038	310.6556	5
16	1703.8486	852.4280	1686.8221	843.9147	1685.8381	843.4227	T	**524.2715**	262.6394	507.2449	254.1261	506.2609	253.6341	4
17	1832.8912	916.9492	1815.8647	908.4360	1814.8807	907.9440	E	**423.2238**	212.1155	406.1973	203.6023	405.2132	203.1103	3
18	1979.9596	990.4835	1962.9331	981.9702	1961.9491	981.4782	F	**294.1812**	**147.5942**	277.1547	139.0810			2
19							K	**147.1128**	74.0600	130.0863	65.5468			1

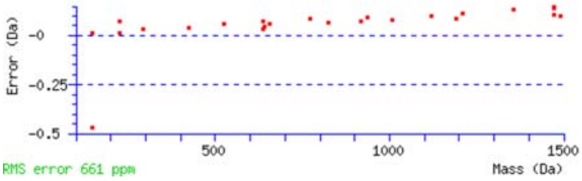

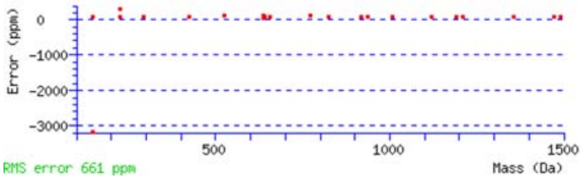

Peptide View

MS/MS Fragmentation of **LMLLLEVISGER**
Found in **gi|11230802|ref|NP_068695.1|**, actinin alpha 4 [Mus musculus]

Match to Query 368: 1371.844448 from(686.929500,2+)

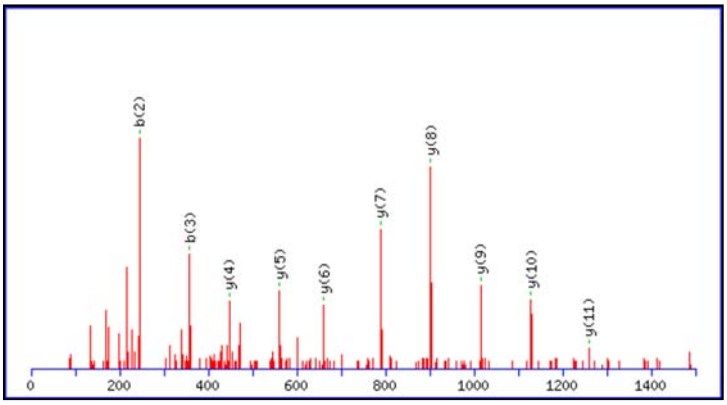

Monoisotopic mass of neutral peptide Mr(calc): 1371.7795
Ions Score: 69 **Expect**: 0.0002
Matches (**Bold Red**): 10/98 fragment ions using 20 most intense peaks

#	b	b^{++}	b^{0}	b^{0++}	Seq.	y	y^{++}	y*	y^{*++}	y^{0}	y^{0++}	#
1	114.0913	57.5493			L							12
2	**245.1318**	123.0695			M	**1259.7028**	630.3550	1242.6762	621.8417	1241.6922	621.3497	11
3	**358.2159**	179.6116			L	**1128.6623**	564.8348	1111.6357	556.3215	1110.6517	555.8295	10
4	471.2999	236.1536			L	**1015.5782**	508.2927	998.5517	499.7795	997.5676	499.2875	9
5	584.3840	292.6956			L	**902.4942**	451.7507	885.4676	443.2374	884.4836	442.7454	8
6	713.4266	357.2169	695.4160	348.2116	E	**789.4101**	395.2087	772.3835	386.6954	771.3995	386.2034	7
7	812.4950	406.7511	794.4844	397.7459	V	**660.3675**	330.6874	643.3410	322.1741	642.3569	321.6821	6
8	925.5791	463.2932	907.5685	454.2879	I	**561.2991**	281.1532	544.2725	272.6399	543.2885	272.1479	5
9	1012.6111	506.8092	994.6005	497.8039	S	**448.2150**	224.6112	431.1885	216.0979	430.2045	215.6059	4
10	1069.6325	535.3199	1051.6220	526.3146	G	361.1830	181.0951	344.1565	172.5819	343.1724	172.0899	3
11	1198.6751	599.8412	1180.6646	590.8359	E	304.1615	152.5844	287.1350	144.0711	286.1510	143.5791	2
12					R	175.1190	88.0631	158.0924	79.5498			1

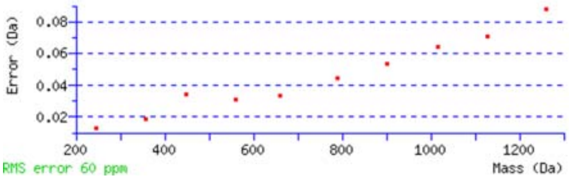

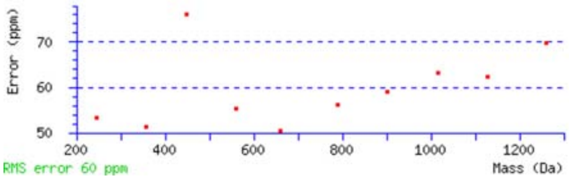

Peptide View

MS/MS Fragmentation of LNYAQWYPIVVFLNPDSK
Found in gi|6678355|ref|NP_033412.1|, tight junction protein 1 [Mus musculus]

Match to Query 532: 2166.216248 from(1084.115400,2+)

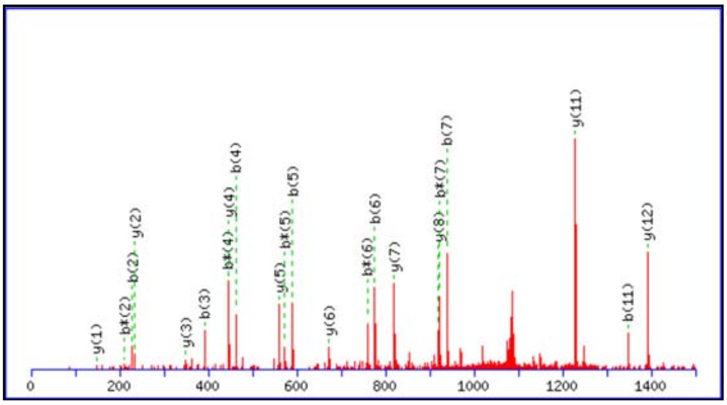

Monoisotopic mass of neutral peptide Mr(calc): 2166.1149
Ions Score: 54 Expect: 0.018
Matches (**Bold Red**): 22/170 fragment ions using 42 most intense peaks

#	b	b^{++}	b*	$b*^{++}$	b^0	b^{0++}	Seq.	y	y^{++}	y*	$y*^{++}$	y^0	y^{0++}	#
1	114.0913	57.5493					L							18
2	**228.1343**	114.5708	**211.1077**	106.0575			N	2054.0382	1027.5227	2037.0116	1019.0094	2036.0276	1018.5174	17
3	**391.1976**	196.1024	374.1710	187.5892			Y	1939.9952	970.5013	1922.9687	961.9880	1921.9847	961.4960	16
4	**462.2347**	231.6210	**445.2082**	223.1077			A	1776.9319	888.9696	1759.9054	880.4563	1758.9213	879.9643	15
5	**590.2933**	295.6503	**573.2667**	287.1370			Q	1705.8948	853.4510	1688.8682	844.9378	1687.8842	844.4458	14
6	**776.3726**	388.6899	**759.3460**	380.1767			W	1577.8362	789.4217	1560.8097	780.9085	1559.8256	780.4165	13
7	**939.4359**	470.2216	**922.4094**	461.7083			Y	**1391.7569**	696.3821	1374.7304	687.8688	1373.7463	687.3768	12
8	1036.4887	518.7480	1019.4621	510.2347			P	**1228.6936**	614.8504	1211.6670	606.3371	1210.6830	605.8451	11
9	1149.5727	575.2900	1132.5462	566.7767			I	1131.6408	566.3240	1114.6143	557.8108	1113.6302	557.3188	10
10	1248.6412	624.8242	1231.6146	616.3109			V	1018.5568	509.7820	1001.5302	501.2687	1000.5462	500.7767	9
11	**1347.7096**	674.3584	1330.6830	665.8451			V	**919.4883**	460.2478	902.4618	451.7345	901.4778	451.2425	8
12	1494.7780	747.8926	1477.7514	739.3794			F	**820.4199**	410.7136	803.3934	402.2003	802.4094	401.7083	7
13	1607.8620	804.4347	1590.8355	795.9214			L	**673.3515**	337.1794	656.3250	328.6661	655.3410	328.1741	6
14	1721.9050	861.4561	1704.8784	852.9428			N	**560.2675**	280.6374	543.2409	272.1241	542.2569	271.6321	5
15	1818.9577	909.9825	1801.9312	901.4692			P	**446.2245**	223.6159	429.1980	215.1026	428.2140	214.6106	4
16	1933.9847	967.4960	1916.9581	958.9827	1915.9741	958.4907	D	**349.1718**	175.0895	332.1452	166.5762	331.1612	166.0842	3
17	2021.0167	1011.0120	2003.9901	1002.4987	2003.0061	1002.0067	S	**234.1448**	117.5761	217.1183	109.0628	216.1343	108.5708	2
18							K	**147.1128**	74.0600	130.0863	65.5468			1

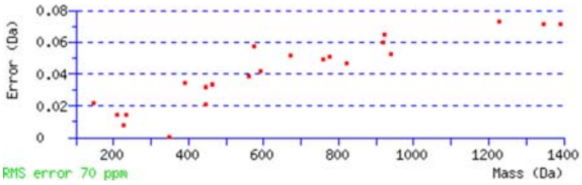

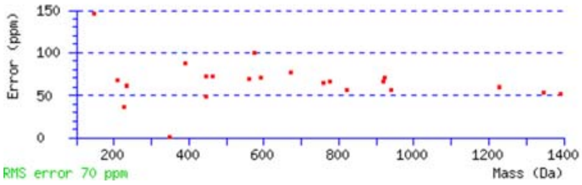

Peptide View

MS/MS Fragmentation of LPAAGVGDMVMATVK
Found in gi|12584986|ref|NP_075029.1|, ribosomal protein L23 [Mus musculus]

Match to Query 298: 1458.823048 from(730.418800,2+)

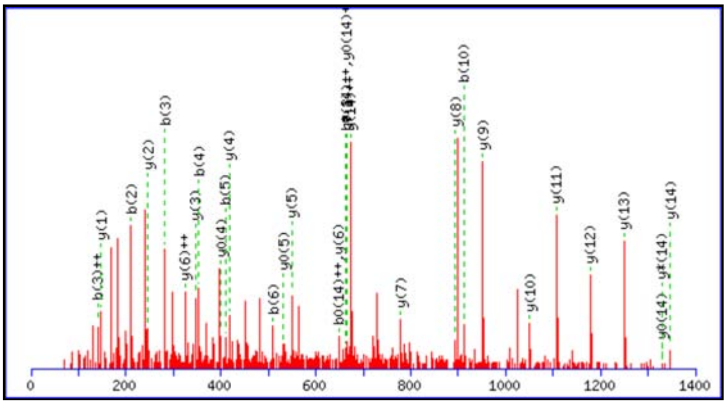

Monoisotopic mass of neutral peptide Mr(calc): 1458.7574
Ions Score: 70 **Expect:** 0.00019
Matches (**Bold Red**): 31/122 fragment ions using 93 most intense peaks

#	b	b^{++}	b^{0}	b^{0++}	Seq.	y	y^{++}	y*	$y*^{++}$	y^{0}	y^{0++}	#
1	114.0913	57.5493			L							15
2	**211.1441**	106.0757			P	**1346.6806**	**673.8439**	**1329.6541**	**665.3307**	**1328.6701**	**664.8387**	14
3	**282.1812**	**141.5942**			A	**1249.6279**	625.3176	1232.6013	616.8043	1231.6173	616.3123	13
4	**353.2183**	177.1128			A	**1178.5908**	589.7990	1161.5642	581.2857	1160.5802	580.7937	12
5	**410.2398**	205.6235			G	**1107.5536**	554.2805	1090.5271	545.7672	1089.5431	545.2752	11
6	**509.3082**	255.1577			V	**1050.5322**	525.7697	1033.5056	517.2565	1032.5216	516.7644	10
7	566.3296	283.6685			G	**951.4638**	476.2355	934.4372	467.7222	933.4532	467.2302	9
8	681.3566	341.1819	**663.3460**	332.1766	D	**894.4423**	447.7248	877.4158	439.2115	876.4317	438.7195	8
9	812.3971	406.7022	794.3865	397.6969	M	**779.4154**	390.2113	762.3888	381.6980	761.4048	381.2060	7
10	**911.4655**	456.2364	893.4549	447.2311	V	**648.3749**	**324.6911**	631.3483	316.1778	630.3643	315.6858	6
11	1042.5060	521.7566	1024.4954	512.7513	M	**549.3065**	275.1569	532.2799	266.6436	**531.2959**	266.1516	5
12	1113.5431	557.2752	1095.5325	548.2699	A	**418.2660**	209.6366	401.2395	201.1234	**400.2554**	200.6314	4
13	1214.5907	607.7990	1196.5802	598.7937	T	**347.2289**	174.1181	330.2023	165.6048	329.2183	165.1128	3
14	1313.6592	657.3332	1295.6486	**648.3279**	V	**246.1812**	123.5942	229.1547	115.0810			2
15					K	**147.1128**	74.0600	130.0863	65.5468			1

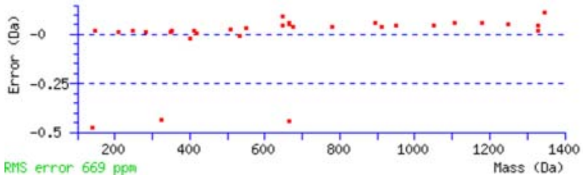

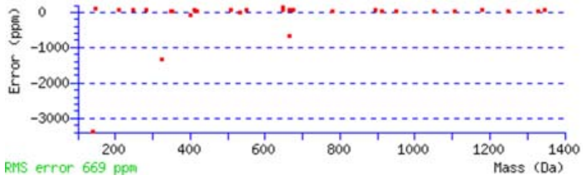

Peptide View

MS/MS Fragmentation of LSLQLALTELETLAEK
Found in gi|40254212|ref|NP_766003.2|, Rho guanine nucleotide exchange factor (GEF) 10-like [Mus musculus]

Match to Query 594: 1771.020048 from(886.517300,2+)

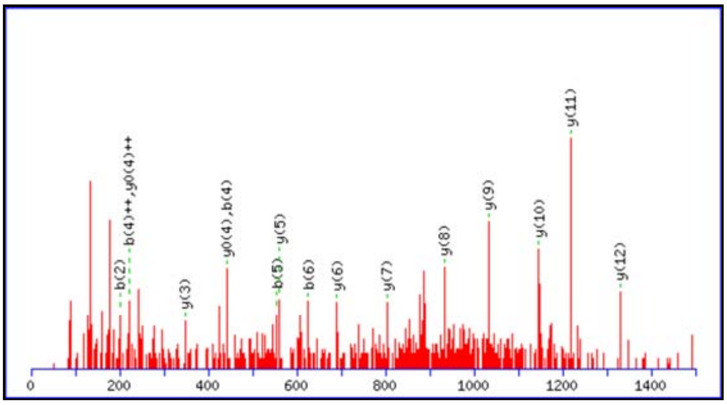

Monoisotopic mass of neutral peptide Mr(calc): 1770.9978
Ions Score: 66 Expect: 0.00081
Matches (Bold Red): 16/170 fragment ions using 27 most intense peaks

#	b	b^{++}	b*	b^{*++}	b^0	b^{0++}	Seq.	y	y^{++}	y*	y^{*++}	y^0	y^{0++}	#
1	114.0913	57.5493					L							16
2	**201.1234**	101.0653			183.1128	92.0600	S	1658.9211	829.9642	1641.8945	821.4509	1640.9105	820.9589	15
3	314.2074	157.6074			296.1969	148.6021	L	1571.8890	786.4482	1554.8625	777.9349	1553.8785	777.4429	14
4	**442.2660**	**221.6366**	425.2395	213.1234	424.2554	212.6314	Q	1458.8050	729.9061	1441.7784	721.3928	1440.7944	720.9008	13
5	**555.3501**	278.1787	538.3235	269.6654	537.3395	269.1734	L	**1330.7464**	665.8768	1313.7198	657.3636	1312.7358	656.8716	12
6	**626.3872**	313.6972	609.3606	305.1840	608.3766	304.6919	A	**1217.6623**	609.3348	1200.6358	600.8215	1199.6518	600.3295	11
7	739.4712	370.2393	722.4447	361.7260	721.4607	361.2340	L	**1146.6252**	573.8162	1129.5987	565.3030	1128.6147	564.8110	10
8	840.5189	420.7631	823.4924	412.2498	822.5084	411.7578	T	**1033.5412**	517.2742	1016.5146	508.7609	1015.5306	508.2689	9
9	969.5615	485.2844	952.5350	476.7711	951.5509	476.2791	E	**932.4935**	466.7504	915.4669	458.2371	914.4829	457.7451	8
10	1082.6456	541.8264	1065.6190	533.3131	1064.6350	532.8211	L	**803.4509**	402.2291	786.4243	393.7158	785.4403	393.2238	7
11	1211.6882	606.3477	1194.6616	597.8344	1193.6776	597.3424	E	**690.3668**	345.6871	673.3403	337.1738	672.3563	336.6818	6
12	1312.7358	656.8716	1295.7093	648.3583	1294.7253	647.8663	T	**561.3242**	281.1658	544.2977	272.6525	543.3137	272.1605	5
13	1425.8199	713.4136	1408.7933	704.9003	1407.8093	704.4083	L	460.2766	230.6419	443.2500	222.1286	**442.2660**	**221.6366**	4
14	1496.8570	748.9321	1479.8305	740.4189	1478.8464	739.9269	A	**347.1925**	174.0999	330.1660	165.5866	329.1819	165.0946	3
15	1625.8996	813.4534	1608.8730	804.9402	1607.8890	804.4482	E	276.1554	138.5813	259.1288	130.0681	258.1448	129.5761	2
16							K	147.1128	74.0600	130.0863	65.5468			1

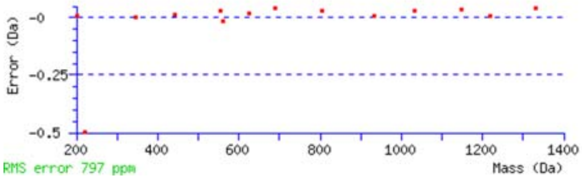

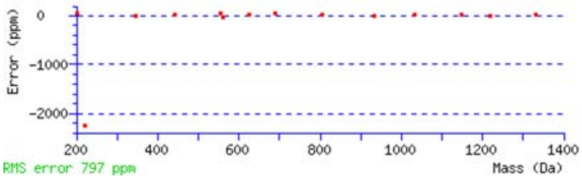

Peptide View

MS/MS Fragmentation of LVLSPADMAAVFINLEDLIK
Found in gi|6678555|ref|NP_033526.1|, Vav2 oncogene [Mus musculus]

Match to Query 702: 2171.237448 from(1086.626000,2+)

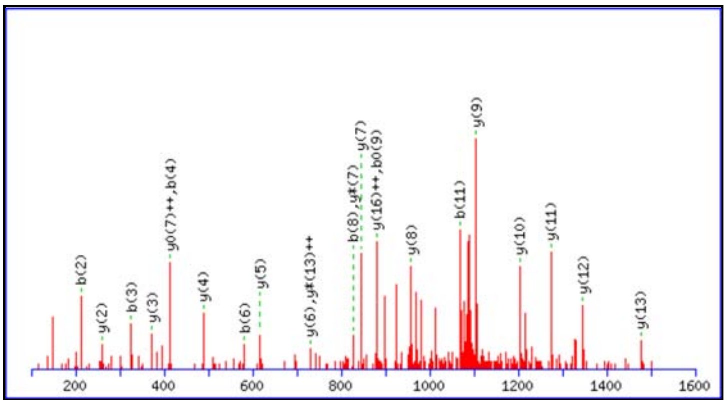

Monoisotopic mass of neutral peptide Mr(calc): 2171.1911
Ions Score: 98 Expect: 7.6e-007
Matches (Bold Red): 23/190 fragment ions using 25 most intense peaks

#	b	b^{++}	b*	b^{*++}	b^0	b^{0++}	Seq.	y	y^{++}	y*	y^{*++}	y^0	y^{0++}	#
1	114.0913	57.5493					L							20
2	**213.1597**	107.0835					V	2059.1143	1030.0608	2042.0878	1021.5475	2041.1038	1021.0555	19
3	**326.2438**	163.6255					L	1960.0459	980.5266	1943.0194	972.0133	1942.0354	971.5213	18
4	**413.2758**	207.1416			395.2653	198.1363	S	1846.9619	923.9846	1829.9353	915.4713	1828.9513	914.9793	17
5	510.3286	255.6679			492.3180	246.6627	P	1759.9298	**880.4686**	1742.9033	871.9553	1741.9193	871.4633	16
6	**581.3657**	291.1865			563.3551	282.1812	A	1662.8771	831.9422	1645.8505	823.4289	1644.8665	822.9369	15
7	696.3926	348.7000			678.3821	339.6947	D	1591.8400	796.4236	1574.8134	787.9103	1573.8294	787.4183	14
8	**827.4331**	414.2202			809.4226	405.2149	M	**1476.8130**	738.9101	1459.7865	**730.3969**	1458.8025	729.9049	13
9	898.4702	449.7388			**880.4597**	440.7335	A	**1345.7725**	673.3899	1328.7460	664.8766	1327.7620	664.3846	12
10	969.5073	485.2573			951.4968	476.2520	A	**1274.7354**	637.8714	1257.7089	629.3581	1256.7249	628.8661	11
11	**1068.5758**	534.7915			1050.5652	525.7862	V	**1203.6983**	602.3528	1186.6718	593.8395	1185.6878	593.3475	10
12	1215.6442	608.3257			1197.6336	599.3204	F	**1104.6299**	552.8186	1087.6034	544.3053	1086.6193	543.8133	9
13	1328.7282	664.8678			1310.7177	655.8625	I	**957.5615**	479.2844	940.5350	470.7711	939.5509	470.2791	8
14	1442.7712	721.8892	1425.7446	713.3759	1424.7606	712.8839	N	**844.4774**	422.7424	**827.4509**	414.2291	826.4669	**413.7371**	7
15	1555.8552	778.4312	1538.8287	769.9180	1537.8447	769.4260	L	**730.4345**	365.7209	713.4080	357.2076	712.4239	356.7156	6
16	1684.8978	842.9525	1667.8713	834.4393	1666.8872	833.9473	E	**617.3505**	309.1789	600.3239	300.6656	599.3399	300.1736	5
17	1799.9247	900.4660	1782.8982	891.9527	1781.9142	891.4607	D	**488.3079**	244.6576	471.2813	236.1443	470.2973	235.6523	4
18	1913.0088	957.0080	1895.9823	948.4948	1894.9982	948.0028	L	**373.2809**	187.1441	356.2544	178.6308			3
19	2026.0929	1013.5501	2009.0663	1005.0368	2008.0823	1004.5448	I	**260.1969**	130.6021	243.1703	122.0888			2
20							K	147.1128	74.0600	130.0863	65.5468			1

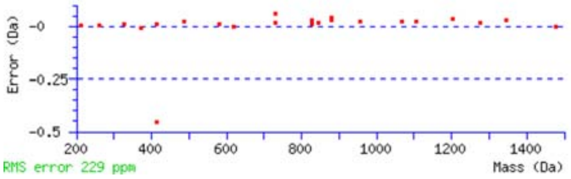

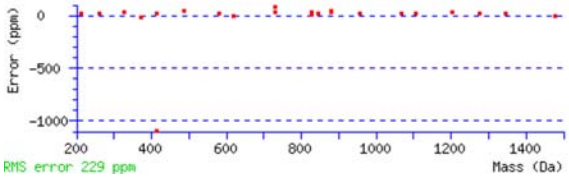

Peptide View

MS/MS Fragmentation of LYTLVTYVPVTTFK
Found in gi|94398865|ref|XP_996947.1|, PREDICTED: similar to ribosomal protein L31 [Mus musculus]

Match to Query 941: 1643.990048 from(823.002300,2+)

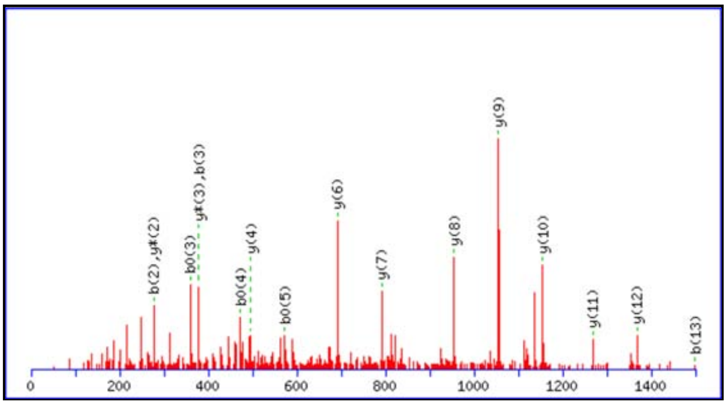

Monoisotopic mass of neutral peptide Mr(calc): 1643.9174
Ions Score: 54 **Expect:** 0.011
Matches (Bold Red): 16/122 fragment ions using 36 most intense peaks

#	b	b++	b0	b0++	Seq.	y	y++	y*	y*++	y0	y0++	#
1	114.0913	57.5493			L							14
2	**277.1547**	139.0810			Y	1531.8406	766.4240	1514.8141	757.9107	1513.8301	757.4187	13
3	**378.2023**	189.6048	**360.1918**	180.5995	T	**1368.7773**	684.8923	1351.7508	676.3790	1350.7667	675.8870	12
4	491.2864	246.1468	**473.2758**	237.1416	L	**1267.7296**	634.3685	1250.7031	625.8552	1249.7191	625.3632	11
5	590.3548	295.6810	**572.3443**	286.6758	V	**1154.6456**	577.8264	1137.6190	569.3131	1136.6350	568.8211	10
6	691.4025	346.2049	673.3919	337.1996	T	**1055.5772**	528.2922	1038.5506	519.7789	1037.5666	519.2869	9
7	854.4658	427.7366	836.4553	418.7313	Y	**954.5295**	477.7684	937.5029	469.2551	936.5189	468.7631	8
8	953.5342	477.2708	935.5237	468.2655	V	**791.4662**	396.2367	774.4396	387.7234	773.4556	387.2314	7
9	1050.5870	525.7971	1032.5764	516.7919	P	**692.3977**	346.7025	675.3712	338.1892	674.3872	337.6972	6
10	1149.6554	575.3313	1131.6448	566.3261	V	595.3450	298.1761	578.3184	289.6629	577.3344	289.1708	5
11	1250.7031	625.8552	1232.6925	616.8499	T	**496.2766**	248.6419	479.2500	240.1286	478.2660	239.6366	4
12	1351.7508	676.3790	1333.7402	667.3737	T	395.2289	198.1181	**378.2023**	189.6048	377.2183	189.1128	3
13	**1498.8192**	749.9132	1480.8086	740.9079	F	294.1812	147.5942	**277.1547**	139.0810			2
14					K	147.1128	74.0600	130.0863	65.5468			1

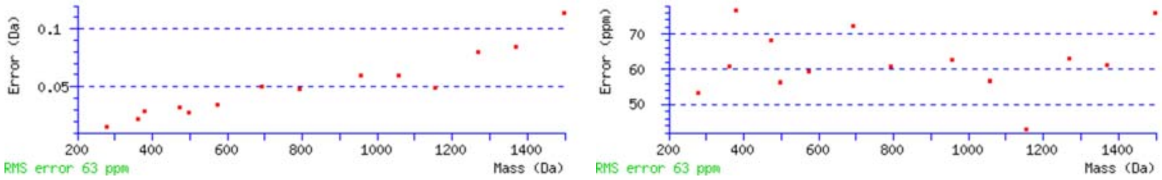

Peptide View

MS/MS Fragmentation of MLGSPVDSVLFYAITTLHNLLLHQEGAK
Found in gi|6671684|ref|NP_031640.1|, catenin (cadherin associated protein), beta 1, 88kDa [Mus musculus]

Match to Query 865: 3066.676096 from(767.676300,4+)

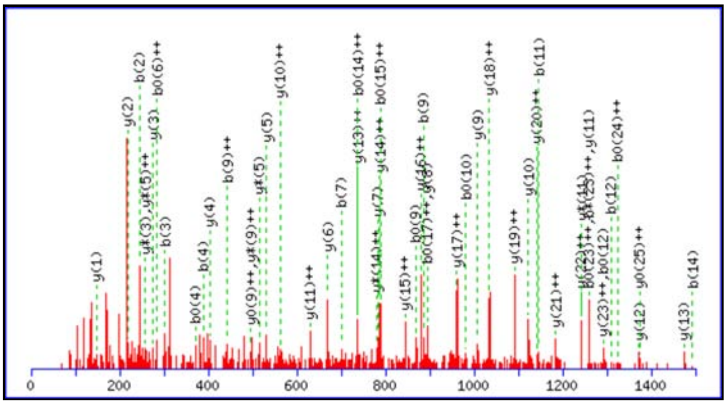

Monoisotopic mass of neutral peptide Mr(calc): 3066.6211
Ions Score: 68 Expect: 0.0016
Matches (**Bold Red**): 54/276 fragment ions using 134 most intense peaks

#	b	b++	b*	b*++	b0	b0++	Seq.	y	y++	y*	y*++	y0	y0++	#
1	132.0478	66.5275					M							28
2	**245.1318**	123.0695					L	2936.5880	1468.7976	2919.5614	1460.2843	2918.5774	1459.7923	27
3	**302.1533**	151.5803					G	2823.5039	1412.2556	2806.4774	1403.7423	2805.4933	1403.2503	26
4	**389.1853**	195.0963			**371.1747**	186.0910	S	2766.4824	1383.7449	2749.4559	1375.2316	2748.4719	**1374.7396**	25
5	486.2381	243.6227			468.2275	234.6174	P	2679.4504	1340.2288	2662.4239	1331.7156	2661.4398	1331.2236	24
6	585.3065	293.1569			567.2959	**284.1516**	V	2582.3977	**1291.7025**	2565.3711	1283.1892	2564.3871	1282.6972	23
7	**700.3334**	350.6703			682.3229	341.6651	D	2483.3292	**1242.1683**	2466.3027	1233.6550	2465.3187	1233.1630	22
8	787.3654	394.1864			769.3549	385.1811	S	2368.3023	**1184.6548**	2351.2758	1176.1415	2350.2917	1175.6495	21
9	**886.4339**	**443.7206**			**868.4233**	434.7153	V	2281.2703	**1141.1388**	2264.2437	1132.6255	2263.2597	1132.1335	20
10	999.5179	500.2626			**981.5074**	491.2573	L	2182.2019	**1091.6046**	2165.1753	1083.0913	2164.1913	1082.5993	19
11	**1146.5863**	573.7968			1128.5758	564.7915	F	2069.1178	**1035.0625**	2052.0913	1026.5493	2051.1072	1026.0573	18
12	**1309.6497**	655.3285			**1291.6391**	646.3232	Y	1922.0494	**961.5283**	1905.0228	953.0151	1904.0388	952.5231	17
13	1380.6868	690.8470			1362.6762	681.8417	A	1758.9861	**879.9967**	1741.9595	871.4834	1740.9755	870.9914	16
14	**1493.7708**	747.3891			1475.7603	**738.3838**	I	1687.9490	**844.4781**	1670.9224	835.9648	1669.9384	835.4728	15
15	1594.8185	797.9129			1576.8079	**788.9076**	T	1574.8649	**787.9361**	1557.8383	**779.4228**	1556.8543	778.9308	14
16	1695.8662	848.4367			1677.8556	839.4314	T	**1473.8172**	**737.4122**	1456.7907	728.8990	1455.8066	728.4070	13
17	1808.9502	904.9788			1790.9397	**895.9735**	L	**1372.7695**	686.8884	1355.7430	678.3751	1354.7590	677.8831	12
18	1946.0092	973.5082			1927.9986	964.5029	H	**1259.6855**	**630.3464**	**1242.6589**	621.8331	1241.6749	621.3411	11
19	2060.0521	1030.5297	2043.0255	1022.0164	2042.0415	1021.5244	N	**1122.6266**	**561.8169**	1105.6000	553.3036	1104.6160	552.8116	10
20	2173.1361	1087.0717	2156.1096	1078.5584	2155.1256	1078.0664	L	**1008.5836**	504.7955	991.5571	**496.2822**	990.5731	**495.7902**	9
21	2286.2202	1143.6137	2269.1937	1135.1005	2268.2096	1134.6085	L	**895.4996**	448.2534	878.4730	439.7401	877.4890	439.2481	8
22	2399.3043	1200.1558	2382.2777	1191.6425	2381.2937	1191.1505	L	**782.4155**	391.7114	765.3890	383.1981	764.4049	382.7061	7
23	2536.3632	1268.6852	2519.3366	**1260.1720**	2518.3526	**1259.6799**	H	**669.3315**	335.1694	652.3049	326.6561	651.3209	326.1641	6
24	2664.4218	1332.7145	2647.3952	1324.2012	2646.4112	**1323.7092**	Q	**532.2725**	266.6399	**515.2460**	**258.1266**	514.2620	257.6346	5
25	2793.4643	1397.2358	2776.4378	1388.7225	2775.4538	1388.2305	E	**404.2140**	202.6106	387.1874	194.0973	386.2034	193.6053	4
26	2850.4858	1425.7465	2833.4593	1417.2333	2832.4752	1416.7413	G	**275.1714**	138.0893	**258.1448**	129.5760			3
27	2921.5229	1461.2651	2904.4964	1452.7518	2903.5124	1452.2598	A	**218.1499**	109.5786	201.1234	101.0653			2
28							K	**147.1128**	74.0600	130.0863	65.5468			1

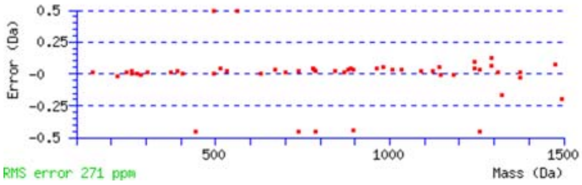

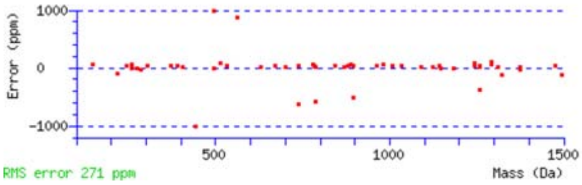

Peptide View

MS/MS Fragmentation of MNFANVFIGANPLAVDLLEK
Found in gi|10092590|ref|NP_036081.1|, mitogen activated protein kinase 14 [Mus musculus]

Match to Query 1164: 2175.267848 from(1088.641200,2+)

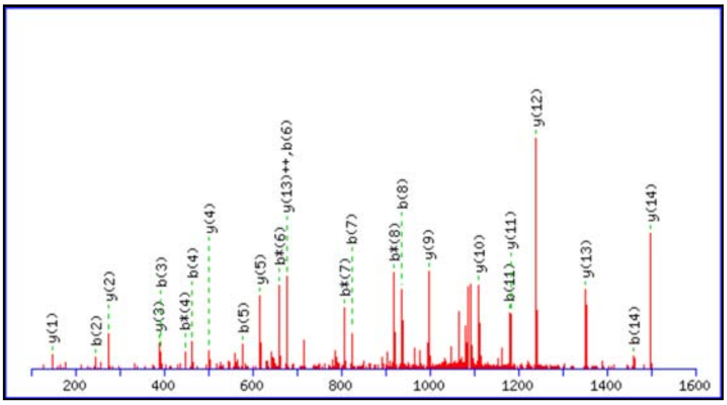

Monoisotopic mass of neutral peptide Mr(calc): 2175.1397
Ions Score: 100 **Expect:** 5.1e-007
Matches (**Bold Red**): 25/194 fragment ions using 31 most intense peaks

#	b	b^{++}	b*	b^{*++}	b^0	b^{0++}	Seq.	y	y^{++}	y*	y^{*++}	y^0	y^{0++}	#
1	132.0478	66.5275					M							20
2	**246.0907**	123.5490	229.0641	115.0357			N	2045.1066	1023.0569	2028.0800	1014.5436	2027.0960	1014.0516	19
3	**393.1591**	197.0832	376.1325	188.5699			F	1931.0636	966.0354	1914.0371	957.5222	1913.0531	957.0302	18
4	**464.1962**	232.6017	**447.1697**	224.0885			A	1783.9952	892.5012	1766.9687	883.9880	1765.9846	883.4960	17
5	**578.2391**	289.6232	561.2126	281.1099			N	1712.9581	856.9827	1695.9316	848.4694	1694.9475	847.9774	16
6	**677.3075**	339.1574	**660.2810**	330.6441			V	1598.9152	799.9612	1581.8886	791.4479	1580.9046	790.9559	15
7	**824.3760**	412.6916	**807.3494**	404.1783			F	**1499.8468**	750.4270	1482.8202	741.9137	1481.8362	741.4217	14
8	**937.4600**	469.2336	**920.4335**	460.7204			I	**1352.7784**	**676.8928**	1335.7518	668.3795	1334.7678	667.8875	13
9	994.4815	497.7444	977.4549	489.2311			G	**1239.6943**	620.3508	1222.6677	611.8375	1221.6837	611.3455	12
10	1065.5186	533.2629	1048.4920	524.7497			A	**1182.6728**	591.8401	1165.6463	583.3268	1164.6623	582.8348	11
11	**1179.5615**	590.2844	1162.5350	581.7711			N	**1111.6357**	556.3215	1094.6092	547.8082	1093.6252	547.3162	10
12	1276.6143	638.8108	1259.5877	630.2975			P	**997.5928**	499.3000	980.5662	490.7868	979.5822	490.2948	9
13	1389.6983	695.3528	1372.6718	686.8395			L	900.5400	450.7737	883.5135	442.2604	882.5295	441.7684	8
14	**1460.7354**	730.8714	1443.7089	722.3581			A	787.4560	394.2316	770.4294	385.7183	769.4454	385.2263	7
15	1559.8039	780.4056	1542.7773	771.8923			V	716.4189	358.7131	699.3923	350.1998	698.4083	349.7078	6
16	1674.8308	837.9190	1657.8042	829.4058	1656.8202	828.9138	D	**617.3505**	309.1789	600.3239	300.6656	599.3399	300.1736	5
17	1787.9149	894.4611	1770.8883	885.9478	1769.9043	885.4558	L	**502.3235**	251.6654	485.2970	243.1521	484.3129	242.6601	4
18	1900.9989	951.0031	1883.9724	942.4898	1882.9884	941.9978	L	**389.2395**	195.1234	372.2129	186.6101	371.2289	186.1181	3
19	2030.0415	1015.5244	2013.0150	1007.0111	2012.0309	1006.5191	E	**276.1554**	138.5813	259.1288	130.0681	258.1448	129.5761	2
20							K	**147.1128**	74.0600	130.0863	65.5468			1

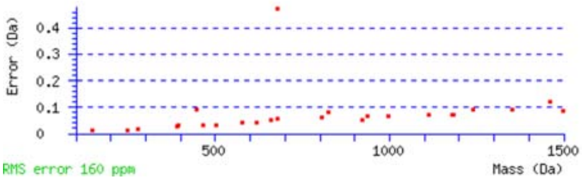

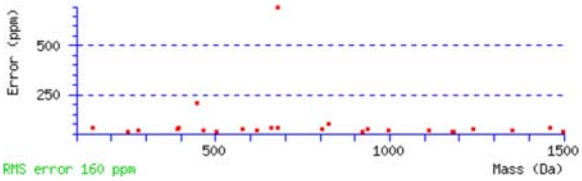

Peptide View

MS/MS Fragmentation of MQSLTLDVLGTSELLLAK
Found in gi|94419105|ref|XP_001005781.1|, PREDICTED: similar to Development and differentiation-enhancing factor 2 (Pyk2 C-terminus associate

Match to Query 466: 1931.148448 from(966.581500,2+)

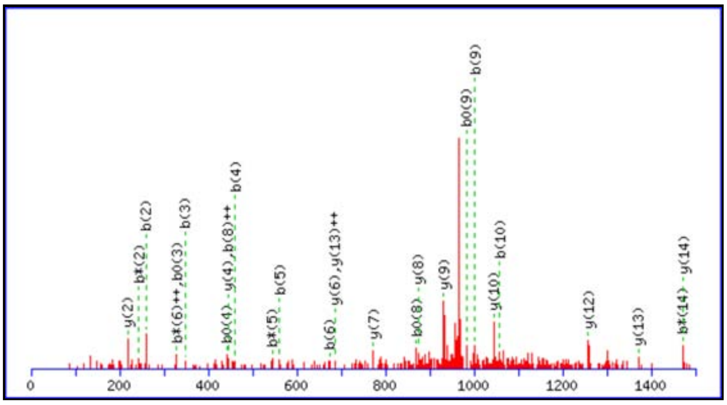

Monoisotopic mass of neutral peptide Mr(calc): 1931.0649
Ions Score: 50 **Expect:** 0.038
Matches (Bold Red): 27/188 fragment ions using 55 most intense peaks

#	b	b^{++}	b*	$b*^{++}$	b^0	b^{0++}	Seq.	y	y^{++}	y*	$y*^{++}$	y^0	y^{0++}	#
1	132.0478	66.5275					M							18
2	**260.1063**	130.5568	**243.0798**	122.0435			Q	1801.0317	901.0195	1784.0051	892.5062	1783.0211	892.0142	17
3	**347.1384**	174.0728	330.1118	165.5595	**329.1278**	165.0675	S	1672.9731	836.9902	1655.9465	828.4769	1654.9625	827.9849	16
4	**460.2224**	230.6149	443.1959	222.1016	**442.2119**	221.6096	L	1585.9411	793.4742	1568.9145	784.9609	1567.9305	784.4689	15
5	**561.2701**	281.1387	**544.2436**	272.6254	543.2595	272.1334	T	**1472.8570**	736.9321	1455.8305	728.4189	1454.8464	727.9269	14
6	**674.3542**	337.6807	657.3276	**329.1674**	656.3436	328.6754	L	**1371.8093**	**686.4083**	1354.7828	677.8950	1353.7988	677.4030	13
7	789.3811	395.1942	772.3546	386.6809	771.3705	386.1889	D	**1258.7253**	629.8663	1241.6987	621.3530	1240.7147	620.8610	12
8	888.4495	**444.7284**	871.4230	436.2151	**870.4390**	435.7231	V	1143.6983	572.3528	1126.6718	563.8395	1125.6878	563.3475	11
9	**1001.5336**	501.2704	984.5070	492.7572	**983.5230**	492.2651	L	**1044.6299**	522.8186	1027.6034	514.3053	1026.6193	513.8133	10
10	**1058.5550**	529.7812	1041.5285	521.2679	1040.5445	520.7759	G	**931.5459**	466.2766	914.5193	457.7633	913.5353	457.2713	9
11	1159.6027	580.3050	1142.5762	571.7917	1141.5922	571.2997	T	**874.5244**	437.7658	857.4978	429.2526	856.5138	428.7606	8
12	1246.6347	623.8210	1229.6082	615.3077	1228.6242	614.8157	S	**773.4767**	387.2420	756.4502	378.7287	755.4661	378.2367	7
13	1375.6773	688.3423	1358.6508	679.8290	1357.6668	679.3370	E	**686.4447**	343.7260	669.4181	335.2127	668.4341	334.7207	6
14	1488.7614	744.8843	**1471.7348**	736.3711	1470.7508	735.8791	L	557.4021	279.2047	540.3755	270.6914			5
15	1601.8455	801.4264	1584.8189	792.9131	1583.8349	792.4211	L	**444.3180**	222.6627	427.2915	214.1494			4
16	1714.9295	857.9684	1697.9030	849.4551	1696.9190	848.9631	L	331.2340	166.1206	314.2074	157.6073			3
17	1785.9666	893.4870	1768.9401	884.9737	1767.9561	884.4817	A	**218.1499**	109.5786	201.1234	101.0653			2
18							K	147.1128	74.0600	130.0863	65.5468			1

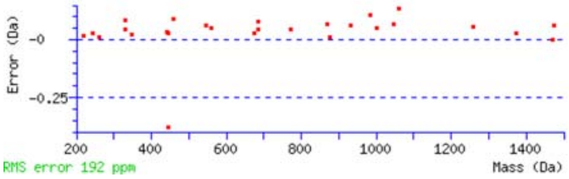

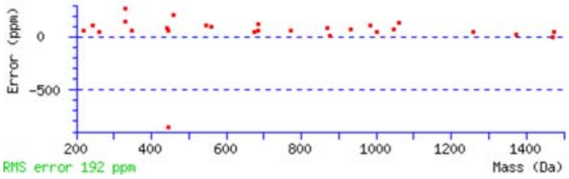

Peptide View

MS/MS Fragmentation of **MSATFIGNSTAIQELFK**
Found in **gi|21746161|ref|NP_076205.1|**, tubulin, beta [Mus musculus]

Match to Query 625: 1856.952648 from(929.483600,2+)

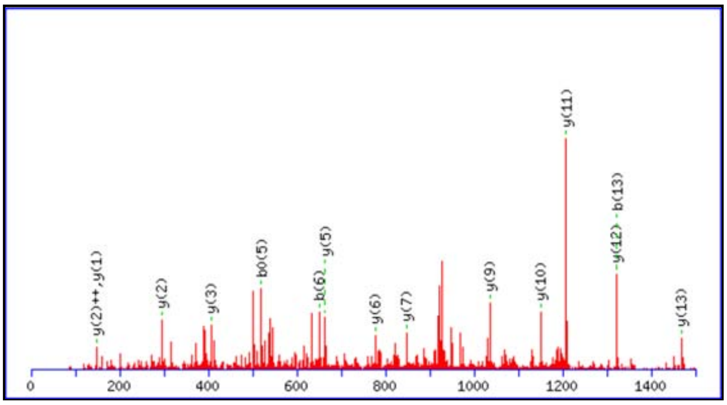

Monoisotopic mass of neutral peptide Mr(calc): 1856.9342
Ions Score: 77 **Expect:** 6.2e-005
Matches (Bold Red): 15/170 fragment ions using 32 most intense peaks

#	b	b^{++}	b*	b^{*++}	b^0	b^{0++}	Seq.	y	y^{++}	y*	y^{*++}	y^0	y^{0++}	#
1	132.0478	66.5275					M							17
2	219.0798	110.0435			201.0692	101.0382	S	1726.9010	863.9541	1709.8745	855.4409	1708.8904	854.9489	16
3	290.1169	145.5621			272.1063	136.5568	A	1639.8690	820.4381	1622.8424	811.9248	1621.8584	811.4328	15
4	391.1646	196.0859			373.1540	187.0806	T	1568.8319	784.9196	1551.8053	776.4063	1550.8213	775.9143	14
5	538.2330	269.6201			**520.2224**	260.6148	F	**1467.7842**	734.3957	1450.7576	725.8825	1449.7736	725.3904	13
6	**651.3170**	326.1622			633.3065	317.1569	I	**1320.7158**	660.8615	1303.6892	652.3482	1302.7052	651.8562	12
7	708.3385	354.6729			690.3279	345.6676	G	**1207.6317**	604.3195	1190.6052	595.8062	1189.6211	595.3142	11
8	822.3814	411.6944	805.3549	403.1811	804.3709	402.6891	N	**1150.6103**	575.8088	1133.5837	567.2955	1132.5997	566.8035	10
9	909.4135	455.2104	892.3869	446.6971	891.4029	446.2051	S	**1036.5673**	518.7873	1019.5408	510.2740	1018.5568	509.7820	9
10	1010.4611	505.7342	993.4346	497.2209	992.4506	496.7289	T	949.5353	475.2713	932.5087	466.7580	931.5247	466.2660	8
11	1081.4983	541.2528	1064.4717	532.7395	1063.4877	532.2475	A	**848.4876**	424.7474	831.4611	416.2342	830.4770	415.7422	7
12	1194.5823	597.7948	1177.5558	589.2815	1176.5718	588.7895	I	**777.4505**	389.2289	760.4240	380.7156	759.4399	380.2236	6
13	**1322.6409**	661.8241	1305.6143	653.3108	1304.6303	652.8188	Q	**664.3664**	332.6869	647.3399	324.1736	646.3559	323.6816	5
14	1451.6835	726.3454	1434.6569	717.8321	1433.6729	717.3401	E	536.3079	268.6576	519.2813	260.1443	518.2973	259.6523	4
15	1564.7675	782.8874	1547.7410	774.3741	1546.7570	773.8821	L	**407.2653**	204.1363	390.2387	195.6230			3
16	1711.8360	856.4216	1694.8094	847.9083	1693.8254	847.4163	F	**294.1812**	**147.5942**	277.1547	139.0810			2
17							K	**147.1128**	74.0600	130.0863	65.5468			1

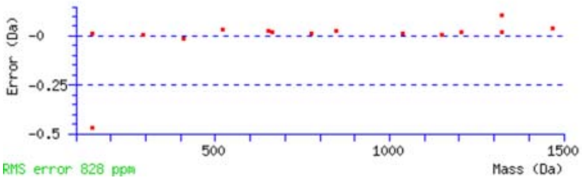

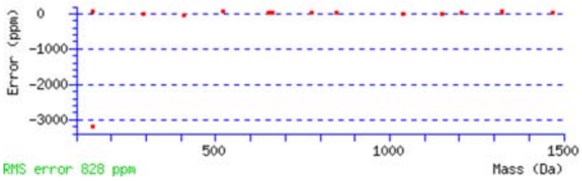

Peptide View

MS/MS Fragmentation of MTQIMFEAFNTPAMYVAIQAVLSLYASGR
Found in gi|30425250|ref|NP_780706.1|, hypothetical protein LOC238880 [Mus musculus]

Match to Query 898: 3253.778472 from(1085.600100,3+)

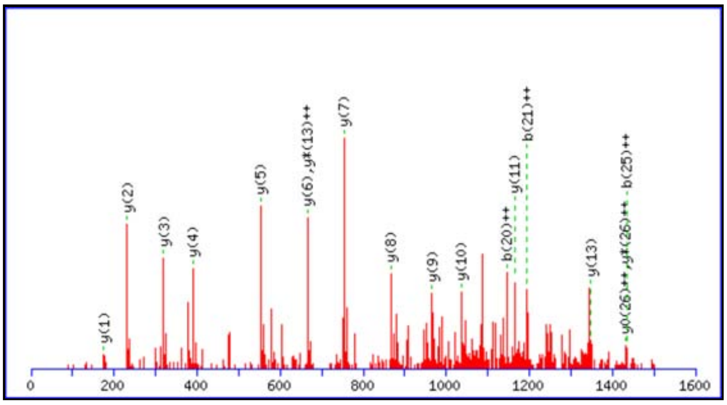

Monoisotopic mass of neutral peptide Mr(calc): 3254.5813
Variable modifications:
M1 : Oxidation (M)
M14 : Oxidation (M)
Ions Score: 62 Expect: 0.0065
Matches (**Bold Red**): 18/326 fragment ions using 27 most intense peaks

#	b	b^{++}	b*	b^{*++}	b^{0}	b^{0++}	Seq.	y	y^{++}	y*	y^{*++}	y^{0}	y^{0++}	#
1	148.0427	74.5250					M							29
2	249.0904	125.0488			231.0798	116.0435	T	3108.5532	1554.7803	3091.5267	1546.2670	3090.5427	1545.7750	28
3	377.1489	189.0781	360.1224	180.5648	359.1384	180.0728	Q	3007.5056	1504.2564	2990.4790	1495.7431	2989.4950	1495.2511	27
4	490.2330	245.6201	473.2064	237.1069	472.2224	236.6149	I	2879.4470	1440.2271	2862.4204	**1431.7139**	2861.4364	**1431.2218**	26
5	621.2735	311.1404	604.2469	302.6271	603.2629	302.1351	M	2766.3629	1383.6851	2749.3364	1375.1718	2748.3524	1374.6798	25
6	768.3419	384.6746	751.3153	376.1613	750.3313	375.6693	F	2635.3224	1318.1649	2618.2959	1309.6516	2617.3119	1309.1596	24
7	897.3845	449.1959	880.3579	440.6826	879.3739	440.1906	E	2488.2540	1244.6307	2471.2275	1236.1174	2470.2435	1235.6254	23
8	968.4216	484.7144	951.3950	476.2012	950.4110	475.7091	A	2359.2114	1180.1094	2342.1849	1171.5961	2341.2009	1171.1041	22
9	1115.4900	558.2486	1098.4634	549.7354	1097.4794	549.2434	F	2288.1743	1144.5908	2271.1478	1136.0775	2270.1638	1135.5855	21
10	1229.5329	615.2701	1212.5064	606.7568	1211.5224	606.2648	N	2141.1059	1071.0566	2124.0794	1062.5433	2123.0954	1062.0513	20
11	1330.5806	665.7939	1313.5541	657.2807	1312.5700	656.7887	T	2027.0630	1014.0351	2010.0364	1005.5219	2009.0524	1005.0299	19
12	1427.6334	714.3203	1410.6068	705.8070	1409.6228	705.3150	P	1926.0153	963.5113	1908.9888	954.9980	1908.0047	954.5060	18
13	1498.6705	749.8389	1481.6439	741.3256	1480.6599	740.8336	A	1828.9626	914.9849	1811.9360	906.4716	1810.9520	905.9796	17
14	1645.7059	823.3566	1628.6793	814.8433	1627.6953	814.3513	M	1757.9254	879.4664	1740.8989	870.9531	1739.9149	870.4611	16
15	1808.7692	904.8882	1791.7427	896.3750	1790.7586	895.8830	Y	1610.8900	805.9487	1593.8635	797.4354	1592.8795	796.9434	15
16	1907.8376	954.4224	1890.8111	945.9092	1889.8270	945.4172	V	1447.8267	724.4170	1430.8002	715.9037	1429.8161	715.4117	14
17	1978.8747	989.9410	1961.8482	981.4277	1960.8642	980.9357	A	**1348.7583**	674.8828	1331.7318	**666.3695**	1330.7477	665.8775	13
18	2091.9588	1046.4830	2074.9322	1037.9698	2073.9482	1037.4777	I	1277.7212	639.3642	1260.6946	630.8510	1259.7106	630.3590	12
19	2220.0174	1110.5123	2202.9908	1101.9990	2202.0068	1101.5070	Q	**1164.6371**	582.8222	1147.6106	574.3089	1146.6266	573.8169	11
20	2291.0545	**1146.0309**	2274.0279	1137.5176	2273.0439	1137.0256	A	**1036.5786**	518.7929	1019.5520	510.2796	1018.5680	509.7876	10
21	2390.1229	**1195.5651**	2373.0963	1187.0518	2372.1123	1186.5598	V	**965.5414**	483.2744	948.5149	474.7611	947.5309	474.2691	9
22	2503.2069	1252.1071	2486.1804	1243.5938	2485.1964	1243.1018	L	**866.4730**	433.7402	849.4465	425.2269	848.4625	424.7349	8
23	2590.2390	1295.6231	2573.2124	1287.1099	2572.2284	1286.6178	S	**753.3890**	377.1981	736.3624	368.6848	735.3784	368.1928	7
24	2703.3230	1352.1652	2686.2965	1343.6519	2685.3125	1343.1599	L	**666.3569**	333.6821	649.3304	325.1688	648.3464	324.6768	6
25	2866.3864	**1433.6968**	2849.3598	1425.1835	2848.3758	1424.6915	Y	**553.2729**	277.1401	536.2463	268.6268	535.2623	268.1348	5
26	2937.4235	1469.2154	2920.3969	1460.7021	2919.4129	1460.2101	A	**390.2096**	195.6084	373.1830	187.0951	372.1990	186.6031	4
27	3024.4555	1512.7314	3007.4290	1504.2181	3006.4449	1503.7261	S	**319.1724**	160.0899	302.1459	151.5766	301.1619	151.0846	3
28	3081.4770	1541.2421	3064.4504	1532.7288	3063.4664	1532.2368	G	**232.1404**	116.5738	215.1139	108.0606			2
29							R	**175.1190**	88.0631	158.0924	79.5498			1

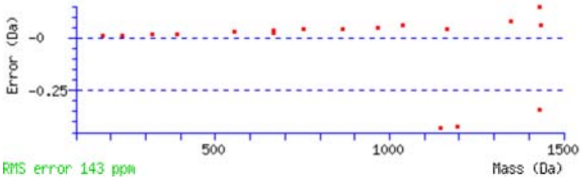

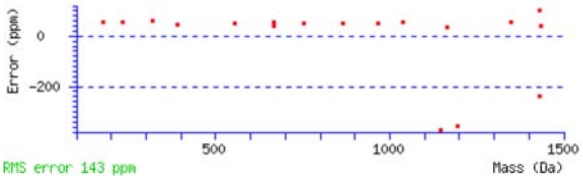

Peptide View

MS/MS Fragmentation of NIAGALAFWMANASELLNFIK
Found in gi|94402335|ref|XP_916103.2|, PREDICTED: similar to Afadin (Af-6 protein) isoform 4 [Mus musculus]

Match to Query 807: 2293.378048 from(1147.696300,2+)

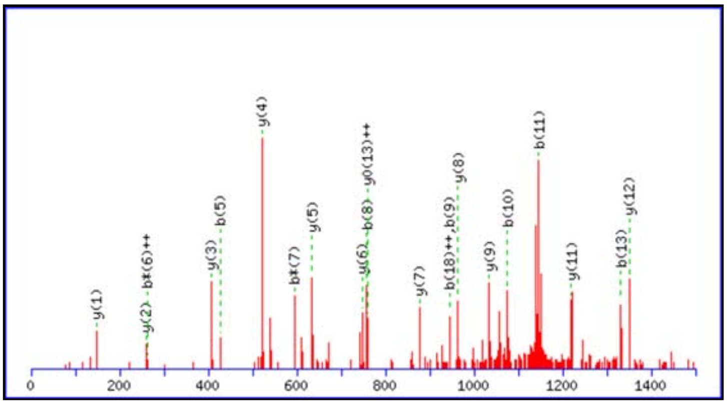

Monoisotopic mass of neutral peptide Mr(calc): 2293.1928
Ions Score: 76 **Expect:** 0.00013
Matches (**Bold Red**): 21/202 fragment ions using 36 most intense peaks

#	b	b^{++}	b*	$b*^{++}$	b^0	b^{0++}	Seq.	y	y^{++}	y*	$y*^{++}$	y^0	y^{0++}	#
1	115.0502	58.0287	98.0237	49.5155			N							21
2	228.1343	114.5708	211.1077	106.0575			I	2180.1572	1090.5822	2163.1307	1082.0690	2162.1466	1081.5770	20
3	299.1714	150.0893	282.1448	141.5761			A	2067.0731	1034.0402	2050.0466	1025.5269	2049.0626	1025.0349	19
4	356.1928	178.6001	339.1663	170.0868			G	1996.0360	998.5217	1979.0095	990.0084	1978.0255	989.5164	18
5	**427.2299**	214.1186	410.2034	205.6053			A	1939.0146	970.0109	1921.9880	961.4976	1921.0040	961.0056	17
6	540.3140	270.6606	523.2875	**262.1474**			L	1867.9775	934.4924	1850.9509	925.9791	1849.9669	925.4871	16
7	611.3511	306.1792	**594.3246**	297.6659			A	1754.8934	877.9503	1737.8669	869.4371	1736.8828	868.9451	15
8	**758.4195**	379.7134	741.3930	371.2001			F	1683.8563	842.4318	1666.8297	833.9185	1665.8457	833.4265	14
9	**944.4988**	472.7531	927.4723	464.2398			W	1536.7879	768.8976	1519.7613	760.3843	1518.7773	**759.8923**	13
10	**1075.5393**	538.2733	1058.5128	529.7600			M	**1350.7086**	675.8579	1333.6820	667.3446	1332.6980	666.8526	12
11	**1146.5764**	573.7919	1129.5499	565.2786			A	**1219.6681**	610.3377	1202.6415	601.8244	1201.6575	601.3324	11
12	1260.6194	630.8133	1243.5928	622.3000			N	1148.6310	574.8191	1131.6044	566.3059	1130.6204	565.8138	10
13	**1331.6565**	666.3319	1314.6299	657.8186			A	**1034.5881**	517.7977	1017.5615	509.2844	1016.5775	508.7924	9
14	1418.6885	709.8479	1401.6619	701.3346	1400.6779	700.8426	S	**963.5509**	482.2791	946.5244	473.7658	945.5404	473.2738	8
15	1547.7311	774.3692	1530.7045	765.8559	1529.7205	765.3639	E	**876.5189**	438.7631	859.4924	430.2498	858.5083	429.7578	7
16	1660.8151	830.9112	1643.7886	822.3979	1642.8046	821.9059	L	**747.4763**	374.2418	730.4498	365.7285			6
17	1773.8992	887.4532	1756.8727	878.9400	1755.8886	878.4480	L	**634.3923**	317.6998	617.3657	309.1865			5
18	1887.9421	**944.4747**	1870.9156	935.9614	1869.9316	935.4694	N	**521.3082**	261.1577	504.2817	252.6445			4
19	2035.0105	1018.0089	2017.9840	1009.4956	2017.0000	1009.0036	F	**407.2653**	204.1363	390.2387	195.6230			3
20	2148.0946	1074.5509	2131.0681	1066.0377	2130.0840	1065.5457	I	**260.1969**	130.6021	243.1703	122.0888			2
21							K	**147.1128**	74.0600	130.0863	65.5468			1

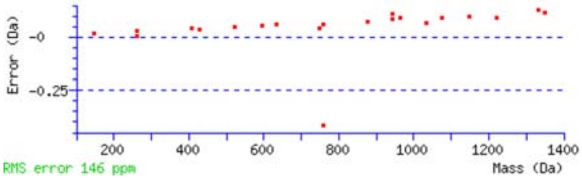

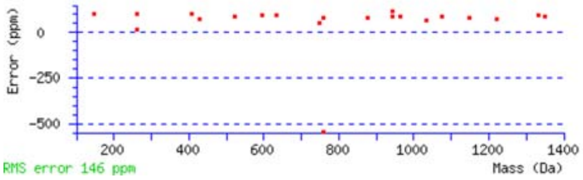

Peptide View

MS/MS Fragmentation of NIILEEGKEILVGDVGQTVDDPYTTFVK
Found in gi|6680924|ref|NP_031713.1|, cofilin 1, non-muscle [Mus musculus]

Match to Query 401: 3091.676172 from(1031.566000,3+)

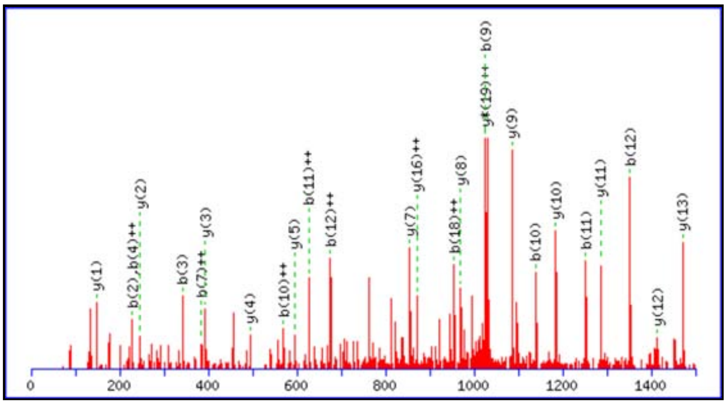

Monoisotopic mass of neutral peptide Mr(calc): 3091.5964
Ions Score: 61 Expect: 0.0078
Matches (**Bold Red**): 26/310 fragment ions using 47 most intense peaks

#	b	b^{++}	b*	b^{*++}	b^{0}	b^{0++}	Seq.	y	y^{++}	y*	y^{*++}	y^{0}	y^{0++}	#
1	115.0502	58.0287	98.0237	49.5155			N							28
2	**228.1343**	114.5708	211.1077	106.0575			I	2978.5608	1489.7840	2961.5342	1481.2708	2960.5502	1480.7788	27
3	**341.2183**	171.1128	324.1918	162.5995			I	2865.4767	1433.2420	2848.4502	1424.7287	2847.4662	1424.2367	26
4	454.3024	**227.6548**	437.2758	219.1416			L	2752.3927	1376.7000	2735.3661	1368.1867	2734.3821	1367.6947	25
5	583.3450	292.1761	566.3184	283.6629	565.3344	283.1708	E	2639.3086	1320.1579	2622.2821	1311.6447	2621.2980	1311.1527	24
6	712.3876	356.6974	695.3610	348.1841	694.3770	347.6921	E	2510.2660	1255.6366	2493.2395	1247.1234	2492.2555	1246.6314	23
7	769.4090	**385.2082**	752.3825	376.6949	751.3985	376.2029	G	2381.2234	1191.1154	2364.1969	1182.6021	2363.2129	1182.1101	22
8	897.5040	449.2556	880.4774	440.7424	879.4934	440.2503	K	2324.2020	1162.6046	2307.1754	1154.0913	2306.1914	1153.5993	21
9	**1026.5466**	513.7769	1009.5200	505.2637	1008.5360	504.7716	E	2196.1070	1098.5571	2179.0805	1090.0439	2178.0964	1089.5519	20
10	**1139.6306**	**570.3190**	1122.6041	561.8057	1121.6201	561.3137	I	2067.0644	1034.0358	2050.0379	**1025.5226**	2049.0539	1025.0306	19
11	**1252.7147**	**626.8610**	1235.6881	618.3477	1234.7041	617.8557	L	1953.9804	977.4938	1936.9538	968.9805	1935.9698	968.4885	18
12	**1351.7831**	**676.3952**	1334.7566	667.8819	1333.7725	667.3899	V	1840.8963	920.9518	1823.8698	912.4385	1822.8857	911.9465	17
13	1408.8046	704.9059	1391.7780	696.3926	1390.7940	695.9006	G	1741.8279	**871.4176**	1724.8013	862.9043	1723.8173	862.4123	16
14	1523.8315	762.4194	1506.8050	753.9061	1505.8209	753.4141	D	1684.8064	842.9069	1667.7799	834.3936	1666.7959	833.9016	15
15	1622.8999	811.9536	1605.8734	803.4403	1604.8894	802.9483	V	1569.7795	785.3934	1552.7529	776.8801	1551.7689	776.3881	14
16	1679.9214	840.4643	1662.8948	831.9511	1661.9108	831.4590	G	**1470.7111**	735.8592	1453.6845	727.3459	1452.7005	726.8539	13
17	1807.9800	904.4936	1790.9534	895.9803	1789.9694	895.4883	Q	**1413.6896**	707.3484	1396.6631	698.8352	1395.6791	698.3432	12
18	1909.0276	**955.0175**	1892.0011	946.5042	1891.0171	946.0122	T	**1285.6310**	643.3192	1268.6045	634.8059	1267.6205	634.3139	11
19	2008.0960	1004.5517	1991.0695	996.0384	1990.0855	995.5464	V	**1184.5834**	592.7953	1167.5568	584.2820	1166.5728	583.7900	10
20	2123.1230	1062.0651	2106.0964	1053.5519	2105.1124	1053.0598	D	**1085.5150**	543.2611	1068.4884	534.7478	1067.5044	534.2558	9
21	2238.1499	1119.5786	2221.1234	1111.0653	2220.1394	1110.5733	D	**970.4880**	485.7476	953.4615	477.2344	952.4774	476.7424	8
22	2335.2027	1168.1050	2318.1761	1159.5917	2317.1921	1159.0997	P	**855.4611**	428.2342	838.4345	419.7209	837.4505	419.2289	7
23	2498.2660	1249.6366	2481.2395	1241.1234	2480.2555	1240.6314	Y	758.4083	379.7078	741.3818	371.1945	740.3977	370.7025	6
24	2599.3137	1300.1605	2582.2871	1291.6472	2581.3031	1291.1552	T	**595.3450**	298.1761	578.3184	289.6629	577.3344	289.1708	5
25	2700.3614	1350.6843	2683.3348	1342.1711	2682.3508	1341.6790	T	**494.2973**	247.6523	477.2708	239.1390	476.2867	238.6470	4
26	2847.4298	1424.2185	2830.4032	1415.7053	2829.4192	1415.2132	F	**393.2496**	197.1284	376.2231	188.6152			3
27	2946.4982	1473.7527	2929.4716	1465.2395	2928.4876	1464.7475	V	**246.1812**	123.5942	229.1547	115.0810			2
28							K	**147.1128**	74.0600	130.0863	65.5468			1

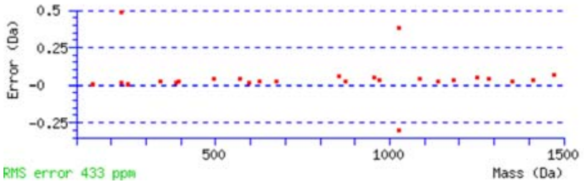

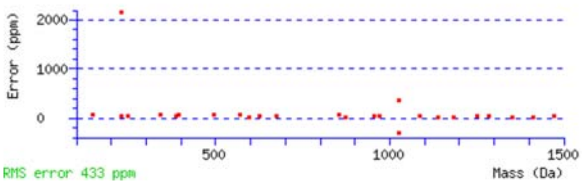

Peptide View

MS/MS Fragmentation of **NQVAMNPTNTVFDAK**
Found in **gi|31981690|ref|NP_112442.2|**, heat shock protein 8 [Mus musculus]

Match to Query 378: 1648.860248 from(825.437400,2+)

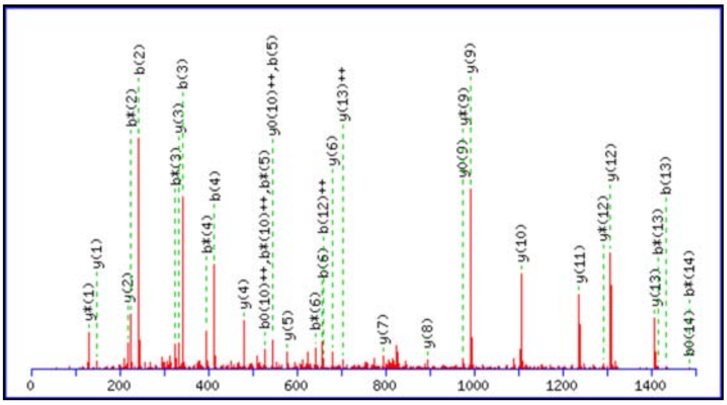

Monoisotopic mass of neutral peptide Mr(calc): 1648.7879
Ions Score: 86 **Expect:** 5.2e-006
Matches (**Bold Red**): 36/150 fragment ions using 74 most intense peaks

#	b	b^{++}	b*	b^{*++}	b^0	b^{0++}	Seq.	y	y^{++}	y*	y^{*++}	y^0	y^{0++}	#
1	115.0502	58.0287	98.0237	49.5155			N							15
2	**243.1088**	122.0580	**226.0822**	113.5448			Q	1535.7522	768.3798	1518.7257	759.8665	1517.7417	759.3745	14
3	**342.1772**	171.5922	**325.1506**	163.0790			V	**1407.6937**	**704.3505**	1390.6671	695.8372	1389.6831	695.3452	13
4	**413.2143**	207.1108	**396.1878**	198.5975			A	**1308.6252**	654.8163	**1291.5987**	646.3030	1290.6147	645.8110	12
5	**544.2548**	272.6310	**527.2282**	264.1178			M	**1237.5881**	619.2977	1220.5616	610.7844	1219.5776	610.2924	11
6	**658.2977**	329.6525	**641.2712**	321.1392			N	**1106.5477**	553.7775	1089.5211	545.2642	1088.5371	**544.7722**	10
7	755.3505	378.1789	738.3239	369.6656			P	**992.5047**	496.7560	**975.4782**	488.2427	**974.4942**	487.7507	9
8	856.3982	428.7027	839.3716	420.1894	838.3876	419.6974	T	**895.4520**	448.2296	878.4254	439.7163	877.4414	439.2243	8
9	970.4411	485.7242	953.4145	477.2109	952.4305	476.7189	N	**794.4043**	397.7058	777.3777	389.1925	776.3937	388.7005	7
10	1071.4888	536.2480	1054.4622	**527.7347**	1053.4782	**527.2427**	T	**680.3614**	340.6843	663.3348	332.1710	662.3508	331.6790	6
11	1170.5572	585.7822	1153.5306	577.2690	1152.5466	576.7769	V	**579.3137**	290.1605	562.2871	281.6472	561.3031	281.1552	5
12	1317.6256	**659.3164**	1300.5990	650.8032	1299.6150	650.3111	F	**480.2453**	240.6263	463.2187	232.1130	462.2347	231.6210	4
13	**1432.6525**	716.8299	**1415.6260**	708.3166	1414.6420	707.8246	D	**333.1769**	167.0921	316.1503	158.5788	315.1663	158.0868	3
14	1503.6896	752.3485	**1486.6631**	743.8352	**1485.6791**	743.3432	A	**218.1499**	109.5786	201.1234	101.0653			2
15							K	**147.1128**	74.0600	**130.0863**	65.5468			1

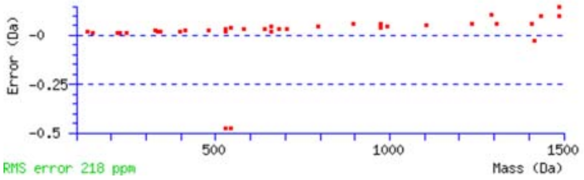

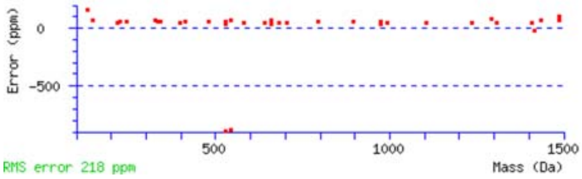

Peptide View

MS/MS Fragmentation of QAGGGAGPPNPSLNGSAPR
Found in gi|63723564|ref|XP_484699.2|, PREDICTED: similar to src homology 2 domain-containing transforming protein C [Mus musculus]

Match to Query 633: 1703.928048 from(852.971300,2+)

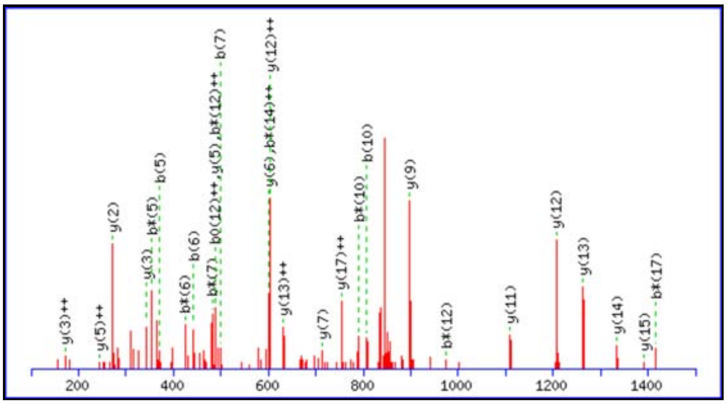

Monoisotopic mass of neutral peptide Mr(calc): 1703.8339
Ions Score: 56 Expect: 0.006
Matches (Bold Red): 29/188 fragment ions using 68 most intense peaks

#	b	b^{++}	b*	b^{*++}	b^0	b^{0++}	Seq.	y	y^{++}	y*	y^{*++}	y^0	y^{0++}	#
1	129.0659	65.0366	112.0393	56.5233			Q							19
2	200.1030	100.5551	183.0764	92.0418			A	1576.7826	788.8949	1559.7561	780.3817	1558.7720	779.8897	18
3	257.1244	129.0659	240.0979	120.5526			G	1505.7455	**753.3764**	1488.7189	744.8631	1487.7349	744.3711	17
4	314.1459	157.5766	297.1193	149.0633			G	1448.7240	724.8657	1431.6975	716.3524	1430.7135	715.8604	16
5	**371.1673**	186.0873	**354.1408**	177.5740			G	**1391.7026**	696.3549	1374.6760	687.8416	1373.6920	687.3496	15
6	**442.2045**	221.6059	**425.1779**	213.0926			A	**1334.6811**	667.8442	1317.6546	659.3309	1316.6705	658.8389	14
7	**499.2259**	250.1166	**482.1994**	241.6033			G	**1263.6440**	**632.3256**	1246.6175	623.8124	1245.6334	623.3204	13
8	596.2787	298.6430	579.2521	290.1297			P	**1206.6225**	**603.8149**	1189.5960	595.3016	1188.6120	594.8096	12
9	693.3314	347.1694	676.3049	338.6561			P	**1109.5698**	555.2885	1092.5432	546.7753	1091.5592	546.2832	11
10	**807.3744**	404.1908	**790.3478**	395.6775			N	1012.5170	506.7621	995.4905	498.2489	994.5065	497.7569	10
11	904.4271	452.7172	887.4006	444.2039			P	**898.4741**	449.7407	881.4475	441.2274	880.4635	440.7354	9
12	991.4592	496.2332	**974.4326**	**487.7199**	973.4486	**487.2279**	S	801.4213	401.2143	784.3948	392.7010	783.4108	392.2090	8
13	1104.5432	552.7752	1087.5167	544.2620	1086.5327	543.7700	L	**714.3893**	357.6983	697.3628	349.1850	696.3787	348.6930	7
14	1218.5861	609.7967	1201.5596	**601.2834**	1200.5756	600.7914	N	**601.3052**	301.1563	584.2787	292.6430	583.2947	292.1510	6
15	1275.6076	638.3074	1258.5811	629.7942	1257.5970	629.3022	G	**487.2623**	**244.1348**	470.2358	235.6215	469.2517	235.1295	5
16	1362.6396	681.8235	1345.6131	673.3102	1344.6291	672.8182	S	430.2409	215.6241	413.2143	207.1108	412.2303	206.6188	4
17	1433.6767	717.3420	**1416.6502**	708.8287	1415.6662	708.3367	A	**343.2088**	**172.1080**	326.1823	163.5948			3
18	1530.7295	765.8684	1513.7030	757.3551	1512.7189	756.8631	P	**272.1717**	136.5895	255.1452	128.0762			2
19							R	175.1190	88.0631	158.0924	79.5498			1

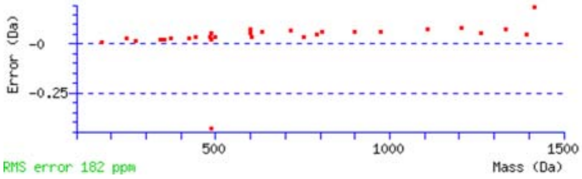

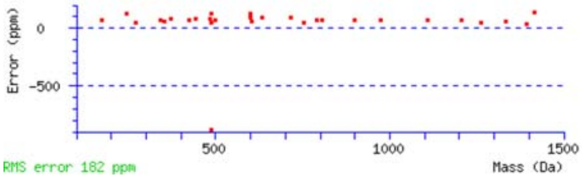

Peptide View

MS/MS Fragmentation of **QPVYIIMELVPGGDFLTFLR**
Found in **gi|6679773|ref|NP_032026.1|**, fer (fms/fps related) protein kinase, testis specific 2 isoform b [Mus musculus]

Match to Query 740: 2313.282648 from(1157.648600,2+)

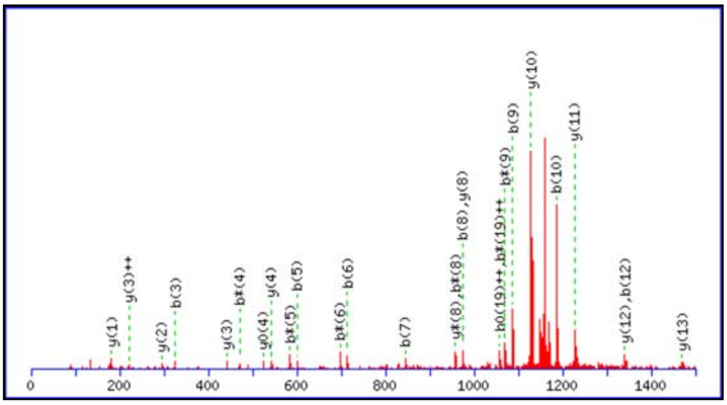

Monoisotopic mass of neutral peptide Mr(calc): 2313.2538
Variable modifications:
R20 : Arginine-13C6 (R-13C6)
Ions Score: 53 Expect: 0.033
Matches (**Bold Red**): 27/208 fragment ions using 45 most intense peaks

#	b	b++	b*	b*++	b0	b0++	Seq.	y	y++	y*	y*++	y0	y0++	#
1	129.0659	65.0366	112.0393	56.5233			Q							20
2	226.1186	113.5629	209.0921	105.0497			P	2186.2025	1093.6049	2169.1759	1085.0916	2168.1919	1084.5996	19
3	**325.1870**	163.0972	308.1605	154.5839			V	2089.1497	1045.0785	2072.1232	1036.5652	2071.1392	1036.0732	18
4	488.2504	244.6288	**471.2238**	236.1155			Y	1990.0813	995.5443	1973.0548	987.0310	1972.0707	986.5390	17
5	**601.3344**	301.1708	**584.3079**	292.6576			I	1827.0180	914.0126	1809.9914	905.4994	1809.0074	905.0073	16
6	**714.4185**	357.7129	**697.3919**	349.1996			I	1713.9339	857.4706	1696.9074	848.9573	1695.9234	848.4653	15
7	**845.4590**	423.2331	828.4324	414.7198			M	1600.8499	800.9286	1583.8233	792.4153	1582.8393	791.9233	14
8	**974.5015**	487.7544	**957.4750**	479.2411	956.4910	478.7491	E	**1469.8094**	735.4083	1452.7828	726.8951	1451.7988	726.4030	13
9	**1087.5856**	544.2964	**1070.5591**	535.7832	1069.5750	535.2912	L	**1340.7668**	670.8870	1323.7402	662.3738	1322.7562	661.8817	12
10	**1186.6540**	593.8306	1169.6275	585.3174	1168.6435	584.8254	V	**1227.6827**	614.3450	1210.6562	605.8317	1209.6722	605.3397	11
11	1283.7068	642.3570	1266.6802	633.8438	1265.6962	633.3517	P	**1128.6143**	564.8108	1111.5878	556.2975	1110.6038	555.8055	10
12	**1340.7282**	670.8678	1323.7017	662.3545	1322.7177	661.8625	G	1031.5616	516.2844	1014.5350	507.7711	1013.5510	507.2791	9
13	1397.7497	699.3785	1380.7231	690.8652	1379.7391	690.3732	G	**974.5401**	487.7737	**957.5135**	479.2604	956.5295	478.7684	8
14	1512.7766	756.8920	1495.7501	748.3787	1494.7661	747.8867	D	917.5186	459.2630	900.4921	450.7497	899.5081	450.2577	7
15	1659.8450	830.4262	1642.8185	821.9129	1641.8345	821.4209	F	802.4917	401.7495	785.4651	393.2362	784.4811	392.7442	6
16	1772.9291	886.9682	1755.9026	878.4549	1754.9185	877.9629	L	655.4233	328.2153	638.3967	319.7020	637.4127	319.2100	5
17	1873.9768	937.4920	1856.9502	928.9788	1855.9662	928.4867	T	**542.3392**	271.6733	525.3127	263.1600	**524.3287**	262.6680	4
18	2021.0452	1011.0262	2004.0186	1002.5130	2003.0346	1002.0210	F	**441.2915**	**221.1494**	424.2650	212.6361			3
19	2134.1293	1067.5683	2117.1027	**1059.0550**	2116.1187	**1058.5630**	L	**294.2231**	147.6152	277.1966	139.1019			2
20							R	**181.1391**	91.0732	164.1125	82.5599			1

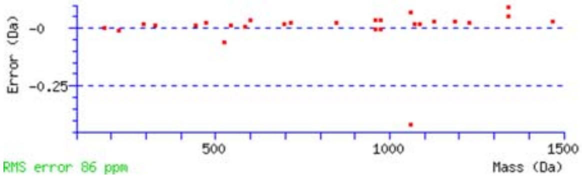

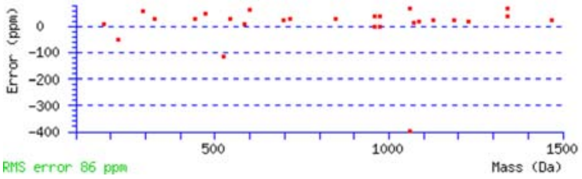

Peptide View

MS/MS Fragmentation of SDVWSFGILLTELTTK
Found in gi|70794809|ref|NP_001020566.1|, Rous sarcoma oncogene isoform 2 [Mus musculus]

Match to Query 678: 1809.103448 from(905.559000,2+)

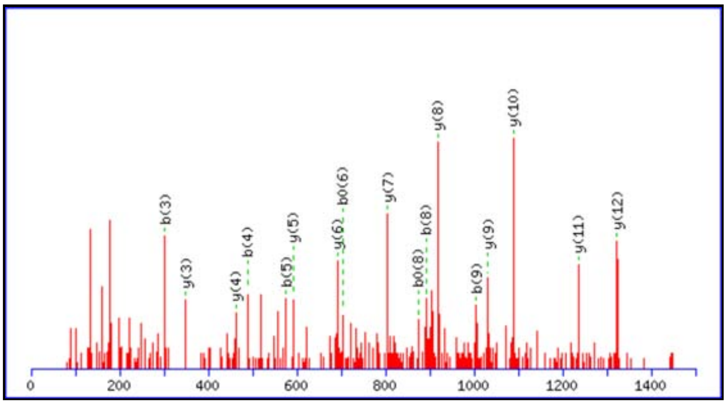

Monoisotopic mass of neutral peptide Mr(calc): 1808.9560
Ions Score: 62 **Expect:** 0.0026
Matches (**Bold Red**): 17/148 fragment ions using 35 most intense peaks

#	b	b^{++}	b^0	b^{0++}	Seq.	y	y^{++}	y*	y^{*++}	y^0	y^{0++}	#
1	88.0393	44.5233	70.0287	35.5180	S							16
2	203.0662	102.0368	185.0557	93.0315	D	1722.9312	861.9693	1705.9047	853.4560	1704.9207	852.9640	15
3	**302.1347**	151.5710	284.1241	142.5657	V	1607.9043	804.4558	1590.8777	795.9425	1589.8937	795.4505	14
4	**488.2140**	244.6106	470.2034	235.6053	W	1508.8359	754.9216	1491.8093	746.4083	1490.8253	745.9163	13
5	**575.2460**	288.1266	557.2354	279.1214	S	**1322.7566**	661.8819	1305.7300	653.3686	1304.7460	652.8766	12
6	722.3144	361.6608	**704.3038**	352.6556	F	**1235.7245**	618.3659	1218.6980	609.8526	1217.7140	609.3606	11
7	779.3359	390.1716	761.3253	381.1663	G	**1088.6561**	544.8317	1071.6296	536.3184	1070.6456	535.8264	10
8	**892.4199**	446.7136	**874.4094**	437.7083	I	**1031.6347**	516.3210	1014.6081	507.8077	1013.6241	507.3157	9
9	**1005.5040**	503.2556	987.4934	494.2503	L	**918.5506**	459.7789	901.5241	451.2657	900.5400	450.7737	8
10	1118.5880	559.7977	1100.5775	550.7924	L	**805.4666**	403.2369	788.4400	394.7236	787.4560	394.2316	7
11	1219.6357	610.3215	1201.6252	601.3162	T	**692.3825**	346.6949	675.3559	338.1816	674.3719	337.6896	6
12	1348.6783	674.8428	1330.6678	665.8375	E	**591.3348**	296.1710	574.3083	287.6578	573.3242	287.1658	5
13	1461.7624	731.3848	1443.7518	722.3795	L	**462.2922**	231.6497	445.2657	223.1365	444.2817	222.6445	4
14	1562.8101	781.9087	1544.7995	772.9034	T	**349.2082**	175.1077	332.1816	166.5944	331.1976	166.1024	3
15	1663.8577	832.4325	1645.8472	823.4272	T	248.1605	124.5839	231.1339	116.0706	230.1499	115.5786	2
16					K	147.1128	74.0600	130.0863	65.5468			1

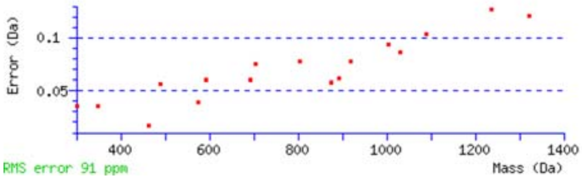

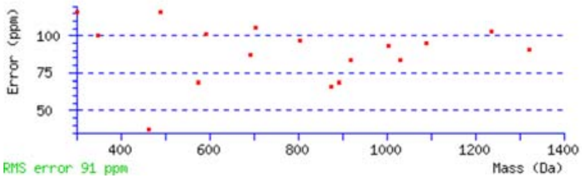

Peptide View

MS/MS Fragmentation of **SDVWSFGILLTELVTK**
Found in **gi|6679879|ref|NP_032080.1|**, protein-tyrosine kinase fyn [Mus musculus]

Match to Query 676: 1807.136848 from(904.575700,2+)

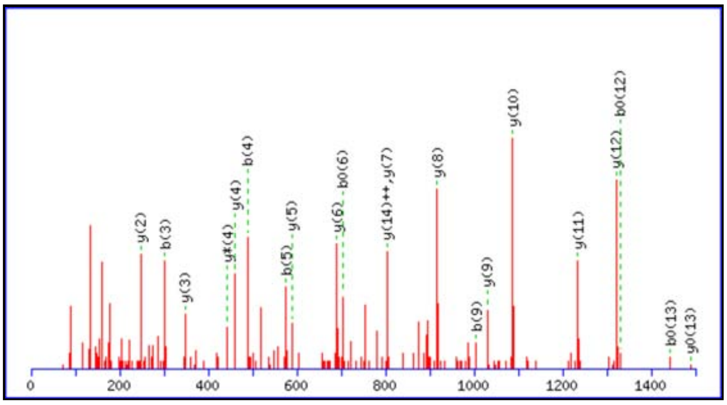

Monoisotopic mass of neutral peptide Mr(calc): 1806.9767
Ions Score: 84 **Expect:** 1.4e-005
Matches (**Bold Red**): 21/148 fragment ions using 32 most intense peaks

#	b	b^{++}	b^{0}	b^{0++}	Seq.	y	y^{++}	y*	y^{*++}	y^{0}	y^{0++}	#
1	88.0393	44.5233	70.0287	35.5180	S							16
2	203.0662	102.0368	185.0557	93.0315	D	1720.9520	860.9796	1703.9254	852.4663	1702.9414	851.9743	15
3	**302.1347**	151.5710	284.1241	142.5657	V	1605.9250	**803.4661**	1588.8985	794.9529	1587.9145	794.4609	14
4	**488.2140**	244.6106	470.2034	235.6053	W	1506.8566	753.9319	1489.8301	745.4187	**1488.8460**	744.9267	13
5	**575.2460**	288.1266	557.2354	279.1214	S	**1320.7773**	660.8923	1303.7508	652.3790	1302.7667	651.8870	12
6	722.3144	361.6608	**704.3038**	352.6556	F	**1233.7453**	617.3763	1216.7187	608.8630	1215.7347	608.3710	11
7	779.3359	390.1716	761.3253	381.1663	G	**1086.6769**	543.8421	1069.6503	535.3288	1068.6663	534.8368	10
8	892.4199	446.7136	874.4094	437.7083	I	**1029.6554**	515.3313	1012.6289	506.8181	1011.6448	506.3261	9
9	**1005.5040**	503.2556	987.4934	494.2503	L	**916.5713**	458.7893	899.5448	450.2760	898.5608	449.7840	8
10	1118.5880	559.7977	1100.5775	550.7924	L	**803.4873**	402.2473	786.4607	393.7340	785.4767	393.2420	7
11	1219.6357	610.3215	1201.6252	601.3162	T	**690.4032**	345.7052	673.3767	337.1920	672.3927	336.7000	6
12	1348.6783	674.8428	**1330.6678**	665.8375	E	**589.3555**	295.1814	572.3290	286.6681	571.3450	286.1761	5
13	1461.7624	731.3848	**1443.7518**	722.3795	L	**460.3130**	230.6601	**443.2864**	222.1468	442.3024	221.6548	4
14	1560.8308	780.9190	1542.8202	771.9137	V	**347.2289**	174.1181	330.2023	165.6048	329.2183	165.1128	3
15	1661.8785	831.4429	1643.8679	822.4376	T	**248.1605**	124.5839	231.1339	116.0706	230.1499	115.5786	2
16					K	147.1128	74.0600	130.0863	65.5468			1

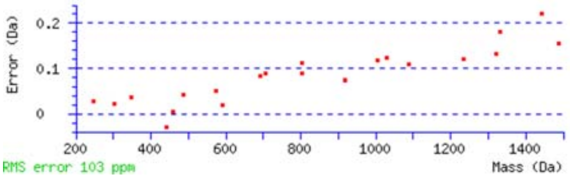

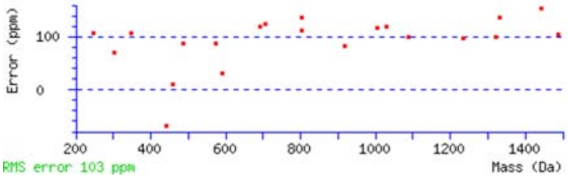

Peptide View

MS/MS Fragmentation of SSSLEGFHSQYK
Found in gi|31542871|ref|NP_067331.2|, growth factor receptor bound protein 2-associated protein 1 [Mus musculus]

Match to Query 388: 1368.650448 from(685.332500,2+)

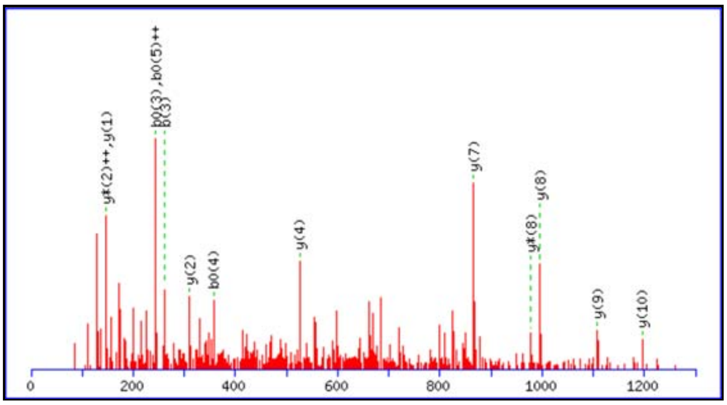

Monoisotopic mass of neutral peptide Mr(calc): 1368.6310
Ions Score: 51 Expect: 0.012
Matches (**Bold Red**): 13/108 fragment ions using 20 most intense peaks

#	b	b^{++}	b*	b^{*++}	b^0	b^{0++}	Seq.	y	y^{++}	y*	y^{*++}	y^0	y^{0++}	#
1	88.0393	44.5233			70.0287	35.5180	S							12
2	175.0713	88.0393			157.0608	79.0340	S	1282.6062	641.8068	1265.5797	633.2935	1264.5957	632.8015	11
3	**262.1034**	131.5553			**244.0928**	122.5500	S	**1195.5742**	598.2907	1178.5477	589.7775	1177.5636	589.2855	10
4	375.1874	188.0974			**357.1769**	179.0921	L	**1108.5422**	554.7747	1091.5156	546.2614	1090.5316	545.7694	9
5	504.2300	252.6186			486.2195	**243.6134**	E	**995.4581**	498.2327	**978.4316**	489.7194	977.4475	489.2274	8
6	561.2515	281.1294			543.2409	272.1241	G	**866.4155**	433.7114	849.3890	425.1981	848.4050	424.7061	7
7	708.3199	354.6636			690.3093	345.6583	F	809.3941	405.2007	792.3675	396.6874	791.3835	396.1954	6
8	845.3788	423.1930			827.3682	414.1878	H	662.3257	331.6665	645.2991	323.1532	644.3151	322.6612	5
9	932.4108	466.7091			914.4003	457.7038	S	**525.2667**	263.1370	508.2402	254.6237	507.2562	254.1317	4
10	1060.4694	530.7383	1043.4429	522.2251	1042.4588	521.7331	Q	438.2347	219.6210	421.2082	211.1077			3
11	1223.5327	612.2700	1206.5062	603.7567	1205.5222	603.2647	Y	**310.1761**	155.5917	293.1496	**147.0784**			2
12							K	**147.1128**	74.0600	130.0863	65.5468			1

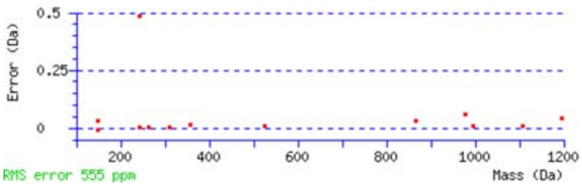

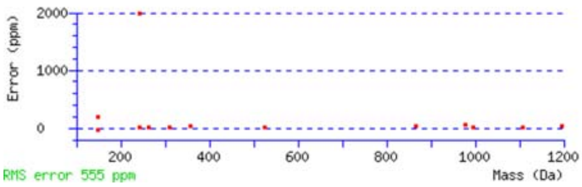

Peptide View

MS/MS Fragmentation of **TAAFLLPILSQIYADGPGEALR**
Found in **gi|6753620|ref|NP_034158.1|**, DEAD/H (Asp-Glu-Ala-Asp/His) box polypeptide 3, X-linked [Mus musculus]

Match to Query 812: 2315.440048 from(1158.727300,2+)

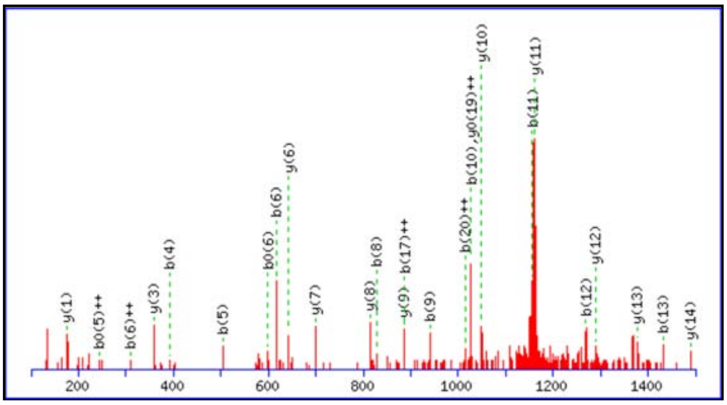

Monoisotopic mass of neutral peptide Mr(calc): 2315.2525
Ions Score: 64 Expect: 0.002
Matches (**Bold Red**): 26/226 fragment ions using 64 most intense peaks

#	b	b^{++}	b*	b^{*++}	b^{0}	b^{0++}	Seq.	y	y^{++}	y*	y^{*++}	y^{0}	y^{0++}	#
1	102.0550	51.5311			84.0444	42.5258	T							22
2	173.0921	87.0497			155.0815	78.0444	A	2215.2121	1108.1097	2198.1855	1099.5964	2197.2015	1099.1044	21
3	244.1292	122.5682			226.1186	113.5629	A	2144.1750	1072.5911	2127.1484	1064.0778	2126.1644	1063.5858	20
4	**391.1976**	196.1024			373.1870	187.0971	F	2073.1379	1037.0726	2056.1113	1028.5593	2055.1273	**1028.0673**	19
5	**504.2816**	252.6445			486.2711	**243.6392**	L	1926.0694	963.5384	1909.0429	955.0251	1908.0589	954.5331	18
6	**617.3657**	**309.1865**			**599.3551**	300.1812	L	1812.9854	906.9963	1795.9588	898.4831	1794.9748	897.9910	17
7	714.4185	357.7129			696.4079	348.7076	P	1699.9013	850.4543	1682.8748	841.9410	1681.8908	841.4490	16
8	**827.5025**	414.2549			809.4920	405.2496	I	1602.8486	801.9279	1585.8220	793.4146	1584.8380	792.9226	15
9	**940.5866**	470.7969			922.5760	461.7916	L	**1489.7645**	745.3859	1472.7380	736.8726	1471.7539	736.3806	14
10	**1027.6186**	514.3129			1009.6081	505.3077	S	**1376.6804**	688.8439	1359.6539	680.3306	1358.6699	679.8386	13
11	**1155.6772**	578.3422	1138.6506	569.8290	1137.6666	569.3370	Q	**1289.6484**	645.3278	1272.6219	636.8146	1271.6378	636.3226	12
12	**1268.7613**	634.8843	1251.7347	626.3710	1250.7507	625.8790	I	**1161.5898**	581.2986	1144.5633	572.7853	1143.5793	572.2933	11
13	**1431.8246**	716.4159	1414.7980	707.9027	1413.8140	707.4106	Y	**1048.5058**	524.7565	1031.4792	516.2432	1030.4952	515.7512	10
14	1502.8617	751.9345	1485.8351	743.4212	1484.8511	742.9292	A	**885.4424**	443.2249	868.4159	434.7116	867.4319	434.2196	9
15	1617.8886	809.4480	1600.8621	800.9347	1599.8781	800.4427	D	**814.4053**	407.7063	797.3788	399.1930	796.3948	398.7010	8
16	1674.9101	837.9587	1657.8835	829.4454	1656.8995	828.9534	G	**699.3784**	350.1928	682.3518	341.6796	681.3678	341.1876	7
17	1771.9629	**886.4851**	1754.9363	877.9718	1753.9523	877.4798	P	**642.3569**	321.6821	625.3304	313.1688	624.3464	312.6768	6
18	1828.9843	914.9958	1811.9578	906.4825	1810.9738	905.9905	G	545.3042	273.1557	528.2776	264.6424	527.2936	264.1504	5
19	1958.0269	979.5171	1941.0004	971.0038	1940.0163	970.5118	E	488.2827	244.6450	471.2562	236.1317	470.2721	235.6397	4
20	2029.0640	**1015.0356**	2012.0375	1006.5224	2011.0535	1006.0304	A	**359.2401**	180.1237	342.2136	171.6104			3
21	2142.1481	1071.5777	2125.1215	1063.0644	2124.1375	1062.5724	L	288.2030	144.6051	271.1765	136.0919			2
22							R	**175.1190**	88.0631	158.0924	79.5498			1

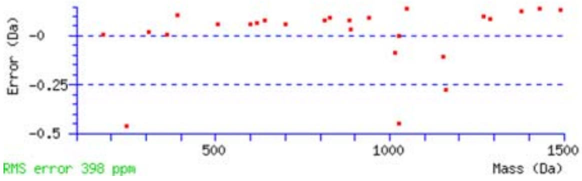

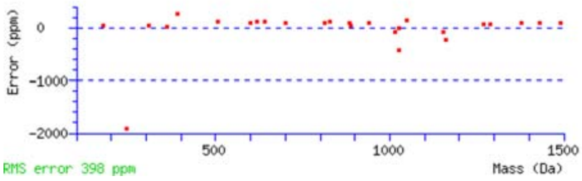

Peptide View

MS/MS Fragmentation of TAIEAFNETIK
Found in gi|33468921|ref|NP_035215.1|, phosphatidylinositol 3-kinase, regulatory subunit, polypeptide 1 isoform b [Mus musculus]

Match to Query 447: 1235.699248 from(618.856900,2+)

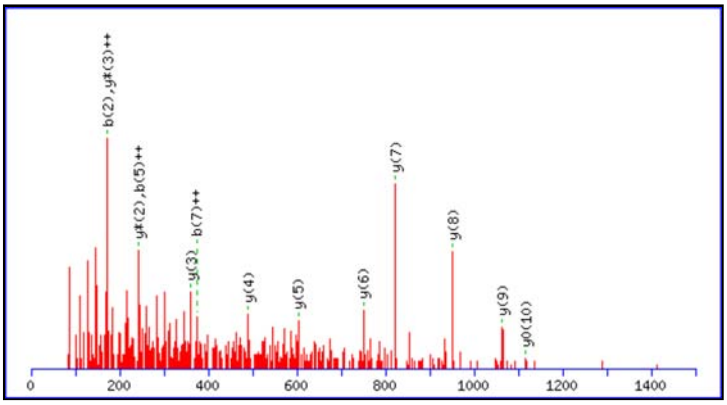

Monoisotopic mass of neutral peptide Mr(calc): 1235.6397
Ions Score: 64 **Expect:** 0.00046
Matches (**Bold Red**): 13/104 fragment ions using 15 most intense peaks

#	b	b^{++}	b*	$b*^{++}$	b^0	b^{0++}	Seq.	y	y^{++}	y*	$y*^{++}$	y^0	y^{0++}	#
1	102.0550	51.5311			84.0444	42.5258	T							11
2	**173.0921**	87.0497			155.0815	78.0444	A	1135.5993	568.3033	1118.5728	559.7900	**1117.5888**	559.2980	10
3	286.1761	143.5917			268.1656	134.5864	I	**1064.5622**	532.7848	1047.5357	524.2715	1046.5517	523.7795	9
4	415.2187	208.1130			397.2082	199.1077	E	**951.4782**	476.2427	934.4516	467.7294	933.4676	467.2374	8
5	486.2558	**243.6316**			468.2453	234.6263	A	**822.4356**	411.7214	805.4090	403.2082	804.4250	402.7161	7
6	633.3242	317.1658			615.3137	308.1605	F	**751.3985**	376.2029	734.3719	367.6896	733.3879	367.1976	6
7	747.3672	**374.1872**	730.3406	365.6739	729.3566	365.1819	N	**604.3301**	302.6687	587.3035	294.1554	586.3195	293.6634	5
8	876.4098	438.7085	859.3832	430.1952	858.3992	429.7032	E	**490.2871**	245.6472	473.2606	237.1339	472.2766	236.6419	4
9	977.4574	489.2324	960.4309	480.7191	959.4469	480.2271	T	**361.2445**	181.1259	344.2180	**172.6126**	343.2340	172.1206	3
10	1090.5415	545.7744	1073.5149	537.2611	1072.5309	536.7691	I	260.1969	130.6021	**243.1703**	122.0888			2
11							K	147.1128	74.0600	130.0863	65.5468			1

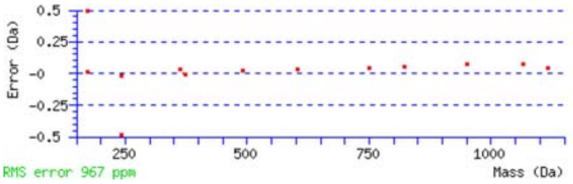

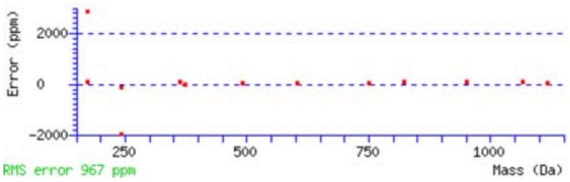

Peptide View

MS/MS Fragmentation of **TEFLSFMNTELAAFTK**
Found in **gi|21886811|ref|NP_058020.1|**, S100 calcium binding protein A11 (calizzarin) [Mus musculus]

Match to Query 605: 1848.976448 from(925.495500,2+)

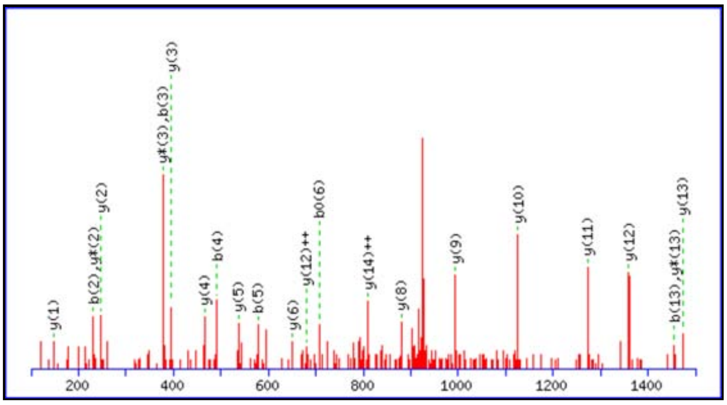

Monoisotopic mass of neutral peptide Mr(calc): 1848.8967
Ions Score: 74 **Expect:** 0.00012
Matches (**Bold Red**): 23/164 fragment ions using 45 most intense peaks

#	b	b^{++}	b*	b^{*++}	b^0	b^{0++}	Seq.	y	y^{++}	y*	y^{*++}	y^0	y^{0++}	#
1	102.0550	51.5311			84.0444	42.5258	T							16
2	**231.0975**	116.0524			213.0870	107.0471	E	1748.8564	874.9318	1731.8298	866.4185	1730.8458	865.9265	15
3	**378.1660**	189.5866			360.1554	180.5813	F	1619.8138	**810.4105**	1602.7872	801.8972	1601.8032	801.4052	14
4	**491.2500**	246.1286			473.2395	237.1234	L	**1472.7454**	736.8763	**1455.7188**	728.3630	1454.7348	727.8710	13
5	**578.2820**	289.6447			560.2715	280.6394	S	**1359.6613**	**680.3343**	1342.6347	671.8210	1341.6507	671.3290	12
6	725.3505	363.1789			**707.3399**	354.1736	F	**1272.6293**	636.8183	1255.6027	628.3050	1254.6187	627.8130	11
7	856.3909	428.6991			838.3804	419.6938	M	**1125.5609**	563.2841	1108.5343	554.7708	1107.5503	554.2788	10
8	970.4339	485.7206	953.4073	477.2073	952.4233	476.7153	N	**994.5204**	497.7638	977.4938	489.2505	976.5098	488.7585	9
9	1071.4815	536.2444	1054.4550	527.7311	1053.4710	527.2391	T	**880.4774**	440.7424	863.4509	432.2291	862.4669	431.7371	8
10	1200.5241	600.7657	1183.4976	592.2524	1182.5136	591.7604	E	779.4298	390.2185	762.4032	381.7052	761.4192	381.2132	7
11	1313.6082	657.3077	1296.5816	648.7945	1295.5976	648.3025	L	**650.3872**	325.6972	633.3606	317.1839	632.3766	316.6919	6
12	1384.6453	692.8263	1367.6188	684.3130	1366.6347	683.8210	A	**537.3031**	269.1552	520.2766	260.6419	519.2925	260.1499	5
13	**1455.6824**	728.3448	1438.6559	719.8316	1437.6719	719.3396	A	**466.2660**	233.6366	449.2395	225.1234	448.2554	224.6314	4
14	1602.7508	801.8791	1585.7243	793.3658	1584.7403	792.8738	F	**395.2289**	198.1181	**378.2023**	189.6048	377.2183	189.1128	3
15	1703.7985	852.4029	1686.7720	843.8896	1685.7879	843.3976	T	**248.1605**	124.5839	**231.1339**	116.0706	230.1499	115.5786	2
16							K	**147.1128**	74.0600	130.0863	65.5468			1

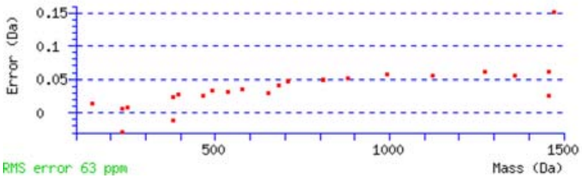

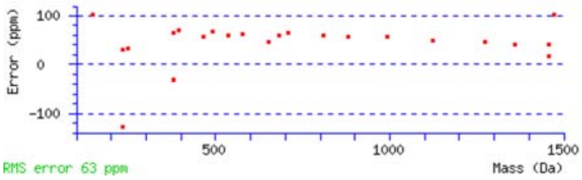

Peptide View

MS/MS Fragmentation of TGTFIVIDILIDIIR
Found in gi|6755228|ref|NP_035332.1|, protein tyrosine phosphatase, non-receptor type 11 [Mus musculus]

Match to Query 561: 1701.081648 from(851.548100,2+)

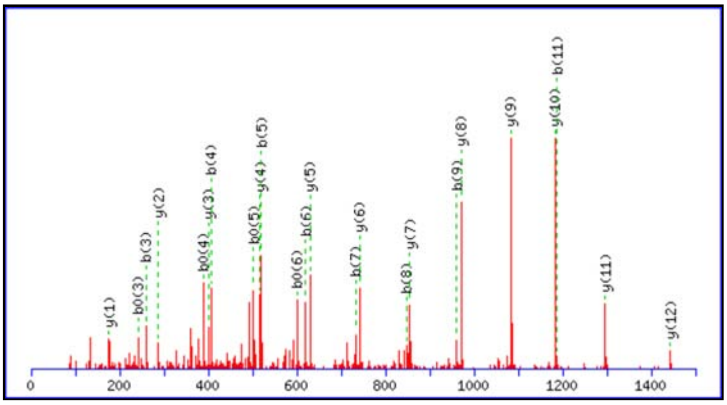

Monoisotopic mass of neutral peptide Mr(calc): 1701.0076
Ions Score: 85 **Expect:** 1.2e-005
Matches (**Bold Red**): 24/134 fragment ions using 44 most intense peaks

#	b	b^{++}	b^0	b^{0++}	Seq.	y	y^{++}	y*	y^{*++}	y^0	y^{0++}	#
1	102.0550	51.5311	84.0444	42.5258	T							15
2	159.0764	80.0418	141.0659	71.0366	G	1600.9672	800.9872	1583.9407	792.4740	1582.9566	791.9820	14
3	**260.1241**	130.5657	**242.1135**	121.5604	T	1543.9458	772.4765	1526.9192	763.9632	1525.9352	763.4712	13
4	**407.1925**	204.0999	**389.1819**	195.0946	F	**1442.8981**	721.9527	1425.8715	713.4394	1424.8875	712.9474	12
5	**520.2766**	260.6419	**502.2660**	251.6366	I	**1295.8297**	648.4185	1278.8031	639.9052	1277.8191	639.4132	11
6	**619.3450**	310.1761	**601.3344**	301.1708	V	**1182.7456**	591.8764	1165.7191	583.3632	1164.7350	582.8712	10
7	**732.4290**	366.7182	714.4185	357.7129	I	**1083.6772**	542.3422	1066.6506	533.8290	1065.6666	533.3370	9
8	**847.4560**	424.2316	829.4454	415.2263	D	**970.5931**	485.8002	953.5666	477.2869	952.5826	476.7949	8
9	**960.5400**	480.7737	942.5295	471.7684	I	**855.5662**	428.2867	838.5396	419.7735	837.5556	419.2815	7
10	1073.6241	537.3157	1055.6135	528.3104	L	**742.4821**	371.7447	725.4556	363.2314	724.4716	362.7394	6
11	**1186.7082**	593.8577	1168.6976	584.8524	I	**629.3981**	315.2027	612.3715	306.6894	611.3875	306.1974	5
12	1301.7351	651.3712	1283.7245	642.3659	D	**516.3140**	258.6606	499.2875	250.1474	498.3034	249.6554	4
13	1414.8192	707.9132	1396.8086	698.9079	I	**401.2871**	201.1472	384.2605	192.6339			3
14	1527.9032	764.4552	1509.8927	755.4500	I	**288.2030**	144.6051	271.1765	136.0919			2
15					R	**175.1190**	88.0631	158.0924	79.5498			1

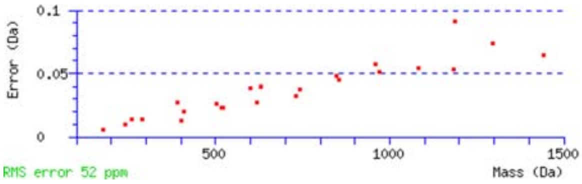

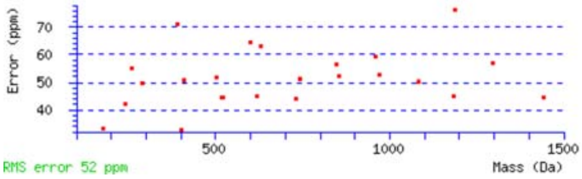

Peptide View

MS/MS Fragmentation of **TITELADWLVLIDQMLK**
Found in **gi|46575908|ref|NP_035812.2|**, utrophin [Mus musculus]

Match to Query 658: 2001.117448 from(1001.566000,2+)

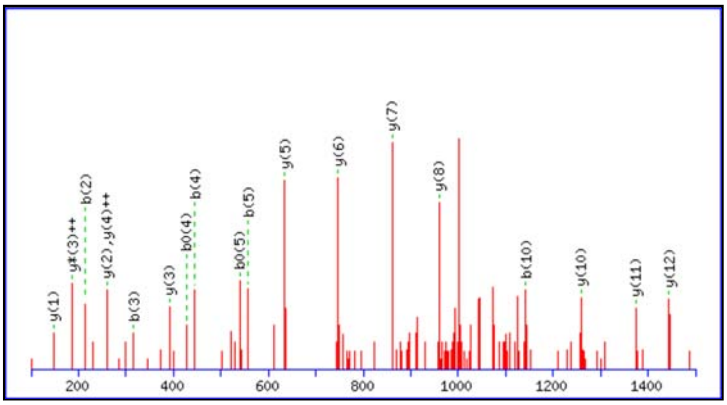

Monoisotopic mass of neutral peptide Mr(calc): 2001.0856
Ions Score: 67 **Expect:** 0.00083
Matches (**Bold Red**): 19/158 fragment ions using 28 most intense peaks

#	b	b^{++}	b*	$b*^{++}$	b^0	b^{0++}	Seq.	y	y^{++}	y*	$y*^{++}$	y^0	y^{0++}	#
1	102.0550	51.5311			84.0444	42.5258	T							17
2	**215.1390**	108.0731			197.1285	99.0679	I	1901.0452	951.0262	1884.0187	942.5130	1883.0346	942.0210	16
3	**316.1867**	158.5970			298.1761	149.5917	T	1787.9611	894.4842	1770.9346	885.9709	1769.9506	885.4789	15
4	**445.2293**	223.1183			**427.2187**	214.1130	E	1686.9135	843.9604	1669.8869	835.4471	1668.9029	834.9551	14
5	**558.3133**	279.6603			**540.3028**	270.6550	L	1557.8709	779.4391	1540.8443	770.9258	1539.8603	770.4338	13
6	629.3505	315.1789			611.3399	306.1736	A	**1444.7868**	722.8970	1427.7603	714.3838	1426.7762	713.8918	12
7	744.3774	372.6923			726.3668	363.6871	D	**1373.7497**	687.3785	1356.7232	678.8652	1355.7391	678.3732	11
8	930.4567	465.7320			912.4461	456.7267	W	**1258.7228**	629.8650	1241.6962	621.3517	1240.7122	620.8597	10
9	1043.5408	522.2740			1025.5302	513.2687	L	1072.6435	536.8254	1055.6169	528.3121	1054.6329	527.8201	9
10	**1142.6092**	571.8082			1124.5986	562.8029	V	**959.5594**	480.2833	942.5328	471.7701	941.5488	471.2781	8
11	1255.6932	628.3503			1237.6827	619.3450	L	**860.4910**	430.7491	843.4644	422.2359	842.4804	421.7438	7
12	1368.7773	684.8923			1350.7667	675.8870	I	**747.4069**	374.2071	730.3804	365.6938	729.3964	365.2018	6
13	1483.8042	742.4058			1465.7937	733.4005	D	**634.3229**	317.6651	617.2963	309.1518	616.3123	308.6598	5
14	1611.8628	806.4350	1594.8363	797.9218	1593.8523	797.4298	Q	519.2959	**260.1516**	502.2694	251.6383			4
15	1742.9033	871.9553	1725.8767	863.4420	1724.8927	862.9500	M	**391.2373**	196.1223	374.2108	**187.6090**			3
16	1855.9874	928.4973	1838.9608	919.9840	1837.9768	919.4920	L	**260.1969**	130.6021	243.1703	122.0888			2
17							K	**147.1128**	74.0600	130.0863	65.5468			1

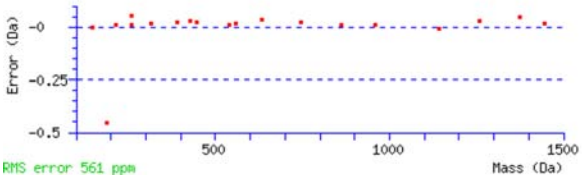

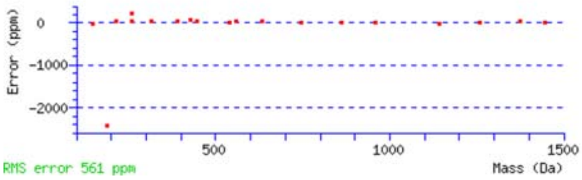

Peptide View

MS/MS Fragmentation of TLGSGAFGQVVEATAHGLSHSQATMK
Found in gi|6679259|ref|NP_032835.1|, platelet derived growth factor receptor, beta polypeptide [Mus musculus]

Match to Query 769: 2584.262472 from(862.428100,3+)

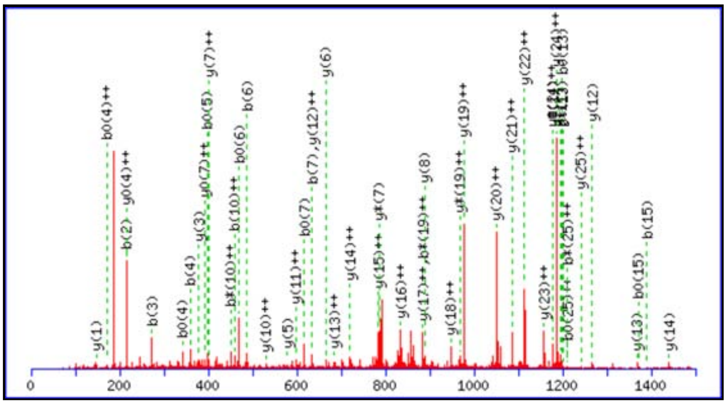

Monoisotopic mass of neutral peptide Mr(calc): 2584.2703
Ions Score: 57 Expect: 0.016
Matches (**Bold Red**): 52/280 fragment ions using 171 most intense peaks

#	b	b^{++}	b*	b^{*++}	b^0	b^{0++}	Seq.	y	y^{++}	y*	y^{*++}	y^0	y^{0++}	#
1	102.0550	51.5311			84.0444	42.5258	T							26
2	**215.1390**	108.0731			197.1285	99.0679	L	2484.2299	**1242.6186**	2467.2034	1234.1053	2466.2194	1233.6133	25
3	**272.1605**	136.5839			254.1499	127.5786	G	2371.1459	**1186.0766**	2354.1193	**1177.5633**	2353.1353	**1177.0713**	24
4	**359.1925**	180.0999			**341.1819**	**171.0946**	S	2314.1244	**1157.5658**	2297.0979	1149.0526	2296.1138	1148.5606	23
5	416.2140	208.6106			**398.2034**	199.6053	G	2227.0924	**1114.0498**	2210.0658	1105.5366	2209.0818	1105.0445	22
6	**487.2511**	244.1292			**469.2405**	235.1239	A	2170.0709	**1085.5391**	2153.0444	1077.0258	2152.0604	1076.5338	21
7	**634.3195**	317.6634			**616.3089**	308.6581	F	2099.0338	**1050.0205**	2082.0073	1041.5073	2081.0232	1041.0153	20
8	691.3409	346.1741			673.3304	337.1688	G	1951.9654	**976.4863**	1934.9389	**967.9731**	1933.9548	967.4811	19
9	819.3995	410.2034	802.3730	401.6901	801.3890	401.1981	Q	1894.9439	**947.9756**	1877.9174	939.4623	1876.9334	938.9703	18
10	918.4679	**459.7376**	901.4414	**451.2243**	900.4574	450.7323	V	1766.8854	**883.9463**	1749.8588	875.4330	1748.8748	874.9410	17
11	1017.5363	509.2718	1000.5098	500.7585	999.5258	500.2665	V	1667.8170	**834.4121**	1650.7904	825.8988	1649.8064	825.4068	16
12	1146.5789	573.7931	1129.5524	565.2798	1128.5684	564.7878	E	1568.7485	**784.8779**	1551.7220	776.3646	1550.7380	775.8726	15
13	1217.6160	609.3117	**1200.5895**	600.7984	**1199.6055**	600.3064	A	**1439.7060**	**720.3566**	1422.6794	711.8433	1421.6954	711.3513	14
14	1318.6637	659.8355	1301.6372	651.3222	1300.6532	650.8302	T	**1368.6688**	**684.8381**	1351.6423	676.3248	1350.6583	675.8328	13
15	**1389.7008**	695.3541	1372.6743	686.8408	**1371.6903**	686.3488	A	**1267.6212**	**634.3142**	1250.5946	625.8009	1249.6106	625.3089	12
16	1526.7597	763.8835	1509.7332	755.3702	1508.7492	754.8782	H	**1196.5841**	**598.7957**	1179.5575	590.2824	**1178.5735**	589.7904	11
17	1583.7812	792.3942	1566.7547	783.8810	1565.7706	783.3890	G	1059.5251	**530.2662**	1042.4986	521.7529	1041.5146	521.2609	10
18	1696.8653	848.9363	1679.8387	840.4230	1678.8547	839.9310	L	1002.5037	501.7555	985.4771	493.2422	984.4931	492.7502	9
19	1783.8973	892.4523	1766.8707	**883.9390**	1765.8867	883.4470	S	**889.4196**	445.2134	872.3931	436.7002	871.4091	436.2082	8
20	1920.9562	960.9817	1903.9297	952.4685	1902.9456	951.9765	H	802.3876	**401.6974**	**785.3610**	393.1842	784.3770	**392.6922**	7
21	2007.9882	1004.4978	1990.9617	995.9845	1989.9777	995.4925	S	**665.3287**	333.1680	648.3021	324.6547	647.3181	324.1627	6
22	2136.0468	1068.5270	2119.0203	1060.0138	2118.0363	1059.5218	Q	**578.2967**	289.6520	561.2701	281.1387	560.2861	280.6467	5
23	2207.0839	1104.0456	2190.0574	1095.5323	2189.0734	1095.0403	A	450.2381	225.6227	433.2115	217.1094	432.2275	**216.6174**	4
24	2308.1316	1154.5694	2291.1051	1146.0562	2290.1210	1145.5642	T	**379.2010**	190.1041	362.1744	181.5908	361.1904	181.0988	3
25	2439.1721	1220.0897	2422.1455	**1211.5764**	2421.1615	**1211.0844**	M	278.1533	139.5803	261.1267	131.0670			2
26							K	**147.1128**	74.0600	130.0863	65.5468			1

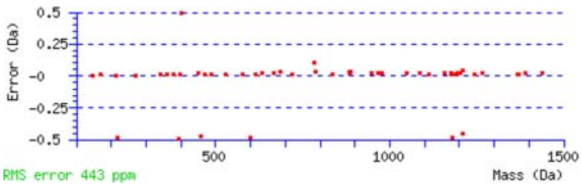

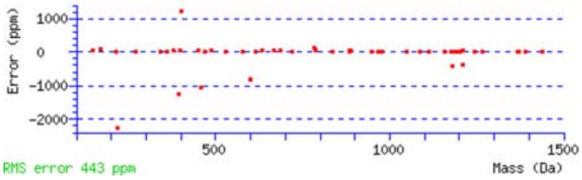

Peptide View

MS/MS Fragmentation of **TLVTQNSGVEALIHAILR**
Found in **gi|28395018|ref|NP_034723.1|**, junction plakoglobin [Mus musculus]

Match to Query 1073: 1940.228472 from(647.750100,3+)

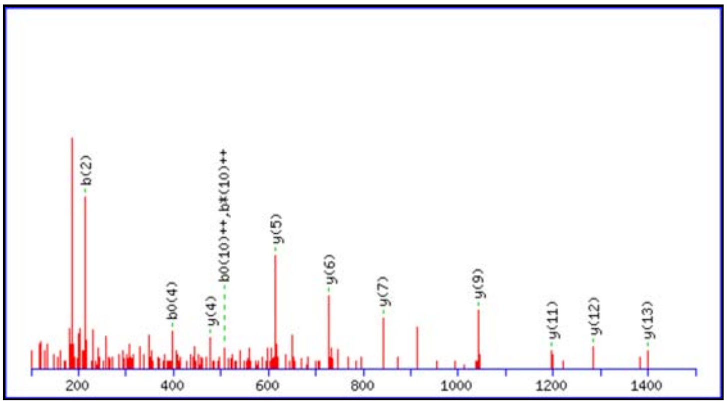

Monoisotopic mass of neutral peptide Mr(calc): 1940.1150
Variable modifications:
R18 : Arginine-13C6 (R-13C6)
Ions Score: 53 **Expect:** 0.02
Matches (**Bold Red**): 12/180 fragment ions using 16 most intense peaks

#	b	b^{++}	b*	b^{*++}	b^0	b^{0++}	Seq.	y	y^{++}	y*	y^{*++}	y^0	y^{0++}	#
1	102.0550	51.5311			84.0444	42.5258	T							18
2	**215.1390**	108.0731			197.1285	99.0679	L	1840.0746	920.5409	1823.0480	912.0277	1822.0640	911.5357	17
3	314.2074	157.6074			296.1969	148.6021	V	1726.9905	863.9989	1709.9640	855.4856	1708.9800	854.9936	16
4	415.2551	208.1312			**397.2445**	199.1259	T	1627.9221	814.4647	1610.8956	805.9514	1609.9116	805.4594	15
5	543.3137	272.1605	526.2871	263.6472	525.3031	263.1552	Q	1526.8744	763.9409	1509.8479	755.4276	1508.8639	754.9356	14
6	657.3566	329.1819	640.3301	320.6687	639.3461	320.1767	N	**1398.8159**	699.9116	1381.7893	691.3983	1380.8053	690.9063	13
7	744.3886	372.6980	727.3621	364.1847	726.3781	363.6927	S	**1284.7729**	642.8901	1267.7464	634.3768	1266.7624	633.8848	12
8	801.4101	401.2087	784.3836	392.6954	783.3995	392.2034	G	**1197.7409**	599.3741	1180.7144	590.8608	1179.7303	590.3688	11
9	900.4785	450.7429	883.4520	442.2296	882.4680	441.7376	V	1140.7194	570.8634	1123.6929	562.3501	1122.7089	561.8581	10
10	1029.5211	515.2642	1012.4946	**506.7509**	1011.5105	**506.2589**	E	**1041.6510**	521.3292	1024.6245	512.8159	1023.6405	512.3239	9
11	1100.5582	550.7827	1083.5317	542.2695	1082.5477	541.7775	A	912.6084	456.8079	895.5819	448.2946			8
12	1213.6423	607.3248	1196.6157	598.8115	1195.6317	598.3195	L	**841.5713**	421.2893	824.5448	412.7760			7
13	1326.7263	663.8668	1309.6998	655.3535	1308.7158	654.8615	I	**728.4873**	364.7473	711.4607	356.2340			6
14	1463.7852	732.3963	1446.7587	723.8830	1445.7747	723.3910	H	**615.4032**	308.2052	598.3767	299.6920			5
15	1534.8224	767.9148	1517.7958	759.4015	1516.8118	758.9095	A	**478.3443**	239.6758	461.3178	231.1625			4
16	1647.9064	824.4568	1630.8799	815.9436	1629.8959	815.4516	I	407.3072	204.1572	390.2806	195.6440			3
17	1760.9905	880.9989	1743.9639	872.4856	1742.9799	871.9936	L	294.2231	147.6152	277.1966	139.1019			2
18							R	181.1391	91.0732	164.1125	82.5599			1

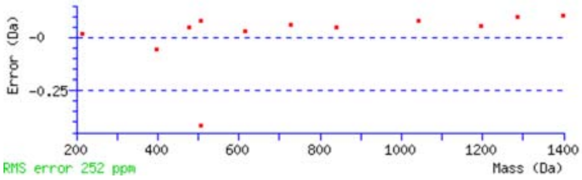

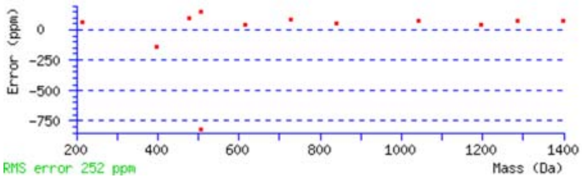

Peptide View

MS/MS Fragmentation of TPFSLVGNVFELNFK
Found in gi|34996495|ref|NP_062616.2|, ribophorin II [Mus musculus]

Match to Query 408: 1710.992248 from(856.503400,2+)

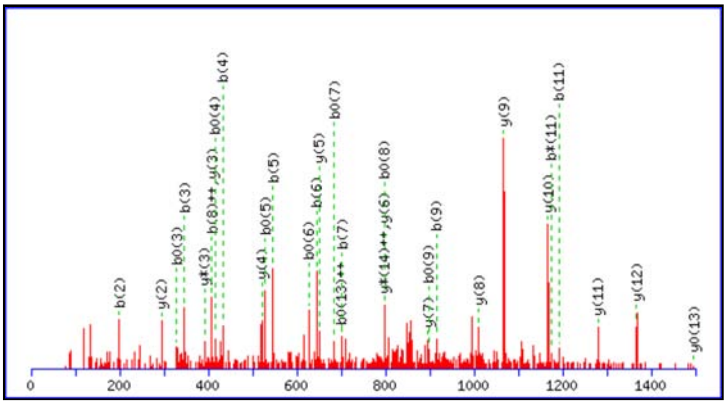

Monoisotopic mass of neutral peptide Mr(calc): 1710.8980
Ions Score: 52 **Expect**: 0.019
Matches (**Bold Red**): 32/146 fragment ions using 79 most intense peaks

#	b	b^{++}	b*	b^{*++}	b^{0}	b^{0++}	Seq.	y	y^{++}	y*	y^{*++}	y^{0}	y^{0++}	#
1	102.0550	51.5311			84.0444	42.5258	T							15
2	**199.1077**	100.0575			181.0972	91.0522	P	1610.8577	805.9325	1593.8311	**797.4192**	1592.8471	796.9272	14
3	**346.1761**	173.5917			**328.1656**	164.5864	F	1513.8049	757.4061	1496.7784	748.8928	**1495.7943**	748.4008	13
4	**433.2082**	217.1077			**415.1976**	208.1024	S	**1366.7365**	683.8719	1349.7100	675.3586	1348.7259	674.8666	12
5	**546.2922**	273.6497			**528.2817**	264.6445	L	**1279.7045**	640.3559	1262.6779	631.8426	1261.6939	631.3506	11
6	**645.3606**	323.1840			**627.3501**	314.1787	V	**1166.6204**	583.8138	1149.5939	575.3006	1148.6098	574.8086	10
7	**702.3821**	351.6947			**684.3715**	342.6894	G	**1067.5520**	534.2796	1050.5255	525.7664	1049.5414	525.2744	9
8	816.4250	**408.7161**	799.3985	400.2029	**798.4145**	399.7109	N	**1010.5305**	505.7689	993.5040	497.2556	992.5200	496.7636	8
9	**915.4934**	458.2504	898.4669	449.7371	**897.4829**	449.2451	V	**896.4876**	448.7474	879.4611	440.2342	878.4770	439.7422	7
10	1062.5618	531.7846	1045.5353	523.2713	1044.5513	522.7793	F	**797.4192**	399.2132	780.3927	390.7000	779.4086	390.2080	6
11	**1191.6044**	596.3059	**1174.5779**	587.7926	1173.5939	587.3006	E	**650.3508**	325.6790	633.3242	317.1658	632.3402	316.6738	5
12	1304.6885	652.8479	1287.6619	644.3346	1286.6779	643.8426	L	**521.3082**	261.1577	504.2817	252.6445			4
13	1418.7314	709.8693	1401.7049	701.3561	1400.7209	**700.8641**	N	**408.2241**	204.6157	**391.1976**	196.1024			3
14	1565.7998	783.4036	1548.7733	774.8903	1547.7893	774.3983	F	**294.1812**	147.5942	277.1547	139.0810			2
15							K	147.1128	74.0600	130.0863	65.5468			1

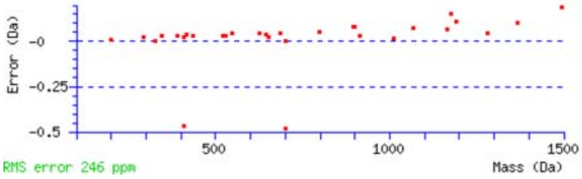

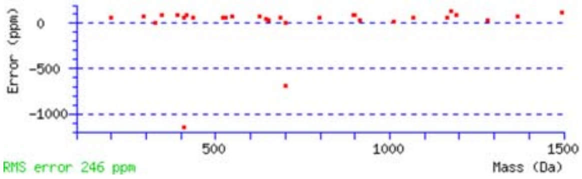

Peptide View

MS/MS Fragmentation of TTFQNLIEGFEALLK
Found in gi|22164788|ref|NP_666257.1|, Janus kinase 1 [Mus musculus]

Match to Query 584: 1722.954048 from(862.484300,2+)

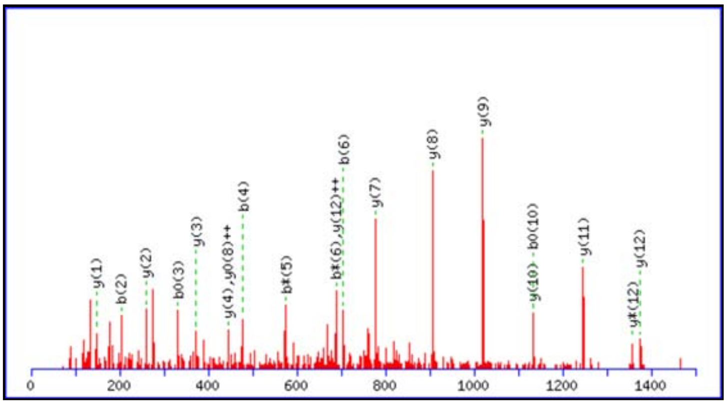

Monoisotopic mass of neutral peptide Mr(calc): 1722.9192
Ions Score: 72 **Expect:** 0.00018
Matches (**Bold Red**): 20/154 fragment ions using 29 most intense peaks

#	b	b^{++}	b*	b^{*++}	b^{0}	b^{0++}	Seq.	y	y^{++}	y*	y^{*++}	y^{0}	y^{0++}	#
1	102.0550	51.5311			84.0444	42.5258	T							15
2	**203.1026**	102.0550			185.0921	93.0497	T	1622.8788	811.9430	1605.8523	803.4298	1604.8682	802.9378	14
3	350.1710	175.5892			**332.1605**	166.5839	F	1521.8311	761.4192	1504.8046	752.9059	1503.8206	752.4139	13
4	**478.2296**	239.6185	461.2031	231.1052	460.2191	230.6132	Q	**1374.7627**	**687.8850**	**1357.7362**	679.3717	1356.7521	678.8797	12
5	592.2726	296.6399	**575.2460**	288.1266	574.2620	287.6346	N	**1246.7041**	623.8557	1229.6776	615.3424	1228.6936	614.8504	11
6	**705.3566**	353.1819	**688.3301**	344.6687	687.3461	344.1767	L	**1132.6612**	566.8342	1115.6347	558.3210	1114.6506	557.8290	10
7	818.4407	409.7240	801.4141	401.2107	800.4301	400.7187	I	**1019.5771**	510.2922	1002.5506	501.7789	1001.5666	501.2869	9
8	947.4833	474.2453	930.4567	465.7320	929.4727	465.2400	E	**906.4931**	453.7502	889.4665	445.2369	888.4825	**444.7449**	8
9	1004.5047	502.7560	987.4782	494.2427	986.4942	493.7507	G	**777.4505**	389.2289	760.4239	380.7156	759.4399	380.2236	7
10	1151.5731	576.2902	1134.5466	567.7769	**1133.5626**	567.2849	F	720.4290	360.7182	703.4025	352.2049	702.4185	351.7129	6
11	1280.6157	640.8115	1263.5892	632.2982	1262.6052	631.8062	E	573.3606	287.1839	556.3341	278.6707	555.3501	278.1787	5
12	1351.6528	676.3301	1334.6263	667.8168	1333.6423	667.3248	A	**444.3180**	222.6627	427.2915	214.1494			4
13	1464.7369	732.8721	1447.7103	724.3588	1446.7263	723.8668	L	**373.2809**	187.1441	356.2544	178.6308			3
14	1577.8210	789.4141	1560.7944	780.9008	1559.8104	780.4088	L	**260.1969**	130.6021	243.1703	122.0888			2
15							K	**147.1128**	74.0600	130.0863	65.5468			1

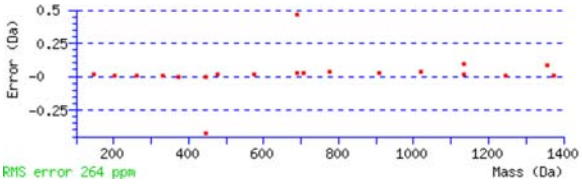

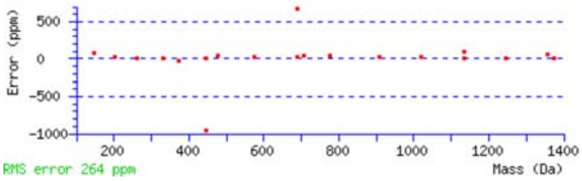

Peptide View

MS/MS Fragmentation of **TVAGGAWTYNTTSAVTVK**
Found in **gi|6677785|ref|NP_033110.1|**, ribosomal protein L37a [Mus musculus]

Match to Query 602: 1825.979048 from(913.996800,2+)

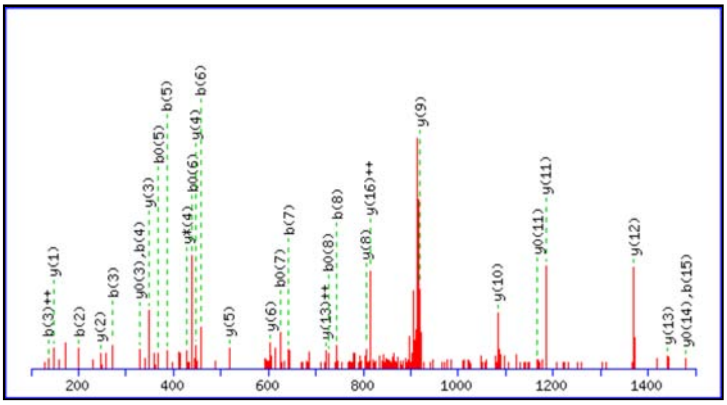

Monoisotopic mass of neutral peptide Mr(calc): 1825.9210
Ions Score: 55 **Expect:** 0.0087
Matches (**Bold Red**): 31/182 fragment ions using 77 most intense peaks

#	b	b^{++}	b*	b^{*++}	b^0	b^{0++}	Seq.	y	y^{++}	y*	y^{*++}	y^0	y^{0++}	#
1	102.0550	51.5311			84.0444	42.5258	T							18
2	**201.1234**	101.0653			183.1128	92.0600	V	1725.8806	863.4439	1708.8541	854.9307	1707.8700	854.4387	17
3	**272.1605**	**136.5839**			254.1499	127.5786	A	1626.8122	**813.9097**	1609.7856	805.3965	1608.8016	804.9045	16
4	**329.1819**	165.0946			311.1714	156.0893	G	1555.7751	778.3912	1538.7485	769.8779	1537.7645	769.3859	15
5	**386.2034**	193.6053			**368.1928**	184.6001	G	1498.7536	749.8804	1481.7271	741.3672	**1480.7431**	740.8752	14
6	**457.2405**	229.1239			**439.2299**	220.1186	A	**1441.7322**	**721.3697**	1424.7056	712.8564	1423.7216	712.3644	13
7	**643.3198**	322.1635			**625.3093**	313.1583	W	**1370.6951**	685.8512	1353.6685	677.3379	1352.6845	676.8459	12
8	**744.3675**	372.6874			**726.3569**	363.6821	T	**1184.6157**	592.8115	1167.5892	584.2982	**1166.6052**	583.8062	11
9	907.4308	454.2191			889.4203	445.2138	Y	**1083.5681**	542.2877	1066.5415	533.7744	1065.5575	533.2824	10
10	1021.4738	511.2405	1004.4472	502.7272	1003.4632	502.2352	N	**920.5047**	460.7560	903.4782	452.2427	902.4942	451.7507	9
11	1122.5214	561.7644	1105.4949	553.2511	1104.5109	552.7591	T	**806.4618**	403.7345	789.4353	395.2213	788.4512	394.7293	8
12	1223.5691	612.2882	1206.5426	603.7749	1205.5586	603.2829	T	705.4141	353.2107	688.3876	344.6974	687.4036	344.2054	7
13	1310.6011	655.8042	1293.5746	647.2909	1292.5906	646.7989	S	**604.3664**	302.6869	587.3399	294.1736	586.3559	293.6816	6
14	1381.6383	691.3228	1364.6117	682.8095	1363.6277	682.3175	A	**517.3344**	259.1708	500.3079	250.6576	499.3238	250.1656	5
15	**1480.7067**	740.8570	1463.6801	732.3437	1462.6961	731.8517	V	**446.2973**	223.6523	**429.2708**	215.1390	428.2867	214.6470	4
16	1581.7543	791.3808	1564.7278	782.8675	1563.7438	782.3755	T	**347.2289**	174.1181	330.2023	165.6048	**329.2183**	165.1128	3
17	1680.8228	840.9150	1663.7962	832.4017	1662.8122	831.9097	V	**246.1812**	123.5942	229.1547	115.0810			2
18							K	**147.1128**	74.0600	130.0863	65.5468			1

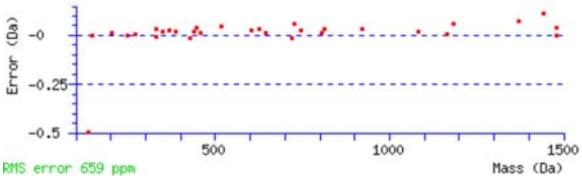

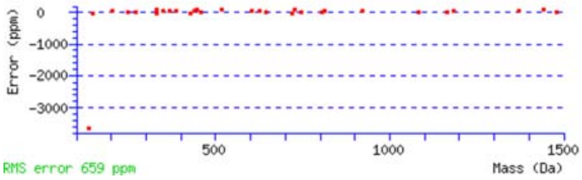

Peptide View

MS/MS Fragmentation of **VIHDNFGIVEGLMTTVHAITATQK**
Found in **gi|41054968|ref|NP_955766.1|**, glyceraldehyde-3-phosphate dehydrogenase (phosphorylating)-like [Mus musculus]

Match to Query 622: 2594.463072 from(865.828300,3+)

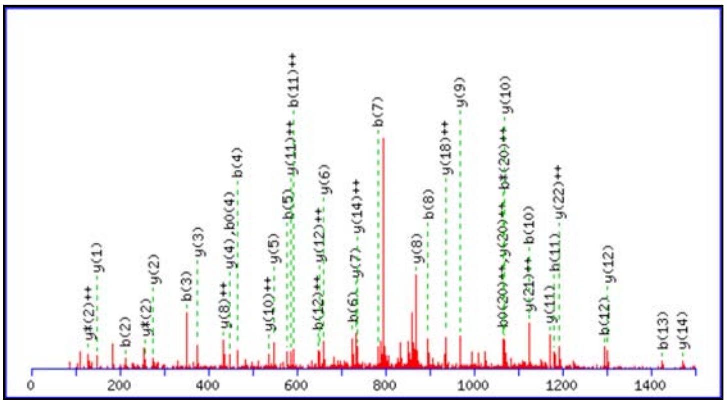

Monoisotopic mass of neutral peptide Mr(calc): 2594.3526
Ions Score: 98 Expect: 1.2e-006
Matches (**Bold Red**): 40/258 fragment ions using 67 most intense peaks

#	b	b^{++}	b*	b^{*++}	b^0	b^{0++}	Seq.	y	y^{++}	y*	y^{*++}	y^0	y^{0++}	#
1	100.0757	50.5415					V							24
2	**213.1597**	107.0835					I	2496.2915	1248.6494	2479.2649	1240.1361	2478.2809	1239.6441	23
3	**350.2187**	175.6130					H	2383.2074	**1192.1073**	2366.1809	1183.5941	2365.1968	1183.1021	22
4	**465.2456**	233.1264			**447.2350**	224.1212	D	2246.1485	**1123.5779**	2229.1220	1115.0646	2228.1379	1114.5726	21
5	**579.2885**	290.1479	562.2620	281.6346	561.2780	281.1426	N	2131.1216	**1066.0644**	2114.0950	1057.5511	2113.1110	1057.0591	20
6	**726.3569**	363.6821	709.3304	355.1688	708.3464	354.6768	F	2017.0786	1009.0430	2000.0521	1000.5297	1999.0681	1000.0377	19
7	**783.3784**	392.1928	766.3518	383.6796	765.3678	383.1876	G	1870.0102	**935.5087**	1852.9837	926.9955	1851.9997	926.5035	18
8	**896.4625**	448.7349	879.4359	440.2216	878.4519	439.7296	I	1812.9888	906.9980	1795.9622	898.4847	1794.9782	897.9927	17
9	995.5309	498.2691	978.5043	489.7558	977.5203	489.2638	V	1699.9047	850.4560	1682.8782	841.9427	1681.8941	841.4507	16
10	**1124.5735**	562.7904	1107.5469	554.2771	1106.5629	553.7851	E	1600.8363	800.9218	1583.8097	792.4085	1582.8257	791.9165	15
11	**1181.5949**	**591.3011**	1164.5684	582.7878	1163.5844	582.2958	G	**1471.7937**	**736.4005**	1454.7672	727.8872	1453.7831	727.3952	14
12	**1294.6790**	**647.8431**	1277.6524	639.3299	1276.6684	638.8378	L	1414.7722	707.8898	1397.7457	699.3765	1396.7617	698.8845	13
13	**1425.7195**	713.3634	1408.6929	704.8501	1407.7089	704.3581	M	**1301.6882**	**651.3477**	1284.6616	642.8345	1283.6776	642.3424	12
14	1526.7671	763.8872	1509.7406	755.3739	1508.7566	754.8819	T	**1170.6477**	**585.8275**	1153.6212	577.3142	1152.6371	576.8222	11
15	1627.8148	814.4110	1610.7883	805.8978	1609.8043	805.4058	T	**1069.6000**	**535.3036**	1052.5735	526.7904	1051.5895	526.2984	10
16	1726.8832	863.9453	1709.8567	855.4320	1708.8727	854.9400	V	**968.5523**	484.7798	951.5258	476.2665	950.5418	475.7745	9
17	1863.9421	932.4747	1846.9156	923.9614	1845.9316	923.4694	H	**869.4839**	**435.2456**	852.4574	426.7323	851.4734	426.2403	8
18	1934.9792	967.9933	1917.9527	959.4800	1916.9687	958.9880	A	**732.4250**	366.7161	715.3985	358.2029	714.4145	357.7109	7
19	2048.0633	1024.5353	2031.0368	1016.0220	2030.0527	1015.5300	I	**661.3879**	331.1976	644.3614	322.6843	643.3773	322.1923	6
20	2149.1110	1075.0591	2132.0844	**1066.5459**	2131.1004	**1066.0538**	T	**548.3039**	274.6556	531.2773	266.1423	530.2933	265.6503	5
21	2220.1481	1110.5777	2203.1215	1102.0644	2202.1375	1101.5724	A	**447.2562**	224.1317	430.2296	215.6184	429.2456	215.1264	4
22	2321.1958	1161.1015	2304.1692	1152.5883	2303.1852	1152.0962	T	**376.2191**	188.6132	359.1925	180.0999	358.2085	179.6079	3
23	2449.2544	1225.1308	2432.2278	1216.6175	2431.2438	1216.1255	Q	**275.1714**	138.0893	**258.1448**	**129.5761**			2
24							K	**147.1128**	74.0600	130.0863	65.5468			1

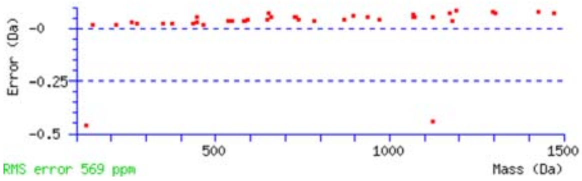

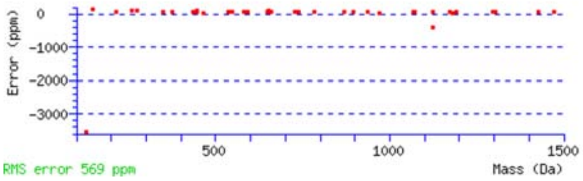

Peptide View

MS/MS Fragmentation of **VINIFQSNLFQALIDIQEFYEVTLLDNPK**
Found in **gi|82965034|ref|XP_929204.1|**, PREDICTED: similar to discs large homolog 1 isoform 6 [Mus musculus]

Match to Query 764: 3423.992172 from(1142.338000,3+)

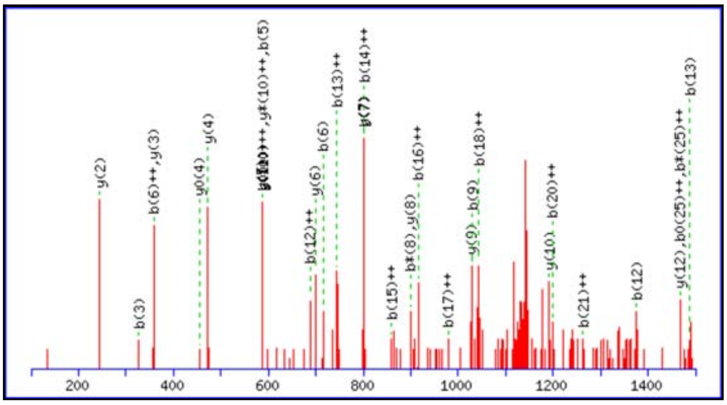

Monoisotopic mass of neutral peptide Mr(calc): 3423.7965
Ions Score: 60 Expect: 0.013
Matches (**Bold Red**): 34/314 fragment ions using 67 most intense peaks

#	b	b^{++}	b*	b^{*++}	b^0	b^{0++}	Seq.	y	y^{++}	y*	y^{*++}	y^0	y^{0++}	#
1	100.0757	50.5415					V							29
2	213.1597	107.0835					I	3325.7354	1663.3713	3308.7089	1654.8581	3307.7249	1654.3661	28
3	**327.2027**	164.1050	310.1761	155.5917			N	3212.6514	1606.8293	3195.6248	1598.3160	3194.6408	1597.8240	27
4	440.2867	220.6470	423.2602	212.1337			I	3098.6084	1549.8079	3081.5819	1541.2946	3080.5979	1540.8026	26
5	**587.3551**	294.1812	570.3286	285.6679			F	2985.5244	1493.2658	2968.4978	1484.7525	2967.5138	1484.2605	25
6	**715.4137**	**358.2105**	698.3872	349.6972			Q	2838.4560	1419.7316	2821.4294	1411.2183	2820.4454	1410.7263	24
7	**802.4458**	401.7265	785.4192	393.2132	784.4352	392.7212	S	2710.3974	1355.7023	2693.3708	1347.1891	2692.3868	1346.6970	23
8	916.4887	458.7480	**899.4621**	450.2347	898.4781	449.7427	N	2623.3654	1312.1863	2606.3388	1303.6730	2605.3548	1303.1810	22
9	**1029.5727**	515.2900	1012.5462	506.7767	1011.5622	506.2847	L	2509.3224	1255.1648	2492.2959	1246.6516	2491.3119	1246.1596	21
10	1176.6412	**588.8242**	1159.6146	580.3109	1158.6306	579.8189	F	2396.2384	1198.6228	2379.2118	1190.1095	2378.2278	1189.6175	20
11	1304.6997	652.8535	1287.6732	644.3402	1286.6892	643.8482	Q	2249.1700	1125.0886	2232.1434	1116.5753	2231.1594	1116.0833	19
12	**1375.7368**	**688.3721**	1358.7103	679.8588	1357.7263	679.3668	A	2121.1114	1061.0593	2104.0848	1052.5460	2103.1008	1052.0540	18
13	**1488.8209**	**744.9141**	1471.7944	736.4008	1470.8103	735.9088	L	2050.0743	1025.5408	2033.0477	1017.0275	2032.0637	1016.5355	17
14	1601.9050	**801.4561**	1584.8784	792.9428	1583.8944	792.4508	I	1936.9902	968.9987	1919.9637	960.4855	1918.9796	959.9935	16
15	1716.9319	**858.9696**	1699.9054	850.4563	1698.9213	849.9643	D	1823.9061	912.4567	1806.8796	903.9434	1805.8956	903.4514	15
16	1830.0160	**915.5116**	1812.9894	906.9983	1812.0054	906.5063	I	1708.8792	854.9432	1691.8527	846.4300	1690.8686	845.9380	14
17	1958.0745	**979.5409**	1941.0480	971.0276	1940.0640	970.5356	Q	1595.7951	798.4012	1578.7686	789.8879	1577.7846	789.3959	13
18	2087.1171	**1044.0622**	2070.0906	1035.5489	2069.1066	1035.0569	E	**1467.7366**	734.3719	1450.7100	725.8586	1449.7260	725.3666	12
19	2234.1855	1117.5964	2217.1590	1109.0831	2216.1750	1108.5911	F	1338.6940	669.8506	1321.6674	661.3373	1320.6834	660.8453	11
20	2397.2489	**1199.1281**	2380.2223	1190.6148	2379.2383	1190.1228	Y	**1191.6256**	596.3164	1174.5990	**587.8031**	1173.6150	**587.3111**	10
21	2526.2915	**1263.6494**	2509.2649	1255.1361	2508.2809	1254.6441	E	**1028.5622**	514.7848	1011.5357	506.2715	1010.5517	505.7795	9
22	2625.3599	1313.1836	2608.3333	1304.6703	2607.3493	1304.1783	V	**899.5196**	450.2635	882.4931	441.7502	881.5091	441.2582	8
23	2726.4076	1363.7074	2709.3810	1355.1941	2708.3970	1354.7021	T	**800.4512**	400.7293	783.4247	392.2160	782.4407	391.7240	7
24	2839.4916	1420.2494	2822.4651	1411.7362	2821.4811	1411.2442	L	**699.4036**	350.2054	682.3770	341.6921	681.3930	341.2001	6
25	2952.5757	1476.7915	2935.5491	**1468.2782**	2934.5651	**1467.7862**	L	**586.3195**	293.6634	569.2929	285.1501	568.3089	284.6581	5
26	3067.6026	1534.3049	3050.5761	1525.7917	3049.5921	1525.2997	D	**473.2354**	237.1214	456.2089	228.6081	**455.2249**	228.1161	4
27	3181.6455	1591.3264	3164.6190	1582.8131	3163.6350	1582.3211	N	**358.2085**	179.6079	341.1819	171.0946			3
28	3278.6983	1639.8528	3261.6718	1631.3395	3260.6877	1630.8475	P	**244.1656**	122.5864	227.1390	114.0731			2
29							K	147.1128	74.0600	130.0863	65.5468			1

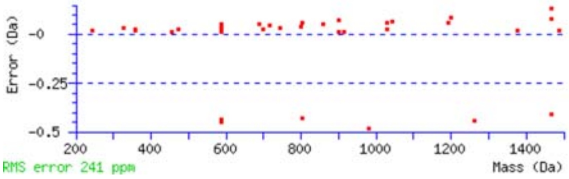

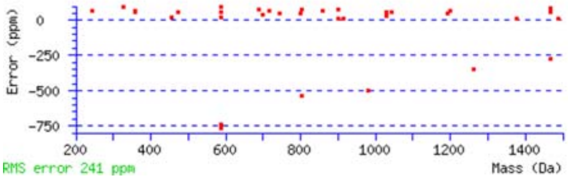

Peptide View

MS/MS Fragmentation of VLALIQAWADAFR
Found in gi|84875524|ref|NP_001034181.1|, target of myb1-like 2 isoform b [Mus musculus]

Match to Query 307: 1472.888048 from(737.451300,2+)

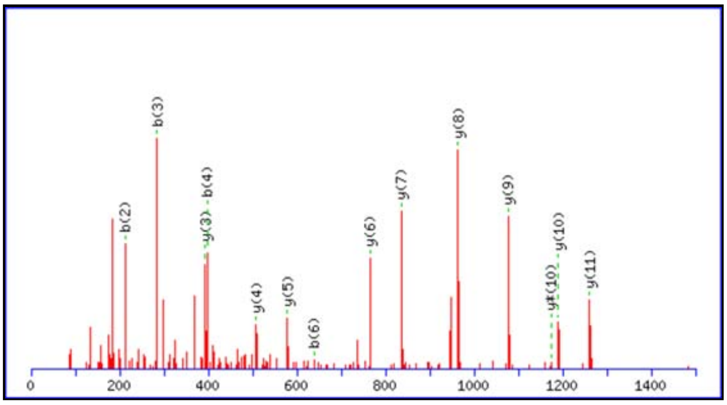

Monoisotopic mass of neutral peptide Mr(calc): 1472.8139
Ions Score: 63 **Expect:** 0.00093
Matches (Bold Red): 14/110 fragment ions using 28 most intense peaks

#	b	b^{++}	b*	$b*^{++}$	b^0	b^{0++}	Seq.	y	y^{++}	y*	$y*^{++}$	y^0	y^{0++}	#
1	100.0757	50.5415					V							13
2	**213.1597**	107.0835					L	1374.7528	687.8800	1357.7263	679.3668	1356.7422	678.8748	12
3	**284.1969**	142.6021					A	**1261.6688**	631.3380	1244.6422	622.8247	1243.6582	622.3327	11
4	**397.2809**	199.1441					L	**1190.6316**	595.8195	**1173.6051**	587.3062	1172.6211	586.8142	10
5	510.3650	255.6861					I	**1077.5476**	539.2774	1060.5210	530.7642	1059.5370	530.2721	9
6	**638.4236**	319.7154	621.3970	311.2021			Q	**964.4635**	482.7354	947.4370	474.2221	946.4530	473.7301	8
7	709.4607	355.2340	692.4341	346.7207			A	**836.4049**	418.7061	819.3784	410.1928	818.3944	409.7008	7
8	895.5400	448.2736	878.5134	439.7604			W	**765.3678**	383.1876	748.3413	374.6743	747.3573	374.1823	6
9	966.5771	483.7922	949.5505	475.2789			A	**579.2885**	290.1479	562.2620	281.6346	561.2780	281.1426	5
10	1081.6040	541.3057	1064.5775	532.7924	1063.5935	532.3004	D	**508.2514**	254.6293	491.2249	246.1161	490.2408	245.6241	4
11	1152.6411	576.8242	1135.6146	568.3109	1134.6306	567.8189	A	**393.2245**	197.1159	376.1979	188.6026			3
12	1299.7095	650.3584	1282.6830	641.8451	1281.6990	641.3531	F	322.1874	161.5973	305.1608	153.0840			2
13							R	175.1190	88.0631	158.0924	79.5498			1

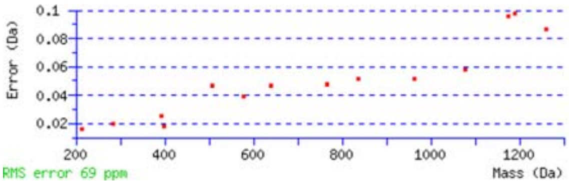

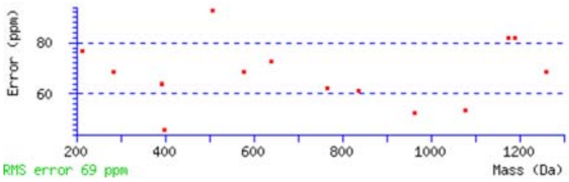

Peptide View

MS/MS Fragmentation of VLNLIQSWADAFR
Found in gi|94378129|ref|XP_001003823.1|, PREDICTED: similar to Target of Myb protein 1 isoform 6 [Mus musculus]

Match to Query 481: 1531.844048 from(766.929300,2+)

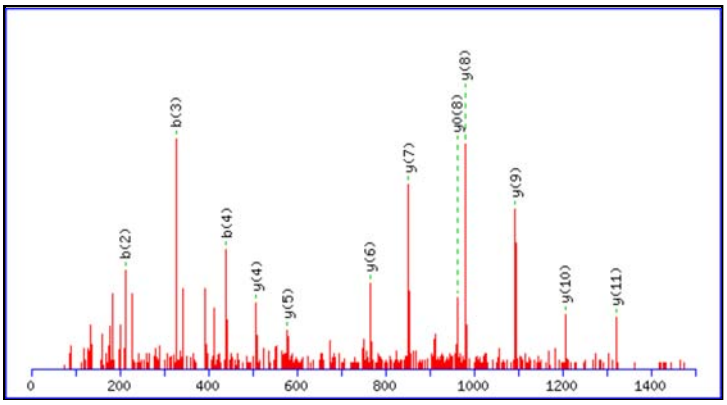

Monoisotopic mass of neutral peptide Mr(calc): 1531.8147
Ions Score: 71 Expect: 0.00014
Matches (Bold Red): 12/122 fragment ions using 19 most intense peaks

#	b	b^{++}	b*	b^{*++}	b^0	b^{0++}	Seq.	y	y^{++}	y*	y^{*++}	y^0	y^{0++}	#
1	100.0757	50.5415					V							13
2	**213.1597**	107.0835					L	1433.7536	717.3804	1416.7270	708.8671	1415.7430	708.3751	12
3	**327.2027**	164.1050	310.1761	155.5917			N	**1320.6695**	660.8384	1303.6429	652.3251	1302.6589	651.8331	11
4	**440.2867**	220.6470	423.2602	212.1337			L	**1206.6266**	603.8169	1189.6000	595.3036	1188.6160	594.8116	10
5	553.3708	277.1890	536.3442	268.6758			I	**1093.5425**	547.2749	1076.5160	538.7616	1075.5319	538.2696	9
6	681.4294	341.2183	664.4028	332.7051			Q	**980.4584**	490.7329	963.4319	482.2196	**962.4479**	481.7276	8
7	768.4614	384.7343	751.4349	376.2211	750.4508	375.7291	S	**852.3999**	426.7036	835.3733	418.1903	834.3893	417.6983	7
8	954.5407	477.7740	937.5142	469.2607	936.5302	468.7687	W	**765.3678**	383.1876	748.3413	374.6743	747.3573	374.1823	6
9	1025.5778	513.2926	1008.5513	504.7793	1007.5673	504.2873	A	**579.2885**	290.1479	562.2620	281.6346	561.2780	281.1426	5
10	1140.6048	570.8060	1123.5782	562.2927	1122.5942	561.8007	D	**508.2514**	254.6293	491.2249	246.1161	490.2408	245.6241	4
11	1211.6419	606.3246	1194.6153	597.8113	1193.6313	597.3193	A	393.2245	197.1159	376.1979	188.6026			3
12	1358.7103	679.8588	1341.6837	671.3455	1340.6997	670.8535	F	322.1874	161.5973	305.1608	153.0840			2
13							R	175.1190	88.0631	158.0924	79.5498			1

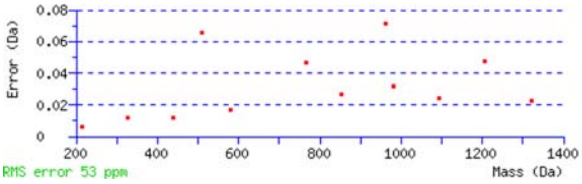

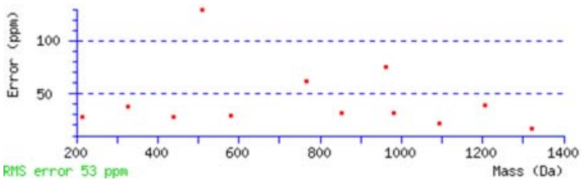

Peptide View

MS/MS Fragmentation of VTLYDPMSGILTSVQTK
Found in gi|56699423|ref|NP_035020.2|, neural precursor cell expressed, developmentally down-regulated gene 4 [Mus musculus]

Match to Query 624: 1851.970848 from(926.992700,2+)

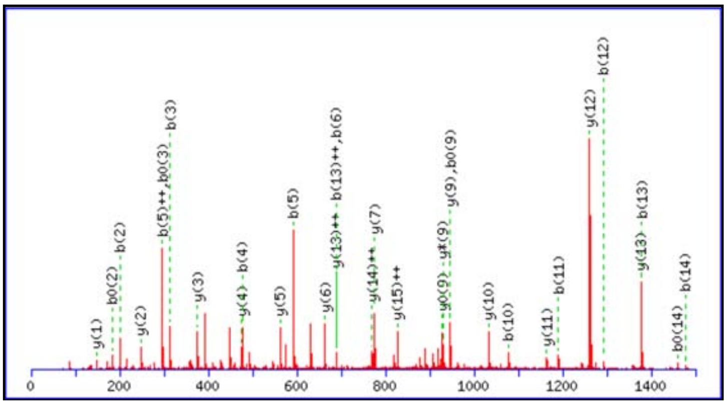

Monoisotopic mass of neutral peptide Mr(calc): 1851.9652
Ions Score: 85 Expect: 1e-005
Matches (Bold Red): 33/160 fragment ions using 53 most intense peaks

#	b	b^{++}	b*	b^{*++}	b^{0}	b^{0++}	Seq.	y	y^{++}	y*	y^{*++}	y^{0}	y^{0++}	#
1	100.0757	50.5415					V							17
2	**201.1234**	101.0653			**183.1128**	92.0600	T	1753.9040	877.4557	1736.8775	868.9424	1735.8935	868.4504	16
3	**314.2074**	157.6074			**296.1969**	148.6021	L	1652.8564	**826.9318**	1635.8298	818.4185	1634.8458	817.9265	15
4	**477.2708**	239.1390			459.2602	230.1337	Y	1539.7723	**770.3898**	1522.7458	761.8765	1521.7617	761.3845	14
5	**592.2977**	**296.6525**			574.2871	287.6472	D	**1376.7090**	**688.8581**	1359.6824	680.3448	1358.6984	679.8528	13
6	**689.3505**	345.1789			671.3399	336.1736	P	**1261.6820**	631.3447	1244.6555	622.8314	1243.6715	622.3394	12
7	820.3909	410.6991			802.3804	401.6938	M	**1164.6293**	582.8183	1147.6027	574.3050	1146.6187	573.8130	11
8	907.4230	454.2151			889.4124	445.2098	S	**1033.5888**	517.2980	1016.5622	508.7848	1015.5782	508.2928	10
9	964.4444	482.7259			**946.4339**	473.7206	G	**946.5568**	473.7820	**929.5302**	465.2687	**928.5462**	464.7767	9
10	**1077.5285**	539.2679			1059.5179	530.2626	I	889.5353	445.2713	872.5088	436.7580	871.5247	436.2660	8
11	**1190.6125**	595.8099			1172.6020	586.8046	L	**776.4512**	388.7293	759.4247	380.2160	758.4407	379.7240	7
12	**1291.6602**	646.3338			1273.6497	637.3285	T	**663.3672**	332.1872	646.3406	323.6740	645.3566	323.1819	6
13	**1378.6923**	**689.8498**			1360.6817	680.8445	S	**562.3195**	281.6634	545.2930	273.1501	544.3089	272.6581	5
14	**1477.7607**	739.3840			**1459.7501**	730.3787	V	**475.2875**	238.1474	458.2609	229.6341	457.2769	229.1421	4
15	1605.8192	803.4133	1588.7927	794.9000	1587.8087	794.4080	Q	**376.2191**	188.6132	359.1925	180.0999	358.2085	179.6079	3
16	1706.8669	853.9371	1689.8404	845.4238	1688.8564	844.9318	T	**248.1605**	124.5839	231.1339	116.0706	230.1499	115.5786	2
17							K	**147.1128**	74.0600	130.0863	65.5468			1

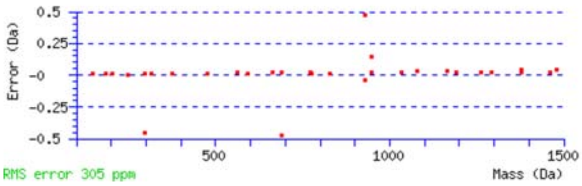

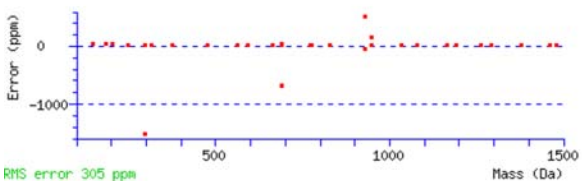

Peptide View

MS/MS Fragmentation of YGFSDPLTFNSVVELINHYR
Found in gi|33468921|ref|NP_035215.1|, phosphatidylinositol 3-kinase, regulatory subunit, polypeptide 1 isoform b [Mus musculus]

Match to Query 822: 2370.364872 from(791.128900,3+)

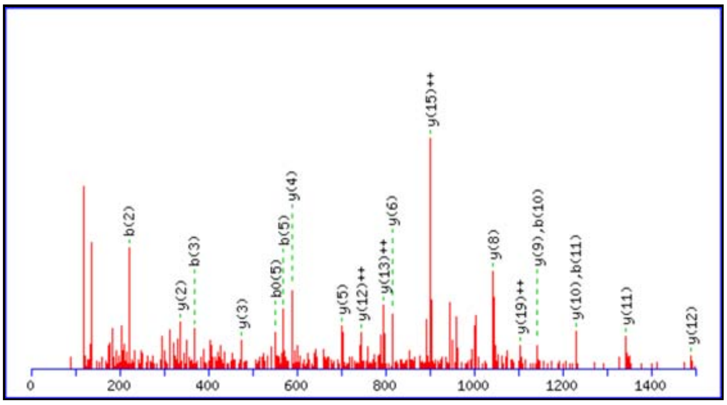

Monoisotopic mass of neutral peptide Mr(calc): 2370.1644
Ions Score: 58 **Expect:** 0.0096
Matches (**Bold Red**): 20/192 fragment ions using 34 most intense peaks

#	b	b^{++}	b*	$b*^{++}$	b^0	b^{0++}	Seq.	y	y^{++}	y*	$y*^{++}$	y^0	y^{0++}	#
1	164.0706	82.5389					Y							20
2	**221.0921**	111.0497					G	2208.1084	**1104.5578**	2191.0818	1096.0445	2190.0978	1095.5525	19
3	**368.1605**	184.5839					F	2151.0869	1076.0471	2134.0604	1067.5338	2133.0763	1067.0418	18
4	455.1925	228.0999			437.1819	219.0946	S	2004.0185	1002.5129	1986.9919	993.9996	1986.0079	993.5076	17
5	**570.2194**	285.6134			**552.2089**	276.6081	D	1916.9865	958.9969	1899.9599	950.4836	1898.9759	949.9916	16
6	667.2722	334.1397			649.2616	325.1345	P	1801.9595	**901.4834**	1784.9330	892.9701	1783.9490	892.4781	15
7	780.3563	390.6818			762.3457	381.6765	L	1704.9068	852.9570	1687.8802	844.4437	1686.8962	843.9517	14
8	881.4039	441.2056			863.3934	432.2003	T	1591.8227	**796.4150**	1574.7962	787.9017	1573.8121	787.4097	13
9	1028.4724	514.7398			1010.4618	505.7345	F	**1490.7750**	**745.8911**	1473.7485	737.3779	1472.7645	736.8859	12
10	**1142.5153**	571.7613	1125.4887	563.2480	1124.5047	562.7560	N	**1343.7066**	672.3569	1326.6801	663.8437	1325.6960	663.3517	11
11	**1229.5473**	615.2773	1212.5208	606.7640	1211.5368	606.2720	S	**1229.6637**	615.3355	1212.6371	606.8222	1211.6531	606.3302	10
12	1328.6157	664.8115	1311.5892	656.2982	1310.6052	655.8062	V	**1142.6317**	571.8195	1125.6051	563.3062	1124.6211	562.8142	9
13	1427.6841	714.3457	1410.6576	705.8324	1409.6736	705.3404	V	**1043.5632**	522.2853	1026.5367	513.7720	1025.5527	513.2800	8
14	1556.7267	778.8670	1539.7002	770.3537	1538.7162	769.8617	E	944.4948	472.7511	927.4683	464.2378	926.4843	463.7458	7
15	1669.8108	835.4090	1652.7842	826.8958	1651.8002	826.4037	L	**815.4522**	408.2298	798.4257	399.7165			6
16	1782.8948	891.9511	1765.8683	883.4378	1764.8843	882.9458	I	**702.3682**	351.6877	685.3416	343.1745			5
17	1896.9378	948.9725	1879.9112	940.4593	1878.9272	939.9672	N	**589.2841**	295.1457	572.2576	286.6324			4
18	2033.9967	1017.5020	2016.9701	1008.9887	2015.9861	1008.4967	H	**475.2412**	238.1242	458.2146	229.6110			3
19	2197.0600	1099.0336	2180.0335	1090.5204	2179.0495	1090.0284	Y	**338.1823**	169.5948	321.1557	161.0815			2
20							R	175.1190	88.0631	158.0924	79.5498			1

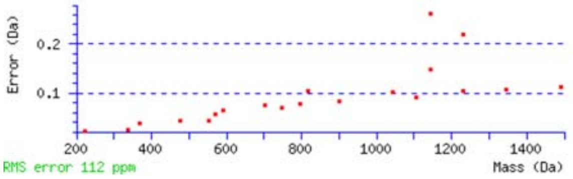

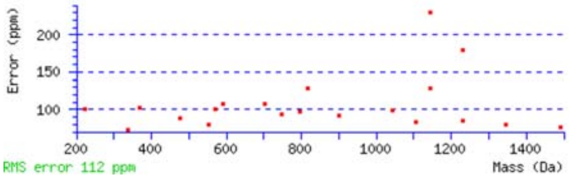

Peptide View

MS/MS Fragmentation of YMDDLQLVIEVFQK
Found in gi|46560563|ref|NP_035495.2|, SH3 domain protein 1B [Mus musculus]

Match to Query 416: 1739.943048 from(870.978800,2+)

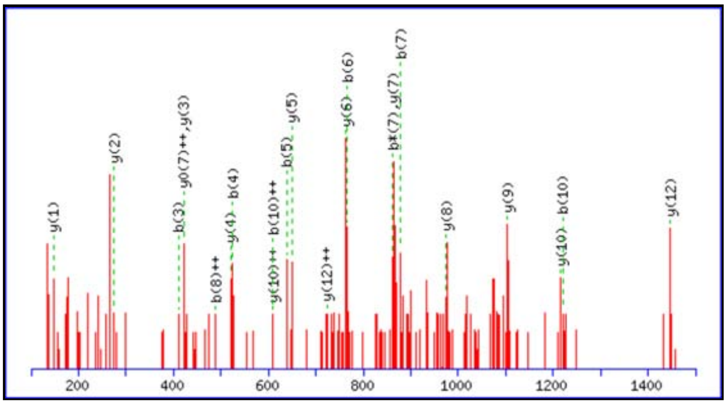

Monoisotopic mass of neutral peptide Mr(calc): 1739.8804
Ions Score: 50 Expect: 0.025
Matches (**Bold Red**): 23/134 fragment ions using 53 most intense peaks

#	b	b^{++}	b*	b^{*++}	b^0	b^{0++}	Seq.	y	y^{++}	y*	y^{*++}	y^0	y^{0++}	#
1	164.0706	82.5389					Y							14
2	295.1111	148.0592					M	1577.8243	789.4158	1560.7978	780.9025	1559.8138	780.4105	13
3	**410.1380**	205.5727			392.1275	196.5674	D	**1446.7838**	**723.8956**	1429.7573	715.3823	1428.7733	714.8903	12
4	**525.1650**	263.0861			507.1544	254.0808	D	1331.7569	666.3821	1314.7304	657.8688	1313.7463	657.3768	11
5	**638.2490**	319.6282			620.2385	310.6229	L	**1216.7300**	**608.8686**	1199.7034	600.3553	1198.7194	599.8633	10
6	**766.3076**	383.6574	749.2811	375.1442	748.2970	374.6522	Q	**1103.6459**	552.3266	1086.6194	543.8133	1085.6353	543.3213	9
7	**879.3917**	440.1995	**862.3651**	431.6862	861.3811	431.1942	L	**975.5873**	488.2973	958.5608	479.7840	957.5768	479.2920	8
8	978.4601	**489.7337**	961.4335	481.2204	960.4495	480.7284	V	**862.5033**	431.7553	845.4767	423.2420	844.4927	**422.7500**	7
9	1091.5441	546.2757	1074.5176	537.7624	1073.5336	537.2704	I	**763.4349**	382.2211	746.4083	373.7078	745.4243	373.2158	6
10	**1220.5867**	**610.7970**	1203.5602	602.2837	1202.5762	601.7917	E	**650.3508**	325.6790	633.3242	317.1658	632.3402	316.6738	5
11	1319.6551	660.3312	1302.6286	651.8179	1301.6446	651.3259	V	**521.3082**	261.1577	504.2817	252.6445			4
12	1466.7235	733.8654	1449.6970	725.3521	1448.7130	724.8601	F	**422.2398**	211.6235	405.2132	203.1103			3
13	1594.7821	797.8947	1577.7556	789.3814	1576.7716	788.8894	Q	**275.1714**	138.0893	258.1448	129.5761			2
14							K	**147.1128**	74.0600	130.0863	65.5468			1

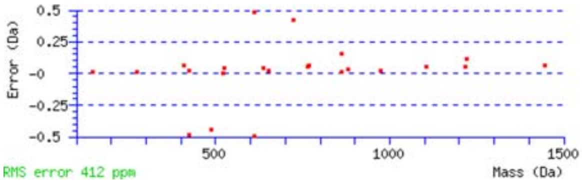

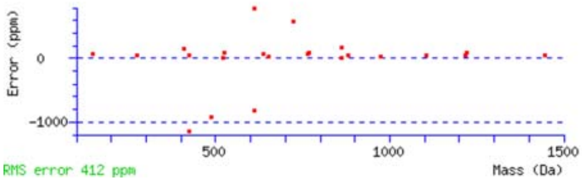

Peptide View

MS/MS Fragmentation of YTAVGQLVQDLLTQVR
Found in gi|21703974|ref|NP_663470.1|, Ras and Rab interactor 1 [Mus musculus]

Match to Query 1016: 1803.110648 from(902.562600,2+)

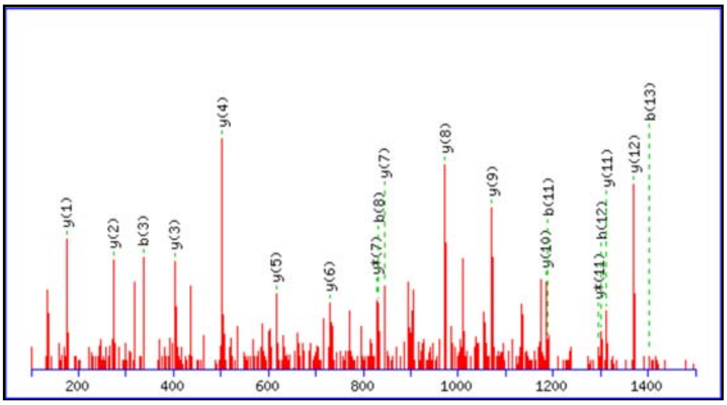

Monoisotopic mass of neutral peptide Mr(calc): 1802.9890
Ions Score: 78 Expect: 6.6e-005
Matches (Bold Red): 19/162 fragment ions using 34 most intense peaks

#	b	b^{++}	b*	b^{*++}	b^0	b^{0++}	Seq.	y	y^{++}	y*	y^{*++}	y^0	y^{0++}	#
1	164.0706	82.5389					Y							16
2	265.1183	133.0628			247.1077	124.0575	T	1640.9330	820.9701	1623.9064	812.4568	1622.9224	811.9648	15
3	**336.1554**	168.5813			318.1448	159.5761	A	1539.8853	770.4463	1522.8587	761.9330	1521.8747	761.4410	14
4	435.2238	218.1155			417.2132	209.1103	V	1468.8482	734.9277	1451.8216	726.4145	1450.8376	725.9224	13
5	492.2453	246.6263			474.2347	237.6210	G	**1369.7798**	685.3935	1352.7532	676.8802	1351.7692	676.3882	12
6	620.3038	310.6556	603.2773	302.1423	602.2933	301.6503	Q	**1312.7583**	656.8828	**1295.7318**	648.3695	1294.7477	647.8775	11
7	733.3879	367.1976	716.3614	358.6843	715.3773	358.1923	L	**1184.6997**	592.8535	1167.6732	584.3402	1166.6892	583.8482	10
8	**832.4563**	416.7318	815.4298	408.2185	814.4458	407.7265	V	**1071.6157**	536.3115	1054.5891	527.7982	1053.6051	527.3062	9
9	960.5149	480.7611	943.4883	472.2478	942.5043	471.7558	Q	**972.5473**	486.7773	955.5207	478.2640	954.5367	477.7720	8
10	1075.5418	538.2746	1058.5153	529.7613	1057.5313	529.2693	D	**844.4887**	422.7480	**827.4621**	414.2347	826.4781	413.7427	7
11	**1188.6259**	594.8166	1171.5993	586.3033	1170.6153	585.8113	L	**729.4617**	365.2345	712.4352	356.7212	711.4512	356.2292	6
12	**1301.7100**	651.3586	1284.6834	642.8453	1283.6994	642.3533	L	**616.3777**	308.6925	599.3511	300.1792	598.3671	299.6872	5
13	**1402.7576**	701.8825	1385.7311	693.3692	1384.7471	692.8772	T	**503.2936**	252.1504	486.2671	243.6372	485.2831	243.1452	4
14	1530.8162	765.9117	1513.7897	757.3985	1512.8057	756.9065	Q	**402.2459**	201.6266	385.2194	193.1133			3
15	1629.8846	815.4460	1612.8581	806.9327	1611.8741	806.4407	V	**274.1874**	137.5973	257.1608	129.0840			2
16							R	**175.1190**	88.0631	158.0924	79.5498			1

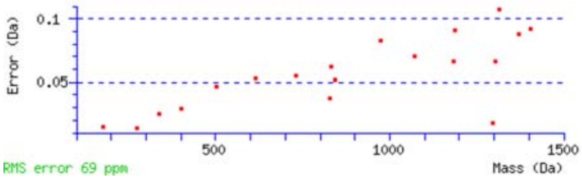

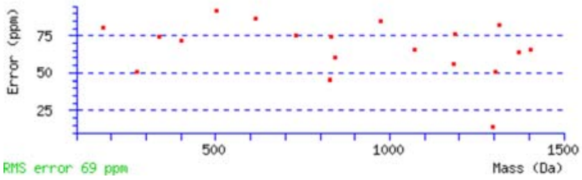

Peptide View

MS/MS Fragmentation of YVQFLSGLLSGAMK
Found in gi|94389897|ref|XP_109868.6|, PREDICTED: tensin 3 [Mus musculus]

Match to Query 476: 1512.813848 from(757.414200,2+)

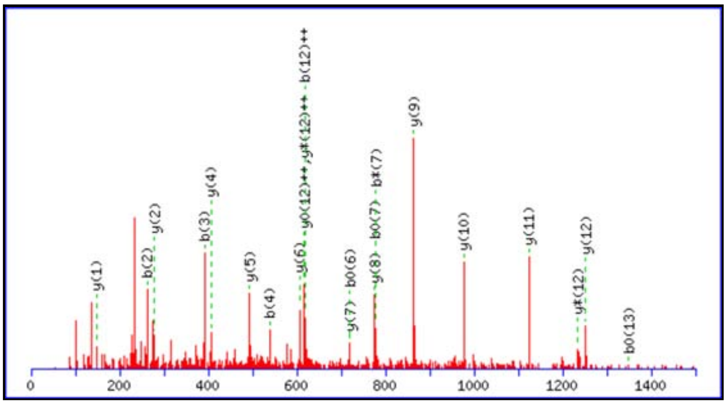

Monoisotopic mass of neutral peptide Mr(calc): 1512.8010
Ions Score: 68 **Expect:** 0.00029
Matches (Bold Red): 22/134 fragment ions using 52 most intense peaks

#	b	b^{++}	b^{*}	b^{*++}	b^{0}	b^{0++}	Seq.	y	y^{++}	y^{*}	y^{*++}	y^{0}	y^{0++}	#
1	164.0706	82.5389					Y							14
2	**263.1390**	132.0731					V	1350.7450	675.8761	1333.7184	667.3628	1332.7344	666.8708	13
3	**391.1976**	196.1024	374.1710	187.5892			Q	**1251.6765**	626.3419	**1234.6500**	**617.8286**	1233.6660	**617.3366**	12
4	**538.2660**	269.6366	521.2395	261.1234			F	**1123.6180**	562.3126	1106.5914	553.7993	1105.6074	553.3073	11
5	651.3501	326.1787	634.3235	317.6654			L	**976.5496**	488.7784	959.5230	480.2651	958.5390	479.7731	10
6	738.3821	369.6947	721.3555	361.1814	**720.3715**	360.6894	S	**863.4655**	432.2364	846.4389	423.7231	845.4549	423.2311	9
7	795.4036	398.2054	**778.3770**	389.6921	**777.3930**	389.2001	G	**776.4335**	388.7204	759.4069	380.2071	758.4229	379.7151	8
8	908.4876	454.7474	891.4611	446.2342	890.4771	445.7422	L	**719.4120**	360.2096	702.3855	351.6964	701.4014	351.2044	7
9	1021.5717	511.2895	1004.5451	502.7762	1003.5611	502.2842	L	**606.3279**	303.6676	589.3014	295.1543	588.3174	294.6623	6
10	1108.6037	554.8055	1091.5772	546.2922	1090.5931	545.8002	S	**493.2439**	247.1256	476.2173	238.6123	475.2333	238.1203	5
11	1165.6252	583.3162	1148.5986	574.8029	1147.6146	574.3109	G	**406.2119**	203.6096	389.1853	195.0963			4
12	1236.6623	**618.8348**	1219.6357	610.3215	1218.6517	609.8295	A	349.1904	175.0988	332.1638	166.5856			3
13	1367.7028	684.3550	1350.6762	675.8417	**1349.6922**	675.3497	M	**278.1533**	139.5803	261.1267	131.0670			2
14							K	**147.1128**	74.0600	130.0863	65.5468			1

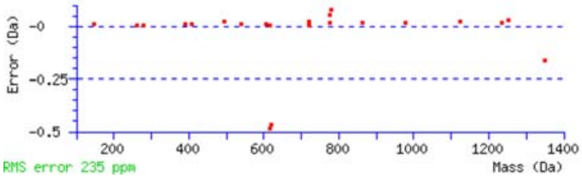

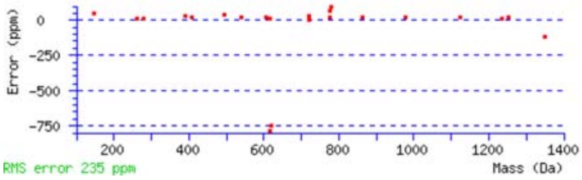

Supplement: Material S3 — Annotated MSMS spectra of peptides used for single peptide identification. (4.19 MB PDF) [file pone.0013926.s013.pdf]
